# Health effects of dietary risks in 195 countries, 1990–2017: a systematic analysis for the Global Burden of Disease Study 2017

**DOI:** 10.1016/S0140-6736(19)30041-8

**Published:** 2019-05-11

**Authors:** GBD 2017 Diet Collaborators, Ashkan Afshin, Patrick John Sur, Kairsten A. Fay, Leslie Cornaby, Giannina Ferrara, Joseph S Salama, Erin C Mullany, Kalkidan Hassen Abate, Cristiana Abbafati, Zegeye Abebe, Mohsen Afarideh, Anju Aggarwal, Sutapa Agrawal, Tomi Akinyemiju, Fares Alahdab, Umar Bacha, Victoria F Bachman, Hamid Badali, Alaa Badawi, Isabela M Bensenor, Eduardo Bernabe, Sibhatu Kassa K Biadgilign, Stan H Biryukov, Leah E Cahill, Juan J Carrero, Kelly M. Cercy, Lalit Dandona, Rakhi Dandona, Anh Kim Dang, Meaza Girma Degefa, Maysaa El Sayed Zaki, Alireza Esteghamati, Sadaf Esteghamati, Jessica Fanzo, Carla Sofia e Sá Farinha, Maryam S Farvid, Farshad Farzadfar, Valery L. Feigin, Joao C Fernandes, Luisa Sorio Flor, Nataliya A. Foigt, Mohammad H Forouzanfar, Morsaleh Ganji, Johanna M. Geleijnse, Richard F Gillum, Alessandra C Goulart, Giuseppe Grosso, Idris Guessous, Samer Hamidi, Graeme J. Hankey, Sivadasanpillai Harikrishnan, Hamid Yimam Hassen, Simon I. Hay, Chi Linh Hoang, Masako Horino, Farhad Islami, Maria D. Jackson, Spencer L. James, Lars Johansson, Jost B. Jonas, Amir Kasaeian, Yousef Saleh Khader, Ibrahim A. Khalil, Young-Ho Khang, Ruth W Kimokoti, Yoshihiro Kokubo, G Anil Kumar, Tea Lallukka, Alan D Lopez, Stefan Lorkowski, Paulo A. Lotufo, Rafael Lozano, Reza Malekzadeh, Winfried März, Toni Meier, Yohannes A Melaku, Walter Mendoza, Gert B.M. Mensink, Renata Micha, Ted R Miller, Mojde Mirarefin, Viswanathan Mohan, Ali H Mokdad, Dariush Mozaffarian, Gabriele Nagel, Mohsen Naghavi, Cuong Tat Nguyen, Molly R Nixon, Kanyin L Ong, David M. Pereira, Hossein Poustchi, Mostafa Qorbani, Rajesh Kumar Rai, Christian Razo-García, Colin D Rehm, Juan A Rivera, Sonia Rodríguez-Ramírez, Gholamreza Roshandel, Gregory A Roth, Juan Sanabria, Tania G Sánchez-Pimienta, Benn Sartorius, Josef Schmidhuber, Aletta Elisabeth Schutte, Sadaf G. Sepanlou, Min-Jeong Shin, Reed J.D. Sorensen, Marco Springmann, Lucjan Szponar, Andrew L Thorne-Lyman, Amanda G Thrift, Mathilde Touvier, Bach Xuan Tran, Stefanos Tyrovolas, Kingsley Nnanna Ukwaja, Irfan Ullah, Olalekan A Uthman, Masoud Vaezghasemi, Tommi Juhani Vasankari, Stein Emil Vollset, Theo Vos, Giang Thu Vu, Linh Gia Vu, Elisabete Weiderpass, Andrea Werdecker, Tissa Wijeratne, Walter C Willett, Jason H Wu, Gelin Xu, Naohiro Yonemoto, Chuanhua Yu, Christopher J L Murray

## Abstract

**Background:**

Suboptimal diet is an important preventable risk factor for non-communicable diseases (NCDs); however, its impact on the burden of NCDs has not been systematically evaluated. This study aimed to evaluate the consumption of major foods and nutrients across 195 countries and to quantify the impact of their suboptimal intake on NCD mortality and morbidity.

**Methods:**

By use of a comparative risk assessment approach, we estimated the proportion of disease-specific burden attributable to each dietary risk factor (also referred to as population attributable fraction) among adults aged 25 years or older. The main inputs to this analysis included the intake of each dietary factor, the effect size of the dietary factor on disease endpoint, and the level of intake associated with the lowest risk of mortality. Then, by use of disease-specific population attributable fractions, mortality, and disability-adjusted life-years (DALYs), we calculated the number of deaths and DALYs attributable to diet for each disease outcome.

**Findings:**

In 2017, 11 million (95% uncertainty interval [UI] 10–12) deaths and 255 million (234–274) DALYs were attributable to dietary risk factors. High intake of sodium (3 million [1–5] deaths and 70 million [34–118] DALYs), low intake of whole grains (3 million [2–4] deaths and 82 million [59–109] DALYs), and low intake of fruits (2 million [1–4] deaths and 65 million [41–92] DALYs) were the leading dietary risk factors for deaths and DALYs globally and in many countries. Dietary data were from mixed sources and were not available for all countries, increasing the statistical uncertainty of our estimates.

**Interpretation:**

This study provides a comprehensive picture of the potential impact of suboptimal diet on NCD mortality and morbidity, highlighting the need for improving diet across nations. Our findings will inform implementation of evidence-based dietary interventions and provide a platform for evaluation of their impact on human health annually.

**Funding:**

Bill & Melinda Gates Foundation.

## Introduction

The relationship between dietary habits and chronic non-communicable diseases (NCDs) has been extensively investigated.^1^, ^2^, ^3^, ^4^, ^5^ Long-term randomised trials with NCD endpoints have not been feasible for most dietary factors, but synthesis of other lines of epidemiological evidence, including long-term prospective observational studies and short-term trials of intermediate outcomes, have provided supporting evidence for potential causal relationships between specific dietary factors (eg, fruits, vegetables, processed meat, and trans fat intake) and NCDs (ischaemic heart disease, diabetes, and colorectal cancer).^2^, ^3^, ^4^, ^5^, ^6^, ^7^ These findings have been widely used to inform national and international dietary guidelines aimed at preventing NCDs.^8^, ^9^ However, because of the complexities of characterising dietary consumption across different nations, assessment of the health effects of suboptimal diet at the population level has not been possible.

In the past decade, efforts have been made to quantify the burden of disease attributable to specific dietary factors.^10^, ^11^, ^12^, ^13^, ^14^, ^15^, ^16^, ^17^, ^18^, ^19^ These efforts, although useful, had several important limitations, including insufficient geographically representative data on dietary consumption, inaccurate characterisation of population distribution of dietary intake, insufficient accounting for biases of different sources of dietary assessment, standardisation of the intake to 2000 kcal per day, and insufficient accounting for within-person variation of intake of dietary factors.

To address these limitations, as part of the Global Burden of Diseases, Injuries, and Risk Factors Study (GBD) 2017, we systematically collected geographically representative dietary data from multiple sources, characterised the population distribution of intake for 15 foods and nutrients among adults aged 25 years or older across 195 countries, estimated the effect of each individual dietary factor on NCD mortality, and quantified the overall impact of poor dietary habits on NCD mortality. We also evaluated the relationship between diet and socioeconomic development, and assessed the trends in disease burden of diet over time. This analysis supersedes all previous results from GBD with respect to dietary risks by comprehensively reanalysing all data from 1990 to 2017, using consistent methods and definitions.

**Research in context**

**Evidence before this study**

We systematically searched MEDLINE and the Global Health Data Exchange (GHDx) to identify studies providing nationally or subnationally representative estimates of consumption of 15 foods and nutrients. We included only studies reporting data collected between Jan 1, 1980, and Dec 31, 2016, in one of the 195 countries included in this analysis. Studies were excluded if done with non-random samples or among specific subpopulations. We estimated the potential health effects of each dietary risk by use of the Global Burden of Diseases, Injuries, and Risk Factors Study comparative risk assessment approach.

**Added value of this study**

This study provides a comprehensive picture of consumption of 15 dietary factors across nations and quantifies the potential impact of suboptimal intake of each diet component on chronic disease mortality and morbidity among 195 countries. Additionally, this study characterises the relationship between diet and development and evaluates the trends in the burden of disease attributable to diet from 1990 to 2017. High intake of sodium, low intake of whole grains, and low intake of fruits were the leading dietary risk factors for deaths and DALYs globally and in many countries.

**Implications of all the available evidence**

This study highlights the need for improving diet at the global, regional, and national level. The findings inform priorities for population-level interventions to improve diet.

## Methods

### Selection of dietary risk factors

We selected 15 dietary risk factors (table) that met GBD selection criteria for risk factors.^10^, ^11^, ^12^, ^13^ These criteria include the importance of the risk factor to either disease burden or policy; the availability of sufficient data to estimate risk factor exposure; the strength of the epidemiological evidence supporting a causal relationship between risk factor exposure and disease endpoints, and availability of data to quantify the magnitude of this relationship per unit of change in the exposure; and evidence supporting the generalisability of the effects to all populations. The process of evaluation of the strength of epidemiological evidence for the causal relationship of each diet–disease pair is described elsewhere^10^, ^11^, ^12^, ^13^ and summarised in the appendix.

**Table:** Dietary risk factor exposure definitions, optimal level, and data representativeness index, 1990–2017

	**Exposure definition**	**Optimal level of intake (optimal range of intake)**	**Data representativeness index (%)**
Diet low in fruits	Mean daily consumption of fruits (fresh, frozen, cooked, canned, or dried fruits, excluding fruit juices and salted or pickled fruits)	250 g (200–300) per day	94·9
Diet low in vegetables	Mean daily consumption of vegetables (fresh, frozen, cooked, canned, or dried vegetables, excluding legumes and salted or pickled vegetables, juices, nuts, seeds, and starchy vegetables such as potatoes or corn)	360 g (290–430) per day	94·9
Diet low in legumes	Mean daily consumption of legumes (fresh, frozen, cooked, canned, or dried legumes)	60 g (50–70) per day	94·9
Diet low in whole grains	Mean daily consumption of whole grains (bran, germ, and endosperm in their natural proportion) from breakfast cereals, bread, rice, pasta, biscuits, muffins, tortillas, pancakes, and other sources	125 g (100–150) per day	94·9
Diet low in nuts and seeds	Mean daily consumption of nut and seed foods	21 g (16–25) per day	94·9
Diet low in milk	Mean daily consumption of milk including non-fat, low-fat, and full-fat milk, excluding soy milk and other plant derivatives	435 g (350–520) per day	94·9
Diet high in red meat	Mean daily consumption of red meat (beef, pork, lamb, and goat, but excluding poultry, fish, eggs, and all processed meats)	23 g (18–27) per day	94·9
Diet high in processed meat	Mean daily consumption of meat preserved by smoking, curing, salting, or addition of chemical preservatives	2 g (0–4) per day	36·9
Diet high in sugar-sweetened beverages	Mean daily consumption of beverages with ≥50 kcal per 226·8 serving, including carbonated beverages, sodas, energy drinks, fruit drinks, but excluding 100% fruit and vegetable juices	3 g (0–5) per day	36·9
Diet low in fibre	Mean daily intake of fibre from all sources including fruits, vegetables, grains, legumes, and pulses	24 g (19–28) per day	94·9
Diet low in calcium	Mean daily intake of calcium from all sources, including milk, yogurt, and cheese	1·25 g (1·00–1·50) per day	94·9
Diet low in seafood omega-3 fatty acids	Mean daily intake of eicosapentaenoic acid and docosahexaenoic acid	250 mg (200–300) per day	94·9
Diet low in polyunsaturated fatty acids	Mean daily intake of omega-6 fatty acids from all sources, mainly liquid vegetable oils, including soybean oil, corn oil, and safflower oil	11% (9–13) of total daily energy	94·9
Diet high in trans fatty acids	Mean daily intake of trans fat from all sources, mainly partially hydrogenated vegetable oils and ruminant products	0·5% (0·0–1·0) of total daily energy	36·9
Diet high in sodium	24 h urinary sodium measured in g per day	3 g (1–5) per day*	26·2

* To reflect the uncertainty in existing evidence on optimal level of intake for sodium, 1–5 g per day was considered as the uncertainty range for the optimal level of sodium where less than 2·3 g per day is the intake level of sodium associated with the lowest level of blood pressure in randomised controlled trials and 4–5 g per day is the level of sodium intake associated with the lowest risk of cardiovascular disease in observational studies.

### Dietary intake at the population level

We did a systematic review of the scientific literature to identify nationally or subnationally representative nutrition surveys providing data on consumption of each dietary factor (appendix). We also searched the Global Health Data Exchange website for nationally or subnationally representative nutrition surveys and household budget surveys. Additionally, for food groups, we used national sales data from Euromonitor and national availability data from United Nations Food and Agriculture Organization food balance sheets. For nutrients, we used data on their national availability from the Global Nutrient Database.^20^ For sodium, we collected data on 24 h urinary sodium, where available. For trans fat, we used sales data from Euromonitor on hydrogenated vegetable oil. The list of all dietary data sources used in GBD 2017 is publicly available at the Global Health Data Exchange website. For each dietary factor, we computed a data representativeness index as the fraction of countries for which we identified any data on the risk factor exposure (table).

Our dietary data were from multiple sources and were affected by different types of biases. We considered 24 h diet recall as the gold standard method for assessing mean intake at the population level and adjusted dietary data from other sources accordingly (appendix). Some types of dietary data (ie, availability, sales, and household data) were only available for all-age groups and both sexes. To split these data into standard age-specific and sex-specific groups, we first estimated the global age and sex patterns of intake using data from nutrition surveys and then used those patterns to split the availability, sales, and household data.

We used the spatiotemporal Gaussian process regression method to estimate the mean intake of each dietary risk factor by age, sex, country, and year (appendix). To improve our estimates in data-sparse models, we tested a wide range of covariates with plausible relationships with intake and included the covariates with best fit and coefficients in the expected direction (appendix).

### Effect size of dietary risks on disease endpoints

For each diet–disease pair, we used data from published meta-analyses of prospective observational studies to estimate the relative risk of mortality and morbidity.^21^ For diet–disease pairs for which evidence was only available on morbidity, we assumed that the estimated relative risks were also applied to mortality (appendix). Considering the relationship of diet and metabolic risk factors and the well established age trend of the relative risks of metabolic risks for cardiovascular disease and type 2 diabetes, we used the age trend of the relative risks of metabolic risk factors^22^ to estimate the age-specific relative risk of dietary risks for cardiovascular disease and type 2 diabetes (appendix). To estimate the impact of sodium on outcomes, we first estimated the relationship between urinary sodium and change in systolic blood pressure, and then estimated the relationship between change in systolic blood pressure and disease outcomes.^14^

### Optimal level of intake

We defined the optimal level of intake as the level of risk exposure that minimises the risk from all causes of death. To estimate the optimal intake for each dietary factor, we first calculated the level of intake associated with the lowest risk of mortality from each disease endpoint based on the studies included in the meta-analyses of the dietary relative risks. Then, we calculated the optimal level of intake as the weighted mean of these numbers using the global proportion of deaths from each disease as the weight. To reflect the uncertainty of optimal level of intake, we assumed a uniform uncertainty distribution of 20% above and below the mean.^13^ For sodium, the evidence supporting the selection of the optimal level of intake was uncertain.^23^, ^24^ Therefore, we included a uniform distribution of different optimal levels of intake in the uncertainty estimation sampling.

### Disease-specific deaths and disability-adjusted life-years

Data on disease-specific deaths and disability-adjusted life-years (DALYs) by age, sex, country, and year were obtained from GBD 2017. The GBD approach to estimating cause-specific mortality and DALYs has been described in detail elsewhere.^25^, ^26^

### Disease burden of dietary risks

We used the GBD comparative risk assessment approach to estimate the population attributable fraction for each diet–disease pair by age, sex, country, and year.^10^, ^11^, ^12^, ^13^ Then, we estimated the number of deaths and DALYs attributable to each dietary risk factor by multiplying the population attributable fraction by the total number of disease-specific deaths and DALYs.

To position countries on the development continuum, we used the Socio-demographic Index (SDI), which is a summary measure calculated on the basis of lag-distributed income per capita, mean educational attainment of individuals aged 15 years or older, and total fertility rate among women younger than 25 years.^12^, ^13^ To estimate gaps in intake or excess of intake of individual components of diet, we compared the current intake of each dietary factor with the midpoint of its optimal range of intake (table). High intake of a dietary component refers to an intake level higher than the midpoint of the optimal range of intake, and low intake refers to an intake level lower than the midpoint of the optimal range of intake.

To incorporate the uncertainty of parameters (exposure, relative risk, optimal level of intake, and mortality) as well as modelling uncertainty, we followed a Monte Carlo approach. We repeated all calculations 1000 times using one draw of each parameter at each iteration. Using these 1000 draws, we calculated the mean and 95% uncertainty interval (UI) for the final estimates.

All statistical analyses were done in Python, version 3.5.

### Role of the funding source

The funder of the study had no role in study design, data collection, data analysis, data interpretation, or writing of the report. The first author and the corresponding author had full access to all the data in the study and had final responsibility for the decision to submit for publication.

## Results

### Consumption of major foods and nutrients

**Figure 1:**
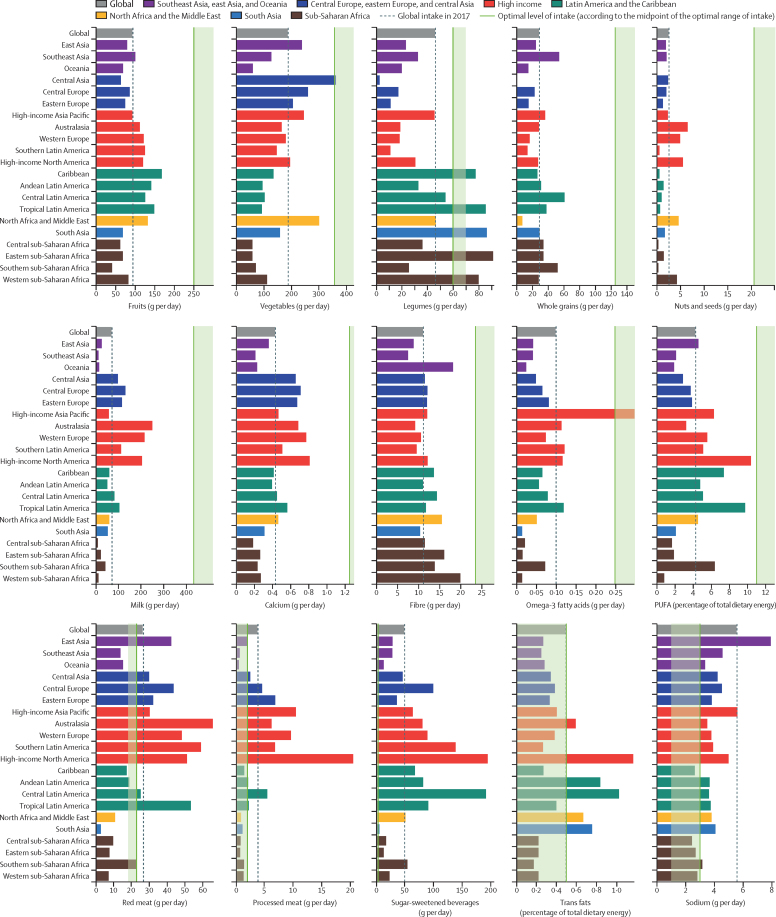
Age-standardised intake of dietary factors among adults aged 25 years or older at the global and regional level in 2017

Globally, consumption of nearly all healthy foods and nutrients was suboptimal in 2017 (figure 1). The largest gaps between current and optimal intake were observed for nuts and seeds, milk, and whole grains, with mean consumption at 12% (95% UI 12–13; 3 g [2–3] of nuts and seeds per day), 16% (16–17; 71 g [70–72] of milk per day), and 23% (23–23; 29 g [29–29] of whole grains per day) of the optimal levels (percentages calculated on the basis of data before rounding). In parallel with suboptimal healthy food consumption, daily intake of all unhealthy foods and nutrients exceeded the optimal level globally (figure 1). The consumption of sugar-sweetened beverages (49 g per day) was far higher than the optimal intake. Similarly, global consumption of processed meat (4 g [4–4] per day, 90% greater than the optimal amount) and sodium (6 g [5–6] per day, 86% greater than the optimal amount) were far above the optimal levels. The global intake of red meat (27 g [26–28] per day) was 18% greater than the optimal intake. Men generally had a higher intake of both healthy and unhealthy foods than did women. Intake of both healthy and unhealthy foods was generally higher among middle-aged adults (50–69 years) and lowest among young adults (25–49 years) with a few exceptions. The highest intake of sugar-sweetened beverages and legumes were observed among young adults and showed a decreasing trend with age.

At the regional level, in 2017, the intake of all healthy foods was lower than the optimal level in all 21 GBD regions (figure 1). The only exceptions were the intake of vegetables in central Asia, seafood omega-3 fatty acids in high-income Asia Pacific, and legumes in the Caribbean, tropical Latin America, south Asia, western sub-Saharan Africa, and eastern sub-Saharan Africa. Among unhealthy food groups, consumption of sodium and sugar-sweetened beverages were higher than the optimal level in nearly every region. Red meat consumption was highest in Australasia, southern Latin America, and tropical Latin America. High-income North America had the highest processed meat intake followed by high-income Asia Pacific and western Europe. The highest intake of trans fats was observed in high-income North America, central Latin America, and Andean Latin America.

### Overall impact of diet on mortality

Globally, in 2017, dietary risks were responsible for 11 million [95% UI 10–12] deaths (22% [95% UI 21–24] of all deaths among adults) and 255 million (234–274) DALYs (15% [14–17] of all DALYs among adults; appendix). Cardiovascular disease was the leading cause of diet-related deaths (10 million [9–10] deaths) and DALYs (207 million [192–222] DALYs), followed by cancers (913 090 [743 345–1 098 432] deaths and 20 million [17–24] DALYs) and type 2 diabetes (338 714 deaths [244 995–447 003] and 24 million [16–33] DALYs). More than 5 million (95% UI 5–5) diet-related deaths (45% [43–46] of total diet-related deaths) and 177 million (163–192) diet-related DALYs (70% [68–71] of total diet-related DALYs) occurred among adults aged younger than 70 years.

Across the 21 GBD regions, in 2017, the highest age-standardised rates of all diet-related deaths and DALYs among adults aged 25 years or older were observed in Oceania (678 [95% UI 616–746] deaths per 100 000 population and 17 804 [16 041–19 907] DALYs per 100 000 population; appendix). The lowest rates of all diet-related deaths among adults (aged 25 years or older) were observed in high-income Asia Pacific (97 [89–106] deaths per 100 000 population) and the lowest rates of all diet-related DALYs were observed in Australasia (2182 [1955–2444] DALYs per 100 000 population). The regions with the highest rates of diet-related cardiovascular disease deaths and DALYs were central Asia (613 [566–658] deaths per 100 000 population) and Oceania (14 755 [13 212–16 512] DALYs per 100 000 population), whereas the lowest rate of cardiovascular disease deaths and DALYs were observed in high-income Asia Pacific (68 [63–75] deaths per 100 000 population and 1443 [1329–1573] DALYs per 100 000 population). Diet-related cancer death and DALY rates were highest in east Asia (41 [34–49] deaths per 100 000 population and 878 [736–1023] DALYs per 100 000 population) and lowest in north Africa and the Middle East (nine [8–11] deaths per 100 000 population and 203 [169–243] DALYs per 100 000 population). Oceania (60 [44–78] deaths per 100 000 population and 2426 [1737–3198] DALYs per 100 000 population) had the highest age-standardised rate of diet-related diabetes deaths and DALYs, and high-income Asia Pacific had the lowest rates (two [2–3] deaths per 100 000 population and 290 [202–395] DALYs per 100 000 population). In 2017, the highest age-standardised proportions of diet-related deaths and DALYs from cardiovascular disease were observed in Oceania (60% [95% UI 56–63] of deaths) and east Asia (64% [60–68] of DALYs), those from cancer in east Asia (15% [13–18] of deaths and 15% [12–17] of DALYs), and those from type 2 diabetes in high-income North America (41% [34–48] of deaths and 50% [42–58] of DALYs; appendix). The lowest age-standardised proportions of deaths and DALYs from these causes were in western Europe (42% [38–45] of deaths and 44% [41–47] of DALYs), western sub-Saharan Africa (5% [4–6] of deaths and 4% [4–5] DALYs), and southeast Asia (29% [20–38] of deaths and 35% [25–46] of DALYs).

**Figure 2:**
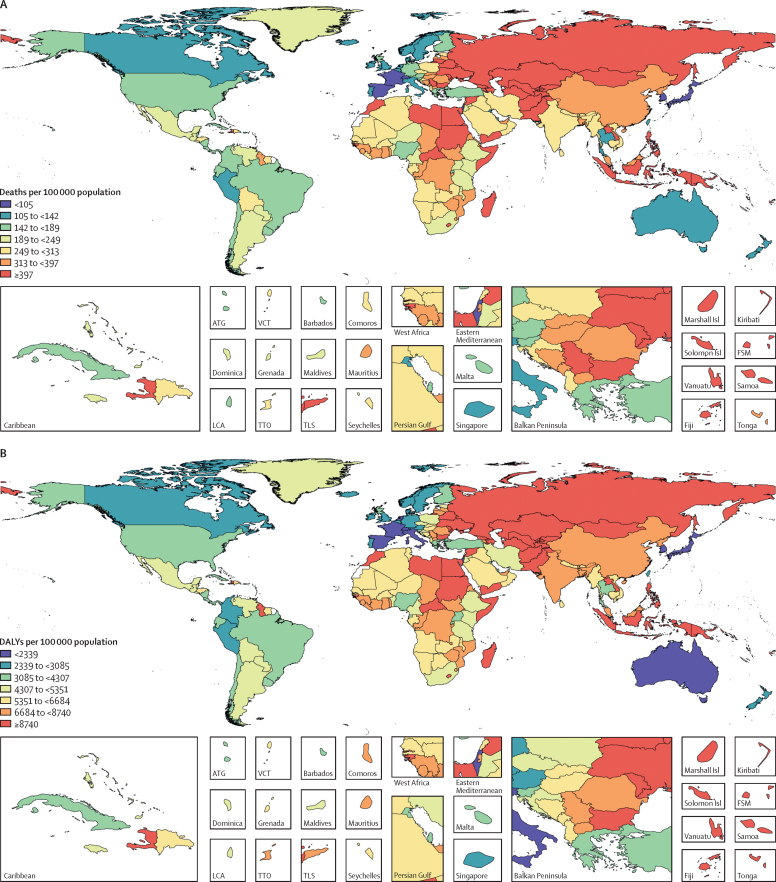
Age-standardised mortality rate per 100 000 population (A) and DALY rate per 100 000 population (B) attributable to diet in 2017 ATG=Antigua and Barbuda. Isl=Islands. FSM=Federated States of Micronesia. LCA=Saint Lucia. TLS=Timor-Leste. TTO=Trinidad and Tobago. VCT=Saint Vincent and the Grenadines.

In 2017, among the world's 20 most populous countries, Egypt had the highest age-standardised rate of all diet-related deaths (552 [95% UI 490–620] deaths per 100 000 population) and DALYs (11 837 [10 525–13 268] DALYs per 100 000 population) and Japan had the lowest rate of all diet-related deaths (97 [89–106)] deaths per 100 000 population) and DALYs (2300 [2099–2513] DALYs per 100 000 population; figure 2). China had the highest age-standardised rates of diet-related cardiovascular disease deaths (299 [275–324] deaths per 100 000 population) and Egypt had the highest DALY rates (10 811 [9577–12 209] DALYs per 100 000 population). China had highest rates of diet-related cancer deaths and DALYs (42 [34–49] deaths per 100 000 population and 889 [744–1036] DALYs per 100 000 population), and Mexico had the highest rates of diet-related type 2 diabetes deaths and DALYs (35 [28–44] deaths per 100 000 population and 1605 [1231–2034] DALYs per 100 000 population). Japan had the lowest rate of diet-related cardiovascular disease deaths and DALYs (69 [63–75] deaths per 100 000 population and 1507 [1389–1639] DALYs per 100 000 population) and diabetes deaths and DALYs (one [1–1] death per 100 000 population and 234 [161–321] DALYs per 100 000 population). Egypt had the lowest rate of diet-related cancer deaths and DALYs (five [4–6] deaths per 100 000 population and 120 [96–146] DALYs per 100 000 population; appendix). The highest age-standardised proportion of all diet-related deaths (30% [27–33]) and DALYs (23% [21–25]) in adults aged 25 years or older were observed in Egypt, and the lowest proportion of all diet-related deaths (11% [9–12]) and DALYs (7% [6–8]) in the same age group were observed in Nigeria (appendix). The highest proportions of diet-related cardiovascular disease deaths and DALYs in 2017 were observed in Pakistan (60% [95% UI 57–64] of deaths and 66% [62–69] of DALYs), cancer deaths and DALYs in China (16% [13–18] of deaths and 15% [13–17] of DALYs), and type 2 diabetes deaths and DALYs in the USA (41% [34–49] of deaths and 50% [43–58] of DALYs). The lowest proportions of cardiovascular disease deaths and DALYs were seen in Turkey (42% [38–47] of deaths and 44% [40–49] of DALYs), cancer deaths and DALYs in Egypt (4% [3–4] of deaths and 3% [3–4] of DALYs), and type 2 diabetes deaths and DALYs in Bangladesh (25% [17–34] of deaths and 34% [23–45] of DALYs).

### Impact of individual components of diet on mortality

**Figure 3:**
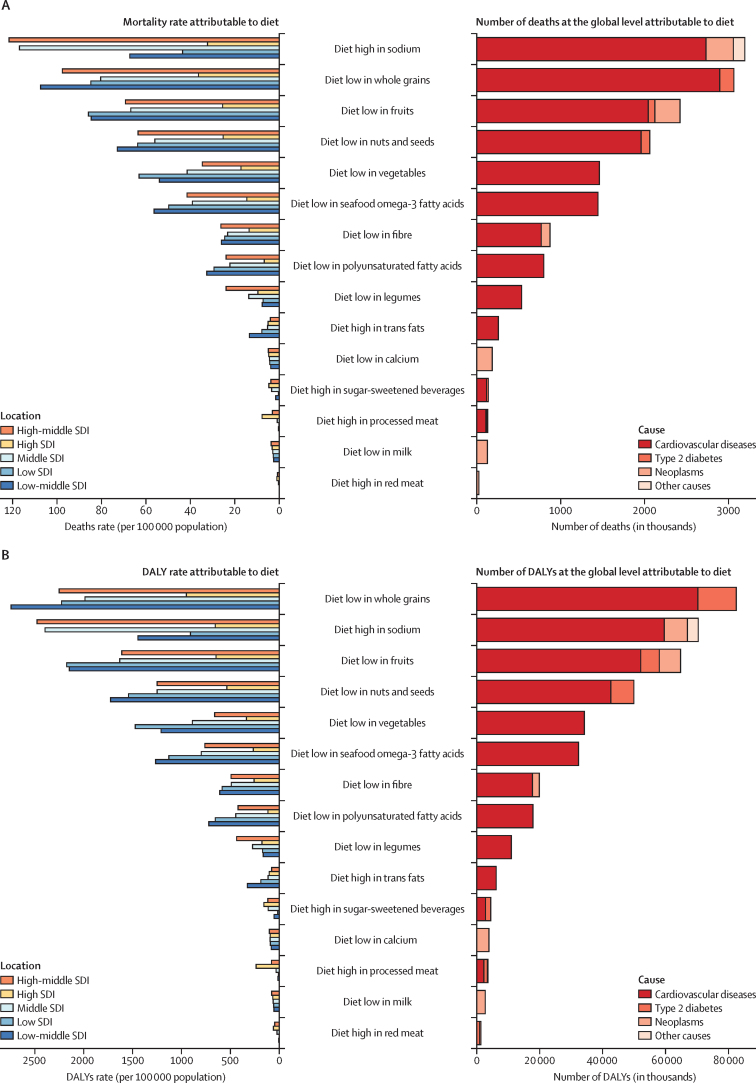
Number of deaths and DALYs and age-standardised mortality rate and DALY rate (per 100 000 population) attributable to individual dietary risks at the global and SDI level in 2017 DALY=disability-adjusted life-year. SDI=Socio-demographic Index.

A small number of dietary risks had a large impact on health outcomes. In 2017, more than half of diet-related deaths and two-thirds of diet-related DALYs were attributable to high intake of sodium (3 million [95% UI 1–5] deaths and 70 million [34–118] DALYs), low intake of whole grains (3 million [2–4] deaths and 82 million [59–109] DALYs), and low intake of fruits (2 million [1–4] deaths and 65 million [41–92] DALYs; figure 3). Low intake of whole grains was the leading dietary risk factor for DALYs among men and women and the leading dietary risk factor for mortality among women. Sodium ranked first for mortality among men followed by whole grains and fruit. Low intake of whole grains was the leading risk for deaths and DALYs among young adults (aged 25–50 years) and sodium ranked first among older adults (≥70 years).

**Figure 4:**
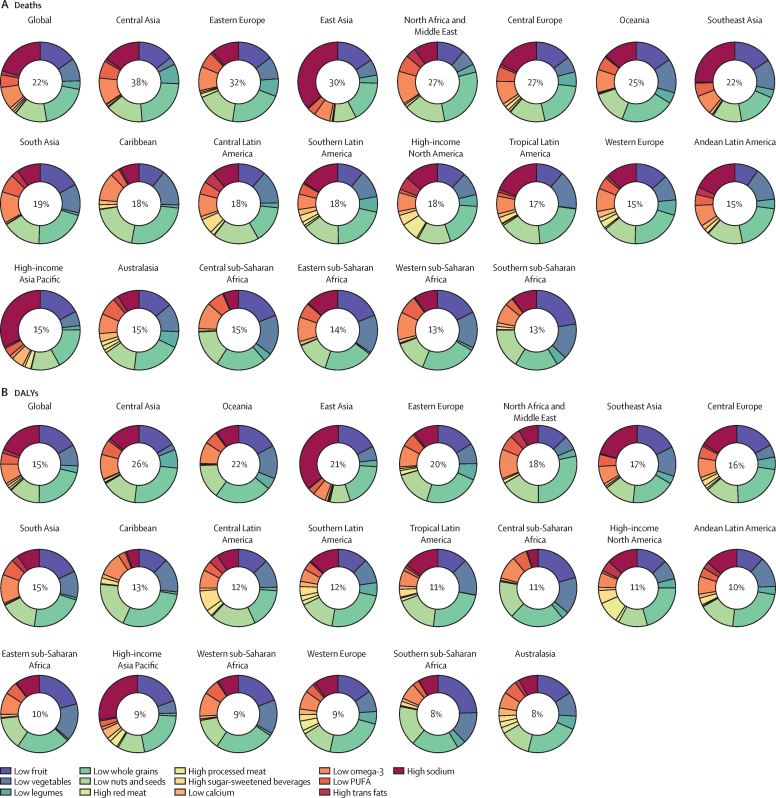
Age-standardised proportions of deaths and DALYs attributable to individual dietary risks at the global and regional level in 2017 DALYs=disability-adjusted life-years.

In 2017, across the 21 GBD regions, a diet low in whole grains was the most common leading dietary risk factor for deaths (in 16 regions) and DALYs (in 17 regions; figure 4). A diet high in sodium was the leading dietary risk factor for deaths and DALYs in east Asia and high-income Asia Pacific regions (appendix). In southern sub-Saharan Africa, a diet low in fruits and in central Latin America a diet low in nuts and seeds were the dietary risk factors responsible for the greatest proportion of deaths and DALYs in 2017.

High intake of sodium was the leading dietary risk for deaths and DALYs in China, Japan, and Thailand. Low intake of whole grains was the leading dietary risk factor for deaths and DALYs in the USA, India, Brazil, Pakistan, Nigeria, Russia, Egypt, Germany, Iran, and Turkey. In Bangladesh, low intake of fruits was the leading dietary risk associated with deaths and DALYs. In Mexico, low intake of nuts and seeds ranked first for diet-related deaths and DALYs. High consumption of red meat, processed meat, trans fat, and sugar-sweetened beverages were towards the bottom in ranking of dietary risks for deaths and DALYs for most high-population countries (appendix).

### Relationship between diet and SDI

Overall, in 2017, the highest age-standardised rates of all diet-related deaths and DALYs were observed in low-middle SDI countries (344 [95% UI 319–369] deaths per 100 000 population and 7797 [7265–8386] DALYs per 100 000 population) and high-middle SDI countries (347 [324–369] deaths per 100 000 population and 6998 [6534–7454] DALYs per 100 000 population; appendix). The lowest burden of exposure to dietary risk was observed in high SDI countries (139 [129–148] deaths per 100 000 population and 3032 [2802–3265] DALYs per 100 000 population). Low-middle SDI had the highest age-standardised rates of diet-related deaths and DALYs for cardiovascular disease (311 [288–335] deaths per 100 000 population and 6685 [6228–7161] DALYs per 100 000 population) and diabetes (14 [10–18] deaths per 100 000 population and 681 [477–914] DALYs per 100 000 population). High-middle SDI had the highest age-standardised rates of diet-related mortality for cancer (29 [24–34] deaths per 100 000 population and 630 [529–731] DALYs per 100 000 population). The lowest age-standardised rate of diet-related deaths and DALYs for cardiovascular disease (113 [104–122] deaths per 100 000 population and 2156 [2005–2306] DALYs per 100 000 population) and diabetes (five [4–6] deaths per 100 000 population and 444 [324–587] DALYs per 100 000 population) was observed in high SDI countries and lowest mortality rate for cancer was observed in low SDI countries (15 [12–17] deaths per 100 000 population and 324 [268–376] DALYs per 100 000 population). The highest proportions of diet-related deaths and DALYs for all causes were observed in high-middle SDI countries (29% [95% UI 27–31] of deaths and 19% [17–21] of DALYs), the lowest proportion of diet-related deaths was observed in low SDI countries (16% [15–17] of deaths), and the lowest proportion of DALYs was observed in high SDI countries (10% [9–11] of DALYs; appendix). Dietary risks were responsible for 55% [51–59] of cardiovascular disease deaths and 60% [56–63] of DALYs in middle SDI countries, and 46% [42–49] of cardiovascular disease deaths and 49% [46–52] of cardiovascular disease DALYs in high SDI countries. Middle SDI countries had the highest proportion of cancer deaths (12% [10–14]) and DALYs (11% [9–13]) and high SDI countries had the lowest proportion of attributable cancer deaths (8% [7–9]) and DALYs (7% [6–9]). The highest burden of diabetes attributable to diet was observed in high SDI countries (35% [28–43] of deaths and 46% [38–55) of DALYs) and lowest attributable burden was observed in the low SDI countries (31% [22–39] of deaths and 39% [29–50] of DALYs).

High-middle and middle SDI countries were at the greatest risk of deaths and DALYs from high consumption of sodium, whereas high and low-middle SDI countries had the greatest risk caused by a diet low in whole grains (figure 3). In low SDI countries, low intake of fruit was the leading dietary risk for deaths and low intake of whole grains was the leading dietary risk for DALYs. Countries at all levels of SDI other than low SDI had the same four leading dietary risks: high sodium, low whole grains, low fruit, and low nuts and seeds. The four leading dietary risks for low SDI countries were a diet in low whole grains, low in fruit, low in nuts and seeds, and low in vegetables.

### Impact of nutrition transition on exposure to dietary risks

Since 1990, the number of deaths (8 million [95% UI 7–8] deaths) and DALYs (184 [172–197] DALYs) attributable to dietary risks significantly increased to 11 million (10–12) deaths and 255 million (234–274) DALYs in 2017 (appendix). The main contributors to this increase were population growth and population ageing. After removing the effect of population growth and population ageing, the age-standardised attributable death and DALY rates showed a significant decrease between 1990 and 2017; from 406 (381–430) deaths per 100 000 population to 275 (258–292) deaths per 100 000 population, and from 8536 (8063–9013) DALYs per 100 000 population to 6080 (5685–6472) DALYs per 100 000 population. This decrease seemed to be driven mostly by decreases in the background mortality rate because, during the same period, the proportion of deaths and DALYs related to dietary risk remained relatively stable.

## Discussion

Our systematic evaluation of dietary consumption patterns across 195 countries provides a comprehensive picture of the health effects of poor dietary habits at the population level. We found that improvement of diet could potentially prevent one in every five deaths globally. Our findings show that, unlike many other risk factors, dietary risks affected people regardless of age, sex, and sociodemographic development of their place of residence. Although the impact of individual dietary factors varied across countries, non-optimal intake of three dietary factors (whole grains, fruits, and sodium) accounted for more than 50% of deaths and 66% of DALYs attributable to diet.

Our findings show that suboptimal diet is responsible for more deaths than any other risks globally, including tobacco smoking,^11^, ^12^ highlighting the urgent need for improving human diet across nations. Although sodium, sugar, and fat have been the main focus of diet policy debate in the past two decades,^27^, ^28^ our assessment shows that the leading dietary risk factors for mortality are diets high in sodium, low in whole grains, low in fruit, low in nuts and seeds, low in vegetables, and low in omega-3 fatty acids; each accounting for more than 2% of global deaths. This finding suggests that dietary policies focusing on promoting the intake of components of diet for which current intake is less than the optimal level might have a greater effect than policies only targeting sugar and fat, highlighting the need for a comprehensive food system interventions to promote the production, distribution, and consumption of these foods across nations.

Over the past decade, the effectiveness of a range of population-level dietary interventions has been systematically evaluated and several promising interventions have been identified.^29^, ^30^, ^31^ These include mass media campaigns, food and menu labeling, food pricing strategies (subsidies and taxation), school procurement policies, and worksite wellness programmes. Cost-effectiveness analyses of these interventions have shown that targeting specific dietary factors (eg, sodium) might not only be cost-effective but cost-saving.^32^, ^33^, ^34^, ^35^ However, improvement of diet through population-level interventions faces several major challenges. First, the observed effects for most of these dietary interventions are far below the level required to achieve optimal diet globally.^29^, ^30^ Second, there is almost no evidence on the effectiveness of these interventions on several important dietary factors (ie, nuts, whole grains, seafood, red meat, and processed meat). Third, cost-effectiveness analyses of dietary interventions are generally based on a range of simplifying assumptions and do not take into account the reactions of consumers (eg, substitution effect), the food industry (eg, food reformulations and pricing strategies), and other stakeholders in the real world.^32^, ^33^, ^34^, ^35^ Fourth, despite the growing public and political will for the implementation of some of these policies (eg, trans fat bans), few countries have successfully adopted and implemented them.^36^, ^37^ Fifth, many of these policies only target consumers but not the wide range of interconnected factors, such as food production, processing, and distribution, that exist throughout the food system. Indeed, these factors might affect dietary consumption, and it is important to include them to improve diet.^38^, ^39^ Therefore, in view of the magnitude of the disease burden attributable to diet and the limitations of the existing interventions, development of novel food system interventions is urgently needed.

Our results show a need for extensive changes in various sectors of the food system at the global, regional, and national levels to improve diet. Changes in agricultural practices, if not done properly, might raise concerns over potential environmental effects on climate change, biodiversity loss, degradation of land and soil, and freshwater depletion.^40^, ^41^, ^42^, ^43^ A growing body of evidence has emerged in the past decade showing that shifting diet from unhealthy animal-based foods (eg, red meat and processed meat) to healthy plant-based foods (eg, fruits, vegetables, and whole grains) might be associated with lower emission of greenhouse gases and thus might be more environmentally sustainable.^40^, ^41^, ^42^, ^43^ The few studies evaluating other environmental effects of the shift from animal-based to plant-based diet have also demonstrated that this shift might be associated with lower land use and water footprint.^41^ However, because of the variations in the methods and research questions across these studies and scarcity of reliable estimates on dietary consumption patterns across nations, a comprehensive assessment of environmental effects of achieving optimal diet globally has not been possible to date. GBD estimates the dietary consumption of key foods and nutrients across 195 nations annually. These data provide a unique opportunity to quantify the environmental burden of current dietary consumption patterns at global, regional, and national levels in a consistent and comparable way. Additionally, these data could potentially be used to evaluate the effect of various food system interventions on human health and environment.^42^

Our study also demonstrates the gaps in nationally representative individual-level data on intake of key foods and nutrients in different regions of the world, highlighting the importance of establishing national surveillance and monitoring systems for key dietary risk factors.^17^, ^18^ For example, although many countries collect data on fruit and vegetable intake, data on intake of specific nutrients such as sodium are scarce. The FAO/WHO Global Individual Food consumption data Tool^44^ aims to address this problem, but several important gaps will remain. In the absence of reliable biomarkers or more accurate methods of dietary assessment, the 24 h diet recall or diet record remains the gold standard method of dietary assessment. However, evidence from validation studies suggests that it is not highly reliable for assessment of foods and nutrients due to recall bias or potential social desirability.^45^, ^46^ This evidence highlights the need for development and validation of innovative dietary assessment methods. In the past decade, new methods have been developed; however, they have not been widely used and their validity has not been systematically evaluated.^47^ Furthermore, accurate estimation of nutrients (eg, fibre, calcium, and polyunsaturated fatty acids), remains a major challenge. Many countries do not have local food composition tables and rely on data from food composition tables from other countries (eg, US Department of Agriculture food composition tables). Additionally, the recipes of mixed dishes as well as formulation of the food products, particularly their content of fat, sugar, and sodium, varies across countries and over time, which makes estimation of the true intake of nutrient more challenging.

Our systematic evaluation of epidemiological evidence shows several important limitations in existing dietary relative risks. The effect sizes of the dietary risk factors on disease endpoints were mostly obtained from meta-analyses of prospective observational studies. Although many of these dietary relative risks have been adjusted for the major confounders (eg, age, sex, smoking, and physical activity), the possibility of residual confounding cannot be excluded. To remove the effect of energy intake as a potential confounder and address measurement error in dietary assessment tools, most cohorts have adjusted for total energy intake in their statistical models. This energy adjustment means that diet components are defined as risks in terms of the share of diet and not as absolute levels of exposure. In other words, an increase in intake of foods and macronutrients should be compensated by a decrease in intake of other dietary factors to hold total energy intake constant. Thus, the relative risk of change in each component of diet depends on the other components for which it is substituted. However, the relative risks estimated from meta-analyses of cohort studies do not generally specify the type of substitution. The definition of dietary factors (eg, whole grains) also varies across studies. Additionally, given the intake of healthy dietary factors are generally positively correlated with each other and inversely correlated with harmful dietary factors, the effect size of the individual dietary factors might be overestimated. Many of the observational studies used for estimation of the relative risks have not corrected risk estimates for dietary measurement error, and some have adjusted for factors along the causal pathways. Although many cohort studies have collected dietary data, only a few of them have published results of their assessment, which increases the possibility of publication bias. These limitations highlight the need for a collaborative effort to collect and harmonise all available dietary data from cohort studies and to do a pooled analysis for each diet–disease pair and quantify the effect size after adjusting for the same set of confounders.

Other potential limitations should also be considered in interpreting and using the findings of our study. We did not evaluate the effect of other forms of malnutrition (ie, undernutrition and obesity). The epidemiological evidence supporting a causal relationship between dietary risks and disease endpoints were mostly from observational studies, and the strength of evidence was generally weaker than the strength of evidence supporting a causal relationship between other established risks factors (eg, tobacco use and high systolic blood pressure) and chronic diseases. Additionally, the strength of evidence varied across foods and nutrients. Dietary data were from mixed sources and were not available for all countries. These factors increase the statistical uncertainty of our estimates for exposure to dietary risks. For sodium, we did not include data from spot urine sample, which resulted in a lower data representativeness index for sodium than that of other dietary risks. In estimation of the NCD burden of diet, we assumed that the distribution of dietary factors is independent within each unit of analysis (ie, country, age, and sex group), which might have resulted in underestimation or overestimation of the combined effect of dietary factors. To quantify the effect of correlation of dietary factors, we used individual-level data from the US National Health and Nutrition Examination Survey and estimated the overall burden of dietary risks (ie, joint population attributable fractions) with and without taking into account their correlation. The absolute difference in the joint population attributable fractions, on average, was less than 2%. Additionally, deaths due to some dietary risk factors might not be mutually exclusive, which could result in overestimation of the burden of disease attributable to diet.

In summary, we found that poor dietary habits are associated with a range of chronic diseases and can potentially be a major contributor to NCD mortality in all countries worldwide. This finding highlights the urgent need for coordinated global efforts to improve the quality of human diet. Given the complexity of dietary behaviours and the wide range of influences on diet, improving diet requires active collaboration of a variety of actors throughout the food system, along with policies targeting multiple sectors of the food system.

For more on the **Global Health Data Exchange** see http://ghdx.healthdata.org

For more on **Euromonitor** see https://www.euromonitor.com/

For more on **food balance sheets** see http://www.fao.org/economic/ess/fbs/en/

For the **Global Nutrient Database** see https://nutrition.healthdata.org/global-nutrient-database

For the list of all **dietary data sources** see http://ghdx.healthdata.org/gbd-2017/data-input-sources
