# Department of Error

**DOI:** 10.1016/S0140-6736(21)01342-8

**Published:** 2021-06-26

*GBD 2017 Diet Collaborators. Health effects of dietary risks in 195 countries, 1990–2017: a systematic analysis for the Global Burden of Disease Study 2017.* Lancet *2019;* **393:** *1958–72—*For this Article, Nayu Ikeda should have been included in the GBD 2017 Diet Collaborators. This correction has been made to the online version as of June 24, 2021.

