# Supplementary material for: Health effects of dietary risks in 195 countries, 1990–2017: a systematic analysis for the Global Burden of Disease Study 2017
Source: Lancet. 2019 May 11;393(10184):1958–72. doi: 10.1016/S0140-6736(19)30041-8 (PMC6899507; doi:10.1016/S0140-6736(19)30041-8)
Supplement: Supplementary appendix [file mmc1.pdf]

THE LANCET

Supplementary appendix

This appendix formed part of the original submission and has been peer reviewed. We post it as supplied by the authors.

Supplement to: GBD 2017 Diet Collaborators. Health effects of dietary risks in 195 countries, 1990–2017: a systematic analysis for the Global Burden of Disease Study 2017. *Lancet* 2019; published online April 3. http://dx.doi.org/10.1016/S0140-6736(19)30041-8.

Health Effects of Dietary Risks in 195 Countries: Findings from the Global Burden of Diseases Study 2017

Supplementary appendix

Table of contents

Overview	2
Selection of dietary risks	2
Estimating the dietary intake	2
Data Sources	2
Crosswalks	3
Age and sex splitting	4
Estimating the mean exposure level	4
Characterizing the distribution of intake	6
Relative risk	7
Theoretical minimum risk exposure level	8
Population attributable fraction	8
References	10
Supplemental Table 1. Epidemiological evidence supporting causality between dietary risk factors and disease endpoints.	11
Supplemental Table 2. Citation of the epidemiological studies used to evaluate the causal relationship between dietary risk-outcome pairs in Supplemental Table 1.	12
Supplemental Table 3. Relative risks used by age and sex and for each outcome for all diet risk factors.	14
Supplemental Table 4. Number, age-standardized rates, and proportion of deaths and DALYs attributable to dietary risks among adults at the global level in 1990 and 2017.	16
Supplemental Table 5. Age-standardized rates of mortality and DALYs attributable to dietary risks among adults at regional and SDI level in 1990 and 2017.	17
Supplemental Table 6. Age-standardized proportions of mortality and DALYs attributable to dietary risks among adults at regional and SDI level in 1990 and 2017.	22
Supplemental Table 7. Age-standardized rates of all-cause mortality and DALYs attributable to dietary risks among adults at the national level in 1990 and 2017.	26
Supplemental Table 8. Age-standardized proportions of all-cause mortality and DALYs attributable to dietary risks among adults at the national level in 1990 and 2017.	54
Supplemental Table 9. Number of DALYs and deaths attributable to individual dietary risks at the global level in 2017.	81
Supplemental Table 10. Age-standardized DALY rate and mortality rate (per 100, 000 population) attributable to individual dietary risks across SDI levels in 2017.	84
Supplemental Figure 1. Flowchart of the estimation process of dietary risk.	88
Supplemental Figure 2. Flowchart of the analytic process for Spatiotemporal Gaussian Process Regression.	89
Supplemental Figure 3. Ranking of dietary risk factors according to their attributable deaths at the global and regional level.	90
Supplemental Figure 4. Ranking of dietary risk factors according to their attributable DALYs at the global and regional level.	91
Supplemental Figure 5. Ranking of dietary risk factors according to their attributable deaths among the world's 20 most populous countries.	92
Supplemental Figure 6. Ranking of dietary risk factors according to their attributable DALYs among the world's 20 most populous countries.	93

Overview

The process of estimating the burden of disease attributable to each dietary risk factor has been shown in Supplemental Figure 1. Briefly, using the World Cancer Research Fund evidence grading criteria, we evaluated the strength of epidemiologic evidence supporting causal relationship between dietary factors and chronic diseases and selected the dietary factors for which we found sufficient evidence on their causal relationship with chronic disease endpoints. We then systematically collected dietary data from multiple sources and harmonized them. Then, we used spatio-temporal Gaussian process regression to estimate the mean exposure level for each dietary factor; used linear regression to estimate the standard deviation of intake based on the mean intake for each dietary factor; and used ensemble approach to characterize the distribution of intake for each dietary factor at the population level. We obtained the relative risk of each disease endpoint per serving of the dietary components from recent dose-response meta-analyses of prospective observational studies and estimated the optimal level of intake based on the level of intake associated with the lowest risk of mortality in prospective cohort studies. Using the exposure level, relative risk, and optimal level of intake, we estimated the population attributable fraction (PAF) for each diet-disease pair. Finally, using disease-specific PAFs and mortality and Disability-Adjusted Life Year (DALY), we calculated the total number of attributable deaths and DALYs across all relevant outcomes.

Selection of dietary risks

We used the World Cancer Research Fund evidence grading criteria to separately assess the strength of the epidemiologic evidence on the causal relationship between each dietary risk factor and disease endpoint, and only included dietary risk factors for which we found convincing or probable evidence on their relationship with chronic diseases. Supplemental Tables 1 and 2 summarize epidemiologic evidence supporting the causal relationship for the risk-outcome pairs included in our analysis.

Estimating the dietary intake

Data Sources

We systematically searched Medline to identify studies providing nationally or subnationally representative estimates of consumption of dietary factors. Additionally, we searched the Global Health Data Exchange (GHDx) database for individual-level data from nutrition surveys or household budget surveys providing dietary data. We included all nationally or subnationally representative studies providing data on mean intake of each dietary factor by age and sex. We only included studies reporting data collected between 1 January 1980 and 31 December 2016 in one of the 195 countries included in this analysis. Studies were excluded if using non-random samples (e.g., case-control studies or convenience samples); conducted among specific subpopulations (e.g., pregnant women, racial or ethnic minorities, immigrants, or individuals with specific diseases); having sample sizes of less than 20 per 5-year age-sex group; providing inadequate information on any of the inclusion criteria. We excluded non-English articles. For eight food groups (fruits, vegetables, legumes, nuts and seeds, red meat, processed meat, milk, SSBs), we used sales data from Euromonitor to capture recent trends in the intake. For six food groups (fruits, vegetables, legumes, nuts and seeds, milk, and red meat), we

used data from United Nations Food and Agriculture Organization (FAO) food balance sheets (FBS). Additionally, we estimated the national availability of nutrients (i.e., fiber, calcium, omega-3 fatty acids, polyunsaturated fatty acids availability, and saturated fatty acids) using data from FAO's Supply Utilization Accounts (SUA) and the United States Department of Agriculture's National Nutrition Database for Standard Reference. For whole grains, we used FBS and SUAs data to estimate the availability of refined grains and total grains at the country level and then calculated the availability of whole grains by difference. Types of data sources (other than 24-hour dietary recall) used in modeling of each dietary factor is summarized in Table A.

Table A. Types of data sources (other than 24-hour dietary recall) and covariates used in modeling of each dietary factor.

	Data Sources				Country level covariate
	Sales	FFQ[1]	HBS[2]	FAO	
**Diet low in fruits**	●	●	●	●	Lag-distributed income, total available kilocalories per person per day
**Diet low in vegetables**	●	●	●	●	Lag-distributed income, total available kilocalories per person per day
**Diet low in legumes**	●	-	●	●	Lag-distributed income, total available kilocalories per person per day
**Diet low in whole grains**	-	●	-	●	Lag-distributed income
**Diet low in nuts and seeds**	●	-	●	●	Lag-distributed income, total available kilocalories per person per day
**Diet low in milk**	●	●	●	●	Lag-distributed income, total available kilocalories per person per day
**Diet high in red meat**	●	●	●	●	Lag-distributed income, total available kilocalories per person per day
**Diet high in processed meat**	●	●	●	-	National availability of red meat (grams/person/day), National availability of pig meat (% of energy/person/day), Lag-distributed income
**Diet high in sugar-sweetened beverages**	●	●	●	-	National availability of sugar (grams/person/day), Lag-distributed income, total available kilocalories per person per day
**Diet low in fiber**	-	●	-	●	Lag-distributed income, total available kilocalories per person per day
**Diet suboptimal in calcium**	-	●	-	●	Lag-distributed income, total available kilocalories per person per day
**Diet low in seafood omega-3 fatty acids**	-	-	-	●	Landlocked nation (Yes/No), Lag-distributed income
**Diet low in polyunsaturated fatty acids**	-	●	-	●	Lag-distributed income, total available kilocalories per person per day
**Diet high in trans fatty acids**	●	●	-	-	-
**Diet high in sodium[3]**	-	-	-	-	-

[1] Food Frequency Questionnaire
[2] Household Budge Survey
[3] For sodium, we used data from the 24-hour urinary sodium and 24-hour dietary recall.

Crosswalks

We used dietary data from multiple sources and each type of dietary data was affected by specific types of biases. To adjust for biases of each dietary assessment method and to make our dietary data more comparable, we considered 24-hour diet recall as the gold standard method for assessing the mean intake at the population level and adjusted dietary data from other sources accordingly in a subset of countries with data from both methods of assessment. For sodium, 24-hour urinary sodium was

considered as the gold standard and we converted dietary sodium to urinary equivalent using a multiplier estimated from the surveys reporting both dietary and urinary sodium. Given that some of our data sources (i.e., availability, sales, and household data) were only providing data for all-age groups and both sexes, we first split these data into standard age-sex groups. Then, we matched dietary data from each non-gold-standard source data to the dietary data from the gold-standard source (i.e., 24-hour diet recall) by age, sex, country, and year and performed a linear regression analysis to estimate the consumption data from data on availability, sales, and household availability by age (a) and sex (s) using the following equation:

$$Dietary\ Intake_{a,s} = \beta * Availablity_{a,\,s} + age + sex$$

Age and sex splitting

Data from UN FAO, Euromonitor, and household budget surveys were for all-age groups and both sexes. To split these data into standard age-sex groups*, we* first characterized the global age and sex patterns of intake for reach dietary factor using data from 24-hour dietary recalls. Then, we applied the identified age patterns to split the availability, sales, and household data into standard age-sex groups.

Estimating the mean exposure level

We used a spatio-temporal Gaussian process regression (ST-GPR) to estimate the full time series of national intake of each diet component (flowchart). This approach is a stochastic modelling technique that is designed to detect signals amidst noisy data. It also serves as a powerful tool for interpolating non-linear trends.[1,2] Unlike classical linear models that assume that the trend underlying data follows a definitive functional form, GPR assumes that the specific trend of interest follows a Gaussian Process, which is defined by a mean function $m(\cdot)$ and a covariance function $Cov(\cdot)$. For example, let $p_{c,a,s,t}$ be the exposure, in normal, log, or logit space, observed in country c, for age group a, and sex s at time t:

$$(p_{c,a,s,t}) = g_{c,a,s}(t) + \epsilon_{c,a,s,t}$$

where

$$\epsilon_{c,a,s,t} \sim Normal(0, \sigma_p^2),$$

$$g_{c,a,s}(t) \sim GP\left(m_{c,a,s}(t), Cov\left(g_{c,a,s}(t)\right)\right).$$

The derivation of the mean and covariance functions, $m_{c,a,s}(t)$ and $Cov\left(g_{c,a,s}(t)\right)$, along with a more detailed description of the error variance (σ_p^2), is described below.

Estimating mean functions

We estimated mean functions using a two-step approach. To be more specific, $m_{c,a,s}(t)$ can be expressed, depending on the exposure transformation, as:

$$log\left(p_{c,a,s}(t)\right) = X_{c,a,s}\beta + h(r_{c,a,s,t})$$

$$logit\left(p_{c,a,s}(t)\right) = X_{c,a,s}\beta + h(r_{c,a,s,t})$$

$$p_{c,a,s}(t) = X_{c,a,s}\beta + h(r_{c,a,s,t})$$

where $X\beta$ is the summation of the components of a hierarchical mixed-effects linear regression, including the intercept and the product of covariates with their corresponding fixed effect coefficients. The second part of the equation, $h(r_{c,a,s,t})$, is a smoothing function for the residuals, $r_{c,a,s,t}$, derived from the linear model. Table A provides a description of covariates used in linear model for each dietary factor.

While the linear component captures the general trend in exposures over time, much of the data varaibility may still not be adequately accounted for. To address this, we fit a locally weighted polynomial regression (LOESS) function $h(r_{c,a,s,t})$ to systematically estimate this residual variability by borrowing strength across time, age, and space patterns (the spatiotemporal component of ST-GPR). The time adjustment parameter, defined by λ , aims to borrow strength from neighbouring time points (ie, the exposure in this year is highly correlated with exposure in the previous year but less so further back in time). The age-adjustment parameter, defined by ω, borrows strength from data in neighbouring age groups. The space-adjustment parameter, defined by ξ, aims to borrow strength across the hierarchy of geographical locations. We further combined the spatial and temporal weights into a single space-time weight, to allow the amount of spatial weight given to a particular point $r_{c,a,s,t}$ to fluctuate given the data availability at each time *t* and location-level *l* in the location hierarchy.

Estimating error variance

σ_p^2 represents the error variance in normal or transformed space including sampling variance of the estimates and prediction error from the crosswalks performed. First, variance was systematically imputed if the data extraction did not include any measure of uncertainty. When some sample sizes for data were available, missing sample sizes were imputed as the 5th percentile of available sample sizes. Missing variances were then predicted from the mean using a regression When sample sizes were entirely missing and could not be imputed, the 95th percentile of available variances at the most granular geographic level (ie, first country, then region, etc.) were used to impute missing variances. Finally, prior to GPR, an approximation of non-sampling variance was added to the error variance. Calculations of non-sampling variance were performed on normal-space variances. Non-sampling variance was calculated as the variance of inverse-variance weighted residuals from the space-time estimate at a given location level hierarchy. If there were fewer than ten data points at a given level of the location hierarchy, the non-sampling variance was replaced with that of the next highest geography level with more than ten data points.

Estimating the covariance function

The final input into GPR is the covariance function, which defines the shape and distribution of the trends. Here, we have chosen the Matern-Euclidian covariance function, which offers the flexibility to model a wide spectrum of trends with varying degrees of smoothness. The function is defined as follows:

$$M(t,t') = \sigma^2 \frac{2^{1-v}}{\Gamma(\mathrm{v})} \left(\frac{d(t,t')\sqrt{2v}}{l}\right)^v K_v\left(\frac{d(t,t')\sqrt{2v}}{l}\right)$$

where $d(\cdot)$is a distance function; σ^2, v, l, and K_v are hyperparameters of the covariance function – specifically σ^2 is the marginal variance, v is the smoothness parameter that defines the differentiability of the function, l is the length scale, which roughly defines the distance between which two points become uncorrelated, and K_v is the Bessel function. We approximated σ^2 by taking the normalised median absolute deviation $MADN(r_c')$ of the difference, which is the normalised absolute deviation of the difference of the first-stage linear regression estimate from the second-stage spatiotemporal smoothing step for each country. We then took the mean of these country-level MADN estimates for all countries with 10+ country-years of data, to ensure that differences between first- and second-stage estimates had sufficient data to truly convey meaningful information on model uncertainty. We used the parameter specifications $v = 2$ for all models.

Prediction using GPR

We integrated over $g_{c,t}(t_*)$ to predict a full time series for country c, age a, sex s, and the prediction time t_*:

$$p_{c,a,s}(t_*) \sim N\left(m_{c,a,s,t}(t_*), \sigma_p^2 I + Cov\left(g_{c,a,s,t}(t_*)\right)\right)$$

Random draws of 1,000 samples were obtained from the distributions above for every country for a given indicator. The final estimated mean for each country was the mean of the draws. In addition, 95% uncertainty intervals were calculated by taking the 2.5th and 97.5th percentile of the sample distribution. The linear modelling process was implemented using the lmer4 package in R, and the ST-GPR analysis was implemented through the PyMC2 package in Python.

Characterizing the distribution of intake

To characterize the population distribution of intake for each dietary factor by age, sex, country, and year, in addition to mean intake, we estimated the standard deviation of intake and the shape of the distribution for each dietary factor. To generate standard deviation for each dietary factor, we first modeled the relationship between standard deviation and mean among adults aged 25 years and older in nationally representative nutrition surveys using 24-hour diet recalls:

$$\ln(\text{Standard deviation}_i) = \beta_0 + \beta_1 \times \ln(Mean_i)$$

These coefficients were then applied to draws of mean intake from ST-GPR to generate draws of standard deviation for each dietary factor. Using 24-hour recalls with multiple measurements, we also quantified the within-person variation in consumption of each dietary component and adjusted the standard deviations accordingly.

To model the distribution of each dietary risk factor, we used an ensemble technique in which a model selection algorithm is used to choose the best model for each risk factor. We drew the initial set of candidate models from commonly used probability density functions (PDFs) families. These included: beta, exponential, gamma, gumbel, inverse gamma, inverse Weibull, log-logistic, lognormal, mirrored gamma, mirrored gumbel, normal, and Weibull. We fitted each PDFs candidate family to each dataset using the method of moments (MoM), and used the Kolmogorov-Smirnov test as the measure of goodness of fit (GoF). Preliminary analysis showed that the GoF ranking of PDF families varied across

datasets for any particular risk factor and that combining the predictions of differently fitted PDF families could dramatically improve the GoF for each dataset. Therefore, we developed a new model for prediction using the ensemble of candidate models, which is a weighted linear combination of all candidate models, { f }, where a set of weights {w} is chosen such that it is the sum of the weights equals to one and the values of the weights were determined by a second GoF criterion with its own validation process. Because of basic differences among risk factors, their distributions, and the risk attribution process, the model selection process was often slightly different for each risk factor. The details can be summarised by (1) the summary statistics for each dataset; (2) a table showing the Kolmogorov-Smirnov statistic for each candidate model and URD; (3) the criterion used for determining the overall GoF; (4) summary results of the validation process; and (5) the weights defining the final ensemble model for each dataset.

Relative risk

We obtained the relative risk of each disease endpoint per serving of the dietary components from recent dose-response meta-analyses of prospective observational studies. Considering the well-established age trend of the relative risks of metabolic risk factors for cardiovascular disease and diabetes,[5] we conducted a literature review to identify the most important metabolic mediators for each dietary factor and used the age trend of the relative risk of that mediator(s) and the disease endpoint to estimate the age-specific relative risk for each dietary factors (Supplemental Table 3). We took the following steps to estimate the age-specific relative risk for each dietary factor:

- For each dietary factor, we conducted a systematic review of literature and identified the metabolic risks that could potentially mediate the effect of diet on the disease endpoint (Table B).
- We obtained the age-specific relative risk for each mediator and disease endpoint from a meta-analysis of pooled cohort studies.[5]
- For each dietary risk and disease endpoint, we estimated the median age at event from the meta-analysis used to estimate the relative risks of the diet-disease pair.
- We assigned the relative risk reported in the meta-analysis to age group that included median age at event.
- We estimated the percent change in the relative risks between age at event and each group for all relevant metabolic mediators and took the average of them.
- We applied the average percent change in the relative risk of metabolic mediators to the relative risk of the dietary factor at age at event and estimated the age-specific relative risk of the dietary factor.

Table B. Metabolic mediators used to determine the age trend of the effect of dietary factors on cardiometabolic outcomes.

	Body Mass Index	Total Serum Cholesterol	Fasting Plasma Glucose	Systolic Blood Pressure
Diet low in fruits	●	●	●	●
Diet low in vegetables	●	●	●	●
Diet low in legumes	●	●	●	●
Diet low in whole grains	●	●	●	-
Diet low in nuts and seeds	●	●	●	●
Diet high in red meats	●	-	●	-
Diet high in processed meats	●	-	●	●

Diet low in fiber	-	●	-	-
Diet low in seafood omega-3 fatty acids	●	-	-	●
Diet low in polyunsaturated fatty acids	-	●	●	-
Diet high in trans fatty acids	●	●	-	-

Theoretical minimum risk exposure level

To estimate the TMREL for each dietary factor, we first calculated the level of intake associated with the lowest risk of mortality from each disease endpoint based on the studies included in the meta-analyses of the dietary relative risks.[6] Then, we calculated the TMREL as the weighted average of these numbers using the global number of deaths from each of outcome as the weight. To reflect the uncertainty of TMREL, we have assumed a uniform uncertainty distribution of 20% above and below the mean. For sodium, the evidence supporting the selection of the TMREL is uncertain. Therefore, we included in the uncertainty estimation sampling a uniform distribution of different TMRELs. The manuscript Table provides the range and distribution of the uncertainty in the TMREL.

Population attributable fraction

For each dietary risk factor, the attributable burden was estimated by comparing observed health outcomes to those that would have been observed if the past exposure level had been sustained at an optimal level, here referred to as theoretical minimum risk exposure level (TMREL). The main inputs to this analysis included the exposure level (where *l* is the lowest level of intake and *u* is highest level of intake) for each risk factor (p); the effect size of the risk factor on each disease endpoint (RR); the risk factor level associated with the lowest risk (TMREL); and the total number of deaths from each disease endpoint. Using the first three inputs, we estimated the population attributable fraction (PAF) for each risk-disease pair by age (a), sex (s), country (c), and year (t). Then, we used disease-specific (o) PAFs and mortality to calculate the total number of attributable deaths across all relevant outcomes (*w*):

$$PAF_{asct} = \frac{\int_l^u RR_{as}(x)P_{asct}(x)dx - RR_{as}(TMREL)}{\int_l^u RR_{as}(x)P_{asct}(x)dx}$$

$$Total\ attributable\ deaths_{asct} = \sum_{o=1}^{w} Death_{oasct}\, PAF_{oasct}$$

Assuming no correlation between dietary factors, we estimated the PAFs and number of deaths for the overall effect of all dietary factors relevant to each outcome based on the following equations:

$$PAF_{oasct} = 1 - \prod_{i}^{n}(1 - PAF_{ioasct})$$

$$Total\ attributable\ deaths_{asct} = \sum_{o=1}^{w} Death_{oasct}\, PAF_{oasct}$$

To account for potential overlaps between the effect of foods and nutrients that are associated with the same outcome (e.g., milk and calcium with colorectal cancer), we developed a mediation matrix and only accounted for the effect of either the food item or the nutrient.

Uncertainty of PAF estimates was calculated from 1,000 draws resulting from PAF calculations using the 1,000 draws of exposure estimates, 1,000 draws of relative risk estimates, and 1,000 draws of the theoretical minimum risk exposure level. The 1,000 draws of PAF estimates were multiplied by 1,000 draws of deaths and DALYs to produce 1,000 draws of attributable burden. All components of the PAF calculation analysis were assumed to be independent of each other.

References

1 Gaussian Process modeling of large scale terrain - IEEE Conference Publication. https://ieeexplore.ieee.org/document/5152677 (accessed Oct 7, 2018).

2 Press TM. Gaussian Processes for Machine Learning. The MIT Press. https://mitpress.mit.edu/books/gaussian-processes-machine-learning (accessed Oct 7, 2018).

3 Bowman KO, Shenton LR. Estimator: Method of Moments. In: Encyclopedia of statistical sciences. Wiley, 1998: 2092–8.

4 Massey FJ. The Kolmogorov-Smirnov Test for Goodness of Fit. *Journal of the American Statistical Association* 1951; **46**: 68–78.

5 Singh GM, Danaei G, Farzadfar F, *et al.* The Age-Specific Quantitative Effects of Metabolic Risk Factors on Cardiovascular Diseases and Diabetes: A Pooled Analysis. *PLOS ONE* 2013; **8**: e65174.

6 Gakidou E, Afshin A, Abajobir AA, *et al.* Global, regional, and national comparative risk assessment of 84 behavioural, environmental and occupational, and metabolic risks or clusters of risks, 1990–2016: a systematic analysis for the Global Burden of Disease Study 2016. *The Lancet* 2017; **390**: 1345–422.

Supplemental Table 1. Epidemiological evidence supporting causality between dietary risk factors and disease endpoints.

Risk	Outcome	RCTs (n)*	RCTs with significant effect in the opposite direction (%)	RCTs with null findings (%)	Prospective observational studies (n)*	Prospective observational studies with significant association in the	Case-control studies assessing the risk-outcome pair relationship (n)**	Case-control studies that show significant association in the opposite direction (%)	Lower limit of RR > 1.5†	Dose-response relationship	Biological plausibility	Analogy
**Dietary risks**												
Diet low in fruits	Lip and oral cavity cancer	0	-	-	2	0	15	0	🔴	🟢	🟢	🟢
Diet low in fruits	Nasopharynx cancer	0	-	-	2	0	15	0	🔴	🟢	🟢	🟢
Diet low in fruits	Other pharynx cancer	0	-	-	2	0	15	0	🔴	🟢	🟢	🟢
Diet low in fruits	Oesophageal cancer	0	-	-	5	0	-	-	🔴	🟢	🟢	🟢
Diet low in fruits	Larynx cancer	0	-	-	2	0	15	0	🔴	🟢	🟢	🟢
Diet low in fruits	Tracheal, bronchus, and lung cancer	0	-	-	22	0	-	-	🔴	🟢	🟢	🟢
Diet low in fruits	Ischaemic heart disease	0	-	-	9	0	-	-	🔴	🟢	🟢	🟢
Diet low in fruits	Ischaemic stroke	0	-	-	9	0	-	-	🔴	🟢	🟢	🟢
Diet low in fruits	Diabetes mellitus	0	-	-	9	0	-	-	🔴	🟢	🟢	🔴
Diet low in vegetables	Oesophageal cancer	0	-	-	5	0	-	-	🔴	🟢	🟢	🔴
Diet low in vegetables	Ischaemic heart disease	0	-	-	9	0	-	-	🔴	🟢	🟢	🟢
Diet low in vegetables	Ischaemic stroke	0	-	-	8	0	-	-	🔴	🟢	🟢	🟢
Diet low in vegetables	Haemorrhagic stroke	0	-	-	5	0	-	-	🔴	🟢	🟢	🟢
Diet low in legumes	Ischaemic heart disease	0	-	-	5	0	-	-	🔴	🟢	🟢	🔴
Diet low in whole grains	Ischaemic heart disease	0	-	-	7	0	-	-	🔴	🟢	🟢	🟢
Diet low in whole grains	Ischaemic stroke	0	-	-	6	0	-	-	🔴	🟢	🟢	🟢
Diet low in whole grains	Haemorrhagic stroke	0	-	-	6	0	-	-	🔴	🟢	🟢	🟢
Diet low in whole grains	Diabetes mellitus	0	-	-	10	0	-	-	🔴	🟢	🟢	🔴
Diet low in nuts and seeds	Ischaemic heart disease	1	0	100	6	0	-	-	🔴	🟢	🟢	🔴
Diet low in nuts and seeds	Diabetes mellitus	1	0	100	5	0	-	-	🔴	🟢	🟢	🔴
Diet low in milk	Colon and rectum cancer	0	-	-	7	0	-	-	🔴	🟢	🟢	🔴
Diet high in red meat	Colon and rectum cancer	0	-	-	8	0	-	-	🔴	🟢	🟢	🔴
Diet high in red meat	Diabetes mellitus	0	-	-	9	11	-	-	🔴	🟢	🟢	🔴
Diet high in processed meat	Colon and rectum cancer	0	-	-	9	11	-	-	🔴	🟢	🟢	🔴
Diet high in processed meat	Ischaemic heart disease	0	-	-	5	0	-	-	🔴	🟢	🟢	🔴
Diet high in processed meat	Diabetes mellitus	0	-	-	8	0	-	-	🔴	🟢	🟢	🔴
Diet high in sugar-sweetened beverages	Diabetes mellitus type 2	0	-	-	17	0	-	-	🔴	🟢	🟢	🔴
Diet high in sugar-sweetened beverages	Ischemic heart disease	0	-	-	4	0	-	-	🔴	🟢	🟢	🔴
Diet low in fibre	Colon and rectum cancer	0	-	-	15	0	-	-	🔴	🟢	🟢	🔴
Diet low in fibre	Ischaemic heart disease	0	-	-	12	0	-	-	🔴	🟢	🟢	🔴
Diet low in calcium	Colon and rectum cancer	0	-	-	13	0	-	-	🔴	🟢	🟢	🔴
Diet low in seafood omega-3 fatty acids	Ischaemic heart disease	17	0	94	16	0	-	-	🔴	🟢	🟢	🔴
Diet low in polyunsaturated fatty acids	Ischaemic heart disease	8	0	75	11	0	-	-	🔴	🟢	🟢	🔴
Diet high in trans fatty acids	Ischaemic heart disease	0	-	-	13	0	-	-	🔴	🟢	🟢	🔴
Diet high in sodium	Stomach cancer	0	-	-	10	0	-	-	🔴	🟢	🟢	🔴
Diet high in sodium‡	Systolic blood pressure	45	0	73	0	-	-	-	🔴	🟢	🟢	🔴

*The numbers in the table represent the independent RCTs and prospective observational studies evaluated the relationship between each risk-outcome pairs. If there were multiple reports from one study, they were counted as one study

†To evaluate the magnitude of the effect size for continuous risks, we evaluated the RR comparing the 75th percentile to the 25th percentile of the exposure distribution at the global level

‡The health effects of sodium on cardiovascular outcomes and chronic kidney disease were assessed through systolic blood pressure.Evidence on the direct effect of sodium on cardiovascular disease mainly comes from prospective cohort studies. Considering that, in GBD, we have only evaluated the effect of sodium mediated through systolic blood pressure, we did not present epidemiologic evidence on the direct effect of sodium on cardiovascular disease in this table. Evidence on the effect of sodium on systolic blood pressure mostly comes from randomized controlled trials. While some cohort studies evaluated the relationship between sodium and systolic blood pressure, we did not identify a systematic evaluation of these studies.

Supplemental Table 2. Citation of the epidemiological studies used to evaluate the causal relationship between dietary risk-outcome pairs in Supplemental Table 1.		
**Risk**	**Outcome**	**Citation/Note**
Diet low in fruits	Lip and oral cavity cancer	Key TJ. Fruit and vegetables and cancer risk. British Journal of Cancer 2011; 104: 6–11.
Diet low in fruits	Lip and oral cavity cancer	Jin, Jian, Zhiguo Ouyang, and Zhaoyan Wang. 2014. "Association of Fruit and Vegetables with the Risk of Nasopharyngeal Cancer: Evidence from a Meta-Analysis." Scientific Reports4 (July): srep05229. doi:10.1038/srep05229.
Diet low in fruits	Lip and oral cavity cancer	Pavia, Maria, Claudia Pileggi, Carmelo GA Nobile, and Italo F. Angelillo. 2006. "Association between Fruit and Vegetable Consumption and Oral Cancer: A Meta-Analysis of Observational Studies." The American Journal of Clinical Nutrition83 (5): 1126–34.
Diet low in fruits	Nasopharynx cancer	Key TJ. Fruit and vegetables and cancer risk. British Journal of Cancer 2011; 104: 6–11.
Diet low in fruits	Nasopharynx cancer	American Institute for Cancer Research, and World Cancer Research Fund, eds. 2007. Food, Nutrition, Physical Activity and the Prevention of Cancer: A Global Perspective: A Project of World Cancer Research Fund International. Washington, D.C: American Institute for Cancer Research.
Diet low in fruits	Nasopharynx cancer	Pavia, Maria, Claudia Pileggi, Carmelo GA Nobile, and Italo F. Angelillo. 2006. "Association between Fruit and Vegetable Consumption and Oral Cancer: A Meta-Analysis of Observational Studies." The American Journal of Clinical Nutrition83 (5): 1126–34.
Diet low in fruits	Other pharynx cancer	Key TJ. Fruit and vegetables and cancer risk. British Journal of Cancer 2011; 104: 6–11.
Diet low in fruits	Other pharynx cancer	American Institute for Cancer Research, and World Cancer Research Fund, eds. 2007. Food, Nutrition, Physical Activity and the Prevention of Cancer: A Global Perspective: A Project of World Cancer Research Fund International. Washington, D.C: American Institute for Cancer Research.
Diet low in fruits	Other pharynx cancer	Pavia, Maria, Claudia Pileggi, Carmelo GA Nobile, and Italo F. Angelillo. 2006. "Association between Fruit and Vegetable Consumption and Oral Cancer: A Meta-Analysis of Observational Studies." The American Journal of Clinical Nutrition83 (5): 1126–34.
Diet low in fruits	Larynx cancer	Key TJ. Fruit and vegetables and cancer risk. British Journal of Cancer 2011; 104: 6–11.
Diet low in fruits	Larynx cancer	American Institute for Cancer Research, and World Cancer Research Fund, eds. 2007. Food, Nutrition, Physical Activity and the Prevention of Cancer: A Global Perspective: A Project of World Cancer Research Fund International. Washington, D.C: American Institute for Cancer Research.
Diet low in fruits	Larynx cancer	Pavia, Maria, Claudia Pileggi, Carmelo GA Nobile, and Italo F. Angelillo. 2006. "Association between Fruit and Vegetable Consumption and Oral Cancer: A Meta-Analysis of Observational Studies." The American Journal of Clinical Nutrition83 (5): 1126–34.
Diet low in fruits	Oesophageal cancer	Liu J, Wang J, Leng Y, Lv C. Intake of fruit and vegetables and risk of esophageal squamous cell carcinoma: a meta-analysis of observational studies. Int J Cancer 2013; 133: 473–85.
Diet low in fruits	Tracheal, bronchus and lung cancer	Vieira AR, Abar L, Vingeliene S, et al. Fruits, vegetables and lung cancer risk: a systematic review and meta-analysis. Ann Oncol 2016; 27: 81–96.
Diet low in fruits	Ischaemic heart disease	Wang X, Ouyang Y, Liu J, et al. Fruit and vegetable consumption and mortality from all causes, cardiovascular disease, and cancer: systematic review and dose-response meta-analysis of prospective cohort studies. BMJ 2014; 349: g4490.
Diet low in fruits	Ischaemic stroke	Hu D, Huang J, Wang Y, Zhang D, Qu Y. Fruits and vegetables consumption and risk of stroke: a meta-analysis of prospective cohort studies. Stroke 2014; 45: 1613–9.
Diet low in fruits	Hemorrhagic stroke	Hu D, Huang J, Wang Y, Zhang D, Qu Y. Fruits and vegetables consumption and risk of stroke: a meta-analysis of prospective cohort studies. Stroke 2014; 45: 1613–9.
Diet low in fruits	Diabetes mellitus	Li M, Fan Y, Zhang X, Hou W, Tang Z. Fruit and vegetable intake and risk of type 2 diabetes mellitus: meta-analysis of prospective cohort studies. BMJ open 2014; 4(11): e005497.
Diet low in vegetables	Oesophageal cancer	Liu J, Wang J, Leng Y, Lv C. Intake of fruit and vegetables and risk of esophageal squamous cell carcinoma: a meta-analysis of observational studies. Int J Cancer 2013; 133: 473–85.
Diet low in vegetables	Ischaemic heart disease	Wang X, Ouyang Y, Liu J, et al. Fruit and vegetable consumption and mortality from all causes, cardiovascular disease, and cancer: systematic review and dose-response meta-analysis of prospective cohort studies. BMJ 2014; 349: g4490.
Diet low in vegetables	Ischaemic stroke	Hu D, Huang J, Wang Y, Zhang D, Qu Y. Fruits and vegetables consumption and risk of stroke: a meta-analysis of prospective cohort studies. Stroke 2014; 45: 1613–9.
Diet low in vegetables	Hemorrhagic stroke	Hu D, Huang J, Wang Y, Zhang D, Qu Y. Fruits and vegetables consumption and risk of stroke: a meta-analysis of prospective cohort studies. Stroke 2014; 45: 1613–9.
Diet low in legumes	Ischaemic heart disease	Afshin A, Micha R, Khatibzadeh S, Mozaffarian D. Consumption of nuts and legumes and risk of incident ischemic heart disease, stroke, and diabetes: a systematic review and meta-analysis. Am J Clin Nutr 2014; 100: 278–88.
Diet low in whole grains	Diabetes mellitus	Aune D, Norat T, Romundstad P, Vatten LJ. Whole grain and refined grain consumption and the risk of type 2 diabetes: a systematic review and dose-response meta-analysis of cohort studies. Eur J Epidemiol 2013; 28: 845–58.
Diet low in whole grains	Ischaemic heart disease	Aune D, Keum N, Giovannucci E, et al. Whole grain consumption and risk of cardiovascular disease, cancer, and all cause and cause specific mortality: systematic review and dose-response meta-analysis of prospective studies. BMJ 2016; 353: i2716.
Diet low in nuts and seeds	Ischaemic heart disease	Afshin A, Micha R, Khatibzadeh S, Mozaffarian D. Consumption of nuts and legumes and risk of incident ischemic heart disease, stroke, and diabetes: a systematic review and meta-analysis. Am J Clin Nutr 2014; 100: 278–88.
Diet low in nuts and seeds	Diabetes mellitus	Afshin A, Micha R, Khatibzadeh S, Mozaffarian D. Consumption of nuts and legumes and risk of incident ischemic heart disease, stroke, and diabetes: a systematic review and meta-analysis. Am J Clin Nutr 2014; 100: 278–88.
Diet low in milk	Colon and rectum cancer	World Cancer Research Fund, American Institute for Cancer Research, Imperial College London. WCRF/AICR Systematic Literature Review Continuous Update Project Report: The Associations between Food, Nutrition and Physical Activity and the Risk of Colorectal Cancer. Oct 2010.
Diet high in red meat	Colon and rectum cancer	World Cancer Research Fund, American Institute for Cancer Research, Imperial College London. WCRF/AICR Systematic Literature Review Continuous Update Project Report: The Associations between Food, Nutrition and Physical Activity and the Risk of Colorectal Cancer. Oct 2010.
Diet high in red meat	Diabetes mellitus	Pan A, Sun Q, Bernstein AM, et al. Red meat consumption and risk of type 2 diabetes: 3 cohorts of US adults and an updated meta-analysis. Am J Clin Nutr 2011; 94: 1088–96.

Supplemental Table 2. Citation of the epidemiological studies used to evaluate the causal relationship between dietary risk-outcome pairs in Supplemental Table 1.		
**Risk**	**Outcome**	**Citation/Note**
Diet high in processed meat	Colon and rectum cancer	World Cancer Research Fund, American Institute for Cancer Research, Imperial College London. WCRF/AICR Systematic Literature Review Continuous Update Project Report: The Associations between Food, Nutrition and Physical Activity and the Risk of Colorectal Cancer. Oct 2010.
Diet high in processed meat	Ischaemic heart disease	Micha R, Wallace SK, Mozaffarian D. Red and processed meat consumption and risk of incident coronary heart disease, stroke, and diabetes mellitus: a systematic review and meta-analysis. Circulation 2010; 121: 2271–83.
Diet high in processed meat	Diabetes mellitus	Pan A, Sun Q, Bernstein AM, et al. Red meat consumption and risk of type 2 diabetes: 3 cohorts of US adults and an updated meta-analysis. Am J Clin Nutr 2011; 94: 1088–96.
Diet high in sugar-sweetened beverages	Diabetes mellitus	Imamura F, O'Connor L, Ye Z, *et al.* Consumption of sugar sweetened beverages, artificially sweetened beverages, and fruit juice and incidence of type 2 diabetes: systematic review, meta-analysis, and estimation of population attributable fraction. *BMJ* 2015; 351: h3576.
Diet high in sugar-sweetened beverages	Ischaemic heart disease	Xi B, Huang Y, Reilly KH, et al. Sugar-sweetened beverages and risk of hypertension and CVD: a dose-response meta-analysis. Br J Nutr 2015; 113: 709–17.
Diet low fibre	Colon and rectum cancer	World Cancer Research Fund, American Institute for Cancer Research, Imperial College London. WCRF/AICR Systematic Literature Review Continuous Update Project Report: The Associations between Food, Nutrition and Physical Activity and the Risk of Colorectal Cancer. Oct 2010.
Diet low fibre	Ischaemic heart disease	Threapleton DE, Greenwood DC, Evans CE, et al. Dietary fibre intake and risk of cardiovascular disease: systematic review and meta-analysis. BMJ (Clinical research ed) 2013; 347: f6879.
Diet low in calcium	Colon and rectum cancer	World Cancer Research Fund, American Institute for Cancer Research, Imperial College London. WCRF/AICR Systematic Literature Review Continuous Update Project Report: The Associations between Food, Nutrition and Physical Activity and the Risk of Colorectal Cancer. Oct 2010.
Diet low in seafood omega-3 fats	Ischaemic heart disease	Chowdhury R, Stevens S, Gorman D, et al. Association between fish consumption, long chain omega 3 fatty acids, and risk of cerebrovascular disease: systematic review and meta-analysis. BMJ (Clinical research ed) 2012; 345: e6698.
Diet low in polyunsaturated fats	Ischaemic heart disease	Farvid MS, Ding M, Pan A, et al. Dietary linoleic acid and risk of coronary heart disease: a systematic review and meta-analysis of prospective cohort studies. Circulation 2014; 130: 1568–78.
Diet low in polyunsaturated fats	Ischaemic heart disease	Mozaffarian D, Micha R, Wallace S. Effects on coronary heart disease of increasing polyunsaturated fat in place of saturated fat: a systematic review and meta-analysis of randomized controlled trials. PLoS Med 2010; 7: e1000252.
Diet high in trans fats	Ischaemic heart disease	Mozaffarian D, Clarke R. Quantitative effects on cardiovascular risk factors and coronary heart disease risk of replacing partially hydrogenated vegetable oils with other fats and oils. Eur J Clin Nutr. 2009; 63(Suppl 2): S22-33.
Diet high in trans fats	Ischaemic heart disease	http://www.bmj.com/content/bmj/suppl/2015/08/11/bmj.h3978.DC1/sour025275.ww2_default.pdf; pg. 44
Diet high in sodium and high systolic blood pressure	n/a	Aburto NJ, Ziolkovska A, Hooper L, Elliott P, Cappuccio FP, Meerpohl JJ. Effect of lower sodium intake on health: systematic review and meta-analyses. BMJ 2013; 346: f1326.
Diet high in sodium	Stomach cancer	World Cancer Research Fund, American Institute for Cancer Research. Food, Nutrition, Physical Activity, and the Prevention of Cancer: a Global Perspective. Washington DC: AICR, 2007.
Diet high in sodium	Stomach cancer	D'Elia, Lanfranco, Giovanni Rossi, Renato Ippolito, Francesco P. Cappuccio, and Pasquale Strazzullo. 2012. "Habitual Salt Intake and Risk of Gastric Cancer: A Meta-Analysis of Prospective Studies." Clinical Nutrition31 (4): 489–98. doi:10.1016/j.clnu.2012.01.003.
Diet low in nuts and seeds	Ischaemic heart disease and diabetes mellitus	Experimental evidence on the relationship of nuts with ischaemic heart disease and diabetes mellitus come from the PREDIMED trial; a randomized trial consisting of three arms: a Mediterranean diet with extra-virgin olive oil, a Mediterranean diet with nuts, and a control diet. Given that the intake of dietary factors other than nuts changed in the intervention arms of this trial, the observed effect might be fully attributable to nuts.
Diet high in sodium	Cardiovascular diseases	Evidence on the direct effect of sodium on cardiovascular disease mainly comes from prospective cohort studies. Considering that, in GBD, we have only evaluated the effect of sodium mediated through systolic blood pressure, we did not present epidemiologic evidence on the direct effect of sodium on cardiovascular disease in this table. Evidence on the effect of sodium on systolic blood pressure mostly comes from randomized controlled trials. While some cohort studies evaluated the relationship between sodium and systolic blood pressure, we did not identify a systematic evaluation of these studies.

Supplemental Table 3. Relative risks used by age and sex and for each outcome for all diet risk factors.

Risk - Outcome	Category / Units	Morbidity / Mortality	Sex	25-29 years	30-34 years	35-39 years	40-44 years	45-49 years	50-54 years	55-59 years	60-64 years	65-69 years	70-74 years	75-79 years	80-84 years	85-89 years	90-94 years	95+ years
**Diet low in fruits**																		
Lip and oral cavity cancer	100 g/day	Both	Both	1.042 (0.994 to 1.091)	1.042 (0.994 to 1.091)	1.042 (0.994 to 1.091)	1.042 (0.994 to 1.091)	1.042 (0.994 to 1.091)	1.042 (0.994 to 1.091)	1.042 (0.994 to 1.091)	1.042 (0.994 to 1.091)	1.042 (0.994 to 1.091)	1.042 (0.994 to 1.091)	1.042 (0.994 to 1.091)	1.042 (0.994 to 1.091)	1.042 (0.994 to 1.091)	1.042 (0.994 to 1.091)	1.042 (0.994 to 1.091)
Nasopharynx cancer	100 g/day	Both	Both	1.043 (0.992 to 1.092)	1.043 (0.992 to 1.092)	1.043 (0.992 to 1.092)	1.043 (0.992 to 1.092)	1.043 (0.992 to 1.092)	1.043 (0.992 to 1.092)	1.043 (0.992 to 1.092)	1.043 (0.992 to 1.092)	1.043 (0.992 to 1.092)	1.043 (0.992 to 1.092)	1.043 (0.992 to 1.092)	1.043 (0.992 to 1.092)	1.043 (0.992 to 1.092)	1.043 (0.992 to 1.092)	1.043 (0.992 to 1.092)
Other pharynx cancer	100 g/day	Both	Both	1.042 (0.996 to 1.095)	1.042 (0.996 to 1.095)	1.042 (0.996 to 1.095)	1.042 (0.996 to 1.095)	1.042 (0.996 to 1.095)	1.042 (0.996 to 1.095)	1.042 (0.996 to 1.095)	1.042 (0.996 to 1.095)	1.042 (0.996 to 1.095)	1.042 (0.996 to 1.095)	1.042 (0.996 to 1.095)	1.042 (0.996 to 1.095)	1.042 (0.996 to 1.095)	1.042 (0.996 to 1.095)	1.042 (0.996 to 1.095)
Oesophageal cancer	100 g/day	Both	Both	1.153 (1.033 to 1.288)	1.153 (1.033 to 1.288)	1.153 (1.033 to 1.288)	1.153 (1.033 to 1.288)	1.153 (1.033 to 1.288)	1.153 (1.033 to 1.288)	1.153 (1.033 to 1.288)	1.153 (1.033 to 1.288)	1.153 (1.033 to 1.288)	1.153 (1.033 to 1.288)	1.153 (1.033 to 1.288)	1.153 (1.033 to 1.288)	1.153 (1.033 to 1.288)	1.153 (1.033 to 1.288)	1.153 (1.033 to 1.288)
Larynx cancer	100 g/day	Both	Both	1.042 (0.995 to 1.095)	1.042 (0.995 to 1.095)	1.042 (0.995 to 1.095)	1.042 (0.995 to 1.095)	1.042 (0.995 to 1.095)	1.042 (0.995 to 1.095)	1.042 (0.995 to 1.095)	1.042 (0.995 to 1.095)	1.042 (0.995 to 1.095)	1.042 (0.995 to 1.095)	1.042 (0.995 to 1.095)	1.042 (0.995 to 1.095)	1.042 (0.995 to 1.095)	1.042 (0.995 to 1.095)	1.042 (0.995 to 1.095)
Tracheal, bronchus, and lung cancer	100 g/day	Both	Both	1.076 (1.031 to 1.123)	1.076 (1.031 to 1.123)	1.076 (1.031 to 1.123)	1.076 (1.031 to 1.123)	1.076 (1.031 to 1.123)	1.076 (1.031 to 1.123)	1.076 (1.031 to 1.123)	1.076 (1.031 to 1.123)	1.076 (1.031 to 1.123)	1.076 (1.031 to 1.123)	1.076 (1.031 to 1.123)	1.076 (1.031 to 1.123)	1.076 (1.031 to 1.123)	1.076 (1.031 to 1.123)	1.076 (1.031 to 1.123)
Ischaemic heart disease	100 g/day	Both	Both	1.254 (1.083 to 1.442)	1.209 (1.07 to 1.361)	1.159 (1.054 to 1.271)	1.131 (1.045 to 1.221)	1.125 (1.043 to 1.211)	1.114 (1.039 to 1.193)	1.099 (1.034 to 1.167)	1.087 (1.03 to 1.146)	1.078 (1.027 to 1.13)	1.07 (1.025 to 1.117)	1.064 (1.022 to 1.106)	1.057 (1.02 to 1.095)	1.057 (1.02 to 1.095)	1.057 (1.02 to 1.095)	1.057 (1.02 to 1.095)
Ischaemic stroke	100 g/day	Both	Both	2.024 (1.465 to 2.818)	1.834 (1.39 to 2.444)	1.621 (1.301 to 2.043)	1.48 (1.239 to 1.787)	1.403 (1.204 to 1.653)	1.333 (1.171 to 1.533)	1.272 (1.142 to 1.432)	1.222 (1.116 to 1.348)	1.181 (1.096 to 1.283)	1.145 (1.078 to 1.225)	1.114 (1.061 to 1.175)	1.054 (1.029 to 1.082)	1.054 (1.029 to 1.082)	1.054 (1.029 to 1.082)	1.054 (1.029 to 1.082)
Intracerebral hemorrhage	100 g/day	Both	Both	1.688 (1.319 to 2.182)	1.576 (1.273 to 1.972)	1.444 (1.215 to 1.732)	1.365 (1.18 to 1.595)	1.336 (1.167 to 1.544)	1.3 (1.15 to 1.483)	1.26 (1.131 to 1.415)	1.226 (1.115 to 1.358)	1.193 (1.099 to 1.305)	1.164 (1.084 to 1.256)	1.133 (1.069 to 1.207)	1.065 (1.034 to 1.1)	1.065 (1.034 to 1.1)	1.065 (1.034 to 1.1)	1.065 (1.034 to 1.1)
Subarachnoid hemorrhage	100 g/day	Both	Both	1.688 (1.319 to 2.182)	1.576 (1.273 to 1.972)	1.444 (1.215 to 1.732)	1.365 (1.18 to 1.595)	1.336 (1.167 to 1.544)	1.3 (1.15 to 1.483)	1.26 (1.131 to 1.415)	1.226 (1.115 to 1.358)	1.193 (1.099 to 1.305)	1.164 (1.084 to 1.256)	1.133 (1.069 to 1.207)	1.065 (1.034 to 1.1)	1.065 (1.034 to 1.1)	1.065 (1.034 to 1.1)	1.065 (1.034 to 1.1)
Diabetes mellitus type 2	100 g/day	Both	Both	1.125 (1.027 to 1.238)	1.122 (1.026 to 1.232)	1.119 (1.026 to 1.226)	1.113 (1.024 to 1.214)	1.102 (1.022 to 1.194)	1.093 (1.02 to 1.176)	1.085 (1.019 to 1.16)	1.076 (1.017 to 1.143)	1.068 (1.015 to 1.128)	1.061 (1.014 to 1.114)	1.052 (1.012 to 1.098)	1.036 (1.008 to 1.066)	1.036 (1.008 to 1.066)	1.036 (1.008 to 1.066)	1.036 (1.008 to 1.066)
**Diet low in vegetables**																		
Ischaemic heart disease	100 g/day	Both	Both	1.249 (1.089 to 1.446)	1.205 (1.074 to 1.362)	1.154 (1.057 to 1.269)	1.126 (1.047 to 1.219)	1.121 (1.045 to 1.21)	1.111 (1.042 to 1.193)	1.098 (1.037 to 1.168)	1.086 (1.032 to 1.148)	1.077 (1.029 to 1.133)	1.07 (1.027 to 1.12)	1.064 (1.024 to 1.109)	1.057 (1.022 to 1.097)	1.057 (1.022 to 1.097)	1.057 (1.022 to 1.097)	1.057 (1.022 to 1.097)
Ischaemic stroke	100 g/day	Both	Both	1.249 (1.049 to 1.463)	1.211 (1.042 to 1.388)	1.165 (1.033 to 1.3)	1.132 (1.027 to 1.238)	1.113 (1.023 to 1.203)	1.095 (1.02 to 1.17)	1.079 (1.017 to 1.141)	1.065 (1.014 to 1.116)	1.054 (1.012 to 1.096)	1.044 (1.009 to 1.077)	1.035 (1.007 to 1.061)	1.017 (1.004 to 1.029)	1.017 (1.004 to 1.029)	1.017 (1.004 to 1.029)	1.017 (1.004 to 1.029)
Intracerebral hemorrhage	100 g/day	Both	Both	1.177 (1.046 to 1.326)	1.153 (1.04 to 1.278)	1.122 (1.032 to 1.22)	1.102 (1.027 to 1.184)	1.095 (1.025 to 1.17)	1.086 (1.023 to 1.153)	1.075 (1.02 to 1.134)	1.066 (1.018 to 1.117)	1.057 (1.015 to 1.101)	1.049 (1.013 to 1.086)	1.04 (1.011 to 1.071)	1.02 (1.005 to 1.035)	1.02 (1.005 to 1.035)	1.02 (1.005 to 1.035)	1.02 (1.005 to 1.035)
Subarachnoid hemorrhage	100 g/day	Both	Both	1.177 (1.046 to 1.326)	1.153 (1.04 to 1.278)	1.122 (1.032 to 1.22)	1.102 (1.027 to 1.184)	1.095 (1.025 to 1.17)	1.086 (1.023 to 1.153)	1.075 (1.02 to 1.134)	1.066 (1.018 to 1.117)	1.057 (1.015 to 1.101)	1.049 (1.013 to 1.086)	1.04 (1.011 to 1.071)	1.02 (1.005 to 1.035)	1.02 (1.005 to 1.035)	1.02 (1.005 to 1.035)	1.02 (1.005 to 1.035)
**Diet low in legumes**																		
Ischaemic heart disease	50 g/day	Both	Both	1.499 (1.18 to 1.89)	1.453 (1.166 to 1.801)	1.388 (1.144 to 1.677)	1.332 (1.125 to 1.573)	1.287 (1.11 to 1.49)	1.237 (1.092 to 1.401)	1.181 (1.071 to 1.303)	1.139 (1.055 to 1.23)	1.111 (1.045 to 1.183)	1.089 (1.036 to 1.146)	1.074 (1.03 to 1.12)	1.101 (1.041 to 1.165)	1.101 (1.041 to 1.165)	1.101 (1.041 to 1.165)	1.101 (1.041 to 1.165)
**Diet low in whole grains**																		
Ischaemic heart disease	50 g/day	Both	Both	1.478 (1.274 to 1.722)	1.387 (1.225 to 1.578)	1.285 (1.168 to 1.418)	1.228 (1.136 to 1.333)	1.216 (1.129 to 1.313)	1.194 (1.117 to 1.281)	1.165 (1.1 to 1.238)	1.141 (1.086 to 1.203)	1.125 (1.076 to 1.179)	1.112 (1.068 to 1.16)	1.102 (1.062 to 1.145)	1.097 (1.059 to 1.138)	1.097 (1.059 to 1.138)	1.097 (1.059 to 1.138)	1.097 (1.059 to 1.138)
Ischaemic stroke	50 g/day	Both	Both	2.075 (1.669 to 2.517)	1.863 (1.548 to 2.199)	1.624 (1.406 to 1.849)	1.466 (1.309 to 1.625)	1.38 (1.255 to 1.505)	1.304 (1.206 to 1.401)	1.241 (1.165 to 1.316)	1.189 (1.13 to 1.247)	1.15 (1.104 to 1.195)	1.117 (1.081 to 1.151)	1.09 (1.063 to 1.116)	1.041 (1.029 to 1.053)	1.041 (1.029 to 1.053)	1.041 (1.029 to 1.053)	1.041 (1.029 to 1.053)
Intracerebral hemorrhage	50 g/day	Both	Both	1.596 (1.406 to 1.825)	1.484 (1.333 to 1.662)	1.349 (1.244 to 1.471)	1.276 (1.194 to 1.369)	1.258 (1.182 to 1.344)	1.232 (1.165 to 1.309)	1.201 (1.143 to 1.267)	1.176 (1.126 to 1.233)	1.15 (1.108 to 1.198)	1.128 (1.092 to 1.169)	1.106 (1.076 to 1.139)	1.05 (1.036 to 1.065)	1.05 (1.036 to 1.065)	1.05 (1.036 to 1.065)	1.05 (1.036 to 1.065)
Subarachnoid hemorrhage	50 g/day	Both	Both	1.596 (1.406 to 1.825)	1.484 (1.333 to 1.662)	1.349 (1.244 to 1.471)	1.276 (1.194 to 1.369)	1.258 (1.182 to 1.344)	1.232 (1.165 to 1.309)	1.201 (1.143 to 1.267)	1.176 (1.126 to 1.233)	1.15 (1.108 to 1.198)	1.128 (1.092 to 1.169)	1.106 (1.076 to 1.139)	1.05 (1.036 to 1.065)	1.05 (1.036 to 1.065)	1.05 (1.036 to 1.065)	1.05 (1.036 to 1.065)
Diabetes mellitus type 2	50 g/day	Both	Both	1.231 (1.125 to 1.349)	1.226 (1.122 to 1.341)	1.22 (1.119 to 1.331)	1.208 (1.113 to 1.313)	1.189 (1.103 to 1.283)	1.172 (1.094 to 1.256)	1.156 (1.085 to 1.232)	1.139 (1.077 to 1.207)	1.125 (1.069 to 1.184)	1.111 (1.061 to 1.163)	1.095 (1.053 to 1.14)	1.064 (1.036 to 1.094)	1.064 (1.036 to 1.094)	1.064 (1.036 to 1.094)	1.064 (1.036 to 1.094)
**Diet low in nuts and seeds**																		
Ischaemic heart disease	4.05 g/day	Morbidity	Both	1.176 (1.055 to 1.322)	1.143 (1.045 to 1.259)	1.105 (1.033 to 1.188)	1.084 (1.027 to 1.15)	1.081 (1.026 to 1.144)	1.074 (1.024 to 1.132)	1.064 (1.021 to 1.114)	1.056 (1.018 to 1.099)	1.05 (1.016 to 1.089)	1.046 (1.015 to 1.081)	1.042 (1.014 to 1.075)	1.039 (1.013 to 1.069)	1.039 (1.013 to 1.069)	1.039 (1.013 to 1.069)	1.039 (1.013 to 1.069)
Ischaemic heart disease	4.05 g/day	Mortality	Both	1.209 (1.128 to 1.296)	1.169 (1.105 to 1.239)	1.124 (1.077 to 1.174)	1.099 (1.062 to 1.138)	1.095 (1.06 to 1.133)	1.088 (1.055 to 1.122)	1.076 (1.048 to 1.105)	1.066 (1.042 to 1.092)	1.059 (1.037 to 1.082)	1.054 (1.034 to 1.075)	1.05 (1.032 to 1.069)	1.046 (1.029 to 1.064)	1.046 (1.029 to 1.064)	1.046 (1.029 to 1.064)	1.046 (1.029 to 1.064)
Diabetes mellitus type 2	4.05 g/day	Both	Both	1.05 (1.025 to 1.075)	1.049 (1.025 to 1.073)	1.048 (1.024 to 1.071)	1.045 (1.023 to 1.068)	1.041 (1.021 to 1.062)	1.038 (1.019 to 1.056)	1.035 (1.018 to 1.052)	1.031 (1.016 to 1.046)	1.028 (1.014 to 1.042)	1.025 (1.013 to 1.037)	1.022 (1.011 to 1.032)	1.015 (1.007 to 1.022)	1.015 (1.007 to 1.022)	1.015 (1.007 to 1.022)	1.015 (1.007 to 1.022)
**Diet low in milk**																		
Colon and rectum cancer	226.8 g/day	Both	Both	1.113 (1.039 to 1.202)	1.113 (1.039 to 1.202)	1.113 (1.039 to 1.202)	1.113 (1.039 to 1.202)	1.113 (1.039 to 1.202)	1.113 (1.039 to 1.202)	1.113 (1.039 to 1.202)	1.113 (1.039 to 1.202)	1.113 (1.039 to 1.202)	1.113 (1.039 to 1.202)	1.113 (1.039 to 1.202)	1.113 (1.039 to 1.202)	1.113 (1.039 to 1.202)	1.113 (1.039 to 1.202)	1.113 (1.039 to 1.202)
**Diet high in red meat**																		
Colon and rectum cancer	100 g/day	Both	Both	1.167 (1.033 to 1.309)	1.167 (1.033 to 1.309)	1.167 (1.033 to 1.309)	1.167 (1.033 to 1.309)	1.167 (1.033 to 1.309)	1.167 (1.033 to 1.309)	1.167 (1.033 to 1.309)	1.167 (1.033 to 1.309)	1.167 (1.033 to 1.309)	1.167 (1.033 to 1.309)	1.167 (1.033 to 1.309)	1.167 (1.033 to 1.309)	1.167 (1.033 to 1.309)	1.167 (1.033 to 1.309)	1.167 (1.033 to 1.309)
Diabetes mellitus type 2	100 g/day	Both	Both	1.322 (1.037 to 1.603)	1.314 (1.036 to 1.588)	1.305 (1.035 to 1.569)	1.288 (1.034 to 1.536)	1.26 (1.031 to 1.48)	1.236 (1.028 to 1.433)	1.213 (1.026 to 1.389)	1.19 (1.023 to 1.345)	1.169 (1.021 to 1.306)	1.15 (1.019 to 1.269)	1.128 (1.016 to 1.229)	1.086 (1.011 to 1.152)	1.086 (1.011 to 1.152)	1.086 (1.011 to 1.152)	1.086 (1.011 to 1.152)
**Diet high in processed meat**																		
Colon and rectum cancer	50 g/day	Both	Both	1.179 (1.093 to 1.267)	1.179 (1.093 to 1.267)	1.179 (1.093 to 1.267)	1.179 (1.093 to 1.267)	1.179 (1.093 to 1.267)	1.179 (1.093 to 1.267)	1.179 (1.093 to 1.267)	1.179 (1.093 to 1.267)	1.179 (1.093 to 1.267)	1.179 (1.093 to 1.267)	1.179 (1.093 to 1.267)	1.179 (1.093 to 1.267)	1.179 (1.093 to 1.267)	1.179 (1.093 to 1.267)	1.179 (1.093 to 1.267)
Ischaemic heart disease	50 g/day	Both	Both	2.568 (1.047 to 4.657)	2.124 (1.038 to 3.478)	1.72 (1.028 to 2.489)	1.545 (1.022 to 2.093)	1.547 (1.022 to 2.097)	1.52 (1.022 to 2.037)	1.467 (1.02 to 1.922)	1.422 (1.018 to 1.826)	1.386 (1.017 to 1.75)	1.354 (1.016 to 1.683)	1.325 (1.015 to 1.622)	1.252 (1.012 to 1.475)	1.252 (1.012 to 1.475)	1.252 (1.012 to 1.475)	1.252 (1.012 to 1.475)
Diabetes mellitus type 2	50 g/day	Both	Both	1.94 (1.395 to 2.545)	1.913 (1.386 to 2.496)	1.881 (1.375 to 2.439)	1.824 (1.354 to 2.337)	1.731 (1.319 to 2.173)	1.653 (1.289 to 2.038)	1.583 (1.261 to 1.918)	1.512 (1.233 to 1.798)	1.45 (1.207 to 1.696)	1.393 (1.183 to 1.603)	1.332 (1.157 to 1.505)	1.216 (1.105 to 1.323)	1.216 (1.105 to 1.323)	1.216 (1.105 to 1.323)	1.216 (1.105 to 1.323)

Supplemental Table 3. Relative risks used by age and sex and for each outcome for all diet risk factors.

Risk - Outcome	Category / Units	Morbidity / Mortality	Sex	25-29 years	30-34 years	35-39 years	40-44 years	45-49 years	50-54 years	55-59 years	60-64 years	65-69 years	70-74 years	75-79 years	80-84 years	85-89 years	90-94 years	95+ years
**Diet high in sugar-sweetened beverages**																		
Ischaemic heart disease		Both	Both	1.377 (0.933 to 1.883)	1.311 (0.943 to 1.717)	1.232 (0.955 to 1.521)	1.195 (0.961 to 1.436)	1.186 (0.963 to 1.413)	1.172 (0.965 to 1.381)	1.156 (0.968 to 1.343)	1.14 (0.971 to 1.306)	1.124 (0.974 to 1.27)	1.11 (0.977 to 1.238)	1.095 (0.98 to 1.205)	1.067 (0.985 to 1.143)	1.067 (0.985 to 1.143)	1.067 (0.985 to 1.143)	1.067 (0.985 to 1.143)
Diabetes mellitus type 2		Both	Both	1.263 (1.129 to 1.4)	1.257 (1.126 to 1.39)	1.25 (1.123 to 1.379)	1.237 (1.117 to 1.358)	1.214 (1.106 to 1.322)	1.195 (1.097 to 1.292)	1.177 (1.088 to 1.264)	1.158 (1.079 to 1.235)	1.141 (1.071 to 1.209)	1.125 (1.063 to 1.185)	1.107 (1.055 to 1.158)	1.08 (1.041 to 1.118)	1.08 (1.041 to 1.118)	1.08 (1.041 to 1.118)	1.08 (1.041 to 1.118)
**Diet low in fibre**																		
Colon and rectum cancer	20 g/day	Both	Both	1.236 (1.133 to 1.35)	1.236 (1.133 to 1.35)	1.236 (1.133 to 1.35)	1.236 (1.133 to 1.35)	1.236 (1.133 to 1.35)	1.236 (1.133 to 1.35)	1.236 (1.133 to 1.35)	1.236 (1.133 to 1.35)	1.236 (1.133 to 1.35)	1.236 (1.133 to 1.35)	1.236 (1.133 to 1.35)	1.236 (1.133 to 1.35)	1.236 (1.133 to 1.35)	1.236 (1.133 to 1.35)	1.236 (1.133 to 1.35)
Ischaemic heart disease	20 g/day	Both	Both	1.688 (1.415 to 2.028)	1.622 (1.379 to 1.922)	1.529 (1.326 to 1.776)	1.45 (1.28 to 1.654)	1.387 (1.243 to 1.558)	1.318 (1.202 to 1.455)	1.242 (1.156 to 1.342)	1.184 (1.119 to 1.258)	1.147 (1.096 to 1.205)	1.118 (1.077 to 1.163)	1.097 (1.064 to 1.135)	1.133 (1.087 to 1.185)	1.133 (1.087 to 1.185)	1.133 (1.087 to 1.185)	1.133 (1.087 to 1.185)
**Diet low in calcium**																		
Colon and rectum cancer	1 g/day	Both	Both	1.372 (1.268 to 1.485)	1.372 (1.268 to 1.485)	1.372 (1.268 to 1.485)	1.372 (1.268 to 1.485)	1.372 (1.268 to 1.485)	1.372 (1.268 to 1.485)	1.372 (1.268 to 1.485)	1.372 (1.268 to 1.485)	1.372 (1.268 to 1.485)	1.372 (1.268 to 1.485)	1.372 (1.268 to 1.485)	1.372 (1.268 to 1.485)	1.372 (1.268 to 1.485)	1.372 (1.268 to 1.485)	1.372 (1.268 to 1.485)
**Diet low in seafood omega-3 fatty acids**																		
Ischaemic heart disease	100 mg/day	Morbidity	Both	1.0 (1.0 to 1.0)	1.0 (1.0 to 1.0)	1.0 (1.0 to 1.0)	1.0 (1.0 to 1.0)	1.0 (1.0 to 1.0)	1.0 (1.0 to 1.0)	1.0 (1.0 to 1.0)	1.0 (1.0 to 1.0)	1.0 (1.0 to 1.0)	1.0 (1.0 to 1.0)	1.0 (1.0 to 1.0)	1.0 (1.0 to 1.0)	1.0 (1.0 to 1.0)	1.0 (1.0 to 1.0)	1.0 (1.0 to 1.0)
Ischaemic heart disease	100 mg/day	Mortality	Both	1.291 (1.109 to 1.505)	1.249 (1.094 to 1.428)	1.199 (1.077 to 1.338)	1.173 (1.067 to 1.293)	1.165 (1.064 to 1.279)	1.154 (1.06 to 1.26)	1.14 (1.055 to 1.235)	1.126 (1.05 to 1.211)	1.113 (1.045 to 1.189)	1.101 (1.04 to 1.167)	1.088 (1.035 to 1.145)	1.062 (1.025 to 1.102)	1.062 (1.025 to 1.102)	1.062 (1.025 to 1.102)	1.062 (1.025 to 1.102)
**Diet low in polyunsaturated fatty acids**																		
Ischaemic heart disease	5% energy/day	Both	Both	1.267 (1.098 to 1.452)	1.211 (1.079 to 1.352)	1.148 (1.056 to 1.244)	1.114 (1.044 to 1.186)	1.111 (1.043 to 1.181)	1.101 (1.039 to 1.165)	1.086 (1.033 to 1.14)	1.075 (1.029 to 1.121)	1.068 (1.026 to 1.11)	1.063 (1.025 to 1.102)	1.06 (1.024 to 1.097)	1.063 (1.025 to 1.102)	1.063 (1.025 to 1.102)	1.063 (1.025 to 1.102)	1.063 (1.025 to 1.102)
**Diet high in trans fatty acids**																		
Ischaemic heart disease	2% energy/day	Both	Both	1.901 (1.591 to 2.275)	1.775 (1.514 to 2.085)	1.615 (1.415 to 1.848)	1.517 (1.352 to 1.707)	1.461 (1.316 to 1.627)	1.396 (1.274 to 1.535)	1.323 (1.225 to 1.433)	1.264 (1.186 to 1.352)	1.222 (1.157 to 1.294)	1.186 (1.132 to 1.246)	1.158 (1.112 to 1.207)	1.15 (1.107 to 1.197)	1.15 (1.107 to 1.197)	1.15 (1.107 to 1.197)	1.15 (1.107 to 1.197)
**Diet high in sodium ***																		
	Non-Black, Non-Hypertensive	Both	Both	-1.366 (-1.937 to -0.795)	-1.882 (-2.434 to -1.330)	-2.397 (-2.967 to -1.828)	-2.913 (-3.533 to -2.292)	-3.428 (-4.126 to -2.730)	-3.944 (-4.738 to -3.150)	-4.459 (-5.362 to -3.556)	-4.975 (-5.995 to -3.954)	-5.490 (-6.634 to -4.347)	-5.490 (-6.634 to -4.347)	-5.490 (-6.634 to -4.347)	-5.490 (-6.634 to -4.347)	-5.490 (-6.634 to -4.347)	-5.490 (-6.634 to -4.347)	-5.490 (-6.634 to -4.347)
	Non-Black, Hypertensive	Both	Both	-3.300 (-4.147 to -2.454)	-3.816 (-4.547 to -3.085)	-4.331 (-4.959 to -3.704)	-4.847 (-5.389 to -4.305)	-5.363 (-5.848 to -4.877)	-5.878 (-6.346 to -5.411)	-6.394 (-6.886 to -5.901)	-6.909 (-7.464 to -6.354)	-7.425 (-8.069 to -6.781)	-7.425 (-8.069 to -6.781)	-7.425 (-8.069 to -6.781)	-7.425 (-8.069 to -6.781)	-7.425 (-8.069 to -6.781)	-7.425 (-8.069 to -6.781)	-7.425 (-8.069 to -6.781)
	Black, Non-Hypertensive	Both	Both	-3.910 (-5.065 to -2.755)	-4.426 (-5.564 to -3.287)	-4.941 (-6.081 to -3.802)	-5.457 (-6.616 to -4.298)	-5.972 (-7.168 to -4.777)	-6.488 (-7.735 to -5.241)	-7.004 (-8.316 to -5.691)	-7.519 (-8.909 to -6.129)	-8.035 (-9.512 to -6.557)	-8.035 (-9.512 to -6.557)	-8.035 (-9.512 to -6.557)	-8.035 (-9.512 to -6.557)	-8.035 (-9.512 to -6.557)	-8.035 (-9.512 to -6.557)	-8.035 (-9.512 to -6.557)
	Black, Hypertensive	Both	Both	-5.844 (-7.222 to -4.467)	-6.360 (-7.663 to -5.057)	-6.876 (-8.117 to -5.635)	-7.391 (-8.584 to -6.198)	-7.907 (-9.068 to -6.745)	-8.422 (-9.569 to -7.275)	-8.938 (-10.088 to -7.788)	-9.453 (-10.624 to -8.282)	-9.969 (-11.178 to -8.760)	-9.969 (-11.178 to -8.760)	-9.969 (-11.178 to -8.760)	-9.969 (-11.178 to -8.760)	-9.969 (-11.178 to -8.760)	-9.969 (-11.178 to -8.760)	-9.969 (-11.178 to -8.760)
Stomach cancer	1 g/day	Both	Both	1.199 (0.987 to 1.443)	1.205 (1.008 to 1.428)	1.205 (0.989 to 1.459)	1.202 (0.996 to 1.443)	1.209 (0.996 to 1.448)	1.198 (0.984 to 1.429)	1.204 (1.008 to 1.428)	1.2 (0.982 to 1.457)	1.206 (1.003 to 1.431)	1.21 (0.995 to 1.444)	1.203 (0.987 to 1.433)	1.205 (0.984 to 1.459)	1.205 (0.984 to 1.459)	1.205 (0.984 to 1.459)	1.205 (0.984 to 1.459)

*Shifts are reported for diet high in sodium as the estimation is based on mediation through high systolic blood pressure.

Supplemental Table 4. Number and age-standardized rates and proportions of deaths and DALYs attributable to dietary risks among adults at the global level in 1990 and 2017.

		DALYs		Deaths	
Measure	Cause	1990	2017	1990	2017
Number	All causes	184355646 (171862034-196608625	254522706 (234215999-274367024)	7665364.2 (7141997.4-8198274.3)	10885706 (10076218-11672383)
	Cardiovascular diseases	155244456 (144899447-166130728)	207217419 (192068188-222073215)	6792200.6 (6293917.6-7277048.8)	9497341 (8735272-10234688)
	Diabetes mellitus type 2	10330876 (6854803-14522666)	23718036 (15795731-33132598)	132620.8 (96688.5-173478.5)	338714 (244995-447003)
	Neoplasms	16737965 (13734027-19832724)	20178522 (16587934-24117338)	672283 (547026-800957)	913090 (743345-1098432)
Proportion	All causes	17.3 (16.0-18.6)	15.3 (14.1-16.6)	24.5 (23.1-26.0)	22.4 (21.0-23.8)
	Cardiovascular diseases	60.3 (56.9-63.5)	57.5 (54.0-60.6)	55.6 (52.1-59.0)	52.9 (49.3-56.2)
	Diabetes mellitus type 2	42.9 (33.4-52.8)	41.4 (32.1-51.4)	34.1 (26.0-42.8)	32.5 (24.5-41.1)
	Neoplasms	12.0 (10.1-13.8)	9.3 (7.9-10.7)	12.2 (10.3-14.2)	9.8 (8.3-11.3)
Rates	All causes	8535.9 (8063.4-9013.4)	6079.6 (5684.7-6472.5)	405.7 (381.4-429.6)	275.2 (257.9-291.8)
	Cardiovascular diseases	7239.6 (6821.8-7640.0)	4964.3 (4656.4-5263.2)	362.4 (338.7-385.7)	240.6 (224.4-255.9)
	Diabetes mellitus type 2	446.8 (317.6-597.9)	550.6 (393.9-736.5)	6.7 (5.1-8.4)	8.4 (6.4-10.7)
	Neoplasms	756.2 (644.2-872.8)	482.4 (412.7-555.3)	33.0 (27.8-38.1)	22.7 (19.1-26.3)

Supplemental Table 5. Age-standardized rates of mortality and DALYs attributable to dietary risks among adults at regional and SDI level in 1990 and 2017.

		DALYs		Deaths	
Location Name	Cause	1990	2017	1990	2017
High SDI	All causes	6052.3 (5686.0-6428.5)	3032.3 (2801.5-3265.2)	307.8 (288.1-327.2)	138.7 (129.2-148.3)
	Cardiovascular diseases	5008.5 (4695.8-5312.5)	2156.4 (2005.1-2306.3)	271.7 (252.4-290.6)	112.9 (104.2-121.6)
	Diabetes mellitus type 2	376.2 (280.3-490.4)	444.0 (324.2-586.5)	5.8 (4.5-7.1)	4.8 (3.8-5.8)
	Neoplasms	620.6 (530.4-716.2)	387.8 (320.8-459.8)	28.4 (24.1-32.8)	19.1 (15.7-22.6)
High-middle SDI	All causes	10776.3 (10182.8-11352.8)	6998.1 (6534.2-7454.4)	536.6 (505.2-567.2)	347.1 (323.5-369.1)
	Cardiovascular diseases	9186.7 (8677.0-9684.9)	5805.1 (5425.8-6182.4)	483.8 (453.8-513.1)	308.6 (285.8-329.9)
	Diabetes mellitus type 2	439.7 (312.0-592.3)	479.7 (340.6-641.9)	4.7 (3.6-6.0)	5.7 (4.3-7.2)
	Neoplasms	1049.2 (872.7-1224.4)	630.2 (529.2-730.5)	44.6 (36.8-52.3)	29.4 (24.4-34.4)
Middle SDI	All causes	8892.9 (8346.2-9455.7)	6772.7 (6322.4-7223.4)	414.0 (384.6-444.4)	316.8 (295.5-337.2)
	Cardiovascular diseases	7359.6 (6894.7-7827.5)	5492.8 (5132.4-5847.1)	361.3 (335.4-387.9)	275.0 (255.6-294.5)
	Diabetes mellitus type 2	470.6 (332.0-632.4)	605.8 (433.0-808.5)	7.6 (5.6-9.8)	10.7 (8.0-13.8)
	Neoplasms	912.4 (761.9-1061.2)	552.6 (468.7-640.4)	39.3 (32.6-46.2)	25.8 (21.5-30.1)
Low-middle SDI	All causes	9404.5 (8802.2-10011.0)	7796.9 (7264.9-8385.6)	417.5 (389.5-447.7)	343.9 (319.3-369.1)
	Cardiovascular diseases	8346.1 (7796.3-8928.1)	6685.4 (6228.2-7160.8)	385.5 (356.9-415.3)	310.9 (288.1-335.4)
	Diabetes mellitus type 2	509.5 (360.6-689.3)	681.2 (476.9-914.2)	9.2 (6.7-12.0)	13.9 (10.1-18.1)
	Neoplasms	451.9 (370.9-539.3)	343.4 (291.1-395.4)	19.0 (15.5-22.8)	15.5 (13.0-17.9)
Low SDI	All causes	7932.0 (7245.7-8609.7)	6870.4 (6390.4-7378.8)	347.8 (316.1-380.4)	299.5 (276.0-323.0)
	Cardiovascular diseases	6980.0 (6366.7-7628.6)	5891.6 (5460.3-6333.0)	318.5 (288.6-349.1)	270.2 (247.8-292.5)
	Diabetes mellitus type 2	495.8 (349.4-670.9)	604.3 (422.6-820.1)	9.9 (7.0-13.1)	12.3 (8.7-16.5)
	Neoplasms	393.3 (310.5-484.0)	324.2 (267.6-376.3)	16.8 (13.5-20.7)	14.7 (12.2-17.0)
Andean Latin America	All causes	5280.7 (4908.8-5675.2)	3158.0 (2846.8-3483.3)	250.5 (230.7-271.2)	143.5 (128.8-159.7)

	Cardiovascular diseases	4341.1 (4038.0-4641.1)	2213.0 (2016.6-2424.4)	218.2 (201.8-233.9)	112.7 (102.2-124.3)
	Diabetes mellitus type 2	448.1 (318.3-600.2)	585.9 (419.7-778.3)	7.8 (5.8-10.1)	11.3 (8.4-14.7)
	Neoplasms	411.1 (238.0-619.6)	276.4 (187.0-391.8)	20.4 (11.8-30.8)	14.6 (9.7-20.6)
Australasia	All causes	5554.6 (5113.9-6083.7)	2181.6 (1955.4-2443.8)	296.7 (272.9-325.7)	112.5 (101.3-125.2)
	Cardiovascular diseases	4688.0 (4317.4-5103.5)	1540.1 (1388.6-1700.5)	266.7 (244.4-292.2)	91.2 (81.9-101.0)
	Diabetes mellitus type 2	343.1 (249.0-449.3)	295.1 (210.4-395.8)	5.4 (4.2-6.7)	4.3 (3.3-5.4)
	Neoplasms	508.9 (409.9-621.5)	329.3 (258.4-412.0)	23.8 (19.4-28.8)	16.1 (12.7-20.1)
Caribbean	All causes	7769.1 (7104.8-8533.2)	5197.5 (4633.6-5902.1)	367.6 (337.7-397.8)	224.2 (200.8-250.4)
	Cardiovascular diseases	6564.7 (6003.1-7190.8)	4060.3 (3612.0-4565.0)	331.2 (302.6-359.7)	193.6 (173.0-217.7)
	Diabetes mellitus type 2	836.5 (594.6-1101.3)	866.2 (620.2-1139.1)	17.6 (13.1-22.6)	17.4 (12.9-22.3)
	Neoplasms	330.4 (277.4-386.6)	240.1 (196.8-300.7)	16.7 (14.0-19.5)	11.7 (9.6-14.4)
Central Asia	All causes	13401.4 (12712.5-14100.6)	12524.3 (11678.3-13353.3)	640.4 (602.2-676.6)	645.6 (598.9-689.4)
	Cardiovascular diseases	11858.1 (11242.1-12477.7)	11189.7 (10394.5-11939.8)	599.0 (560.8-634.8)	613.1 (566.0-657.6)
	Diabetes mellitus type 2	564.5 (394.9-769.5)	850.1 (614.4-1131.4)	3.5 (2.7-4.4)	11.1 (8.3-13.9)
	Neoplasms	907.4 (717.4-1102.5)	421.6 (324.2-527.5)	36.0 (28.0-43.9)	18.9 (14.7-23.5)
Central Europe	All causes	12582.3 (11963.1-13224.5)	6137.3 (5738.2-6566.4)	649.4 (613.7-682.7)	316.8 (295.1-338.7)
	Cardiovascular diseases	11160.5 (10585.6-11708.0)	5015.3 (4685.9-5349.6)	603.1 (568.2-636.5)	284.5 (263.5-305.3)
	Diabetes mellitus type 2	470.6 (338.4-629.1)	530.2 (377.5-715.4)	5.3 (4.1-6.6)	5.3 (4.1-6.6)
	Neoplasms	852.3 (717.0-987.1)	552.9 (452.5-664.6)	37.2 (31.3-42.7)	25.2 (20.8-30.2)
Central Latin America	All causes	5855.5 (5473.9-6272.2)	4269.2 (3916.7-4682.6)	272.8 (255.6-290.6)	186.0 (171.9-200.7)
	Cardiovascular diseases	4562.6 (4286.5-4874.9)	2842.0 (2620.6-3078.9)	236.7 (220.6-253.5)	148.1 (135.5-161.5)
	Diabetes mellitus type 2	975.8 (728.9-1257.4)	1124.3 (847.7-1443.1)	20.6 (15.8-26.1)	23.6 (18.5-29.5)
	Neoplasms	259.0 (190.0-350.8)	210.6 (173.5-254.5)	12.7 (9.2-17.5)	10.0 (8.2-12.2)

Central Sub-Saharan Africa	All causes	8580.6 (7558.5-9753.2)	7152.4 (6287.9-8127.0)	390.8 (342.6-443.5)	321.2 (283.3-362.8)
	Cardiovascular diseases	7303.0 (6402.2-8284.1)	5810.2 (5107.5-6606.4)	355.3 (311.4-402.7)	287.9 (251.9-325.6)
	Diabetes mellitus type 2	897.0 (626.5-1209.3)	1016.2 (708.5-1375.3)	18.4 (12.9-24.9)	18.1 (12.6-24.8)
	Neoplasms	350.8 (257.4-454.2)	289.8 (223.4-371.7)	15.6 (11.7-20.0)	13.2 (10.4-16.7)
East Asia	All causes	9510.0 (8824.2-10222.8)	6991.9 (6470.4-7478.8)	449.4 (410.6-489.2)	345.5 (319.5-371.5)
	Cardiovascular diseases	7497.4 (6910.6-8063.9)	5589.1 (5173.2-6004.6)	375.9 (341.1-410.4)	294.8 (271.5-319.0)
	Diabetes mellitus type 2	331.5 (223.4-456.3)	389.7 (273.5-529.6)	2.7 (1.9-3.6)	4.1 (3.1-5.2)
	Neoplasms	1510.0 (1257.1-1756.1)	878.0 (736.1-1022.7)	64.8 (53.6-76.3)	41.2 (33.8-48.6)
Eastern Europe	All causes	12352.2 (11656.2-13084.2)	10018.3 (9364.5-10717.7)	641.1 (600.8-680.9)	505.5 (468.5-541.0)
	Cardiovascular diseases	11143.8 (10455.1-11842.8)	9093.5 (8477.7-9723.6)	606.7 (565.2-647.8)	480.9 (444.3-515.9)
	Diabetes mellitus type 2	408.9 (288.7-551.2)	421.2 (297.7-568.7)	2.1 (1.7-2.6)	3.3 (2.5-4.0)
	Neoplasms	773.3 (589.2-981.9)	482.4 (404.8-561.9)	31.5 (24.2-39.8)	20.6 (17.3-24.0)
Eastern Sub-Saharan Africa	All causes	8702.5 (7777.1-9699.2)	5675.0 (5122.8-6220.2)	392.8 (348.3-443.4)	258.3 (229.1-289.7)
	Cardiovascular diseases	7451.4 (6641.8-8343.7)	4661.1 (4188.7-5166.2)	349.6 (308.2-395.6)	225.9 (198.8-254.3)
	Diabetes mellitus type 2	689.9 (491.5-922.7)	657.8 (461.3-886.8)	17.5 (12.4-23.6)	14.9 (10.2-20.6)
	Neoplasms	424.8 (335.3-516.4)	289.2 (231.8-351.6)	19.1 (15.2-23.1)	13.8 (11.1-16.7)
High-income Asia Pacific	All causes	5379.2 (5001.3-5755.6)	2271.4 (2068.9-2483.5)	265.8 (245.5-287.2)	97.2 (89.0-106.2)
	Cardiovascular diseases	3950.4 (3701.0-4217.1)	1443.3 (1328.9-1573.3)	210.1 (195.0-226.9)	68.4 (62.7-74.5)
	Diabetes mellitus type 2	278.1 (191.3-382.6)	289.5 (201.5-394.8)	2.7 (2.0-3.6)	2.0 (1.5-2.6)
	Neoplasms	1020.6 (827.6-1220.8)	477.8 (402.6-554.6)	46.9 (37.1-56.9)	24.3 (19.9-28.7)
High-income North America	All causes	6129.3 (5705.8-6586.7)	3838.4 (3539.9-4143.9)	301.7 (279.1-325.3)	166.0 (154.0-177.7)
	Cardiovascular diseases	5125.7 (4774.8-5465.5)	2795.2 (2599.9-2985.3)	270.6 (249.1-291.3)	138.9 (127.8-149.6)
	Diabetes mellitus type 2	486.7 (373.8-615.2)	622.8 (468.7-797.3)	7.5 (6.2-8.9)	7.8 (6.4-9.2)

	Neoplasms	490.3 (374.9-621.1)	358.2 (271.8-451.8)	22.6 (17.4-28.3)	16.9 (12.7-21.3)
North Africa and Middle East	All causes	11605.7 (10669.9-12495.5)	7169.7 (6600.5-7796.2)	533.3 (491.4-575.1)	324.1 (296.3-353.8)
	Cardiovascular diseases	10654.9 (9791.0-11511.5)	6208.2 (5704.0-6764.7)	507.6 (466.9-550.5)	301.8 (273.8-330.3)
	Diabetes mellitus type 2	621.4 (433.3-840.4)	709.7 (486.2-957.1)	11.0 (7.9-14.6)	10.6 (7.7-13.6)
	Neoplasms	274.9 (208.2-350.5)	203.4 (168.7-242.8)	12.1 (9.2-15.7)	9.4 (7.8-11.4)
Oceania	All causes	17475.0 (15847.0-19197.3)	17804.2 (16041.3-19906.8)	677.2 (621.1-737.4)	678.1 (615.7-746.0)
	Cardiovascular diseases	15251.8 (13801.5-16826.7)	14754.8 (13211.6-16512.4)	613.2 (561.2-668.9)	587.9 (532.0-648.2)
	Diabetes mellitus type 2	1632.9 (1181.3-2144.9)	2425.5 (1737.4-3197.7)	35.2 (26.0-45.3)	60.1 (43.6-78.1)
	Neoplasms	455.4 (363.3-554.4)	438.5 (344.4-544.1)	21.2 (16.8-25.8)	20.1 (15.9-24.9)
South Asia	All causes	8514.5 (7913.5-9112.3)	7573.7 (7017.9-8144.3)	371.5 (341.7-402.6)	332.0 (306.1-359.8)
	Cardiovascular diseases	7656.4 (7068.8-8237.0)	6596.9 (6103.0-7119.4)	345.9 (316.5-376.1)	302.8 (277.3-330.0)
	Diabetes mellitus type 2	434.7 (301.0-591.9)	604.3 (421.1-820.9)	8.0 (5.6-10.7)	13.2 (9.5-17.4)
	Neoplasms	356.0 (282.6-432.2)	301.2 (250.6-348.8)	15.0 (12.0-18.1)	13.2 (11.0-15.2)
Southeast Asia	All causes	9621.6 (9012.1-10253.1)	7191.8 (6669.1-7758.0)	430.5 (401.7-460.2)	315.0 (292.9-337.6)
	Cardiovascular diseases	8149.4 (7652.6-8659.8)	5874.6 (5486.1-6305.8)	381.0 (355.7-408.5)	274.2 (254.1-294.7)
	Diabetes mellitus type 2	610.6 (412.5-837.7)	777.2 (532.6-1064.7)	11.9 (8.4-15.9)	14.8 (10.3-19.9)
	Neoplasms	617.5 (519.9-717.5)	386.7 (328.6-447.3)	27.3 (22.9-31.7)	18.5 (15.5-21.4)
Southern Latin America	All causes	7525.0 (7081.0-7978.9)	4047.6 (3667.5-4425.1)	370.6 (347.1-394.3)	189.3 (172.3-207.2)
	Cardiovascular diseases	6284.8 (5911.7-6648.4)	2934.7 (2672.6-3193.9)	328.7 (306.7-350.4)	154.5 (140.9-169.1)
	Diabetes mellitus type 2	573.4 (427.6-745.3)	628.2 (467.3-815.9)	9.8 (7.8-12.1)	10.2 (8.1-12.6)
	Neoplasms	598.2 (481.4-717.5)	422.0 (354.2-493.3)	28.6 (22.9-34.7)	21.1 (17.7-24.7)
Southern Sub-Saharan Africa	All causes	5663.6 (5130.0-6236.1)	5521.0 (5017.2-6075.2)	234.2 (209.7-260.1)	241.6 (220.0-262.6)
	Cardiovascular diseases	4406.5 (3976.4-4855.3)	3871.6 (3546.8-4202.5)	198.0 (175.5-221.7)	192.3 (173.7-211.5)

	Diabetes mellitus type 2	775.6 (548.5-1037.3)	1213.0 (872.6-1596.7)	16.0 (11.2-21.2)	29.5 (21.4-38.5)
	Neoplasms	433.3 (321.2-548.0)	380.5 (288.1-476.5)	18.4 (13.8-23.1)	17.2 (13.1-21.5)
Tropical Latin America	All causes	8093.3 (7541.9-8634.9)	4196.9 (3883.8-4511.4)	372.6 (345.6-398.5)	184.2 (170.3-198.6)
	Cardiovascular diseases	7065.5 (6570.5-7536.8)	3291.4 (3042.4-3530.1)	337.9 (311.1-363.0)	154.1 (141.3-166.4)
	Diabetes mellitus type 2	557.8 (402.6-726.5)	553.7 (415.8-715.1)	12.6 (9.5-16.1)	13.2 (10.1-16.6)
	Neoplasms	397.8 (302.1-500.0)	288.0 (244.3-333.3)	18.9 (14.2-24.1)	13.7 (11.5-16.0)
Western Europe	All causes	5313.3 (4947.1-5729.1)	2441.5 (2229.9-2678.8)	283.0 (261.4-304.8)	121.0 (111.5-130.9)
	Cardiovascular diseases	4488.8 (4162.3-4814.6)	1718.9 (1581.0-1859.3)	253.3 (233.2-273.6)	99.7 (91.2-108.6)
	Diabetes mellitus type 2	327.5 (240.3-433.0)	376.3 (267.6-507.0)	5.7 (4.4-7.2)	3.9 (3.0-4.9)
	Neoplasms	478.0 (397.3-561.6)	327.6 (263.3-399.8)	23.0 (19.1-27.0)	16.2 (13.1-19.6)
Western Sub-Saharan Africa	All causes	5618.7 (4953.4-6333.8)	5020.0 (4462.2-5613.5)	271.2 (237.3-307.3)	234.6 (206.9-264.3)
	Cardiovascular diseases	4964.5 (4398.6-5594.0)	4230.1 (3749.4-4762.3)	250.8 (219.8-284.4)	211.1 (185.5-238.7)
	Diabetes mellitus type 2	389.6 (271.4-521.9)	532.1 (368.2-725.1)	7.8 (5.6-10.3)	10.9 (7.9-14.7)
	Neoplasms	223.7 (174.4-278.5)	211.9 (169.2-258.6)	10.8 (8.5-13.6)	10.6 (8.4-12.9)

Supplemental Table 6. Age-standardized proportions of mortality and DALYs attributable to dietary risks among adults at regional and SDI level in 1990 and 2017.

		DALYs		Deaths	
Location Name	Cause	1990	2017	1990	2017
High SDI	All causes	15.9 (14.5-17.4)	10.1 (9.3-11.1)	25.1 (23.5-26.7)	17.1 (15.9-18.2)
	Cardiovascular diseases	55.1 (51.7-58.3)	48.6 (45.5-51.7)	51.8 (48.1-55.3)	45.8 (42.4-49.2)
	Diabetes mellitus type 2	44.7 (36.8-53.6)	46.2 (38.1-54.9)	35.2 (28.0-43.1)	35.4 (28.4-43.0)
	Neoplasms	8.9 (7.6-10.2)	7.4 (6.1-8.8)	9.2 (7.8-10.5)	7.8 (6.5-9.3)
High-middle SDI	All causes	22.4 (20.7-24.0)	19.0 (17.4-20.6)	31.8 (29.9-33.5)	28.9 (27.1-30.7)
	Cardiovascular diseases	63.0 (59.5-66.3)	58.0 (54.5-61.2)	58.2 (54.6-61.6)	53.9 (50.3-57.6)
	Diabetes mellitus type 2	44.8 (35.2-55.0)	42.7 (33.6-52.6)	34.9 (26.6-44.0)	32.2 (24.5-40.8)
	Neoplasms	14.6 (12.2-17.1)	11.0 (9.3-12.6)	15.2 (12.6-17.9)	11.6 (9.7-13.5)
Middle SDI	All causes	18.5 (17.1-20.1)	17.5 (16.1-18.9)	24.6 (22.9-26.3)	25.0 (23.3-26.5)
	Cardiovascular diseases	63.1 (59.2-66.7)	59.7 (56.0-63.0)	57.6 (53.5-61.6)	54.8 (51.1-58.5)
	Diabetes mellitus type 2	40.9 (31.1-51.3)	39.3 (30.1-49.3)	32.0 (23.7-41.0)	30.9 (23.1-39.6)
	Neoplasms	15.7 (13.1-18.2)	11.0 (9.3-12.7)	16.2 (13.4-19.1)	11.7 (9.8-13.6)
Low-middle SDI	All causes	15.3 (14.2-16.5)	15.4 (14.1-16.6)	19.9 (18.6-21.3)	20.6 (19.2-22.0)
	Cardiovascular diseases	60.7 (57.2-64.2)	57.9 (54.2-61.7)	55.7 (52.2-59.3)	53.2 (49.5-57.0)
	Diabetes mellitus type 2	41.5 (31.2-52.1)	39.4 (29.5-49.8)	33.5 (24.6-42.9)	31.6 (23.1-40.7)
	Neoplasms	9.8 (8.1-11.7)	7.5 (6.4-8.7)	10.1 (8.3-12.0)	8.0 (6.8-9.3)
Low SDI	All causes	10.6 (9.6-11.6)	12.5 (11.4-13.5)	13.5 (12.3-14.7)	16.1 (14.8-17.4)
	Cardiovascular diseases	56.3 (52.5-59.8)	56.2 (52.4-60.0)	51.7 (47.8-55.3)	51.3 (47.4-54.9)
	Diabetes mellitus type 2	40.2 (29.9-50.7)	39.1 (28.9-49.6)	32.7 (23.8-42.2)	30.5 (22.0-39.4)
	Neoplasms	8.0 (6.4-9.8)	7.4 (6.1-8.5)	8.4 (6.7-10.2)	7.9 (6.6-9.1)
Andean Latin America	All causes	12.8 (11.8-14.0)	10.0 (9.1-11.0)	19.1 (17.6-20.6)	15.4 (14.2-16.9)
	Cardiovascular diseases	57.9 (54.4-61.3)	51.7 (48.4-55.2)	54.5 (50.7-58.2)	49.8 (46.1-53.4)
	Diabetes mellitus type 2	39.8 (30.3-49.9)	38.7 (29.6-48.7)	32.7 (24.4-41.8)	31.3 (23.2-40.1)
	Neoplasms	8.1 (4.7-12.2)	6.4 (4.4-8.9)	9.0 (5.2-13.7)	7.3 (4.9-10.4)
Australasia	All causes	15.1 (13.4-16.9)	7.7 (6.8-8.7)	25.3 (23.3-27.8)	15.2 (14.0-16.8)
	Cardiovascular diseases	53.3 (49.2-57.8)	43.7 (40.1-47.4)	50.6 (46.3-55.2)	41.8 (38.1-45.8)
	Diabetes mellitus type 2	41.6 (32.9-50.4)	40.9 (32.4-49.7)	33.1 (25.7-41.0)	31.1 (24.0-38.7)

	Neoplasms	7.4 (6.0-9.1)	6.4 (5.1-8.0)	7.8 (6.3-9.5)	6.8 (5.4-8.4)
Caribbean	All causes	17.4 (15.7-19.2)	13.0 (11.5-14.6)	25.1 (23.1-27.1)	18.5 (16.8-20.4)
	Cardiovascular diseases	54.0 (49.4-58.7)	46.5 (42.1-51.6)	51.7 (47.4-56.0)	43.8 (39.6-48.4)
	Diabetes mellitus type 2	40.7 (30.9-51.1)	39.6 (30.2-49.7)	33.6 (25.2-43.0)	32.5 (24.2-41.5)
	Neoplasms	6.0 (5.0-7.0)	4.4 (3.6-5.4)	6.7 (5.7-7.8)	4.8 (3.9-5.9)
Central Asia	All causes	27.0 (24.9-29.1)	26.1 (23.9-28.2)	39.2 (36.9-41.5)	38.0 (35.4-40.5)
	Cardiovascular diseases	67.3 (63.8-70.6)	60.7 (56.8-64.4)	63.9 (60.0-67.6)	58.3 (54.2-62.3)
	Diabetes mellitus type 2	47.8 (37.9-58.0)	46.3 (36.8-56.5)	41.2 (31.8-50.9)	40.1 (31.1-49.7)
	Neoplasms	14.7 (11.6-17.8)	8.6 (6.6-10.7)	15.0 (11.7-18.4)	9.3 (7.2-11.4)
Central Europe	All causes	25.1 (23.2-27.0)	16.3 (14.8-17.7)	37.2 (35.1-39.1)	26.6 (24.9-28.4)
	Cardiovascular diseases	64.4 (61.2-67.3)	51.9 (48.8-55.2)	60.9 (57.4-64.3)	49.5 (46.0-53.0)
	Diabetes mellitus type 2	46.3 (37.3-56.0)	45.4 (36.7-54.8)	37.7 (29.4-46.6)	33.9 (26.3-42.2)
	Neoplasms	11.1 (9.3-12.9)	8.2 (6.7-9.8)	11.9 (10.0-13.6)	8.6 (7.1-10.3)
Central Latin America	All causes	13.9 (12.9-14.9)	12.2 (11.2-13.2)	20.0 (18.7-21.3)	18.0 (16.7-19.4)
	Cardiovascular diseases	56.3 (52.9-59.7)	52.0 (48.1-56.1)	53.1 (49.6-56.8)	49.7 (45.7-53.9)
	Diabetes mellitus type 2	40.9 (32.3-50.4)	41.0 (32.8-50.0)	33.8 (26.1-42.5)	33.3 (26.2-41.5)
	Neoplasms	5.7 (4.1-7.6)	5.4 (4.5-6.6)	6.2 (4.5-8.6)	5.8 (4.8-7.2)
Central Sub-Saharan Africa	All causes	10.3 (9.2-11.6)	10.9 (9.7-12.3)	14.1 (12.6-15.8)	14.8 (13.2-16.5)
	Cardiovascular diseases	50.5 (45.6-55.9)	50.3 (45.7-55.0)	47.2 (42.2-52.6)	46.8 (42.2-51.5)
	Diabetes mellitus type 2	38.9 (28.9-49.2)	39.6 (29.6-49.9)	31.9 (23.3-41.1)	32.1 (23.5-41.4)
	Neoplasms	6.3 (4.7-8.2)	6.1 (4.8-7.7)	6.7 (5.0-8.6)	6.5 (5.1-8.2)
East Asia	All causes	20.6 (18.9-22.4)	21.0 (19.2-22.9)	25.9 (23.8-28.2)	29.8 (27.6-31.9)
	Cardiovascular diseases	65.3 (60.5-69.6)	64.3 (60.0-68.4)	58.5 (53.4-63.6)	58.1 (53.7-62.3)
	Diabetes mellitus type 2	44.9 (34.3-55.7)	44.5 (34.9-54.6)	34.5 (25.5-43.9)	32.6 (24.6-41.2)
	Neoplasms	20.4 (16.9-23.6)	14.8 (12.4-17.1)	21.2 (17.5-25.0)	15.5 (12.8-18.2)
Eastern Europe	All causes	23.0 (21.1-24.8)	19.7 (18.0-21.3)	35.9 (33.7-38.1)	32.0 (29.7-34.3)
	Cardiovascular diseases	61.6 (58.0-65.2)	55.5 (51.9-59.2)	58.3 (54.4-62.1)	54.2 (50.2-58.1)
	Diabetes mellitus type 2	49.7 (40.6-59.1)	45.8 (36.7-55.5)	40.7 (32.2-49.6)	36.7 (28.6-45.6)
	Neoplasms	10.7 (8.2-13.6)	7.9 (6.6-9.2)	11.2 (8.6-14.2)	8.5 (7.1-9.8)

Eastern Sub-Saharan Africa	All causes	10.0 (8.9-11.2)	10.0 (9.0-11.0)	13.5 (11.9-15.2)	13.8 (12.2-15.4)
	Cardiovascular diseases	54.7 (50.1-59.7)	52.2 (47.8-56.3)	50.4 (45.4-55.6)	48.7 (44.0-53.2)
	Diabetes mellitus type 2	38.5 (28.6-48.7)	37.5 (27.8-47.9)	32.2 (23.4-41.7)	30.8 (22.3-40.0)
	Neoplasms	6.9 (5.5-8.3)	5.7 (4.6-7.0)	7.6 (6.1-9.1)	6.4 (5.2-7.8)
High-income Asia Pacific	All causes	16.3 (14.8-18.0)	9.2 (8.3-10.2)	24.6 (22.7-26.6)	15.2 (13.9-16.6)
	Cardiovascular diseases	56.6 (53.1-60.1)	48.5 (45.2-51.9)	51.7 (47.9-55.8)	43.7 (40.2-47.6)
	Diabetes mellitus type 2	39.6 (30.3-49.3)	42.0 (33.6-51.3)	31.4 (23.3-40.2)	30.7 (23.4-39.0)
	Neoplasms	16.7 (13.6-20.1)	10.9 (9.2-12.8)	17.0 (13.5-20.6)	11.4 (9.3-13.4)
High-income North America	All causes	15.4 (13.9-16.8)	10.9 (10.0-11.8)	25.4 (23.5-27.4)	17.6 (16.4-18.8)
	Cardiovascular diseases	56.5 (52.7-60.1)	50.8 (47.4-54.2)	54.5 (50.2-58.6)	48.5 (44.8-52.1)
	Diabetes mellitus type 2	50.9 (43.6-58.9)	49.8 (42.2-58.0)	41.4 (34.5-49.0)	40.6 (33.7-48.2)
	Neoplasms	6.9 (5.2-8.6)	6.6 (5.0-8.3)	7.3 (5.6-9.1)	6.8 (5.1-8.6)
North Africa and Middle East	All causes	22.6 (20.4-24.8)	17.9 (16.1-19.8)	32.1 (29.6-34.6)	27.3 (25.1-29.6)
	Cardiovascular diseases	61.0 (56.7-65.5)	54.5 (50.3-58.8)	56.6 (52.3-61.0)	50.2 (45.8-54.6)
	Diabetes mellitus type 2	41.3 (31.4-51.9)	39.6 (30.0-50.2)	33.7 (24.9-43.3)	31.8 (23.3-41.0)
	Neoplasms	6.3 (4.8-8.2)	5.3 (4.4-6.3)	6.7 (5.1-8.6)	5.7 (4.7-6.9)
Oceania	All causes	19.8 (18.3-21.3)	22.0 (20.4-23.6)	23.0 (21.4-24.6)	25.3 (23.7-27.0)
	Cardiovascular diseases	60.2 (56.2-64.0)	62.8 (58.9-66.5)	57.5 (53.7-61.4)	59.5 (55.8-63.2)
	Diabetes mellitus type 2	44.2 (33.8-54.8)	43.3 (33.1-53.8)	37.3 (27.9-47.0)	36.8 (27.7-46.3)
	Neoplasms	7.2 (5.8-8.7)	6.9 (5.6-8.4)	8.5 (6.7-10.3)	7.9 (6.4-9.6)
South Asia	All causes	13.4 (12.3-14.4)	15.1 (13.7-16.4)	16.5 (15.2-17.8)	19.2 (17.6-20.7)
	Cardiovascular diseases	60.1 (56.3-63.9)	58.1 (54.0-62.3)	55.0 (51.2-58.7)	52.7 (48.6-57.0)
	Diabetes mellitus type 2	41.2 (30.8-51.9)	38.3 (28.5-48.6)	32.6 (23.7-42.0)	29.7 (21.6-38.6)
	Neoplasms	8.8 (7.0-10.7)	7.2 (6.0-8.4)	9.2 (7.4-11.0)	7.6 (6.4-8.8)
Southeast Asia	All causes	18.0 (16.6-19.3)	16.9 (15.5-18.2)	23.3 (21.8-24.8)	22.2 (20.8-23.7)
	Cardiovascular diseases	65.7 (61.9-69.4)	58.0 (54.2-61.5)	60.4 (56.5-64.1)	53.8 (50.1-57.4)
	Diabetes mellitus type 2	37.8 (27.4-48.5)	35.2 (25.3-45.8)	31.2 (22.1-40.8)	28.7 (20.2-38.1)
	Neoplasms	11.6 (9.7-13.4)	8.0 (6.8-9.2)	12.5 (10.5-14.4)	8.9 (7.5-10.2)
Southern Latin America	All causes	17.7 (16.2-19.1)	11.7 (10.7-12.7)	25.8 (24.1-27.5)	17.7 (16.5-19.0)

	Cardiovascular diseases	56.1 (52.8-59.3)	48.8 (45.5-52.1)	53.1 (49.6-56.6)	46.1 (42.7-49.6)
	Diabetes mellitus type 2	47.4 (38.8-56.6)	46.8 (38.6-55.7)	39.7 (31.6-48.2)	37.8 (30.4-45.9)
	Neoplasms	8.2 (6.6-9.9)	7.3 (6.2-8.4)	8.9 (7.1-10.8)	7.9 (6.7-9.2)
Southern Sub-Saharan Africa	All causes	9.4 (8.5-10.4)	8.4 (7.7-9.2)	13.7 (12.3-15.2)	12.9 (11.8-14.1)
	Cardiovascular diseases	50.0 (46.1-54.0)	48.6 (44.6-52.6)	45.7 (41.5-49.8)	44.8 (40.8-48.9)
	Diabetes mellitus type 2	38.3 (28.6-48.6)	38.0 (28.5-48.0)	31.7 (23.0-41.2)	31.6 (23.1-40.9)
	Neoplasms	7.4 (5.5-9.6)	7.1 (5.4-8.9)	7.6 (5.7-9.7)	7.3 (5.7-9.2)
Tropical Latin America	All causes	16.9 (15.4-18.4)	11.1 (10.1-12.2)	23.8 (22.1-25.5)	16.8 (15.6-18.1)
	Cardiovascular diseases	55.4 (51.6-59.2)	47.3 (43.8-50.7)	51.1 (46.9-54.8)	44.4 (40.8-48.1)
	Diabetes mellitus type 2	39.5 (30.7-49.4)	39.6 (31.3-49.2)	32.1 (24.2-41.0)	31.4 (24.1-40.1)
	Neoplasms	7.6 (5.7-9.5)	6.1 (5.1-7.0)	8.2 (6.2-10.5)	6.6 (5.5-7.6)
Western Europe	All causes	14.3 (12.9-15.7)	8.7 (7.8-9.5)	22.9 (21.2-24.7)	15.5 (14.3-16.7)
	Cardiovascular diseases	50.8 (47.2-54.5)	44.0 (40.8-47.1)	47.5 (43.7-51.3)	41.9 (38.5-45.3)
	Diabetes mellitus type 2	42.0 (33.9-51.0)	44.2 (35.8-53.1)	32.2 (25.1-40.1)	31.6 (24.7-39.2)
	Neoplasms	6.7 (5.6-7.9)	6.1 (4.9-7.5)	7.2 (6.0-8.4)	6.5 (5.3-7.8)
Western Sub-Saharan Africa	All causes	8.8 (7.8-9.7)	9.0 (8.1-9.9)	12.6 (11.3-14.0)	13.1 (11.9-14.4)
	Cardiovascular diseases	50.4 (46.2-54.7)	50.5 (46.4-54.6)	46.8 (42.6-51.3)	47.0 (42.8-51.0)
	Diabetes mellitus type 2	39.5 (29.8-49.9)	37.2 (27.6-47.6)	32.0 (23.7-41.3)	30.0 (21.9-39.2)
	Neoplasms	4.5 (3.5-5.5)	4.3 (3.6-5.2)	4.9 (3.9-6.1)	4.8 (3.9-5.7)

Supplemental Table 7. Age-standardized rates of all-cause mortality and DALYs attributable to dietary risks among adults at the national level in 1990 and 2017.					
Location Name	Cause	DALYs		Deaths	
		1990	2017	1990	2017
Afghanistan	All causes	22058.5 (14826.7-26416.9)	17966.0 (15817.9-20329.5)	948.4 (702.9-1112.1)	750.5 (666.8-838.5)
	Cardiovascular diseases	20382.5 (13612.6-24485.7)	16024.6 (14010.9-18120.2)	902.1 (671.6-1061.6)	698.2 (618.6-783.2)
	Diabetes mellitus type 2	1005.7 (664.3-1446.4)	1296.8 (883.3-1765.8)	19.0 (10.3-30.0)	25.7 (16.1-36.6)
	Neoplasms	593.7 (309.1-945.8)	585.4 (352.8-873.8)	24.0 (13.5-37.4)	24.0 (15.2-35.5)
Albania	All causes	8122.8 (7625.8-8629.8)	5369.7 (4622.6-6231.9)	410.6 (383.8-438.0)	288.5 (245.7-336.3)
	Cardiovascular diseases	7137.5 (6721.8-7587.0)	4756.3 (4070.1-5533.3)	379.1 (354.3-405.4)	273.3 (232.6-318.1)
	Diabetes mellitus type 2	269.0 (186.0-370.9)	314.7 (214.8-429.6)	1.5 (1.1-1.9)	1.6 (1.2-2.2)
	Neoplasms	594.2 (485.0-699.1)	255.4 (185.2-330.0)	25.4 (20.3-30.2)	11.7 (8.6-15.1)
Algeria	All causes	9933.9 (9031.7-10821.7)	5712.5 (5115.5-6295.4)	489.5 (443.5-535.7)	285.5 (256.0-315.1)
	Cardiovascular diseases	9156.3 (8299.4-10010.6)	4868.3 (4359.4-5401.7)	470.9 (425.8-518.0)	268.9 (239.6-298.6)
	Diabetes mellitus type 2	511.7 (359.1-695.7)	664.6 (455.0-910.2)	6.3 (4.2-8.6)	8.1 (5.9-10.5)
	Neoplasms	223.6 (178.6-269.5)	142.3 (118.0-167.9)	10.2 (8.1-12.4)	6.7 (5.5-7.9)
American Samoa	All causes	8760.0 (7812.2-9951.8)	8812.8 (7697.9-9965.3)	346.2 (313.4-385.0)	350.4 (314.6-389.3)
	Cardiovascular diseases	6526.2 (5819.8-7364.3)	5900.6 (5261.9-6596.1)	286.4 (256.2-319.7)	271.8 (242.7-301.9)
	Diabetes mellitus type 2	1897.0 (1377.7-2488.3)	2475.4 (1764.2-3239.4)	41.3 (30.7-53.2)	51.8 (37.4-67.6)
	Neoplasms	288.8 (230.2-354.6)	292.3 (235.1-355.5)	14.9 (11.7-18.3)	15.7 (12.4-19.0)
Andorra	All causes	3665.4 (3257.0-4123.1)	2206.3 (1956.1-2469.0)	185.2 (164.7-207.9)	101.8 (90.2-114.4)
	Cardiovascular diseases	3043.7 (2691.3-3423.2)	1590.7 (1398.5-1800.9)	165.0 (146.3-186.2)	86.0 (75.4-97.6)
	Diabetes mellitus type 2	268.7 (189.6-361.9)	340.4 (235.6-460.4)	2.4 (1.8-3.1)	1.7 (1.3-2.3)
	Neoplasms	338.5 (270.6-420.0)	261.0 (200.5-332.4)	17.1 (13.7-21.0)	13.4 (10.5-16.8)
Angola	All causes	9801.2 (8399.8-11261.6)	6122.3 (5321.5-7016.0)	439.3 (374.0-503.8)	275.9 (236.1-320.4)
	Cardiovascular diseases	8275.5 (6991.1-9593.8)	4727.5 (4077.3-5462.1)	395.9 (335.1-457.1)	240.1 (205.0-279.9)
	Diabetes mellitus type 2	1022.4 (732.2-1366.2)	1033.9 (713.0-1406.2)	21.1 (14.6-28.5)	18.2 (12.7-24.9)
	Neoplasms	454.4 (325.4-597.7)	284.9 (221.9-363.2)	19.7 (14.5-25.5)	13.1 (10.4-16.5)
Antigua and Barbuda	All causes	6491.1 (5903.5-7092.3)	3904.3 (3469.0-4405.6)	293.5 (268.9-318.3)	172.8 (155.9-190.5)
	Cardiovascular diseases	5092.9 (4655.9-5545.2)	2597.0 (2332.4-2885.3)	252.7 (230.4-274.6)	136.1 (121.6-151.1)

	Diabetes mellitus type 2	1079.8 (778.5-1400.2)	1068.7 (780.9-1387.2)	24.6 (18.2-32.1)	24.1 (17.8-31.3)
	Neoplasms	260.2 (203.2-337.2)	195.5 (164.0-229.6)	13.2 (10.1-17.2)	10.2 (8.6-12.1)
Argentina	All causes	7882.3 (7389.2-8364.5)	4307.7 (3861.0-4789.4)	384.3 (358.2-410.1)	203.1 (182.2-225.6)
	Cardiovascular diseases	6697.4 (6265.6-7106.8)	3218.3 (2883.4-3556.9)	344.6 (319.4-368.9)	169.9 (152.0-188.9)
	Diabetes mellitus type 2	561.7 (420.5-725.7)	630.4 (465.5-824.1)	9.8 (7.8-12.1)	10.5 (8.2-13.1)
	Neoplasms	553.8 (461.1-647.1)	404.9 (341.0-471.5)	26.1 (21.8-30.8)	19.6 (16.4-22.8)
Armenia	All causes	11874.7 (11140.0-12625.1)	7787.8 (7206.6-8395.0)	614.2 (571.5-655.8)	406.5 (373.2-439.3)
	Cardiovascular diseases	10367.5 (9726.8-11010.7)	6391.3 (5909.2-6870.8)	575.5 (532.4-615.5)	369.0 (337.4-399.9)
	Diabetes mellitus type 2	691.2 (477.3-938.4)	943.6 (676.7-1256.2)	7.5 (5.3-10.3)	16.3 (12.1-20.9)
	Neoplasms	754.6 (595.1-919.1)	401.8 (318.6-484.2)	29.6 (23.2-36.3)	18.8 (14.9-22.6)
Australia	All causes	5406.9 (4968.6-5940.1)	2086.6 (1847.0-2364.3)	292.2 (268.2-322.2)	107.7 (96.3-121.6)
	Cardiovascular diseases	4556.9 (4192.0-4972.2)	1466.0 (1312.5-1635.7)	263.0 (240.4-289.4)	87.4 (77.8-97.6)
	Diabetes mellitus type 2	345.6 (250.5-453.7)	291.9 (207.1-392.4)	5.4 (4.2-6.8)	4.3 (3.3-5.4)
	Neoplasms	491.3 (393.5-605.3)	314.4 (242.3-399.7)	23.0 (18.6-28.1)	15.3 (11.9-19.3)
Austria	All causes	5333.9 (4938.5-5745.0)	2566.7 (2322.4-2827.2)	292.9 (268.8-317.5)	143.1 (129.4-157.8)
	Cardiovascular diseases	4606.4 (4252.3-4972.2)	1900.7 (1720.5-2084.6)	266.2 (242.8-289.4)	122.4 (109.8-135.9)
	Diabetes mellitus type 2	265.4 (187.6-359.9)	369.5 (260.8-503.5)	4.4 (3.2-5.8)	5.1 (3.9-6.4)
	Neoplasms	447.7 (379.1-520.2)	268.3 (215.9-328.1)	21.6 (18.3-25.2)	13.5 (10.9-16.2)
Azerbaijan	All causes	13310.7 (12552.9-14124.2)	12850.4 (11739.9-13927.8)	622.4 (581.0-664.6)	688.6 (629.8-747.1)
	Cardiovascular diseases	11925.6 (11237.1-12640.6)	11455.4 (10479.3-12411.2)	587.4 (547.0-627.7)	652.9 (595.4-710.5)
	Diabetes mellitus type 2	556.8 (383.3-777.8)	810.6 (579.5-1098.9)	3.4 (2.2-4.7)	9.3 (6.9-12.1)
	Neoplasms	757.3 (582.0-939.9)	515.3 (378.3-661.5)	29.6 (22.5-37.4)	23.3 (16.9-29.9)
Bahrain	All causes	11908.9 (10825.8-13107.3)	4322.9 (3785.4-4970.5)	585.2 (531.3-640.6)	190.5 (168.3-215.9)
	Cardiovascular diseases	10476.8 (9483.8-11532.3)	2663.5 (2341.6-2978.3)	534.6 (480.1-589.0)	134.1 (117.8-150.8)
	Diabetes mellitus type 2	1032.0 (726.8-1348.2)	1470.1 (1065.9-1929.1)	29.6 (21.2-38.5)	46.6 (33.2-60.9)
	Neoplasms	296.4 (231.3-360.1)	125.7 (102.6-155.3)	15.2 (11.7-18.8)	6.5 (5.3-8.0)
Bangladesh	All causes	6378.2 (5606.8-7300.0)	7754.5 (6967.6-8599.4)	284.1 (247.0-328.9)	344.4 (308.8-383.3)
	Cardiovascular diseases	5449.9 (4761.2-6280.2)	6791.6 (6101.9-7533.6)	254.6 (219.9-296.3)	310.6 (277.7-346.3)

	Diabetes mellitus type 2	370.5 (242.7-521.2)	502.2 (336.1-708.5)	6.6 (4.2-9.5)	12.6 (8.3-17.4)
	Neoplasms	528.6 (409.4-647.5)	393.0 (312.3-478.4)	21.7 (17.0-26.7)	18.1 (14.4-22.1)
Barbados	All causes	6610.4 (6071.5-7184.3)	3897.6 (3438.2-4422.4)	324.9 (300.4-348.5)	170.7 (153.6-187.8)
	Cardiovascular diseases	4863.6 (4500.9-5244.7)	2402.9 (2152.5-2668.6)	266.7 (245.6-287.0)	125.7 (112.9-138.8)
	Diabetes mellitus type 2	1308.9 (986.1-1675.1)	1149.0 (825.5-1511.9)	35.6 (27.1-45.0)	27.0 (19.9-35.0)
	Neoplasms	393.8 (330.5-462.9)	315.5 (263.0-378.6)	20.2 (16.7-24.4)	16.3 (13.6-19.2)
Belarus	All causes	11636.2 (10983.8-12304.0)	9875.3 (9091.2-10707.3)	588.9 (551.3-623.5)	526.6 (482.8-570.7)
	Cardiovascular diseases	10438.4 (9786.9-11050.1)	9054.6 (8272.6-9854.5)	554.9 (516.8-590.9)	505.8 (462.0-548.9)
	Diabetes mellitus type 2	410.0 (290.7-554.6)	376.0 (259.5-515.5)	2.3 (1.8-2.8)	1.3 (1.0-1.6)
	Neoplasms	760.3 (527.6-1015.8)	428.3 (348.6-509.3)	30.9 (21.4-41.5)	18.9 (15.4-22.7)
Belgium	All causes	5302.4 (4916.0-5738.4)	2442.3 (2198.3-2734.9)	276.0 (254.8-297.0)	113.7 (103.4-124.3)
	Cardiovascular diseases	4318.5 (4004.4-4629.1)	1627.7 (1487.6-1777.8)	241.4 (221.9-261.4)	92.3 (83.4-101.7)
	Diabetes mellitus type 2	393.4 (279.2-529.2)	444.4 (309.9-611.2)	5.0 (3.8-6.3)	2.8 (2.2-3.5)
	Neoplasms	567.8 (450.3-699.4)	353.6 (267.6-453.7)	28.3 (22.7-34.3)	17.6 (13.6-22.3)
Belize	All causes	6539.4 (5974.2-7154.8)	4625.0 (4113.8-5213.3)	315.3 (289.1-341.3)	188.6 (172.0-207.5)
	Cardiovascular diseases	5375.5 (4884.1-5852.0)	3082.1 (2794.9-3392.8)	283.0 (257.7-308.0)	147.3 (133.8-161.9)
	Diabetes mellitus type 2	906.8 (644.3-1200.7)	1283.0 (931.5-1656.0)	18.7 (13.7-24.3)	29.0 (21.2-37.0)
	Neoplasms	212.6 (166.2-276.6)	217.8 (179.7-258.0)	11.1 (8.4-14.7)	10.1 (8.4-11.9)
Benin	All causes	6201.4 (5554.2-6946.6)	5331.2 (4589.8-6204.8)	290.7 (259.5-325.6)	241.8 (208.1-279.6)
	Cardiovascular diseases	5411.4 (4821.7-6084.4)	4357.0 (3714.2-5111.5)	267.4 (236.4-299.9)	214.5 (183.4-250.0)
	Diabetes mellitus type 2	481.5 (335.9-648.7)	653.3 (438.0-908.0)	9.2 (6.5-12.3)	12.1 (8.3-16.7)
	Neoplasms	251.4 (186.0-330.8)	266.7 (192.3-342.3)	11.7 (8.6-15.7)	12.8 (9.3-16.5)
Bermuda	All causes	6840.2 (6193.9-7503.0)	2592.0 (2293.2-2914.3)	341.9 (312.0-373.9)	121.4 (108.2-134.8)
	Cardiovascular diseases	5766.0 (5199.2-6346.5)	1808.3 (1588.1-2016.7)	307.3 (278.1-338.2)	101.2 (88.7-113.0)
	Diabetes mellitus type 2	673.9 (499.4-876.3)	574.3 (422.9-762.8)	13.5 (10.3-17.3)	8.5 (6.4-10.7)
	Neoplasms	351.9 (298.3-415.7)	181.2 (145.2-231.9)	18.5 (15.6-21.6)	10.2 (8.1-12.6)
Bhutan	All causes	7355.7 (6375.8-8487.0)	5639.6 (4769.6-6545.5)	325.6 (279.6-381.6)	259.9 (218.8-302.8)
	Cardiovascular diseases	6521.8 (5582.0-7589.9)	4735.2 (3969.8-5550.2)	301.3 (258.0-355.3)	231.4 (194.2-270.2)

	Diabetes mellitus type 2	410.1 (277.0-566.0)	492.5 (333.8-680.4)	6.2 (4.2-8.5)	9.4 (6.2-12.9)
	Neoplasms	371.2 (276.9-467.7)	291.2 (227.0-357.9)	15.8 (11.9-19.9)	13.7 (10.9-16.8)
Bolivia	All causes	8860.6 (7948.2-9902.1)	5109.3 (4395.8-5880.7)	410.1 (367.4-454.2)	249.3 (212.4-287.9)
	Cardiovascular diseases	7347.7 (6499.2-8301.7)	3761.2 (3186.2-4421.9)	357.7 (319.1-398.8)	202.3 (172.8-235.6)
	Diabetes mellitus type 2	769.2 (539.7-1036.5)	812.1 (572.8-1101.0)	16.1 (11.5-21.8)	17.5 (12.3-23.8)
	Neoplasms	624.4 (301.3-1004.6)	421.2 (256.2-626.6)	30.4 (14.8-48.4)	22.6 (13.5-33.7)
Bosnia and Herzegovina	All causes	10663.9 (10034.7-11350.6)	6321.2 (5783.9-6942.9)	544.7 (510.5-578.6)	334.9 (306.6-365.2)
	Cardiovascular diseases	9401.5 (8837.2-9995.4)	5028.8 (4586.9-5480.2)	505.5 (471.8-537.4)	295.5 (267.6-322.8)
	Diabetes mellitus type 2	421.4 (283.6-590.1)	783.7 (550.8-1049.7)	3.9 (2.5-7.0)	15.8 (11.3-21.1)
	Neoplasms	720.2 (590.3-853.1)	460.5 (363.5-573.1)	30.8 (25.0-36.3)	21.4 (17.1-26.3)
Botswana	All causes	6683.1 (5794.3-7613.3)	5521.0 (4919.7-6188.0)	309.2 (268.4-353.8)	264.1 (236.4-295.2)
	Cardiovascular diseases	5272.3 (4529.6-6060.2)	3892.4 (3460.2-4362.6)	266.5 (231.3-305.0)	215.4 (191.6-242.6)
	Diabetes mellitus type 2	944.9 (669.2-1278.9)	1297.1 (936.9-1714.5)	22.1 (15.4-30.1)	32.1 (23.0-42.6)
	Neoplasms	415.8 (309.2-542.7)	274.5 (211.0-348.9)	18.5 (14.0-23.7)	13.8 (10.8-17.5)
Brazil	All causes	8141.1 (7586.1-8687.4)	4176.4 (3864.6-4485.7)	374.4 (347.0-400.9)	183.1 (168.9-197.1)
	Cardiovascular diseases	7110.1 (6609.5-7582.9)	3281.8 (3031.0-3522.0)	339.5 (312.7-364.9)	153.3 (140.4-165.7)
	Diabetes mellitus type 2	556.9 (401.6-725.0)	543.6 (408.3-703.1)	12.7 (9.5-16.2)	12.9 (9.9-16.3)
	Neoplasms	401.7 (304.8-504.7)	288.2 (244.5-333.9)	19.1 (14.3-24.4)	13.7 (11.4-16.0)
Brunei	All causes	12099.0 (11223.4-12987.0)	6613.5 (6030.5-7220.8)	549.2 (511.1-586.8)	289.5 (265.2-314.4)
	Cardiovascular diseases	9212.5 (8564.7-9901.0)	4496.9 (4130.5-4875.6)	443.8 (411.0-479.5)	218.2 (199.4-238.2)
	Diabetes mellitus type 2	1576.2 (1173.0-2018.0)	1228.7 (916.0-1595.1)	44.8 (33.8-57.6)	30.3 (22.8-38.7)
	Neoplasms	994.2 (841.6-1149.9)	669.6 (562.3-780.4)	46.3 (38.4-54.0)	31.0 (26.0-36.3)
Bulgaria	All causes	13768.8 (13022.8-14532.0)	9101.7 (8422.0-9791.5)	741.7 (697.8-787.5)	453.0 (416.5-490.1)
	Cardiovascular diseases	12412.7 (11720.0-13126.6)	7981.6 (7365.5-8613.3)	699.2 (654.9-744.6)	421.7 (386.5-456.8)
	Diabetes mellitus type 2	454.2 (330.2-605.4)	471.3 (338.9-630.8)	4.9 (3.8-6.1)	3.9 (3.0-4.8)
	Neoplasms	808.3 (691.3-922.1)	594.2 (496.0-706.6)	34.3 (28.9-39.4)	25.2 (21.1-29.6)
Burkina Faso	All causes	5928.1 (4988.3-6745.8)	6532.3 (5769.6-7377.5)	270.2 (227.4-309.3)	304.1 (268.5-343.6)
	Cardiovascular diseases	4997.6 (4174.1-5730.7)	5418.2 (4746.0-6146.4)	238.4 (199.8-274.9)	267.9 (236.0-302.6)

	Diabetes mellitus type 2	537.0 (381.9-715.7)	712.2 (495.3-951.3)	12.8 (8.6-17.7)	16.4 (11.2-22.1)
	Neoplasms	350.6 (265.9-434.7)	346.2 (272.5-425.0)	17.1 (13.1-20.9)	17.0 (13.5-20.8)
Burundi	All causes	12546.7 (10861.6-14647.5)	7156.6 (6143.3-8143.5)	561.0 (487.0-652.7)	333.9 (285.1-383.1)
	Cardiovascular diseases	10921.7 (9384.8-12845.6)	5957.3 (5067.3-6871.5)	505.8 (436.9-592.4)	296.6 (251.9-342.8)
	Diabetes mellitus type 2	908.4 (648.5-1202.4)	824.5 (580.2-1102.4)	23.9 (16.7-31.7)	19.3 (13.3-25.6)
	Neoplasms	555.6 (397.0-716.3)	317.3 (234.1-401.6)	23.6 (17.0-30.4)	14.8 (11.0-18.6)
Cambodia	All causes	12180.4 (10979.9-13368.0)	6673.7 (5919.3-7482.9)	535.8 (484.9-590.9)	301.9 (268.9-337.3)
	Cardiovascular diseases	10503.1 (9450.4-11568.2)	5496.0 (4870.3-6153.3)	479.8 (433.5-529.4)	265.7 (236.1-297.5)
	Diabetes mellitus type 2	537.0 (349.0-758.2)	581.8 (362.9-827.5)	7.8 (4.9-11.1)	8.2 (5.2-11.7)
	Neoplasms	910.6 (706.2-1124.1)	501.5 (402.0-601.9)	39.1 (30.4-48.3)	23.7 (19.0-28.5)
Cameroon	All causes	5017.7 (4417.0-5664.7)	4986.5 (4195.0-5857.2)	241.8 (212.1-273.2)	227.6 (192.5-265.7)
	Cardiovascular diseases	4322.4 (3779.8-4928.4)	4085.2 (3402.0-4838.7)	219.2 (190.9-249.2)	198.3 (166.4-232.8)
	Diabetes mellitus type 2	409.8 (279.6-562.2)	600.4 (406.4-822.5)	9.4 (6.3-13.3)	14.8 (9.9-20.6)
	Neoplasms	224.8 (171.5-294.4)	245.3 (183.9-312.9)	10.5 (8.0-13.9)	11.9 (8.9-15.2)
Canada	All causes	5457.5 (5115.1-5830.9)	2625.2 (2400.6-2882.0)	279.0 (261.0-296.9)	127.2 (117.3-137.2)
	Cardiovascular diseases	4442.9 (4177.0-4706.8)	1839.8 (1689.6-1992.6)	246.9 (229.6-263.3)	102.7 (94.0-111.8)
	Diabetes mellitus type 2	472.4 (347.0-621.7)	418.0 (305.9-550.0)	6.0 (4.8-7.3)	5.5 (4.3-6.7)
	Neoplasms	513.8 (425.4-615.2)	330.5 (266.5-403.5)	24.7 (20.6-29.4)	16.9 (13.7-20.6)
Cape Verde	All causes	4987.8 (4476.1-5510.7)	4043.9 (3620.8-4516.8)	239.3 (213.0-264.5)	187.2 (167.0-208.4)
	Cardiovascular diseases	4362.8 (3892.7-4832.1)	3200.9 (2872.2-3564.0)	221.8 (196.7-247.7)	163.1 (145.1-182.7)
	Diabetes mellitus type 2	320.8 (217.4-441.7)	511.7 (351.8-707.1)	4.1 (2.9-5.5)	8.2 (5.9-11.0)
	Neoplasms	283.6 (187.1-434.2)	295.4 (216.5-376.0)	12.8 (8.2-20.2)	14.4 (10.6-18.9)
Central African Republic	All causes	11454.0 (9786.0-13221.2)	11063.8 (9367.7-12749.1)	505.2 (437.1-572.0)	475.8 (400.9-550.9)
	Cardiovascular diseases	10010.3 (8489.2-11628.5)	9444.9 (7878.9-11019.8)	465.5 (400.5-530.5)	434.2 (364.1-503.6)
	Diabetes mellitus type 2	969.3 (666.8-1319.6)	1169.5 (810.9-1574.3)	19.5 (13.2-26.6)	21.9 (14.7-29.5)
	Neoplasms	434.0 (306.7-573.7)	392.3 (281.4-526.8)	18.3 (13.4-23.9)	16.7 (12.2-21.9)
Chad	All causes	6376.9 (5567.4-7212.7)	6995.4 (6131.3-8018.6)	302.3 (263.8-343.3)	319.3 (280.6-366.1)
	Cardiovascular diseases	5618.3 (4882.4-6387.6)	5931.0 (5150.6-6862.1)	280.7 (243.1-319.9)	288.1 (251.8-332.0)

	Diabetes mellitus type 2	479.5 (338.3-642.0)	701.0 (492.0-938.9)	8.9 (6.2-11.9)	13.9 (9.6-19.1)
	Neoplasms	238.6 (179.6-311.0)	315.8 (237.6-403.6)	11.1 (8.4-14.5)	15.2 (11.5-19.5)
Chile	All causes	6405.5 (5954.5-6887.4)	3458.2 (3086.8-3868.0)	328.0 (304.1-352.5)	158.3 (138.9-180.1)
	Cardiovascular diseases	5106.2 (4806.2-5401.6)	2309.7 (2077.2-2554.4)	282.2 (263.7-300.2)	119.8 (106.5-134.1)
	Diabetes mellitus type 2	574.6 (423.6-749.5)	624.7 (467.4-811.9)	10.2 (8.0-12.4)	10.1 (8.1-12.4)
	Neoplasms	655.0 (433.2-893.4)	439.7 (330.7-556.8)	32.4 (21.4-44.5)	23.5 (17.7-30.1)
China	All causes	9602.7 (8912.1-10322.9)	7054.0 (6521.2-7547.9)	454.5 (415.2-494.7)	350.2 (323.8-376.6)
	Cardiovascular diseases	7567.3 (6976.0-8146.0)	5642.8 (5224.5-6055.6)	380.0 (344.6-415.1)	299.1 (275.3-324.0)
	Diabetes mellitus type 2	326.1 (219.1-449.2)	385.8 (270.5-524.0)	2.5 (1.8-3.4)	4.0 (3.0-5.0)
	Neoplasms	1537.6 (1280.2-1787.4)	888.9 (744.2-1036.4)	66.0 (54.6-77.7)	41.7 (34.2-49.3)
Colombia	All causes	6487.2 (6057.7-6957.7)	3065.9 (2752.2-3387.9)	312.8 (288.7-338.0)	143.1 (127.9-159.2)
	Cardiovascular diseases	5500.1 (5152.1-5878.3)	2356.1 (2104.2-2611.0)	279.4 (258.8-299.7)	121.9 (108.6-135.6)
	Diabetes mellitus type 2	458.0 (321.4-618.8)	372.4 (259.4-504.4)	8.1 (5.9-10.5)	5.6 (4.0-7.3)
	Neoplasms	440.2 (294.3-609.8)	264.1 (202.0-333.1)	21.2 (14.1-29.6)	12.3 (9.5-15.5)
Comoros	All causes	11134.9 (9698.1-12747.5)	6684.3 (5779.4-7639.4)	501.8 (432.8-580.8)	310.8 (266.2-357.7)
	Cardiovascular diseases	9642.9 (8349.5-11091.9)	5463.9 (4685.6-6320.0)	451.3 (389.6-520.2)	273.5 (233.7-315.9)
	Diabetes mellitus type 2	780.0 (554.3-1040.3)	796.1 (554.4-1079.9)	18.7 (12.9-25.5)	16.7 (11.1-23.3)
	Neoplasms	555.7 (420.9-709.6)	357.8 (276.4-450.8)	24.2 (18.4-30.7)	16.9 (13.1-21.1)
Congo	All causes	11906.7 (10524.7-13463.5)	8211.5 (7096.7-9294.5)	530.2 (472.4-592.8)	379.5 (326.2-432.4)
	Cardiovascular diseases	10269.9 (8993.8-11692.8)	6580.7 (5697.1-7491.0)	482.6 (429.2-542.1)	333.7 (286.8-381.9)
	Diabetes mellitus type 2	1084.0 (762.6-1445.5)	1167.0 (808.0-1584.7)	23.4 (16.7-31.3)	23.2 (16.3-31.0)
	Neoplasms	496.5 (369.1-648.7)	384.5 (295.9-492.0)	21.3 (16.4-27.1)	17.9 (14.1-22.2)
Costa Rica	All causes	5158.3 (4781.0-5570.4)	3304.0 (2988.7-3653.3)	272.6 (250.3-297.3)	153.2 (136.9-171.1)
	Cardiovascular diseases	4236.3 (3960.4-4513.0)	2488.7 (2259.1-2724.7)	240.7 (223.0-257.1)	128.3 (115.7-141.8)
	Diabetes mellitus type 2	454.2 (321.2-612.2)	411.8 (287.9-564.9)	7.3 (5.4-9.5)	4.6 (3.4-6.1)
	Neoplasms	411.8 (229.6-667.1)	326.4 (243.2-435.6)	21.6 (11.7-34.8)	16.3 (12.0-21.8)
Cote d'Ivoire	All causes	8436.9 (7534.4-9346.5)	7572.9 (6612.0-8616.7)	392.4 (349.3-434.3)	343.2 (299.2-390.2)
	Cardiovascular diseases	7689.4 (6829.1-8517.2)	6679.6 (5785.0-7620.0)	369.9 (330.0-409.3)	317.8 (276.5-361.0)

	Diabetes mellitus type 2	451.8 (310.8-609.2)	626.3 (417.5-856.6)	9.2 (6.3-12.4)	13.0 (8.8-17.9)
	Neoplasms	167.3 (131.1-208.4)	155.3 (121.8-191.9)	7.9 (6.2-10.0)	7.5 (5.8-9.3)
Croatia	All causes	11471.7 (10869.1-12112.5)	5258.2 (4821.8-5709.8)	612.9 (576.3-648.9)	285.0 (262.3-309.8)
	Cardiovascular diseases	9854.0 (9301.5-10400.9)	4120.4 (3806.6-4454.2)	557.7 (522.7-592.1)	252.5 (231.4-275.3)
	Diabetes mellitus type 2	514.9 (360.0-709.7)	581.2 (400.9-798.2)	5.8 (4.3-7.5)	5.2 (3.9-6.7)
	Neoplasms	1014.3 (857.7-1166.6)	516.3 (417.6-627.1)	45.9 (38.6-52.9)	25.2 (20.5-30.3)
Cuba	All causes	7073.0 (6434.7-7789.4)	3570.7 (3072.6-4162.7)	364.3 (332.6-396.7)	171.8 (148.5-197.8)
	Cardiovascular diseases	6078.3 (5554.7-6641.0)	2870.4 (2461.7-3314.0)	333.8 (303.7-364.7)	154.3 (133.1-177.6)
	Diabetes mellitus type 2	581.6 (408.9-776.5)	444.2 (299.8-619.9)	9.8 (7.2-12.7)	4.5 (3.2-5.9)
	Neoplasms	391.0 (310.1-482.9)	237.4 (171.1-328.9)	19.7 (15.8-24.0)	12.1 (8.8-16.5)
Cyprus	All causes	5645.2 (5145.1-6201.0)	3284.6 (2957.0-3651.9)	288.8 (261.3-317.8)	156.9 (141.3-174.7)
	Cardiovascular diseases	4721.7 (4287.3-5202.4)	2431.0 (2181.9-2689.3)	254.3 (228.5-281.3)	129.7 (115.8-145.0)
	Diabetes mellitus type 2	607.8 (436.6-798.4)	537.1 (386.0-719.7)	18.8 (13.6-24.6)	11.1 (8.3-14.4)
	Neoplasms	282.0 (235.2-334.9)	285.0 (231.4-343.4)	13.6 (11.4-16.1)	13.9 (11.3-16.7)
Czech Republic	All causes	14078.8 (13344.3-14802.6)	5086.2 (4664.8-5549.3)	736.4 (696.7-775.0)	273.5 (251.9-295.8)
	Cardiovascular diseases	12358.9 (11726.6-12978.6)	3969.0 (3661.0-4301.9)	674.8 (636.7-712.3)	243.1 (223.8-263.3)
	Diabetes mellitus type 2	498.8 (357.1-666.1)	609.9 (435.5-828.4)	6.4 (4.8-8.1)	5.9 (4.5-7.6)
	Neoplasms	1106.6 (925.6-1298.7)	473.7 (377.9-577.5)	50.9 (42.6-59.3)	23.0 (18.3-27.8)
Democratic Republic of the Congo	All causes	7848.7 (6695.4-9105.1)	7244.8 (6133.6-8481.4)	361.4 (308.2-419.4)	325.6 (275.9-379.8)
	Cardiovascular diseases	6669.1 (5676.6-7782.8)	5958.1 (5044.0-6941.0)	329.0 (279.5-382.7)	294.5 (249.1-344.7)
	Diabetes mellitus type 2	848.3 (582.4-1151.3)	989.1 (683.0-1348.7)	17.4 (11.8-24.0)	17.4 (11.9-24.3)
	Neoplasms	310.7 (223.8-411.5)	280.5 (207.8-371.1)	14.1 (10.3-18.7)	12.8 (9.6-16.6)
Denmark	All causes	6663.1 (6146.0-7243.0)	2416.0 (2140.2-2743.1)	355.6 (326.7-386.2)	114.5 (105.0-125.2)
	Cardiovascular diseases	5712.2 (5267.6-6166.7)	1518.8 (1382.1-1656.3)	327.9 (299.8-355.4)	92.1 (83.6-100.5)
	Diabetes mellitus type 2	429.3 (302.9-585.4)	575.9 (406.7-781.9)	3.7 (2.8-4.7)	5.3 (4.1-6.8)
	Neoplasms	510.4 (397.4-636.5)	303.7 (220.0-405.9)	23.6 (18.5-29.1)	16.0 (11.7-21.0)
Djibouti	All causes	9118.5 (7452.9-11052.9)	6980.8 (5562.3-8636.9)	423.3 (346.0-515.0)	319.0 (253.5-393.2)
	Cardiovascular diseases	7628.2 (6191.3-9358.4)	5541.7 (4313.4-6893.1)	370.4 (303.7-450.2)	271.7 (214.5-334.1)

	Diabetes mellitus type 2	752.7 (527.5-1009.9)	937.7 (667.7-1252.1)	19.2 (12.2-27.7)	22.7 (15.0-31.9)
	Neoplasms	595.8 (425.9-804.1)	419.8 (302.3-571.4)	26.6 (19.2-35.4)	19.8 (14.6-26.2)
Dominica	All causes	6033.0 (5531.3-6591.8)	4353.6 (3900.6-4880.2)	295.2 (270.0-322.2)	194.8 (175.7-215.8)
	Cardiovascular diseases	4576.2 (4181.3-4967.8)	2883.6 (2602.9-3205.5)	249.7 (226.9-272.4)	151.6 (136.2-169.2)
	Diabetes mellitus type 2	1070.7 (759.1-1403.9)	1131.5 (808.5-1501.4)	25.1 (18.4-32.3)	25.9 (18.9-33.9)
	Neoplasms	328.1 (242.9-454.4)	290.1 (235.8-357.5)	17.2 (12.3-24.2)	14.6 (11.7-18.4)
Dominican Republic	All causes	5489.6 (4931.5-6121.1)	5760.3 (4969.6-6711.2)	250.1 (224.1-275.2)	256.4 (220.3-295.8)
	Cardiovascular diseases	4849.6 (4333.8-5405.2)	4908.4 (4160.9-5738.2)	233.4 (208.0-257.7)	234.4 (200.3-271.2)
	Diabetes mellitus type 2	455.6 (311.8-618.3)	646.4 (456.6-867.7)	7.3 (5.3-9.8)	12.1 (8.5-16.2)
	Neoplasms	155.1 (129.3-182.6)	168.1 (136.1-205.0)	7.9 (6.6-9.5)	8.1 (6.5-9.8)
Ecuador	All causes	5057.6 (4646.2-5532.0)	3617.2 (3214.8-4065.3)	239.1 (216.6-265.5)	167.2 (145.9-191.8)
	Cardiovascular diseases	4104.2 (3822.9-4391.3)	2454.7 (2209.5-2716.9)	207.1 (191.4-223.1)	128.1 (114.1-143.4)
	Diabetes mellitus type 2	517.2 (370.6-680.5)	773.9 (570.0-999.1)	9.5 (6.9-12.3)	16.7 (12.3-21.6)
	Neoplasms	363.8 (164.3-598.4)	269.9 (176.2-390.9)	18.8 (8.5-30.9)	15.0 (9.5-22.0)
Egypt	All causes	14560.3 (13550.5-15621.8)	11836.7 (10524.9-13268.2)	694.4 (643.9-749.6)	551.8 (490.4-620.3)
	Cardiovascular diseases	13815.0 (12814.8-14854.2)	10810.8 (9577.4-12209.0)	679.7 (627.9-735.5)	531.9 (470.8-598.0)
	Diabetes mellitus type 2	552.3 (378.8-766.6)	841.8 (575.3-1146.3)	6.1 (4.2-8.4)	11.4 (8.0-15.1)
	Neoplasms	139.0 (114.9-165.8)	119.5 (96.5-146.5)	6.0 (4.9-7.3)	5.3 (4.3-6.4)
El Salvador	All causes	6235.6 (5795.5-6697.1)	4742.7 (4139.9-5411.9)	285.2 (264.9-304.9)	214.1 (185.4-245.3)
	Cardiovascular diseases	5413.2 (5026.7-5799.6)	3309.6 (2866.7-3797.1)	264.2 (243.9-283.9)	171.0 (148.1-195.3)
	Diabetes mellitus type 2	554.0 (379.2-764.6)	1001.6 (721.0-1328.7)	7.8 (5.7-10.2)	22.3 (15.9-29.5)
	Neoplasms	208.1 (142.7-303.0)	246.3 (181.3-340.9)	10.3 (6.8-15.1)	11.9 (8.6-16.6)
Equatorial Guinea	All causes	12873.7 (10725.4-15299.1)	4162.4 (3239.2-5229.6)	569.9 (478.4-663.2)	190.6 (143.1-248.6)
	Cardiovascular diseases	11179.7 (9167.9-13391.6)	2773.9 (2051.4-3653.3)	521.8 (435.4-609.1)	154.2 (115.2-201.9)
	Diabetes mellitus type 2	1159.6 (795.7-1540.9)	1087.8 (760.3-1463.0)	25.0 (17.2-33.7)	20.1 (13.4-28.1)
	Neoplasms	487.7 (333.4-661.6)	195.4 (133.7-276.7)	20.7 (14.6-27.5)	9.6 (6.8-13.1)
Eritrea	All causes	13189.1 (11073.0-15137.8)	7645.7 (6585.3-8677.7)	562.9 (483.5-650.0)	345.2 (293.9-401.1)
	Cardiovascular diseases	11453.4 (9585.3-13240.3)	6278.7 (5357.5-7194.6)	504.3 (430.7-581.2)	301.4 (254.4-351.8)

	Diabetes mellitus type 2	860.6 (603.7-1171.5)	831.5 (579.0-1129.3)	22.1 (14.9-30.8)	19.3 (12.7-27.5)
	Neoplasms	714.2 (520.4-915.3)	459.2 (350.4-577.7)	29.2 (21.9-37.0)	20.3 (15.6-25.3)
Estonia	All causes	13325.3 (12588.3-14036.6)	4925.5 (4244.7-5707.8)	704.6 (662.3-744.8)	265.3 (223.9-318.1)
	Cardiovascular diseases	12114.4 (11457.5-12769.2)	4005.6 (3409.1-4748.9)	670.0 (628.8-709.2)	243.4 (203.8-293.7)
	Diabetes mellitus type 2	444.6 (311.0-599.5)	510.4 (353.5-700.7)	2.1 (1.6-2.6)	2.3 (1.7-3.0)
	Neoplasms	741.0 (549.5-935.3)	375.6 (296.1-460.4)	31.7 (23.6-40.2)	17.8 (14.1-21.6)
Ethiopia	All causes	11169.6 (9705.9-12774.9)	4633.2 (4043.4-5242.3)	498.7 (436.8-563.0)	216.8 (184.6-249.2)
	Cardiovascular diseases	9470.9 (8163.0-10970.2)	3663.1 (3165.2-4173.3)	437.7 (380.7-497.9)	184.0 (155.9-213.5)
	Diabetes mellitus type 2	938.3 (661.3-1252.8)	617.4 (435.8-824.9)	25.5 (17.2-34.6)	14.9 (9.8-20.9)
	Neoplasms	581.0 (429.3-727.2)	295.2 (235.2-355.0)	26.7 (20.4-32.9)	14.7 (11.7-17.7)
Federated States of Micronesia	All causes	15559.9 (13771.4-17421.5)	14892.0 (12879.0-16841.3)	646.4 (575.5-719.9)	610.2 (545.2-678.6)
	Cardiovascular diseases	13274.5 (11706.1-15011.3)	11348.0 (9753.0-12961.3)	580.1 (513.1-650.5)	506.9 (451.2-565.9)
	Diabetes mellitus type 2	1606.5 (1130.9-2132.5)	2848.4 (2053.1-3765.9)	31.1 (22.0-40.8)	66.9 (48.4-88.2)
	Neoplasms	496.8 (389.4-606.6)	440.8 (353.1-538.4)	24.7 (19.2-30.0)	21.9 (17.7-26.5)
Fiji	All causes	14616.7 (13180.9-16174.1)	15546.5 (13592.6-17575.9)	610.5 (546.4-673.6)	636.2 (562.1-713.1)
	Cardiovascular diseases	11408.1 (10235.2-12586.0)	9896.4 (8796.4-11036.6)	511.5 (457.4-564.3)	448.8 (399.7-501.2)
	Diabetes mellitus type 2	2765.6 (2066.8-3555.9)	5150.0 (3779.2-6653.0)	75.3 (55.7-97.1)	160.9 (116.9-208.9)
	Neoplasms	276.2 (230.3-324.2)	267.9 (221.7-313.5)	14.1 (11.7-16.6)	13.6 (11.2-16.0)
Finland	All causes	7630.6 (7121.8-8141.6)	3129.0 (2831.9-3448.8)	394.7 (365.6-422.5)	160.1 (146.0-175.1)
	Cardiovascular diseases	6883.1 (6419.6-7359.7)	2420.8 (2212.6-2649.8)	373.2 (345.0-399.9)	146.6 (133.1-160.8)
	Diabetes mellitus type 2	376.1 (268.8-509.5)	470.7 (324.4-651.8)	3.5 (2.7-4.5)	1.6 (1.2-2.0)
	Neoplasms	360.2 (285.2-436.5)	224.3 (177.3-277.2)	17.6 (13.8-21.3)	11.3 (8.9-13.9)
France	All causes	3604.6 (3351.3-3882.3)	1887.1 (1707.7-2087.0)	191.3 (177.6-206.0)	89.1 (81.7-97.1)
	Cardiovascular diseases	2871.0 (2663.8-3072.8)	1261.8 (1148.2-1375.4)	163.4 (150.7-176.5)	68.9 (62.5-75.6)
	Diabetes mellitus type 2	199.6 (142.2-267.0)	271.9 (190.1-367.5)	2.6 (2.0-3.5)	3.0 (2.3-3.8)
	Neoplasms	519.1 (417.2-633.8)	338.8 (258.1-428.9)	24.5 (20.0-29.3)	16.3 (12.7-20.4)
Gabon	All causes	7010.1 (6083.1-7936.1)	5639.6 (4904.6-6410.4)	326.1 (283.5-371.5)	261.2 (226.2-299.6)
	Cardiovascular diseases	5870.8 (5061.6-6663.2)	4295.4 (3715.6-4949.2)	294.0 (254.8-334.5)	224.1 (192.7-259.5)

	Diabetes mellitus type 2	822.0 (568.7-1139.0)	997.2 (684.0-1377.8)	17.1 (11.7-24.5)	19.3 (13.3-26.6)
	Neoplasms	253.2 (197.2-319.2)	251.3 (194.8-322.7)	11.6 (9.2-14.4)	11.8 (9.3-14.9)
Georgia	All causes	14566.1 (13717.1-15483.7)	11020.8 (10307.7-11717.8)	728.2 (682.2-775.6)	564.4 (522.2-610.0)
	Cardiovascular diseases	13478.1 (12673.4-14314.4)	9578.8 (8954.8-10203.6)	703.7 (657.6-750.5)	531.3 (491.9-573.1)
	Diabetes mellitus type 2	482.6 (323.6-675.3)	866.6 (604.9-1184.7)	1.7 (1.3-2.3)	8.6 (6.4-11.2)
	Neoplasms	543.3 (436.1-655.6)	507.6 (396.2-613.0)	21.1 (16.7-25.6)	21.5 (16.7-26.0)
Germany	All causes	6465.8 (6009.3-6936.3)	3024.6 (2722.7-3370.5)	355.3 (327.8-381.7)	162.0 (145.6-180.8)
	Cardiovascular diseases	5592.5 (5181.0-6003.2)	2268.4 (2026.1-2523.7)	322.9 (297.2-347.8)	136.9 (121.8-153.7)
	Diabetes mellitus type 2	316.5 (232.6-414.8)	349.6 (251.8-466.1)	6.1 (4.7-7.7)	5.0 (3.9-6.2)
	Neoplasms	534.9 (452.6-623.7)	380.4 (298.9-477.5)	25.4 (21.4-29.5)	18.2 (14.4-22.6)
Ghana	All causes	6711.2 (5842.7-7650.1)	6777.4 (5959.0-7674.6)	316.1 (276.0-360.4)	314.1 (274.1-358.8)
	Cardiovascular diseases	5975.3 (5183.8-6843.1)	5767.4 (5037.8-6577.7)	294.3 (256.3-335.4)	284.9 (248.1-325.3)
	Diabetes mellitus type 2	494.8 (347.6-667.9)	721.9 (497.8-975.0)	10.7 (7.5-14.2)	16.2 (11.3-21.8)
	Neoplasms	202.4 (155.6-262.2)	173.1 (129.2-224.7)	9.7 (7.5-12.6)	8.7 (6.3-11.6)
Greece	All causes	4693.3 (4305.3-5083.0)	3397.5 (3065.6-3741.0)	247.8 (225.3-269.0)	166.5 (150.2-182.7)
	Cardiovascular diseases	4138.0 (3785.9-4490.8)	2757.9 (2494.9-3029.5)	230.9 (208.8-251.4)	150.1 (135.1-165.1)
	Diabetes mellitus type 2	279.9 (193.6-388.3)	359.6 (247.9-503.6)	2.9 (2.2-3.8)	2.2 (1.7-2.9)
	Neoplasms	242.7 (191.1-295.3)	252.1 (200.4-314.9)	12.0 (9.5-14.7)	12.5 (9.9-15.5)
Greenland	All causes	8899.8 (8189.7-9681.6)	4594.9 (4187.9-5004.2)	404.9 (371.5-439.7)	211.3 (191.3-231.5)
	Cardiovascular diseases	7222.9 (6629.1-7819.2)	3462.8 (3158.2-3786.5)	347.8 (317.4-378.0)	171.2 (154.9-189.7)
	Diabetes mellitus type 2	613.8 (457.4-792.5)	414.0 (305.8-533.1)	8.0 (6.4-9.8)	5.3 (4.2-6.4)
	Neoplasms	1039.5 (823.5-1290.3)	680.9 (525.9-846.2)	48.2 (38.1-60.4)	33.3 (25.7-41.5)
Grenada	All causes	9870.3 (9067.8-10745.2)	5702.0 (5082.9-6381.2)	464.3 (429.8-500.3)	244.4 (220.7-270.0)
	Cardiovascular diseases	8173.2 (7536.2-8893.4)	4045.4 (3651.1-4484.7)	411.5 (379.5-446.5)	196.3 (176.4-217.1)
	Diabetes mellitus type 2	1261.2 (908.5-1645.8)	1324.7 (953.5-1732.6)	31.2 (22.9-40.2)	32.1 (23.5-41.7)
	Neoplasms	372.4 (308.7-437.3)	285.2 (233.7-350.2)	18.1 (14.9-21.6)	13.5 (11.2-16.1)
Guam	All causes	8473.6 (7697.5-9259.9)	9565.7 (8727.9-10421.5)	384.9 (348.2-424.2)	394.4 (361.1-429.8)
	Cardiovascular diseases	7102.4 (6445.8-7800.1)	7883.8 (7196.1-8535.9)	346.1 (310.4-383.4)	353.1 (321.9-384.9)

	Diabetes mellitus type 2	923.1 (660.7-1220.8)	1175.4 (840.4-1568.7)	15.4 (11.4-19.9)	16.0 (11.9-20.6)
	Neoplasms	321.6 (249.1-413.2)	284.3 (219.0-366.6)	16.4 (12.7-20.6)	13.5 (10.4-17.2)
Guatemala	All causes	5290.2 (4818.7-5851.3)	4344.8 (3846.4-4877.1)	251.6 (228.2-275.1)	193.6 (173.7-215.9)
	Cardiovascular diseases	4595.2 (4149.4-5088.3)	2780.5 (2464.3-3095.5)	234.8 (210.4-261.2)	149.3 (132.1-166.4)
	Diabetes mellitus type 2	498.9 (338.9-689.4)	1274.3 (907.3-1672.5)	5.9 (4.2-7.8)	29.0 (21.0-38.2)
	Neoplasms	169.7 (121.4-269.0)	211.5 (144.0-331.2)	9.1 (6.1-15.1)	10.9 (7.1-17.5)
Guinea	All causes	5685.4 (4990.6-6444.1)	7452.3 (6470.7-8503.3)	272.0 (236.7-308.3)	340.8 (296.0-387.8)
	Cardiovascular diseases	5125.3 (4484.4-5856.1)	6607.4 (5689.2-7573.9)	255.2 (221.9-291.3)	315.3 (273.0-360.0)
	Diabetes mellitus type 2	333.2 (224.0-457.5)	560.3 (379.5-763.3)	6.7 (4.5-9.2)	12.6 (8.8-17.3)
	Neoplasms	181.8 (136.1-260.1)	234.6 (173.1-320.4)	8.3 (6.3-11.8)	10.7 (8.1-14.6)
Guinea-Bissau	All causes	10490.4 (9022.3-12097.5)	9538.3 (8271.4-10923.3)	469.6 (408.0-534.6)	415.9 (364.8-473.5)
	Cardiovascular diseases	9490.2 (8135.5-10997.0)	8351.4 (7234.5-9576.8)	437.1 (377.3-499.0)	379.4 (330.8-430.5)
	Diabetes mellitus type 2	513.5 (341.3-705.4)	749.7 (504.3-1024.4)	11.0 (7.3-15.3)	16.5 (11.1-22.6)
	Neoplasms	415.3 (281.1-571.2)	373.5 (276.7-481.6)	18.5 (12.7-25.1)	17.2 (12.9-22.0)
Guyana	All causes	12603.4 (11587.2-13747.9)	8987.4 (7924.1-10162.9)	553.4 (511.4-597.4)	385.9 (343.1-435.3)
	Cardiovascular diseases	11172.7 (10299.1-12123.0)	7199.4 (6360.5-8081.2)	514.3 (473.4-557.2)	336.9 (298.0-379.2)
	Diabetes mellitus type 2	1133.7 (797.5-1506.6)	1528.1 (1102.7-1989.2)	24.8 (18.1-32.1)	36.9 (26.9-48.3)
	Neoplasms	257.4 (215.8-307.6)	221.1 (182.3-266.7)	12.3 (10.2-15.0)	10.2 (8.4-12.1)
Haiti	All causes	13760.0 (12137.0-15593.7)	9425.6 (7888.2-11069.7)	612.1 (543.8-685.6)	425.3 (358.8-495.1)
	Cardiovascular diseases	12090.4 (10602.5-13704.7)	7868.4 (6561.1-9324.7)	561.5 (497.8-630.6)	379.7 (320.0-443.6)
	Diabetes mellitus type 2	1256.1 (855.7-1699.3)	1202.7 (845.4-1602.8)	30.3 (20.6-41.8)	28.2 (19.3-38.1)
	Neoplasms	362.7 (255.0-498.8)	324.4 (240.6-421.0)	17.6 (12.3-24.6)	15.8 (12.0-20.5)
Honduras	All causes	6770.0 (6104.6-7515.5)	5825.6 (4980.8-6720.9)	282.5 (252.4-312.2)	271.1 (228.7-313.9)
	Cardiovascular diseases	6110.9 (5512.4-6802.4)	4979.7 (4213.9-5764.7)	268.8 (240.1-298.9)	252.0 (211.4-293.3)
	Diabetes mellitus type 2	455.7 (306.4-636.2)	620.3 (424.0-851.9)	4.4 (3.0-6.0)	8.3 (5.7-11.3)
	Neoplasms	173.9 (134.7-221.5)	191.4 (151.1-237.7)	8.1 (6.2-10.7)	9.3 (7.3-11.5)
Hungary	All causes	14881.2 (14185.0-15619.4)	6634.4 (6156.1-7167.9)	736.8 (700.7-776.2)	330.9 (306.6-357.9)
	Cardiovascular diseases	12956.5 (12346.3-13618.3)	5253.7 (4880.0-5663.3)	669.6 (632.4-707.2)	289.3 (266.7-313.7)

	Diabetes mellitus type 2	552.9 (396.3-733.8)	576.0 (412.3-770.0)	5.5 (4.2-6.8)	5.3 (4.1-6.6)
	Neoplasms	1263.0 (1061.0-1470.9)	762.1 (606.8-930.3)	57.8 (48.5-66.9)	34.3 (27.7-41.6)
Iceland	All causes	5578.9 (5184.1-6000.1)	2431.0 (2189.3-2715.5)	299.2 (276.3-321.8)	123.9 (113.0-134.8)
	Cardiovascular diseases	4845.6 (4487.5-5201.6)	1771.5 (1620.3-1920.9)	276.5 (253.7-298.8)	109.6 (99.2-119.0)
	Diabetes mellitus type 2	316.6 (225.4-427.1)	421.8 (289.7-579.3)	2.6 (2.0-3.3)	2.0 (1.6-2.6)
	Neoplasms	406.0 (320.9-493.3)	226.9 (175.8-292.4)	19.5 (15.5-23.7)	11.6 (9.0-14.8)
India	All causes	8581.9 (7924.1-9221.5)	7148.9 (6602.7-7769.3)	371.1 (340.6-400.4)	309.9 (282.2-339.9)
	Cardiovascular diseases	7735.6 (7139.0-8313.8)	6234.6 (5743.8-6779.5)	345.9 (316.3-375.0)	283.7 (256.6-313.0)
	Diabetes mellitus type 2	435.4 (300.6-593.9)	580.0 (405.0-790.6)	8.1 (5.7-10.9)	12.2 (8.8-16.0)
	Neoplasms	336.2 (262.7-414.5)	272.5 (225.6-317.9)	14.1 (11.1-17.3)	11.8 (9.9-13.7)
Indonesia	All causes	10218.6 (9512.8-11008.5)	9124.8 (8420.2-9898.3)	441.8 (407.9-481.7)	409.7 (377.6-444.2)
	Cardiovascular diseases	8840.0 (8219.6-9516.6)	7785.5 (7201.4-8430.2)	398.4 (366.7-435.1)	370.4 (340.2-403.0)
	Diabetes mellitus type 2	649.1 (436.2-885.9)	891.6 (594.2-1224.2)	12.6 (8.7-17.2)	17.8 (12.1-24.3)
	Neoplasms	504.3 (415.6-600.0)	325.5 (270.5-381.6)	22.1 (17.9-26.4)	16.1 (13.3-19.0)
Iran	All causes	9061.7 (8439.1-9657.7)	4930.1 (4467.7-5364.3)	452.0 (417.4-484.7)	251.0 (226.9-275.5)
	Cardiovascular diseases	8306.4 (7677.5-8899.6)	4032.9 (3656.6-4394.6)	429.9 (396.1-463.8)	226.9 (203.4-249.4)
	Diabetes mellitus type 2	420.6 (288.5-572.3)	625.8 (420.4-865.5)	6.4 (4.6-8.5)	10.6 (7.4-14.3)
	Neoplasms	297.8 (197.3-445.3)	229.6 (173.2-308.9)	13.9 (9.1-20.9)	11.4 (8.5-15.9)
Iraq	All causes	14783.3 (13136.9-16591.4)	5858.1 (5343.8-6420.3)	680.5 (605.1-759.3)	251.1 (229.2-272.7)
	Cardiovascular diseases	13089.9 (11587.1-14741.9)	4780.7 (4360.4-5203.2)	633.9 (562.6-707.9)	229.7 (209.0-251.0)
	Diabetes mellitus type 2	1293.7 (926.5-1721.5)	865.3 (606.0-1166.3)	28.3 (19.8-37.6)	11.7 (8.5-15.0)
	Neoplasms	270.8 (212.0-334.1)	154.3 (125.4-184.8)	11.8 (9.3-14.4)	7.1 (5.8-8.6)
Ireland	All causes	7421.7 (6830.7-8070.3)	2560.6 (2289.4-2839.0)	389.9 (358.1-423.7)	127.8 (116.6-139.4)
	Cardiovascular diseases	6456.1 (5925.1-7007.5)	1794.7 (1628.3-1960.3)	356.8 (325.9-388.9)	108.5 (98.7-118.6)
	Diabetes mellitus type 2	400.6 (285.5-538.9)	453.6 (310.4-626.2)	5.0 (3.8-6.3)	2.7 (2.1-3.4)
	Neoplasms	543.4 (434.8-661.3)	291.0 (225.9-368.6)	26.9 (21.7-32.5)	15.3 (11.9-19.2)
Israel	All causes	4705.7 (4221.4-5244.6)	1845.5 (1634.0-2078.9)	254.9 (227.5-284.7)	88.9 (80.0-97.7)
	Cardiovascular diseases	4051.7 (3598.2-4543.0)	1143.8 (1013.9-1271.0)	231.7 (205.0-261.5)	66.9 (59.4-74.3)

	Diabetes mellitus type 2	360.3 (250.7-491.9)	463.9 (330.0-622.2)	6.9 (5.0-9.0)	8.8 (6.4-11.5)
	Neoplasms	253.8 (213.1-300.5)	195.5 (157.7-240.5)	13.5 (11.3-16.0)	10.5 (8.4-12.9)
Italy	All causes	4317.8 (4001.5-4637.5)	2121.0 (1919.9-2345.4)	228.3 (210.1-246.0)	107.7 (97.6-118.5)
	Cardiovascular diseases	3425.4 (3156.9-3694.3)	1413.3 (1277.8-1550.7)	195.7 (178.9-211.5)	88.0 (79.1-98.0)
	Diabetes mellitus type 2	405.3 (296.0-525.6)	424.4 (299.8-572.0)	9.2 (7.0-11.6)	5.1 (3.8-6.5)
	Neoplasms	460.6 (377.7-545.1)	264.0 (219.0-312.5)	22.1 (17.8-27.0)	13.4 (11.0-16.0)
Jamaica	All causes	5393.7 (4877.8-5962.1)	5210.0 (4417.2-6131.9)	265.8 (242.6-292.8)	226.1 (195.4-260.4)
	Cardiovascular diseases	4137.5 (3782.3-4571.7)	3300.7 (2829.6-3827.0)	224.9 (204.7-248.0)	163.9 (141.3-188.3)
	Diabetes mellitus type 2	960.8 (680.9-1262.8)	1574.3 (1138.0-2090.6)	24.8 (17.8-32.6)	46.3 (32.9-62.1)
	Neoplasms	249.1 (201.7-310.1)	296.6 (234.4-382.5)	13.2 (10.5-16.8)	13.9 (11.1-17.6)
Japan	All causes	4699.0 (4357.7-5043.8)	2300.2 (2098.8-2512.8)	238.5 (219.7-258.3)	96.9 (88.5-105.9)
	Cardiovascular diseases	3342.6 (3123.8-3576.7)	1506.9 (1388.6-1639.2)	184.4 (170.8-199.7)	68.6 (62.9-74.6)
	Diabetes mellitus type 2	241.5 (166.9-333.2)	234.0 (161.1-321.0)	2.1 (1.6-2.7)	0.9 (0.7-1.1)
	Neoplasms	981.3 (815.2-1148.2)	496.5 (419.7-575.7)	45.8 (37.1-54.9)	24.9 (20.4-29.4)
Jordan	All causes	9587.4 (8555.2-10661.7)	4718.7 (4186.8-5291.1)	408.5 (364.6-452.1)	214.0 (189.8-243.1)
	Cardiovascular diseases	7962.4 (7052.2-8867.8)	3490.7 (3104.3-3935.1)	356.6 (315.5-396.4)	179.6 (157.8-204.2)
	Diabetes mellitus type 2	1248.8 (906.9-1630.0)	939.1 (663.8-1258.3)	36.0 (26.1-46.9)	20.0 (14.5-26.2)
	Neoplasms	297.0 (240.5-355.9)	229.3 (182.4-284.6)	12.4 (10.1-14.9)	11.3 (9.0-13.9)
Kazakhstan	All causes	13707.9 (12981.2-14440.4)	10335.7 (9519.2-11166.1)	643.3 (604.8-679.8)	529.0 (487.4-568.1)
	Cardiovascular diseases	11788.1 (11128.9-12425.0)	9198.2 (8444.3-9983.9)	586.4 (548.7-621.5)	500.8 (460.6-539.7)
	Diabetes mellitus type 2	560.2 (392.7-764.4)	607.6 (432.9-822.8)	3.4 (2.5-4.3)	4.9 (3.8-6.1)
	Neoplasms	1290.2 (1029.1-1545.3)	474.6 (375.1-581.2)	51.7 (40.8-62.5)	21.1 (16.9-25.6)
Kenya	All causes	5213.3 (4610.6-5921.3)	4971.4 (4373.0-5600.9)	244.1 (212.3-282.9)	225.4 (196.6-260.2)
	Cardiovascular diseases	4234.4 (3715.2-4836.5)	3957.8 (3493.6-4491.0)	212.7 (184.4-246.9)	195.9 (170.4-226.5)
	Diabetes mellitus type 2	607.2 (421.8-822.7)	718.6 (501.4-973.1)	14.2 (9.4-20.1)	15.2 (10.0-21.2)
	Neoplasms	296.9 (229.9-370.2)	243.8 (187.1-312.5)	13.5 (10.4-16.9)	11.5 (8.9-14.7)
Kiribati	All causes	17068.9 (15448.2-18683.3)	17679.1 (15373.4-19930.1)	647.3 (588.7-705.1)	642.8 (565.4-718.1)
	Cardiovascular diseases	13493.1 (12275.4-14678.5)	12321.0 (10856.1-13859.0)	542.9 (494.8-592.9)	488.6 (432.8-545.6)

	Diabetes mellitus type 2	2978.8 (2170.4-3808.9)	4756.1 (3529.2-6142.6)	74.7 (54.8-96.3)	124.9 (90.6-162.1)
	Neoplasms	457.2 (368.3-544.5)	431.0 (347.8-518.0)	22.1 (17.6-26.9)	20.3 (16.4-24.4)
Kuwait	All causes	6418.5 (5919.8-6944.8)	3780.9 (3396.3-4207.0)	269.9 (247.5-291.9)	138.3 (125.4-152.5)
	Cardiovascular diseases	5486.2 (5074.2-5907.3)	2993.1 (2706.5-3295.3)	246.1 (224.5-268.5)	124.9 (112.6-138.5)
	Diabetes mellitus type 2	710.2 (515.0-938.3)	641.5 (458.3-873.1)	13.6 (10.3-17.2)	6.6 (5.0-8.5)
	Neoplasms	155.3 (129.6-182.1)	112.8 (90.2-140.0)	7.1 (5.9-8.4)	5.6 (4.4-6.9)
Kyrgyzstan	All causes	13395.6 (12606.4-14222.5)	10453.2 (9702.5-11213.8)	625.1 (586.3-664.5)	539.4 (498.9-579.5)
	Cardiovascular diseases	11836.3 (11119.5-12567.4)	9565.4 (8863.3-10299.1)	583.3 (545.1-621.9)	520.4 (479.4-560.4)
	Diabetes mellitus type 2	517.6 (356.3-704.4)	515.7 (356.3-713.1)	2.8 (2.0-3.6)	3.2 (2.4-4.1)
	Neoplasms	976.2 (739.4-1226.7)	331.0 (228.8-443.9)	37.3 (28.2-46.9)	14.5 (10.3-19.4)
Laos	All causes	18710.4 (16486.3-20913.6)	9039.5 (7828.7-10266.7)	819.9 (725.2-914.4)	402.6 (353.4-455.8)
	Cardiovascular diseases	16868.6 (14811.7-19018.7)	7794.8 (6711.3-8866.0)	757.3 (666.8-844.6)	365.2 (319.1-413.0)
	Diabetes mellitus type 2	569.5 (362.2-808.0)	676.5 (430.4-958.9)	8.9 (5.4-13.2)	10.6 (7.0-15.1)
	Neoplasms	899.7 (699.0-1102.6)	420.6 (335.0-512.2)	38.3 (30.5-47.2)	19.9 (15.9-24.1)
Latvia	All causes	13287.6 (12618.3-13935.5)	7745.0 (6990.3-8541.9)	692.1 (655.3-727.5)	396.3 (357.4-436.2)
	Cardiovascular diseases	12098.6 (11497.5-12704.5)	6650.2 (5972.9-7381.8)	657.9 (623.0-691.9)	367.9 (330.2-405.6)
	Diabetes mellitus type 2	435.2 (312.5-583.6)	581.9 (415.2-772.4)	2.5 (1.9-3.1)	5.1 (4.0-6.5)
	Neoplasms	725.5 (547.1-905.9)	485.8 (393.1-579.4)	30.8 (23.4-38.4)	22.2 (18.2-26.5)
Lebanon	All causes	8132.8 (7048.6-9308.2)	5569.9 (4906.8-6372.6)	377.9 (323.8-430.4)	265.0 (231.8-303.6)
	Cardiovascular diseases	7074.3 (6049.5-8137.1)	4437.4 (3872.2-5053.0)	349.6 (296.5-400.1)	237.5 (205.3-273.1)
	Diabetes mellitus type 2	768.6 (513.5-1053.5)	837.1 (560.5-1143.4)	15.1 (10.1-20.9)	13.5 (9.2-18.4)
	Neoplasms	249.0 (196.6-309.0)	265.0 (207.8-337.0)	11.4 (9.0-14.1)	12.6 (9.9-15.7)
Lesotho	All causes	7338.4 (6476.3-8269.0)	9773.0 (8143.0-11497.2)	333.8 (292.9-374.9)	436.5 (360.9-521.0)
	Cardiovascular diseases	5795.2 (5057.9-6558.0)	7446.4 (6112.0-8850.9)	287.9 (252.2-326.5)	362.2 (298.9-431.3)
	Diabetes mellitus type 2	1067.3 (742.9-1447.8)	1747.9 (1194.2-2361.6)	25.7 (17.1-35.8)	49.5 (32.8-68.8)
	Neoplasms	427.4 (304.4-552.6)	503.8 (363.2-660.7)	18.2 (13.1-23.2)	21.5 (15.8-27.8)
Liberia	All causes	6802.8 (5988.0-7719.8)	6770.2 (5867.4-7790.5)	318.2 (277.7-362.1)	308.8 (266.8-353.4)
	Cardiovascular diseases	6042.0 (5275.1-6866.7)	5746.9 (4922.3-6630.3)	295.5 (257.2-336.8)	278.9 (240.5-319.9)

	Diabetes mellitus type 2	468.6 (314.0-639.6)	701.4 (485.6-949.1)	9.2 (6.3-12.6)	14.3 (10.0-19.5)
	Neoplasms	246.1 (188.0-312.5)	279.9 (208.4-355.0)	11.5 (8.8-14.6)	13.7 (10.3-17.4)
Libya	All causes	7988.5 (7018.4-9042.1)	8845.1 (7720.7-9944.1)	377.6 (330.0-429.5)	397.7 (348.3-447.8)
	Cardiovascular diseases	6935.6 (6043.4-7900.1)	7336.1 (6414.7-8282.9)	350.3 (303.8-400.7)	359.7 (314.6-404.9)
	Diabetes mellitus type 2	681.2 (465.6-933.2)	1091.1 (749.7-1470.0)	10.4 (6.9-14.6)	19.2 (13.3-25.7)
	Neoplasms	296.3 (235.5-362.8)	348.5 (276.8-427.8)	13.1 (10.5-16.0)	15.4 (12.2-19.0)
Lithuania	All causes	11346.8 (10738.7-11916.8)	7517.8 (6960.6-8101.4)	613.8 (575.8-649.4)	403.9 (372.0-434.5)
	Cardiovascular diseases	10200.8 (9595.2-10750.5)	6538.1 (6040.7-7055.2)	580.9 (542.0-617.3)	379.1 (348.3-409.0)
	Diabetes mellitus type 2	416.0 (295.2-558.6)	477.0 (336.6-645.1)	2.0 (1.5-2.4)	2.4 (1.9-2.9)
	Neoplasms	700.0 (525.0-886.9)	480.9 (391.1-571.3)	30.0 (22.6-38.0)	21.5 (17.6-25.5)
Luxembourg	All causes	5509.4 (5097.9-5949.0)	2334.2 (2048.6-2631.3)	290.1 (267.8-314.0)	113.2 (99.8-126.6)
	Cardiovascular diseases	4691.5 (4327.7-5044.7)	1579.0 (1385.8-1779.9)	264.8 (243.0-287.7)	96.8 (83.9-108.9)
	Diabetes mellitus type 2	394.5 (281.3-530.4)	500.1 (343.9-673.3)	4.1 (3.2-5.1)	2.7 (2.0-3.4)
	Neoplasms	402.5 (335.9-477.3)	230.6 (182.2-289.6)	20.1 (16.9-23.7)	12.3 (9.8-15.1)
Macedonia	All causes	11818.0 (11149.8-12533.3)	6834.9 (6260.7-7426.0)	601.7 (562.1-646.3)	319.3 (292.4-347.7)
	Cardiovascular diseases	10347.5 (9723.9-10991.8)	5603.1 (5124.9-6096.0)	554.6 (515.9-597.5)	285.7 (259.1-312.4)
	Diabetes mellitus type 2	573.5 (411.2-771.5)	737.3 (525.1-985.4)	9.3 (7.1-11.8)	12.5 (9.2-16.2)
	Neoplasms	762.5 (631.3-890.9)	443.2 (364.3-523.7)	32.6 (26.8-38.1)	19.0 (15.7-22.5)
Madagascar	All causes	11296.9 (10145.5-12536.7)	9221.5 (7875.4-10672.5)	493.2 (439.7-555.3)	411.2 (351.0-480.3)
	Cardiovascular diseases	10207.3 (9130.9-11373.4)	8210.0 (6986.4-9577.9)	457.0 (406.2-514.6)	380.6 (323.8-444.8)
	Diabetes mellitus type 2	553.2 (382.0-756.7)	640.1 (437.1-880.0)	12.8 (8.6-17.6)	13.5 (9.2-18.8)
	Neoplasms	422.6 (327.5-524.7)	315.8 (237.2-398.4)	18.2 (14.2-22.6)	14.3 (10.9-18.1)
Malawi	All causes	7294.0 (5462.0-8629.3)	5575.8 (4925.5-6318.1)	339.2 (267.0-402.1)	248.8 (217.2-284.1)
	Cardiovascular diseases	6139.9 (4545.7-7367.1)	4449.3 (3914.1-5056.8)	300.4 (237.1-357.6)	215.1 (187.1-247.0)
	Diabetes mellitus type 2	638.6 (426.2-888.4)	709.5 (473.1-983.6)	15.2 (9.7-21.8)	14.8 (9.8-20.8)
	Neoplasms	389.7 (220.0-569.6)	352.6 (195.5-535.9)	17.3 (10.5-24.2)	15.3 (8.8-22.5)
Malaysia	All causes	11458.9 (10782.0-12145.7)	7554.1 (6917.6-8278.0)	545.7 (512.0-578.3)	339.8 (309.7-372.4)
	Cardiovascular diseases	9822.2 (9264.1-10389.9)	6344.3 (5792.8-6960.6)	488.4 (457.4-518.8)	303.9 (275.9-334.2)

	Diabetes mellitus type 2	707.1 (489.5-966.1)	599.0 (407.1-844.0)	12.3 (9.0-16.2)	4.1 (3.0-5.4)
	Neoplasms	678.6 (578.6-780.3)	450.0 (374.5-534.3)	33.9 (28.8-39.1)	23.5 (19.6-27.8)
Maldives	All causes	14874.1 (13763.6-16202.3)	4394.3 (4004.7-4808.8)	671.8 (620.8-727.2)	205.4 (187.0-223.9)
	Cardiovascular diseases	13158.3 (12154.1-14333.2)	3582.9 (3282.5-3901.7)	612.6 (564.7-663.6)	183.4 (166.0-200.1)
	Diabetes mellitus type 2	734.8 (495.8-1024.1)	507.3 (330.6-727.5)	15.3 (10.0-21.7)	6.0 (4.2-8.3)
	Neoplasms	413.8 (330.5-495.1)	132.5 (106.3-161.2)	18.5 (15.0-22.1)	6.8 (5.5-8.2)
Mali	All causes	7844.9 (7000.7-8712.9)	5713.0 (4975.2-6538.1)	370.9 (329.2-416.8)	271.7 (235.4-309.6)
	Cardiovascular diseases	7022.4 (6262.2-7871.6)	4832.2 (4178.3-5536.6)	346.7 (306.1-392.4)	247.3 (213.5-282.8)
	Diabetes mellitus type 2	501.6 (355.8-670.7)	624.7 (435.9-847.5)	10.2 (7.0-13.7)	12.6 (8.5-17.0)
	Neoplasms	274.7 (197.2-411.4)	215.1 (150.6-312.1)	12.0 (8.8-17.7)	10.0 (7.0-14.3)
Malta	All causes	6666.8 (6114.1-7283.2)	3591.6 (3248.4-3973.4)	355.7 (323.8-387.0)	179.0 (163.8-194.2)
	Cardiovascular diseases	5610.5 (5126.1-6113.1)	2544.3 (2315.9-2761.2)	323.2 (292.6-354.0)	154.8 (139.5-168.6)
	Diabetes mellitus type 2	644.8 (468.8-855.2)	713.5 (512.2-967.8)	12.1 (9.3-15.2)	7.2 (5.6-9.0)
	Neoplasms	383.9 (315.1-451.7)	303.5 (244.5-365.1)	19.0 (15.5-22.4)	15.4 (12.5-18.4)
Marshall Islands	All causes	17801.0 (16287.0-19419.1)	17928.1 (15879.1-20103.7)	752.3 (691.0-818.2)	720.7 (643.9-795.7)
	Cardiovascular diseases	15248.8 (13918.6-16674.2)	14439.1 (12707.1-16189.0)	674.9 (617.7-738.1)	621.5 (553.4-689.7)
	Diabetes mellitus type 2	1770.3 (1241.6-2368.3)	2656.5 (1868.1-3530.1)	36.4 (25.7-48.7)	57.4 (40.1-76.9)
	Neoplasms	567.1 (448.8-694.5)	553.4 (438.3-680.6)	28.1 (22.0-34.6)	26.5 (20.9-32.5)
Mauritania	All causes	9022.4 (8017.7-10025.7)	5748.3 (4988.5-6539.9)	416.0 (368.1-465.6)	267.9 (231.2-305.9)
	Cardiovascular diseases	8024.7 (7083.2-8995.3)	4655.4 (4004.4-5334.3)	385.0 (340.2-431.8)	236.2 (202.6-270.6)
	Diabetes mellitus type 2	634.6 (452.2-842.9)	784.7 (557.7-1045.2)	14.5 (10.1-19.5)	16.3 (11.3-22.4)
	Neoplasms	298.6 (223.9-389.3)	257.1 (196.6-331.1)	13.7 (10.3-17.8)	12.9 (10.0-16.4)
Mauritius	All causes	15422.9 (14518.4-16325.9)	8078.9 (7276.1-8950.1)	687.2 (640.4-733.3)	345.5 (314.2-377.3)
	Cardiovascular diseases	13308.5 (12492.1-14087.6)	4957.7 (4539.1-5373.3)	619.3 (575.4-660.0)	243.0 (220.5-266.5)
	Diabetes mellitus type 2	1069.2 (768.0-1404.0)	2333.9 (1709.0-3005.0)	20.8 (15.3-26.6)	63.3 (46.5-81.6)
	Neoplasms	560.0 (470.5-649.5)	323.4 (272.1-375.9)	25.9 (21.5-30.3)	15.8 (13.2-18.4)
Mexico	All causes	5287.1 (4862.8-5759.6)	4562.6 (4114.6-5070.6)	239.5 (223.5-256.6)	192.6 (176.2-210.0)
	Cardiovascular diseases	3675.3 (3428.1-3943.6)	2697.0 (2461.1-2955.2)	197.6 (182.8-212.5)	145.2 (130.4-160.1)

	Diabetes mellitus type 2	1388.3 (1053.2-1764.4)	1605.3 (1231.2-2034.1)	31.0 (24.0-39.0)	35.3 (27.8-43.6)
	Neoplasms	177.5 (135.6-233.0)	160.5 (134.7-190.1)	8.7 (6.6-11.7)	7.6 (6.3-9.0)
Moldova	All causes	12643.3 (11835.2-13434.9)	9497.4 (8798.2-10244.6)	705.0 (656.1-750.8)	482.4 (444.4-521.2)
	Cardiovascular diseases	11559.9 (10800.2-12308.9)	8559.5 (7912.8-9249.7)	677.7 (629.8-723.0)	461.3 (423.6-500.1)
	Diabetes mellitus type 2	462.0 (317.3-638.2)	475.1 (323.8-664.8)	3.1 (2.3-4.0)	2.6 (1.9-3.3)
	Neoplasms	597.7 (472.2-729.1)	445.6 (373.8-518.3)	23.5 (18.7-28.7)	18.0 (15.1-20.9)
Mongolia	All causes	17286.5 (16038.1-18688.3)	12627.9 (11554.1-13760.0)	857.8 (794.5-928.5)	609.4 (555.0-666.6)
	Cardiovascular diseases	14902.6 (13838.5-16070.1)	11217.0 (10238.3-12264.1)	769.7 (712.1-831.5)	562.8 (510.9-616.4)
	Diabetes mellitus type 2	415.5 (284.7-569.0)	464.6 (323.0-632.2)	0.9 (0.7-1.2)	1.3 (1.0-1.6)
	Neoplasms	1719.9 (1202.1-2262.0)	872.3 (580.7-1177.6)	76.9 (53.9-101.1)	42.2 (28.4-57.1)
Montenegro	All causes	9414.3 (8764.4-10086.9)	6494.7 (5853.1-7172.4)	476.0 (439.0-513.6)	331.7 (297.3-369.2)
	Cardiovascular diseases	8354.9 (7761.9-8978.3)	5602.0 (5043.5-6199.1)	447.1 (410.8-483.2)	312.0 (279.2-347.3)
	Diabetes mellitus type 2	430.0 (289.0-601.5)	504.4 (338.2-700.0)	0.6 (0.5-0.8)	0.9 (0.7-1.3)
	Neoplasms	484.2 (377.1-605.2)	329.0 (257.0-417.6)	21.8 (17.2-26.8)	15.7 (12.3-19.4)
Morocco	All causes	13302.4 (12217.5-14517.1)	9017.0 (7712.1-10376.3)	611.8 (557.8-670.7)	435.7 (373.0-505.4)
	Cardiovascular diseases	12473.0 (11442.2-13616.1)	8077.0 (6893.1-9348.4)	592.2 (538.6-649.4)	414.7 (353.9-481.8)
	Diabetes mellitus type 2	578.4 (401.3-792.0)	748.5 (504.7-1020.6)	8.8 (5.9-12.2)	12.1 (8.1-16.6)
	Neoplasms	211.0 (165.7-255.0)	150.8 (115.4-190.2)	9.2 (7.3-11.1)	7.0 (5.4-8.7)
Mozambique	All causes	10089.2 (8587.1-11580.5)	8408.3 (7304.8-9484.7)	459.2 (385.4-533.1)	367.0 (315.7-421.1)
	Cardiovascular diseases	8882.6 (7543.0-10272.8)	7082.7 (6094.4-8022.9)	417.6 (349.3-486.4)	324.9 (277.0-374.4)
	Diabetes mellitus type 2	690.9 (487.1-922.3)	869.3 (613.4-1157.3)	16.9 (11.4-23.3)	20.1 (14.1-26.9)
	Neoplasms	416.8 (340.8-491.8)	395.5 (312.4-498.5)	20.0 (16.5-23.6)	18.9 (15.0-23.4)
Myanmar	All causes	11411.6 (9905.8-12914.0)	6017.8 (5337.4-6705.4)	498.2 (434.5-562.2)	265.2 (236.5-294.7)
	Cardiovascular diseases	8897.9 (7671.3-10063.6)	4262.9 (3812.3-4761.1)	412.9 (358.6-465.6)	207.0 (185.5-229.7)
	Diabetes mellitus type 2	1055.5 (710.2-1453.0)	1027.2 (696.8-1409.4)	24.1 (15.8-33.7)	23.7 (16.1-32.1)
	Neoplasms	1099.7 (846.3-1359.3)	565.2 (448.1-701.3)	46.4 (35.9-57.2)	26.5 (20.9-32.7)
Namibia	All causes	9141.2 (8200.7-10072.6)	5559.1 (4858.9-6325.3)	422.4 (379.7-464.0)	259.3 (225.4-297.3)
	Cardiovascular diseases	7665.9 (6850.4-8439.5)	4205.5 (3669.2-4809.7)	379.8 (338.7-418.8)	223.9 (193.9-258.6)

	Diabetes mellitus type 2	1122.0 (809.1-1484.0)	1068.6 (755.2-1428.7)	27.3 (19.7-36.1)	22.1 (15.3-29.8)
	Neoplasms	295.6 (236.5-363.8)	239.9 (186.7-301.4)	12.9 (10.5-15.6)	11.1 (8.8-13.6)
Nepal	All causes	7633.6 (6647.1-8704.1)	6652.5 (5736.8-7504.7)	325.7 (281.0-375.3)	303.3 (262.7-344.0)
	Cardiovascular diseases	6850.5 (5928.5-7839.1)	5717.5 (4859.3-6532.6)	302.5 (258.8-349.2)	272.6 (233.8-309.4)
	Diabetes mellitus type 2	383.3 (256.6-531.9)	506.4 (343.5-694.7)	6.4 (4.1-9.1)	11.3 (7.3-15.8)
	Neoplasms	360.5 (275.5-449.9)	322.3 (252.3-391.8)	15.1 (11.7-18.8)	14.8 (11.8-17.8)
Netherlands	All causes	5200.7 (4785.6-5668.0)	2236.6 (1989.3-2512.4)	260.9 (240.2-284.1)	106.6 (97.7-116.3)
	Cardiovascular diseases	4319.0 (3970.0-4663.7)	1437.1 (1309.1-1565.4)	231.5 (212.2-251.6)	84.2 (76.9-92.0)
	Diabetes mellitus type 2	437.5 (315.4-582.1)	442.1 (309.1-607.7)	7.1 (5.4-9.0)	3.6 (2.8-4.6)
	Neoplasms	431.4 (342.9-535.9)	341.6 (256.6-445.7)	21.6 (17.1-26.5)	17.7 (13.5-22.6)
New Zealand	All causes	6306.8 (5824.4-6822.1)	2687.0 (2452.4-2936.5)	320.0 (295.5-346.4)	138.7 (126.7-151.0)
	Cardiovascular diseases	5355.7 (4942.7-5783.4)	1933.9 (1766.7-2094.3)	285.7 (262.0-309.6)	112.2 (101.7-122.7)
	Diabetes mellitus type 2	331.2 (243.4-433.8)	311.9 (225.0-412.6)	5.3 (4.1-6.6)	4.3 (3.3-5.3)
	Neoplasms	597.4 (488.9-714.1)	408.8 (332.0-497.0)	27.9 (23.2-33.1)	20.5 (16.7-25.0)
Nicaragua	All causes	5468.2 (4991.0-5982.9)	3788.8 (3382.9-4263.0)	258.2 (236.4-280.5)	171.8 (154.1-192.6)
	Cardiovascular diseases	4371.2 (4016.7-4755.3)	2629.3 (2355.0-2932.7)	229.2 (209.9-250.0)	139.9 (124.6-156.3)
	Diabetes mellitus type 2	780.6 (549.0-1044.6)	852.5 (610.6-1133.2)	13.4 (9.8-17.2)	17.6 (12.6-23.0)
	Neoplasms	218.3 (153.0-308.6)	180.1 (138.1-233.4)	10.6 (7.3-15.0)	8.7 (6.8-11.1)
Niger	All causes	6962.1 (5828.2-7975.1)	5469.7 (4665.9-6347.6)	328.9 (276.1-378.2)	248.6 (211.2-288.9)
	Cardiovascular diseases	6179.6 (5128.5-7113.9)	4590.3 (3902.5-5361.7)	305.6 (256.0-352.1)	224.0 (190.0-260.9)
	Diabetes mellitus type 2	487.3 (341.6-656.7)	617.7 (433.0-838.2)	9.9 (6.6-13.9)	12.1 (8.3-16.7)
	Neoplasms	253.0 (180.5-337.1)	224.4 (160.6-297.1)	11.6 (8.5-15.5)	10.9 (7.8-14.5)
Nigeria	All causes	4493.5 (3623.9-5501.2)	3436.3 (2613.4-4408.7)	229.8 (184.8-279.6)	169.1 (127.8-216.6)
	Cardiovascular diseases	3964.2 (3162.3-4865.4)	2857.6 (2136.9-3705.3)	212.7 (170.9-258.9)	151.7 (114.2-195.5)
	Diabetes mellitus type 2	304.0 (205.9-414.3)	377.0 (254.8-522.3)	5.9 (3.9-8.1)	7.0 (4.4-10.3)
	Neoplasms	198.0 (138.6-273.4)	178.2 (122.9-245.8)	10.0 (7.1-13.6)	9.3 (6.6-12.6)
North Korea	All causes	8205.6 (7027.0-9371.5)	8224.4 (7232.5-9211.1)	362.8 (302.8-415.2)	374.5 (326.9-421.3)
	Cardiovascular diseases	6704.2 (5689.5-7699.1)	7024.3 (6154.2-7918.5)	313.1 (257.9-360.5)	335.5 (290.4-379.9)

	Diabetes mellitus type 2	313.8 (210.2-444.6)	399.5 (266.7-565.8)	2.8 (1.9-4.0)	3.6 (2.4-5.0)
	Neoplasms	1009.3 (806.9-1234.3)	707.9 (541.2-887.0)	41.1 (32.7-49.8)	31.6 (24.5-39.3)
Northern Mariana Islands	All causes	7241.5 (6368.0-8210.5)	6422.2 (5737.1-7145.2)	318.9 (280.7-360.1)	262.0 (236.7-289.3)
	Cardiovascular diseases	5563.4 (4893.9-6342.6)	4515.6 (4100.9-4952.0)	265.2 (232.8-300.2)	207.2 (187.5-228.6)
	Diabetes mellitus type 2	1118.5 (820.0-1477.1)	1366.1 (968.7-1812.9)	21.1 (15.6-27.7)	24.9 (18.1-32.7)
	Neoplasms	346.4 (274.2-435.3)	294.8 (233.4-367.0)	19.1 (14.8-23.7)	15.5 (12.0-19.1)
Norway	All causes	6269.0 (5820.7-6728.5)	2396.6 (2171.6-2662.9)	319.4 (295.4-342.6)	114.8 (105.4-124.4)
	Cardiovascular diseases	5408.5 (5011.2-5781.9)	1620.2 (1483.1-1760.3)	294.5 (270.8-316.9)	95.8 (87.0-104.5)
	Diabetes mellitus type 2	417.5 (300.2-555.5)	462.2 (327.6-609.1)	3.6 (2.8-4.4)	3.1 (2.5-3.7)
	Neoplasms	429.7 (366.0-502.6)	298.3 (238.2-364.7)	20.8 (17.6-24.3)	15.1 (12.0-18.6)
Oman	All causes	11518.2 (9867.6-13306.8)	6592.3 (5679.2-7560.3)	519.0 (443.7-600.2)	299.2 (258.9-341.8)
	Cardiovascular diseases	10561.0 (8935.5-12293.7)	5515.2 (4711.0-6401.0)	494.4 (420.0-572.0)	274.0 (236.0-314.0)
	Diabetes mellitus type 2	671.6 (470.9-908.3)	903.6 (644.4-1203.3)	11.8 (7.9-16.4)	16.8 (11.8-22.5)
	Neoplasms	230.3 (149.1-341.0)	115.5 (83.6-157.7)	10.5 (6.9-15.5)	5.9 (4.3-8.1)
Pakistan	All causes	9771.6 (8835.3-10754.5)	11811.7 (10305.3-13468.8)	432.3 (388.5-479.4)	551.2 (477.9-629.6)
	Cardiovascular diseases	8851.9 (7952.8-9767.3)	10224.1 (8803.9-11711.3)	406.7 (364.2-452.6)	499.2 (431.4-573.1)
	Diabetes mellitus type 2	495.1 (347.0-676.1)	956.4 (684.2-1270.8)	7.9 (5.4-10.9)	24.2 (16.8-32.8)
	Neoplasms	381.3 (293.9-481.3)	470.7 (358.2-599.6)	15.8 (12.2-19.7)	20.3 (15.7-25.5)
Palestine	All causes	10716.4 (9311.9-12181.9)	6889.7 (6248.9-7572.6)	494.2 (430.9-560.5)	319.2 (290.2-350.3)
	Cardiovascular diseases	9257.1 (7996.4-10580.8)	5460.7 (4951.2-5991.9)	449.3 (389.0-511.3)	277.8 (250.6-306.7)
	Diabetes mellitus type 2	1013.2 (725.5-1338.5)	1064.1 (757.1-1412.0)	25.5 (18.6-33.6)	24.9 (18.3-32.2)
	Neoplasms	379.2 (298.1-468.7)	309.7 (256.0-370.2)	16.2 (12.7-19.8)	13.8 (11.4-16.6)
Panama	All causes	4917.7 (4579.6-5272.1)	3132.0 (2846.5-3458.8)	259.9 (241.0-278.0)	142.8 (130.0-155.9)
	Cardiovascular diseases	4089.2 (3787.4-4381.6)	2160.7 (1981.3-2347.6)	234.8 (216.4-252.8)	113.8 (103.6-124.8)
	Diabetes mellitus type 2	468.1 (323.8-638.2)	652.1 (473.2-866.7)	7.3 (5.3-9.6)	13.3 (9.8-17.3)
	Neoplasms	309.1 (239.8-392.8)	246.0 (202.0-294.2)	15.2 (11.6-19.3)	12.0 (9.8-14.4)
Papua New Guinea	All causes	19248.9 (17079.2-21614.3)	19208.9 (16967.3-21788.6)	715.8 (643.1-794.9)	716.6 (639.1-801.7)
	Cardiovascular diseases	17175.0 (15134.4-19387.9)	16543.0 (14560.6-18804.4)	660.5 (589.4-732.9)	643.8 (571.8-722.7)

	Diabetes mellitus type 2	1442.8 (1024.0-1929.2)	2011.0 (1429.2-2679.7)	26.0 (18.1-35.1)	42.7 (30.1-57.9)
	Neoplasms	505.7 (388.9-628.2)	486.0 (371.5-622.1)	22.7 (17.3-28.3)	21.9 (16.6-27.8)
Paraguay	All causes	6104.0 (5595.9-6610.2)	5103.3 (4454.7-5829.4)	297.9 (273.0-323.0)	233.4 (202.8-267.0)
	Cardiovascular diseases	5202.8 (4752.1-5650.6)	3737.1 (3237.6-4272.6)	272.2 (248.2-296.7)	189.8 (164.3-218.4)
	Diabetes mellitus type 2	595.2 (426.6-792.2)	978.4 (701.2-1283.6)	10.4 (7.7-13.5)	23.9 (17.2-31.9)
	Neoplasms	238.9 (177.1-309.4)	281.7 (225.0-345.0)	12.1 (9.0-15.8)	14.1 (11.2-17.1)
Peru	All causes	4411.1 (4067.4-4782.1)	2389.8 (2081.4-2696.6)	214.7 (195.9-234.9)	106.9 (93.3-121.8)
	Cardiovascular diseases	3634.4 (3349.6-3919.8)	1666.7 (1455.9-1880.7)	187.6 (172.0-203.1)	84.1 (73.6-95.3)
	Diabetes mellitus type 2	329.1 (231.0-447.1)	427.0 (298.0-580.6)	4.9 (3.6-6.3)	7.2 (5.1-9.5)
	Neoplasms	374.1 (238.1-533.4)	239.1 (163.3-338.8)	18.5 (11.7-26.4)	12.5 (8.4-17.5)
Philippines	All causes	8422.5 (7728.6-9080.2)	9541.8 (8458.4-10694.3)	404.7 (371.2-438.6)	435.0 (385.1-488.1)
	Cardiovascular diseases	7474.3 (6906.2-8049.1)	7987.4 (7048.9-8969.2)	375.0 (344.4-406.1)	385.9 (340.9-434.7)
	Diabetes mellitus type 2	421.3 (284.1-586.3)	912.6 (636.0-1237.1)	5.7 (4.0-7.7)	18.3 (12.8-24.8)
	Neoplasms	320.4 (273.2-370.5)	360.6 (293.8-436.5)	14.4 (12.0-16.7)	16.5 (13.4-19.8)
Poland	All causes	11587.3 (10919.0-12244.2)	5210.9 (4779.6-5645.6)	580.2 (541.1-616.0)	257.7 (237.6-277.5)
	Cardiovascular diseases	10322.2 (9682.1-10908.7)	4080.2 (3767.3-4391.6)	540.7 (503.2-575.8)	224.1 (206.1-242.2)
	Diabetes mellitus type 2	484.7 (350.3-639.0)	517.3 (367.8-687.7)	6.0 (4.7-7.5)	5.1 (4.0-6.4)
	Neoplasms	738.4 (589.3-878.8)	588.1 (471.0-716.6)	31.9 (25.7-37.6)	27.4 (21.9-33.2)
Portugal	All causes	6067.1 (5584.9-6646.3)	2712.7 (2423.7-3047.3)	322.2 (295.9-354.3)	119.6 (109.3-131.1)
	Cardiovascular diseases	4980.7 (4545.3-5514.1)	1628.9 (1486.9-1779.9)	289.2 (262.8-322.3)	93.3 (84.4-103.0)
	Diabetes mellitus type 2	587.7 (418.9-796.6)	668.5 (466.5-915.9)	9.2 (6.9-11.9)	7.1 (5.4-9.1)
	Neoplasms	479.2 (379.8-609.9)	391.9 (333.5-455.8)	22.9 (18.4-29.0)	18.1 (15.4-21.1)
Puerto Rico	All causes	6050.0 (5564.1-6577.2)	3266.1 (2877.5-3720.6)	290.1 (267.9-311.2)	125.7 (114.5-138.2)
	Cardiovascular diseases	4647.6 (4281.9-5008.6)	1889.7 (1700.2-2086.1)	248.4 (227.5-267.8)	91.7 (82.2-101.1)
	Diabetes mellitus type 2	977.8 (715.3-1274.6)	1118.9 (812.7-1468.1)	19.7 (15.1-25.0)	21.3 (16.1-26.9)
	Neoplasms	356.5 (297.9-417.0)	213.9 (171.2-271.0)	17.9 (14.6-21.2)	10.3 (8.3-12.7)
Qatar	All causes	9627.2 (8445.0-10910.9)	3771.0 (3182.3-4436.5)	504.3 (440.8-571.0)	180.0 (151.7-211.4)
	Cardiovascular diseases	7927.4 (6856.9-9045.6)	2434.6 (2030.6-2896.5)	440.4 (382.3-500.5)	136.2 (114.0-161.4)

	Diabetes mellitus type 2	1334.4 (955.6-1754.0)	1143.2 (823.5-1505.9)	42.6 (30.6-56.8)	32.6 (23.0-43.2)
	Neoplasms	228.6 (179.9-285.8)	114.2 (90.3-141.5)	12.4 (9.8-15.4)	6.3 (5.0-7.8)
Romania	All causes	12907.5 (12221.8-13665.0)	7029.2 (6501.8-7605.2)	707.0 (662.4-752.6)	366.1 (334.4-399.2)
	Cardiovascular diseases	11679.9 (11047.7-12353.9)	6063.1 (5572.1-6569.8)	670.3 (626.9-713.2)	340.8 (310.7-372.4)
	Diabetes mellitus type 2	382.0 (273.4-523.3)	441.1 (311.3-589.8)	2.7 (2.1-3.5)	3.1 (2.4-3.9)
	Neoplasms	704.7 (584.7-817.3)	479.6 (387.0-579.9)	28.3 (23.4-32.7)	20.2 (16.4-24.3)
Russian Federation	All causes	12410.7 (11691.1-13164.3)	9390.7 (8783.6-10029.1)	634.9 (594.5-675.5)	464.7 (430.9-497.5)
	Cardiovascular diseases	11199.0 (10479.9-11927.7)	8471.4 (7907.1-9072.0)	599.9 (558.4-641.4)	439.3 (406.2-472.1)
	Diabetes mellitus type 2	407.0 (288.7-542.9)	420.7 (300.6-566.4)	2.2 (1.8-2.8)	4.0 (3.1-4.9)
	Neoplasms	778.7 (591.6-1000.0)	476.0 (398.3-553.8)	32.0 (24.3-40.8)	20.7 (17.3-24.1)
Rwanda	All causes	7575.7 (6208.4-9321.0)	3420.8 (2846.8-4011.8)	346.5 (275.8-434.4)	167.2 (132.8-201.7)
	Cardiovascular diseases	6526.2 (5270.8-8076.3)	2701.2 (2183.3-3216.8)	310.3 (245.0-389.7)	145.1 (112.5-177.3)
	Diabetes mellitus type 2	634.0 (417.4-895.1)	525.3 (355.2-738.2)	16.9 (10.4-25.0)	12.1 (7.5-17.8)
	Neoplasms	252.8 (173.9-350.6)	134.0 (94.6-178.6)	11.3 (7.8-15.7)	6.6 (4.8-8.7)
Saint Lucia	All causes	8122.5 (7452.6-8835.5)	4419.6 (3892.5-4993.3)	388.8 (359.1-417.0)	188.4 (169.6-207.1)
	Cardiovascular diseases	6285.1 (5788.4-6805.0)	2914.3 (2599.0-3238.9)	331.4 (304.4-357.5)	145.5 (130.6-161.1)
	Diabetes mellitus type 2	1449.0 (1050.3-1890.4)	1231.9 (885.8-1620.8)	37.5 (27.6-48.6)	29.4 (21.6-38.2)
	Neoplasms	326.6 (258.3-416.6)	234.3 (191.7-280.2)	16.5 (12.7-21.7)	11.4 (9.4-13.6)
Saint Vincent and the Grenadines	All causes	8010.1 (7350.6-8753.8)	6185.2 (5520.1-6959.7)	388.1 (358.0-419.1)	263.9 (239.4-291.6)
	Cardiovascular diseases	6257.5 (5779.3-6757.1)	4255.5 (3853.6-4716.7)	334.2 (306.6-361.4)	211.5 (190.9-233.6)
	Diabetes mellitus type 2	1418.4 (1026.8-1849.8)	1628.1 (1174.9-2131.5)	36.7 (27.3-47.3)	38.3 (28.1-49.8)
	Neoplasms	286.1 (234.1-351.0)	262.3 (220.1-309.7)	14.6 (11.7-18.0)	12.1 (10.2-14.3)
Samoa	All causes	8962.3 (7741.1-10348.9)	9070.6 (7932.3-10241.0)	361.8 (314.1-413.2)	411.0 (362.8-457.7)
	Cardiovascular diseases	7644.5 (6598.4-8787.0)	7093.2 (6178.0-7925.7)	328.7 (285.0-375.7)	355.8 (311.5-397.6)
	Diabetes mellitus type 2	1073.5 (764.2-1440.5)	1676.9 (1182.8-2241.2)	21.2 (14.9-28.4)	37.1 (26.4-49.3)
	Neoplasms	223.7 (177.4-277.7)	217.8 (174.0-266.6)	10.3 (8.2-12.6)	11.3 (8.9-14.1)
Sao Tome and Principe	All causes	5648.7 (5057.0-6296.2)	6135.1 (5386.5-6952.6)	257.8 (229.7-287.0)	285.2 (249.2-327.0)
	Cardiovascular diseases	4990.3 (4467.0-5590.6)	5197.4 (4545.8-5960.8)	238.3 (211.2-267.5)	256.5 (222.3-295.8)

	Diabetes mellitus type 2	337.9 (231.1-465.8)	497.7 (339.5-677.9)	4.5 (3.1-6.0)	6.9 (5.0-9.1)
	Neoplasms	263.9 (201.0-338.0)	351.8 (265.0-448.5)	12.7 (9.7-16.5)	17.5 (13.2-22.6)
Saudi Arabia	All causes	6874.8 (5888.9-8010.5)	6512.2 (5844.6-7272.6)	323.8 (276.2-376.8)	300.1 (271.9-333.4)
	Cardiovascular diseases	5979.6 (5083.8-7050.5)	5519.1 (4925.6-6188.4)	300.4 (254.5-351.5)	275.9 (247.8-307.7)
	Diabetes mellitus type 2	631.0 (442.1-840.7)	713.2 (510.2-959.3)	10.7 (7.6-14.2)	10.2 (7.5-13.0)
	Neoplasms	173.0 (129.2-222.8)	193.2 (158.0-233.3)	8.3 (6.1-11.2)	9.7 (8.0-11.5)
Senegal	All causes	6754.2 (6049.9-7524.7)	6059.5 (5305.0-6880.4)	311.5 (277.4-351.0)	273.4 (238.3-309.9)
	Cardiovascular diseases	5795.2 (5159.5-6502.5)	4922.2 (4314.1-5623.3)	282.0 (249.3-317.6)	240.5 (208.8-274.9)
	Diabetes mellitus type 2	580.3 (412.8-779.8)	802.9 (563.2-1086.7)	11.8 (8.1-16.1)	16.7 (11.2-23.2)
	Neoplasms	323.9 (252.4-407.4)	280.8 (203.9-355.2)	15.2 (11.8-19.1)	13.7 (10.1-17.5)
Serbia	All causes	12560.0 (11722.8-13387.4)	7412.0 (6822.4-8037.1)	643.6 (601.5-687.7)	438.5 (403.7-477.8)
	Cardiovascular diseases	11028.5 (10294.6-11780.1)	6115.1 (5644.1-6670.8)	594.6 (553.2-637.9)	399.4 (366.5-436.7)
	Diabetes mellitus type 2	569.9 (399.7-775.0)	653.4 (464.7-888.0)	8.0 (5.8-10.4)	9.1 (6.6-11.9)
	Neoplasms	811.0 (666.2-962.8)	580.8 (461.4-716.6)	34.6 (28.4-41.0)	26.6 (21.3-32.3)
Seychelles	All causes	9823.9 (8914.8-10785.0)	5968.0 (5371.6-6576.1)	441.6 (391.4-495.8)	269.9 (235.2-308.9)
	Cardiovascular diseases	8415.6 (7583.4-9305.3)	4542.4 (4034.6-5105.5)	394.0 (346.7-446.0)	224.4 (193.0-260.9)
	Diabetes mellitus type 2	421.6 (279.9-600.7)	674.6 (450.1-946.5)	3.9 (2.7-5.3)	7.7 (5.3-10.4)
	Neoplasms	620.5 (528.8-717.6)	496.6 (395.4-608.2)	27.3 (23.2-31.4)	23.5 (18.8-28.6)
Sierra Leone	All causes	7503.7 (6075.7-8752.0)	7778.0 (6760.8-8842.6)	345.6 (281.6-405.6)	351.1 (305.8-401.2)
	Cardiovascular diseases	6752.1 (5478.2-7976.5)	6800.9 (5892.0-7752.2)	322.2 (262.6-379.2)	320.6 (279.3-367.6)
	Diabetes mellitus type 2	417.9 (293.4-567.9)	628.7 (434.6-859.9)	8.1 (5.5-11.0)	13.7 (9.6-18.6)
	Neoplasms	289.0 (211.2-376.9)	303.0 (230.7-381.7)	13.5 (9.9-17.7)	14.8 (11.3-18.7)
Singapore	All causes	7466.0 (6958.2-7978.0)	2638.7 (2369.7-2909.7)	360.6 (336.7-384.2)	112.5 (102.1-123.3)
	Cardiovascular diseases	6049.3 (5635.6-6459.3)	1993.8 (1793.7-2196.3)	308.7 (286.1-330.3)	92.9 (83.1-102.5)
	Diabetes mellitus type 2	457.4 (311.1-636.3)	260.1 (173.3-366.1)	6.6 (4.7-8.8)	1.0 (0.7-1.3)
	Neoplasms	817.9 (695.5-938.7)	299.6 (243.3-358.5)	39.3 (33.1-45.5)	15.2 (12.4-18.3)
Slovakia	All causes	14647.1 (13930.0-15358.1)	6411.7 (5918.8-6941.7)	748.4 (707.4-785.8)	356.3 (329.8-386.3)
	Cardiovascular diseases	13013.9 (12391.1-13628.4)	5309.9 (4886.8-5792.8)	692.4 (653.6-728.6)	322.4 (296.5-350.6)

	Diabetes mellitus type 2	444.4 (320.9-593.8)	444.4 (314.1-604.7)	5.2 (3.9-6.5)	3.2 (2.4-4.0)
	Neoplasms	1030.4 (873.6-1189.1)	614.1 (510.1-723.9)	44.3 (37.6-50.9)	28.8 (24.1-33.7)
Slovenia	All causes	8091.0 (7580.1-8614.8)	3085.1 (2807.2-3390.3)	415.8 (386.3-445.6)	154.8 (139.7-171.9)
	Cardiovascular diseases	6657.1 (6193.4-7115.6)	2229.0 (2025.5-2473.3)	364.6 (335.2-394.0)	130.6 (116.4-146.5)
	Diabetes mellitus type 2	412.4 (298.7-550.3)	443.1 (309.7-596.2)	5.1 (3.9-6.4)	3.7 (2.9-4.6)
	Neoplasms	934.5 (795.6-1072.4)	388.1 (313.1-467.1)	42.6 (35.9-48.9)	19.4 (15.7-23.1)
Solomon Islands	All causes	17170.5 (15143.9-19271.7)	14711.2 (13061.4-16393.4)	706.9 (629.7-789.3)	602.3 (539.6-663.2)
	Cardiovascular diseases	15235.1 (13376.3-17144.1)	12012.4 (10653.6-13332.4)	653.4 (578.6-730.4)	527.9 (472.4-583.0)
	Diabetes mellitus type 2	1361.6 (956.1-1833.0)	2169.7 (1546.7-2890.0)	25.6 (17.9-34.6)	48.7 (34.8-64.4)
	Neoplasms	439.5 (331.5-560.5)	368.9 (283.0-463.2)	20.9 (15.6-26.9)	17.7 (13.4-22.3)
Somalia	All causes	11737.1 (8667.8-15059.0)	9562.5 (7767.8-11627.0)	532.1 (411.3-662.5)	431.0 (346.6-525.0)
	Cardiovascular diseases	10224.9 (7436.0-13167.8)	8134.7 (6516.3-9978.7)	479.4 (369.5-597.7)	385.0 (306.9-469.5)
	Diabetes mellitus type 2	782.7 (539.7-1073.7)	893.2 (632.9-1196.1)	19.9 (12.7-28.2)	20.9 (14.0-29.1)
	Neoplasms	576.6 (370.7-814.7)	457.5 (330.3-604.0)	25.5 (17.2-34.6)	20.8 (15.5-27.2)
South Africa	All causes	5388.5 (4840.5-5961.9)	5024.6 (4518.4-5539.4)	212.6 (187.0-237.8)	219.0 (198.7-239.3)
	Cardiovascular diseases	4146.3 (3685.8-4605.2)	3397.7 (3089.9-3722.0)	177.3 (153.8-201.0)	170.0 (153.0-188.0)
	Diabetes mellitus type 2	759.6 (534.8-1015.3)	1204.9 (873.9-1578.3)	15.4 (10.7-20.6)	29.8 (21.4-38.8)
	Neoplasms	434.3 (320.0-552.1)	367.3 (276.7-464.4)	18.1 (13.5-22.9)	16.8 (12.8-21.1)
South Korea	All causes	8770.0 (8074.6-9501.7)	2265.6 (2018.0-2527.8)	430.9 (395.3-469.6)	104.3 (93.6-116.3)
	Cardiovascular diseases	7026.9 (6469.5-7584.2)	1315.1 (1171.3-1471.6)	369.6 (339.6-400.7)	72.1 (64.3-80.5)
	Diabetes mellitus type 2	437.7 (292.6-614.2)	449.4 (313.8-616.4)	5.7 (4.0-7.9)	6.6 (4.8-8.7)
	Neoplasms	1203.9 (796.8-1599.3)	447.4 (362.2-539.9)	51.2 (34.8-67.6)	23.2 (18.6-28.0)
South Sudan	All causes	8740.6 (6631.4-11157.1)	7004.5 (5637.6-8600.8)	413.1 (322.7-513.7)	316.8 (255.5-386.5)
	Cardiovascular diseases	7318.5 (5496.9-9421.9)	5714.8 (4567.8-7066.3)	361.5 (282.2-452.4)	275.0 (222.1-337.2)
	Diabetes mellitus type 2	720.0 (486.4-1000.1)	820.0 (564.8-1122.1)	19.0 (12.2-27.1)	19.3 (12.7-27.4)
	Neoplasms	517.8 (336.5-733.7)	381.5 (266.6-521.4)	23.3 (16.0-31.8)	17.5 (12.5-23.4)
Spain	All causes	3965.3 (3605.2-4425.8)	1979.8 (1768.6-2247.0)	206.8 (187.4-231.4)	89.5 (80.1-100.2)
	Cardiovascular diseases	3186.6 (2867.4-3562.0)	1300.3 (1170.9-1444.0)	179.8 (160.9-203.0)	69.5 (61.7-78.2)

	Diabetes mellitus type 2	360.6 (260.9-478.2)	332.4 (232.1-458.4)	7.7 (5.8-9.7)	3.2 (2.5-4.1)
	Neoplasms	401.1 (331.0-472.2)	334.8 (276.0-406.4)	18.4 (15.2-21.7)	16.1 (13.2-19.7)
Sri Lanka	All causes	9361.8 (8764.9-10036.1)	5502.2 (4778.3-6260.5)	443.7 (411.8-478.4)	260.0 (225.8-293.5)
	Cardiovascular diseases	8263.5 (7723.8-8870.3)	4172.5 (3599.7-4733.0)	406.2 (376.7-437.9)	217.7 (189.0-246.0)
	Diabetes mellitus type 2	486.6 (339.1-665.7)	957.1 (671.0-1292.2)	9.9 (7.0-13.5)	24.0 (16.7-32.4)
	Neoplasms	371.9 (313.1-429.2)	237.4 (186.0-289.0)	17.1 (14.1-20.0)	11.6 (9.1-14.0)
Sudan	All causes	16310.2 (14187.2-18345.2)	10580.7 (8934.8-12329.6)	737.0 (642.6-829.8)	489.6 (415.5-568.3)
	Cardiovascular diseases	15457.5 (13364.5-17443.8)	9648.8 (8076.4-11327.6)	714.1 (620.8-806.5)	466.7 (394.5-543.8)
	Diabetes mellitus type 2	494.6 (343.1-679.8)	617.5 (428.5-842.1)	7.2 (4.5-10.3)	8.4 (5.7-11.6)
	Neoplasms	313.5 (204.8-452.2)	276.4 (190.8-381.8)	13.8 (9.3-20.1)	12.8 (9.0-17.7)
Suriname	All causes	8680.1 (7896.7-9482.0)	6251.4 (5509.7-7127.0)	405.9 (372.1-438.1)	260.7 (232.2-292.7)
	Cardiovascular diseases	7525.7 (6826.7-8204.7)	4786.5 (4216.7-5415.4)	372.7 (339.9-404.4)	221.6 (196.4-248.4)
	Diabetes mellitus type 2	804.4 (569.1-1088.3)	1103.0 (790.6-1481.0)	15.5 (11.3-20.3)	21.2 (15.4-28.0)
	Neoplasms	294.8 (249.5-344.8)	309.1 (252.7-375.1)	14.6 (12.2-17.2)	14.9 (12.3-17.8)
Swaziland	All causes	8243.1 (7301.8-9291.2)	8554.6 (7124.6-10055.4)	377.9 (332.5-427.1)	377.3 (314.8-448.9)
	Cardiovascular diseases	6234.9 (5480.9-7099.4)	5995.4 (4981.7-7082.6)	315.0 (276.1-359.2)	297.8 (246.9-354.2)
	Diabetes mellitus type 2	1455.6 (1017.4-1929.1)	2028.9 (1378.0-2799.3)	38.7 (27.3-52.1)	56.6 (37.4-79.2)
	Neoplasms	477.2 (352.5-629.1)	436.1 (317.6-584.7)	20.8 (15.6-26.9)	18.9 (14.0-24.8)
Sweden	All causes	5836.2 (5445.5-6228.9)	2654.0 (2402.6-2919.4)	321.3 (297.6-343.9)	136.2 (124.8-148.4)
	Cardiovascular diseases	5106.3 (4747.1-5438.9)	1933.2 (1765.2-2107.2)	299.2 (275.7-321.3)	118.5 (107.5-130.4)
	Diabetes mellitus type 2	361.6 (262.4-473.6)	454.1 (323.4-606.4)	4.4 (3.4-5.4)	4.1 (3.2-5.0)
	Neoplasms	352.3 (293.5-412.5)	246.6 (196.5-303.7)	17.1 (14.4-20.1)	12.7 (10.0-15.5)
Switzerland	All causes	4374.3 (4056.9-4727.3)	1946.6 (1742.3-2171.5)	241.8 (224.0-260.6)	102.8 (92.6-113.7)
	Cardiovascular diseases	3703.0 (3431.8-3977.5)	1291.3 (1158.2-1420.6)	222.4 (204.8-241.0)	86.7 (77.2-97.0)
	Diabetes mellitus type 2	377.8 (270.3-506.6)	397.4 (277.7-546.7)	5.8 (4.4-7.5)	2.9 (2.2-3.7)
	Neoplasms	281.6 (227.2-338.4)	239.6 (188.2-299.5)	13.0 (10.4-15.8)	12.0 (9.4-14.8)
Syria	All causes	14119.0 (12811.1-15467.0)	9675.3 (8353.6-11042.9)	653.9 (592.2-716.6)	457.0 (394.5-524.7)
	Cardiovascular diseases	13511.8 (12238.5-14839.8)	8999.3 (7708.4-10337.4)	642.3 (581.0-705.7)	446.4 (385.3-512.6)

	Diabetes mellitus type 2	360.2 (242.2-514.0)	466.8 (309.9-662.9)	0.6 (0.4-0.9)	0.9 (0.6-1.3)
	Neoplasms	184.5 (146.2-224.4)	166.3 (129.1-208.8)	8.2 (6.4-10.0)	7.7 (6.0-9.5)
Taiwan	All causes	5868.9 (5385.2-6357.7)	2886.3 (2569.3-3230.3)	284.0 (259.7-308.6)	127.0 (115.5-139.3)
	Cardiovascular diseases	4569.3 (4181.9-4970.5)	1780.0 (1600.8-1957.6)	238.8 (217.0-260.6)	87.9 (78.7-97.7)
	Diabetes mellitus type 2	630.5 (438.2-850.6)	577.7 (406.2-774.2)	12.3 (8.8-16.1)	11.2 (8.2-14.8)
	Neoplasms	535.2 (442.6-626.9)	443.7 (364.5-533.8)	25.6 (20.9-30.5)	22.5 (18.2-26.7)
Tajikistan	All causes	10935.0 (10290.2-11671.2)	11394.9 (10441.3-12402.5)	510.2 (474.8-547.1)	535.4 (487.5-584.5)
	Cardiovascular diseases	9474.9 (8898.8-10093.1)	9966.0 (9075.5-10862.5)	473.0 (438.9-506.9)	502.1 (457.2-549.8)
	Diabetes mellitus type 2	632.7 (445.6-862.3)	994.3 (708.8-1330.5)	5.3 (3.8-7.2)	14.4 (10.6-18.8)
	Neoplasms	752.6 (561.5-953.7)	390.4 (261.5-541.6)	29.7 (21.8-37.9)	17.3 (11.8-23.7)
Tanzania	All causes	6110.7 (5103.2-7128.4)	4906.8 (4210.1-5608.5)	294.7 (244.7-349.4)	232.3 (194.5-271.8)
	Cardiovascular diseases	5194.2 (4309.9-6104.5)	4062.4 (3465.6-4696.2)	260.0 (216.0-306.2)	203.5 (169.4-239.5)
	Diabetes mellitus type 2	460.5 (309.6-644.9)	518.2 (345.9-726.4)	12.5 (7.9-18.0)	12.0 (7.5-17.1)
	Neoplasms	333.8 (255.0-413.2)	230.0 (189.6-273.3)	16.2 (12.6-19.8)	11.5 (9.5-13.7)
Thailand	All causes	6788.7 (6243.6-7336.3)	3415.8 (3033.6-3796.0)	313.3 (287.7-339.5)	137.1 (122.9-152.2)
	Cardiovascular diseases	5487.2 (5041.4-5916.1)	2521.9 (2252.8-2807.2)	267.1 (244.0-290.3)	109.4 (97.4-122.0)
	Diabetes mellitus type 2	512.3 (350.4-696.5)	457.7 (305.4-633.6)	11.2 (7.7-15.2)	6.7 (4.6-9.1)
	Neoplasms	546.3 (459.7-637.5)	284.7 (234.8-343.8)	24.1 (20.2-28.0)	13.3 (10.8-16.0)
The Bahamas	All causes	6600.7 (6034.9-7199.8)	4722.6 (4204.9-5260.8)	286.3 (261.9-312.8)	196.9 (174.6-220.9)
	Cardiovascular diseases	5108.1 (4649.9-5551.4)	3462.2 (3083.0-3847.3)	242.9 (219.9-267.3)	163.7 (143.9-184.9)
	Diabetes mellitus type 2	1066.7 (793.2-1368.2)	949.5 (687.7-1232.4)	23.4 (17.4-30.0)	18.2 (13.5-23.2)
	Neoplasms	367.5 (311.5-427.5)	269.6 (226.2-318.7)	17.1 (14.4-19.9)	12.8 (10.7-15.1)
The Gambia	All causes	7608.1 (6467.1-8844.7)	8178.3 (7121.9-9279.0)	355.3 (304.2-410.6)	376.4 (327.2-426.6)
	Cardiovascular diseases	6847.7 (5824.0-8019.7)	7210.7 (6255.2-8198.6)	332.8 (285.4-385.5)	348.6 (303.7-396.0)
	Diabetes mellitus type 2	501.6 (343.3-682.9)	721.6 (495.7-978.7)	10.8 (6.9-15.5)	16.2 (10.9-22.3)
	Neoplasms	211.2 (166.1-261.5)	196.7 (152.3-244.4)	9.7 (7.7-11.9)	9.3 (7.2-11.6)
Timor-Leste	All causes	12104.6 (10739.8-13600.4)	8720.0 (7519.1-9950.7)	546.2 (485.8-615.1)	402.3 (349.1-456.0)
	Cardiovascular diseases	10765.3 (9506.8-12163.2)	7484.4 (6354.0-8583.7)	504.4 (446.3-568.8)	367.0 (316.5-416.6)

	Diabetes mellitus type 2	540.2 (361.2-760.4)	717.2 (477.8-999.7)	7.0 (4.4-10.4)	10.0 (6.5-14.1)
	Neoplasms	556.5 (437.4-686.7)	402.2 (316.3-501.8)	24.7 (19.5-30.9)	19.8 (15.5-24.3)
Togo	All causes	7099.6 (6304.7-7961.2)	6862.6 (5955.2-7890.2)	338.5 (298.4-380.9)	313.1 (271.2-361.8)
	Cardiovascular diseases	6360.1 (5615.3-7167.6)	5925.7 (5100.7-6915.6)	316.0 (277.4-356.9)	285.7 (247.1-331.9)
	Diabetes mellitus type 2	449.0 (313.4-604.8)	625.4 (433.7-845.3)	9.3 (6.4-12.6)	12.8 (8.9-17.7)
	Neoplasms	247.7 (187.0-319.1)	268.7 (198.0-343.8)	11.4 (8.6-14.7)	12.7 (9.4-16.3)
Tonga	All causes	9096.6 (8301.0-9987.2)	8740.4 (7740.0-9837.5)	410.2 (379.6-442.2)	365.0 (325.1-406.4)
	Cardiovascular diseases	6444.9 (5926.9-6969.0)	5464.1 (4903.9-6096.6)	317.6 (292.6-341.5)	257.6 (230.6-287.6)
	Diabetes mellitus type 2	2025.6 (1461.8-2647.9)	2638.8 (1917.1-3450.6)	56.5 (41.2-74.1)	72.2 (51.5-94.3)
	Neoplasms	452.3 (347.1-560.2)	389.2 (298.6-484.0)	25.4 (18.9-31.9)	20.7 (15.8-25.8)
Trinidad and Tobago	All causes	11051.7 (9972.2-12323.9)	6962.0 (5868.0-8190.1)	475.0 (431.2-527.0)	282.0 (237.1-332.3)
	Cardiovascular diseases	8230.2 (7503.5-9056.4)	4476.1 (3769.3-5242.8)	390.4 (353.0-432.6)	209.9 (176.6-246.6)
	Diabetes mellitus type 2	2490.9 (1868.6-3156.9)	2214.1 (1644.6-2850.0)	68.7 (52.1-86.7)	59.5 (43.3-76.9)
	Neoplasms	302.4 (255.7-357.3)	232.7 (178.5-297.6)	14.6 (12.3-17.1)	10.9 (8.5-13.8)
Tunisia	All causes	7848.9 (6961.8-8731.1)	5595.3 (4700.4-6605.0)	447.2 (396.1-497.5)	305.7 (256.4-360.9)
	Cardiovascular diseases	7181.0 (6333.6-8007.4)	4819.4 (4009.8-5743.1)	428.2 (377.3-478.1)	287.1 (239.6-339.8)
	Diabetes mellitus type 2	406.5 (281.7-556.2)	555.8 (375.4-771.5)	5.8 (3.9-7.9)	7.6 (5.2-10.6)
	Neoplasms	225.2 (178.7-271.1)	185.1 (137.6-246.0)	11.3 (8.9-13.8)	9.2 (6.9-12.0)
Turkey	All causes	8355.6 (7456.7-9355.7)	3677.5 (3240.5-4115.0)	363.0 (322.9-404.5)	160.6 (141.5-179.4)
	Cardiovascular diseases	7193.7 (6346.3-8083.1)	2861.3 (2520.3-3195.4)	327.3 (288.4-367.0)	140.6 (122.8-157.5)
	Diabetes mellitus type 2	735.9 (500.6-1002.3)	564.5 (380.0-778.2)	17.3 (11.6-23.6)	8.5 (5.9-11.3)
	Neoplasms	372.4 (273.8-475.2)	208.8 (164.6-260.8)	15.8 (11.7-20.3)	9.5 (7.4-11.8)
Turkmenistan	All causes	16080.0 (15197.0-16927.7)	14088.6 (12964.4-15210.8)	770.9 (722.6-814.9)	662.3 (609.9-715.7)
	Cardiovascular diseases	14464.5 (13676.0-15203.5)	12756.2 (11772.5-13776.9)	727.3 (681.1-771.0)	633.8 (582.0-685.4)
	Diabetes mellitus type 2	586.9 (407.6-811.7)	892.9 (645.4-1182.0)	3.1 (2.3-4.0)	9.5 (7.3-11.9)
	Neoplasms	948.6 (706.2-1185.4)	355.5 (279.3-430.8)	38.3 (28.3-48.0)	15.7 (12.4-19.0)
Uganda	All causes	5108.7 (4154.5-6212.2)	4109.0 (3522.3-4738.0)	244.6 (191.5-302.3)	196.3 (164.4-232.3)
	Cardiovascular diseases	4375.6 (3457.4-5345.9)	3363.0 (2829.9-3962.5)	220.4 (172.7-271.9)	173.3 (143.8-207.0)

	Diabetes mellitus type 2	439.1 (285.6-625.8)	486.0 (310.1-691.7)	10.3 (6.1-15.6)	10.2 (6.4-15.3)
	Neoplasms	182.9 (140.8-231.4)	197.0 (146.7-267.1)	8.4 (6.5-10.7)	9.1 (7.0-12.0)
Ukraine	All causes	12254.4 (11522.0-12976.7)	12405.5 (11423.4-13471.4)	656.1 (614.2-697.8)	644.3 (590.7-699.7)
	Cardiovascular diseases	11036.0 (10344.0-11726.3)	11447.9 (10494.2-12438.2)	622.6 (580.4-663.0)	621.1 (567.4-676.5)
	Diabetes mellitus type 2	409.8 (286.1-559.7)	418.6 (284.1-570.9)	1.8 (1.3-2.3)	1.7 (1.3-2.2)
	Neoplasms	782.9 (596.1-973.5)	521.7 (433.9-614.8)	31.0 (24.0-38.6)	20.9 (17.5-24.5)
United Arab Emirates	All causes	7994.3 (6920.0-9164.9)	7508.5 (6288.5-8805.7)	381.1 (327.2-439.3)	345.4 (290.8-409.8)
	Cardiovascular diseases	6698.0 (5757.8-7745.9)	6013.9 (5018.8-7117.7)	338.1 (289.5-391.5)	298.9 (249.5-355.6)
	Diabetes mellitus type 2	954.8 (683.8-1282.2)	1093.1 (756.1-1462.6)	25.7 (18.0-35.7)	26.7 (17.9-36.3)
	Neoplasms	233.0 (179.0-292.1)	306.0 (234.8-392.0)	11.8 (9.0-14.9)	15.2 (11.8-19.3)
United Kingdom	All causes	6648.6 (6167.4-7190.2)	2678.9 (2453.8-2938.8)	344.8 (318.1-372.8)	127.4 (117.9-137.2)
	Cardiovascular diseases	5852.0 (5403.9-6300.8)	1954.5 (1808.5-2090.9)	313.6 (288.2-338.9)	105.9 (97.6-113.7)
	Diabetes mellitus type 2	255.2 (189.3-337.0)	350.6 (245.1-474.7)	4.1 (3.2-5.0)	2.1 (1.6-2.5)
	Neoplasms	528.8 (424.2-648.6)	361.4 (280.3-452.5)	26.5 (21.4-32.1)	18.8 (14.7-23.3)
United States	All causes	6204.1 (5774.0-6668.0)	3982.4 (3671.6-4301.8)	304.2 (281.1-328.5)	170.7 (158.2-183.1)
	Cardiovascular diseases	5201.0 (4846.4-5553.6)	2908.8 (2706.4-3108.5)	273.2 (251.2-294.7)	143.2 (131.7-154.4)
	Diabetes mellitus type 2	488.6 (377.8-615.6)	647.1 (486.0-825.7)	7.7 (6.4-9.1)	8.0 (6.6-9.6)
	Neoplasms	488.1 (369.0-622.2)	361.3 (271.0-459.0)	22.4 (17.1-28.3)	16.8 (12.6-21.4)
Uruguay	All causes	7826.7 (7371.0-8283.7)	4107.1 (3704.7-4535.0)	373.5 (351.0-395.2)	186.9 (168.2-206.8)
	Cardiovascular diseases	6239.2 (5880.6-6573.1)	2873.8 (2592.5-3176.7)	322.4 (301.7-342.0)	148.9 (134.1-164.4)
	Diabetes mellitus type 2	682.8 (505.6-901.1)	632.9 (459.2-828.8)	9.0 (7.1-11.0)	8.1 (6.2-10.0)
	Neoplasms	844.6 (695.1-989.2)	542.3 (446.8-637.8)	39.2 (32.4-45.7)	26.8 (22.0-31.5)
Uzbekistan	All causes	12745.4 (11924.4-13541.6)	16173.8 (14573.8-17776.2)	609.3 (563.7-652.8)	891.8 (799.8-982.7)
	Cardiovascular diseases	11375.1 (10644.4-12123.6)	14706.4 (13203.4-16228.5)	575.7 (530.2-619.2)	856.8 (763.5-944.7)
	Diabetes mellitus type 2	592.3 (412.4-809.1)	1080.2 (780.3-1412.3)	3.9 (2.9-4.9)	17.8 (13.3-22.8)
	Neoplasms	714.1 (548.7-881.8)	317.7 (239.0-402.1)	28.1 (21.3-35.0)	14.4 (11.2-18.0)
Vanuatu	All causes	17680.4 (14512.7-21172.6)	16636.3 (13365.5-19933.0)	740.8 (622.3-869.3)	698.2 (565.4-828.4)
	Cardiovascular diseases	15975.4 (12995.6-19251.2)	14214.6 (11268.0-17292.6)	691.1 (577.5-811.3)	631.3 (510.3-751.3)

	Diabetes mellitus type 2	1043.6 (723.4-1412.2)	1728.3 (1211.3-2321.8)	16.5 (10.3-23.5)	32.2 (20.3-45.1)
	Neoplasms	518.9 (396.9-663.4)	492.0 (377.4-623.9)	25.1 (19.2-31.6)	23.8 (18.2-30.1)
Venezuela	All causes	7526.8 (7027.7-8036.2)	5215.9 (4569.7-5902.0)	359.5 (333.7-386.5)	239.0 (209.1-270.2)
	Cardiovascular diseases	6407.6 (5973.7-6834.2)	3995.7 (3465.4-4562.7)	324.0 (298.9-348.7)	201.5 (175.8-229.2)
	Diabetes mellitus type 2	716.6 (505.8-955.9)	832.2 (602.6-1114.8)	15.6 (11.4-20.3)	18.9 (13.7-25.1)
	Neoplasms	332.7 (237.7-446.8)	291.9 (233.6-354.9)	16.5 (11.6-22.5)	13.8 (11.0-16.9)
Vietnam	All causes	9188.0 (8297.3-10053.3)	5850.1 (5204.8-6534.5)	418.3 (377.8-460.0)	272.0 (245.1-303.0)
	Cardiovascular diseases	7553.6 (6772.2-8294.5)	4548.5 (4052.3-5117.7)	359.6 (323.9-396.5)	227.5 (203.9-254.0)
	Diabetes mellitus type 2	488.4 (324.9-685.0)	589.8 (386.5-830.7)	9.0 (6.0-12.6)	10.7 (7.1-14.8)
	Neoplasms	913.4 (747.3-1083.1)	590.5 (475.7-724.4)	39.3 (32.5-46.3)	27.3 (22.1-33.3)
Virgin Islands, U.S.	All causes	7355.0 (6647.0-8065.4)	5698.3 (4922.2-6508.5)	369.8 (334.6-405.9)	268.7 (230.3-304.5)
	Cardiovascular diseases	6087.7 (5511.8-6682.7)	4278.8 (3633.8-4911.0)	329.0 (294.8-361.9)	227.4 (192.5-259.4)
	Diabetes mellitus type 2	863.9 (624.3-1134.3)	1034.0 (771.3-1320.5)	19.8 (14.6-25.7)	21.7 (16.5-27.7)
	Neoplasms	348.8 (295.0-412.4)	331.2 (265.7-410.1)	18.1 (15.1-21.1)	16.6 (13.3-20.3)
Yemen	All causes	17641.0 (13254.0-21937.9)	12590.9 (10332.4-15212.3)	780.1 (609.4-954.8)	582.0 (481.9-694.2)
	Cardiovascular diseases	16797.7 (12531.1-20822.8)	11664.5 (9572.3-14187.6)	756.7 (588.8-925.5)	557.8 (461.5-668.9)
	Diabetes mellitus type 2	427.3 (294.1-578.9)	545.6 (379.9-744.2)	5.7 (3.3-8.3)	7.2 (4.8-10.1)
	Neoplasms	372.1 (221.4-590.9)	344.8 (226.0-502.1)	16.0 (10.0-24.6)	15.6 (10.6-22.4)
Zambia	All causes	8541.2 (7515.5-9525.9)	6012.6 (5326.9-6744.9)	397.4 (351.7-447.3)	274.0 (239.7-310.2)
	Cardiovascular diseases	7146.6 (6245.1-8002.3)	4809.9 (4225.7-5431.0)	345.9 (303.4-388.6)	233.9 (203.6-265.2)
	Diabetes mellitus type 2	722.0 (493.2-982.4)	773.4 (532.9-1045.3)	19.2 (12.8-26.7)	18.3 (12.3-24.9)
	Neoplasms	496.3 (389.0-603.8)	345.0 (275.2-417.3)	23.5 (18.6-28.4)	17.0 (13.7-20.5)
Zimbabwe	All causes	5803.0 (5172.3-6471.0)	7776.0 (6725.1-8799.3)	281.8 (249.6-314.9)	347.7 (299.0-395.7)
	Cardiovascular diseases	4654.9 (4151.1-5203.5)	6132.6 (5269.7-6978.8)	247.7 (219.9-276.9)	301.9 (260.1-344.3)
	Diabetes mellitus type 2	654.0 (449.9-891.9)	1103.7 (749.7-1501.9)	12.1 (8.4-16.0)	22.4 (15.2-30.3)
	Neoplasms	449.6 (325.8-570.2)	482.3 (352.9-613.7)	20.1 (14.8-25.3)	20.9 (15.6-26.5)

Supplemental Table 8. Age-standardized proportions of all-cause mortality and DALYs attributable to dietary risks among adults at the national level in 1990 and 2017.

Location Name	Cause	DALYs		Deaths	
		1990	2017	1990	2017
Afghanistan	All causes	24.0 (16.4-27.9)	25.2 (22.9-27.5)	31.6 (23.9-35.7)	32.8 (30.3-35.5)
	Cardiovascular diseases	66.8 (62.0-71.4)	65.2 (60.8-69.6)	61.8 (57.0-66.5)	59.9 (55.4-64.7)
	Diabetes mellitus type 2	45.2 (34.4-56.2)	45.1 (34.3-55.9)	39.0 (29.0-49.5)	39.6 (29.4-50.2)
	Neoplasms	8.9 (5.1-13.5)	9.0 (5.6-13.2)	9.0 (5.5-13.5)	9.1 (6.0-13.3)
Albania	All causes	21.8 (19.8-23.9)	16.5 (14.5-18.6)	34.6 (32.3-36.9)	28.1 (25.7-30.5)
	Cardiovascular diseases	62.8 (59.5-66.2)	49.3 (45.6-52.9)	59.1 (55.4-62.9)	46.2 (42.3-50.0)
	Diabetes mellitus type 2	46.8 (37.3-56.8)	43.9 (34.9-53.5)	38.1 (29.5-47.1)	32.9 (25.1-41.3)
	Neoplasms	12.3 (9.9-14.6)	5.5 (4.1-6.9)	12.7 (10.0-15.2)	5.8 (4.4-7.4)
Algeria	All causes	23.8 (21.2-26.3)	17.3 (15.2-19.4)	36.5 (33.3-39.7)	29.5 (26.6-32.4)
	Cardiovascular diseases	62.7 (58.2-67.0)	53.1 (48.4-58.1)	58.1 (53.4-62.6)	49.5 (44.6-54.6)
	Diabetes mellitus type 2	45.1 (35.0-55.5)	42.3 (32.2-52.6)	35.9 (27.1-45.0)	33.3 (24.6-42.6)
	Neoplasms	7.0 (5.6-8.4)	4.9 (4.1-5.8)	7.5 (6.0-9.1)	5.4 (4.4-6.3)
American Samoa	All causes	17.4 (15.5-19.5)	17.7 (15.9-19.7)	19.8 (18.0-21.7)	21.0 (19.3-22.6)
	Cardiovascular diseases	49.2 (44.4-54.8)	51.5 (47.6-56.0)	45.2 (41.2-49.7)	49.3 (45.5-53.0)
	Diabetes mellitus type 2	40.6 (31.0-50.8)	40.8 (31.0-51.2)	33.3 (24.8-42.7)	33.0 (24.3-42.4)
	Neoplasms	4.9 (3.9-6.0)	4.5 (3.7-5.4)	5.4 (4.3-6.6)	5.4 (4.3-6.5)
Andorra	All causes	11.3 (10.1-12.7)	8.1 (7.1-9.1)	18.7 (17.1-20.3)	14.1 (12.7-15.5)
	Cardiovascular diseases	45.9 (42.1-49.6)	42.0 (38.5-45.7)	43.6 (39.7-47.3)	40.2 (36.5-43.9)
	Diabetes mellitus type 2	44.7 (36.4-53.4)	45.9 (37.6-54.7)	32.6 (25.6-40.3)	31.7 (25.0-39.1)
	Neoplasms	5.6 (4.5-6.8)	5.1 (4.0-6.4)	6.0 (4.9-7.2)	5.5 (4.4-6.9)
Angola	All causes	11.3 (10.0-12.7)	9.9 (8.9-11.0)	14.7 (13.1-16.4)	13.7 (12.2-15.5)
	Cardiovascular diseases	51.3 (46.7-56.0)	46.5 (42.2-50.8)	48.1 (43.5-52.7)	44.4 (39.9-49.4)
	Diabetes mellitus type 2	42.5 (32.5-52.9)	38.9 (29.4-49.4)	35.6 (26.7-45.0)	31.3 (23.0-40.5)
	Neoplasms	7.2 (5.3-9.2)	5.7 (4.5-7.1)	7.5 (5.7-9.6)	6.1 (4.9-7.5)
Antigua and Barbuda	All causes	15.9 (14.4-17.4)	11.4 (10.2-12.7)	22.5 (20.7-24.2)	16.3 (14.8-17.8)
	Cardiovascular diseases	47.8 (44.1-51.7)	37.6 (34.1-41.2)	45.5 (41.9-49.1)	36.4 (33.0-39.9)
	Diabetes mellitus type 2	39.4 (30.2-49.6)	38.4 (29.5-48.3)	32.1 (23.9-41.2)	30.6 (23.0-39.4)

	Neoplasms	4.9 (3.9-6.4)	3.9 (3.3-4.7)	5.6 (4.3-7.4)	4.4 (3.7-5.2)
Argentina	All causes	18.6 (17.0-20.2)	12.1 (11.0-13.1)	26.7 (24.9-28.5)	17.9 (16.6-19.3)
	Cardiovascular diseases	55.5 (52.1-58.9)	48.1 (44.8-51.5)	52.3 (48.6-55.9)	45.6 (41.9-49.2)
	Diabetes mellitus type 2	47.6 (39.0-56.8)	45.8 (37.3-54.9)	39.7 (31.7-48.4)	37.5 (30.0-45.9)
	Neoplasms	7.7 (6.4-8.9)	6.8 (5.8-7.8)	8.3 (6.9-9.8)	7.4 (6.3-8.5)
Armenia	All causes	25.2 (23.2-27.1)	20.2 (18.4-22.0)	37.3 (34.8-39.8)	30.1 (27.8-32.4)
	Cardiovascular diseases	64.2 (60.5-67.4)	57.8 (53.7-61.6)	60.9 (56.8-64.8)	55.1 (50.6-59.3)
	Diabetes mellitus type 2	44.9 (34.8-55.7)	42.6 (32.9-53.1)	38.4 (29.0-48.5)	35.7 (27.0-45.2)
	Neoplasms	10.8 (8.5-13.1)	6.5 (5.2-7.9)	10.8 (8.5-13.3)	7.0 (5.6-8.4)
Australia	All causes	14.9 (13.3-16.7)	7.4 (6.6-8.4)	25.2 (23.2-27.8)	14.7 (13.5-16.2)
	Cardiovascular diseases	53.2 (49.0-57.9)	43.0 (39.4-46.9)	50.5 (46.1-55.5)	41.2 (37.5-45.3)
	Diabetes mellitus type 2	41.5 (32.9-50.3)	40.6 (32.1-49.5)	32.8 (25.4-40.7)	30.6 (23.7-38.3)
	Neoplasms	7.3 (5.8-9.0)	6.2 (4.8-7.7)	7.6 (6.2-9.3)	6.5 (5.1-8.1)
Austria	All causes	14.0 (12.6-15.4)	9.1 (8.1-10.0)	22.6 (20.7-24.5)	18.0 (16.4-19.6)
	Cardiovascular diseases	46.9 (43.2-50.6)	43.5 (40.0-46.9)	42.5 (38.7-46.1)	42.7 (38.6-46.7)
	Diabetes mellitus type 2	38.0 (29.4-46.9)	40.6 (32.1-49.2)	28.8 (21.7-36.8)	29.7 (22.8-37.3)
	Neoplasms	6.5 (5.5-7.6)	5.4 (4.3-6.6)	7.0 (5.9-8.2)	5.7 (4.7-6.9)
Azerbaijan	All causes	28.9 (26.6-31.2)	27.9 (25.5-30.2)	41.5 (38.8-44.1)	39.2 (36.6-41.8)
	Cardiovascular diseases	68.7 (65.2-72.0)	61.6 (57.6-65.4)	65.8 (62.0-69.5)	59.3 (55.3-63.3)
	Diabetes mellitus type 2	44.8 (34.8-55.5)	44.3 (34.7-54.5)	39.1 (29.8-49.3)	37.9 (29.0-47.4)
	Neoplasms	13.9 (10.7-17.3)	9.4 (7.0-12.0)	14.1 (10.5-17.7)	10.2 (7.4-13.1)
Bahrain	All causes	22.9 (20.5-25.4)	13.6 (11.8-15.6)	29.9 (27.2-32.7)	20.6 (18.5-23.0)
	Cardiovascular diseases	57.9 (52.5-63.4)	47.6 (42.8-52.1)	54.6 (49.3-60.0)	45.0 (40.1-49.7)
	Diabetes mellitus type 2	37.1 (27.6-47.0)	35.1 (26.1-44.9)	30.8 (22.4-39.6)	29.0 (21.0-37.8)
	Neoplasms	5.6 (4.4-6.8)	4.7 (3.9-5.7)	5.9 (4.5-7.2)	4.9 (4.0-5.9)
Bangladesh	All causes	10.3 (9.0-11.8)	19.0 (17.1-21.0)	13.3 (11.6-15.3)	26.0 (23.9-28.0)
	Cardiovascular diseases	52.6 (47.8-57.8)	60.0 (55.6-63.9)	47.1 (42.7-51.8)	53.3 (49.1-57.4)
	Diabetes mellitus type 2	37.7 (26.6-49.0)	33.8 (23.4-44.6)	29.3 (20.0-38.7)	25.2 (16.9-34.0)
	Neoplasms	10.4 (8.4-12.4)	10.9 (8.9-13.0)	10.8 (8.6-12.8)	11.6 (9.3-13.7)

Barbados	All causes	16.6 (15.2-18.1)	11.5 (10.4-12.9)	23.7 (22.0-25.4)	15.6 (14.4-17.1)
	Cardiovascular diseases	49.9 (46.2-53.5)	39.8 (36.6-43.3)	47.8 (44.1-51.4)	37.8 (34.6-41.1)
	Diabetes mellitus type 2	42.1 (33.0-51.8)	40.1 (30.8-50.2)	34.4 (26.3-43.2)	31.2 (23.3-39.8)
	Neoplasms	6.4 (5.3-7.5)	5.4 (4.6-6.5)	7.2 (6.0-8.7)	6.0 (5.0-7.1)
Belarus	All causes	23.5 (21.5-25.3)	21.9 (19.7-23.9)	36.7 (34.4-38.9)	36.1 (33.2-38.7)
	Cardiovascular diseases	64.2 (60.5-67.8)	59.1 (54.8-63.1)	62.9 (58.6-66.6)	58.0 (53.3-62.3)
	Diabetes mellitus type 2	50.1 (40.4-59.8)	48.3 (39.3-57.8)	40.6 (32.0-49.6)	36.8 (28.9-45.4)
	Neoplasms	10.9 (7.5-14.5)	7.7 (6.3-9.2)	11.4 (7.8-15.3)	8.3 (6.8-9.9)
Belgium	All causes	13.9 (12.6-15.3)	8.1 (7.3-9.0)	21.8 (20.2-23.5)	13.9 (12.7-15.1)
	Cardiovascular diseases	51.9 (48.2-55.6)	42.5 (39.3-45.6)	48.6 (44.7-52.5)	40.8 (37.4-44.1)
	Diabetes mellitus type 2	43.0 (34.7-52.1)	45.8 (37.3-54.8)	31.9 (24.8-39.7)	32.4 (25.4-40.2)
	Neoplasms	7.4 (5.9-9.2)	6.5 (4.9-8.2)	7.9 (6.3-9.6)	6.8 (5.3-8.6)
Belize	All causes	16.1 (14.5-17.6)	11.3 (10.2-12.6)	23.5 (21.8-25.3)	15.9 (14.5-17.4)
	Cardiovascular diseases	53.7 (49.3-57.9)	44.5 (40.6-48.5)	51.8 (47.6-55.9)	42.8 (38.9-46.8)
	Diabetes mellitus type 2	41.6 (31.6-52.0)	40.9 (31.2-51.2)	33.8 (25.2-43.2)	34.3 (25.6-43.7)
	Neoplasms	5.4 (4.2-7.0)	4.6 (3.9-5.5)	6.2 (4.7-8.0)	5.0 (4.2-6.0)
Benin	All causes	9.4 (8.5-10.5)	9.8 (8.7-10.8)	13.1 (11.8-14.5)	13.2 (11.9-14.6)
	Cardiovascular diseases	51.9 (47.8-56.0)	50.6 (46.2-54.6)	48.0 (43.7-52.2)	46.7 (42.3-50.7)
	Diabetes mellitus type 2	40.5 (30.2-50.8)	37.6 (27.4-48.1)	33.0 (24.0-42.5)	30.1 (21.5-39.4)
	Neoplasms	5.0 (3.8-6.5)	5.4 (4.1-6.8)	5.4 (4.1-7.1)	5.8 (4.4-7.3)
Bermuda	All causes	16.8 (15.0-18.5)	9.6 (8.6-10.7)	24.4 (22.3-26.5)	15.4 (13.7-16.9)
	Cardiovascular diseases	50.1 (45.5-54.7)	37.8 (33.8-41.7)	48.2 (43.8-52.7)	37.0 (33.0-40.8)
	Diabetes mellitus type 2	39.2 (30.8-48.7)	40.7 (32.5-49.0)	31.0 (23.9-39.6)	30.5 (23.4-38.0)
	Neoplasms	4.7 (4.0-5.6)	3.7 (3.0-4.7)	5.5 (4.6-6.3)	4.2 (3.4-5.2)
Bhutan	All causes	12.3 (11.0-13.9)	14.7 (12.9-16.5)	15.6 (13.9-17.7)	20.9 (18.7-23.1)
	Cardiovascular diseases	54.8 (51.0-59.3)	58.5 (54.2-62.3)	50.5 (46.5-54.8)	54.5 (50.2-58.5)
	Diabetes mellitus type 2	40.3 (29.6-51.0)	36.8 (27.0-47.4)	31.2 (22.4-40.4)	28.0 (20.1-37.1)
	Neoplasms	8.2 (6.5-9.9)	8.4 (7.0-9.8)	8.7 (6.9-10.4)	9.0 (7.5-10.4)
Bolivia	All causes	15.1 (13.6-16.7)	12.5 (11.2-13.7)	19.9 (18.0-22.0)	17.8 (16.2-19.4)

	Cardiovascular diseases	57.8 (53.7-61.9)	53.8 (49.8-57.8)	53.6 (49.5-57.6)	50.8 (46.8-54.8)
	Diabetes mellitus type 2	39.2 (29.4-49.8)	37.2 (27.8-47.6)	32.9 (24.0-42.4)	30.5 (22.1-39.7)
	Neoplasms	8.4 (4.2-13.7)	7.1 (4.6-10.3)	9.3 (4.6-15.1)	8.1 (5.1-11.7)
Bosnia and Herzegovina	All causes	24.5 (22.4-26.7)	16.6 (15.0-18.3)	37.2 (34.9-39.5)	26.1 (24.1-28.2)
	Cardiovascular diseases	64.2 (60.6-67.6)	48.3 (44.8-51.7)	60.8 (57.0-64.5)	45.7 (42.2-49.3)
	Diabetes mellitus type 2	43.3 (33.0-53.7)	37.9 (29.1-47.8)	36.0 (26.7-45.7)	29.9 (22.3-38.5)
	Neoplasms	11.8 (9.7-14.0)	7.0 (5.6-8.6)	12.2 (10.0-14.4)	7.3 (5.9-9.0)
Botswana	All causes	9.9 (8.5-11.4)	9.5 (8.5-10.6)	14.8 (12.9-16.6)	15.1 (13.6-16.7)
	Cardiovascular diseases	50.1 (45.8-54.4)	49.5 (45.4-53.5)	46.6 (42.3-50.8)	46.2 (42.0-50.4)
	Diabetes mellitus type 2	40.0 (30.3-50.2)	39.1 (29.8-48.9)	33.7 (24.9-43.2)	32.2 (24.1-41.1)
	Neoplasms	7.5 (5.7-9.6)	5.8 (4.5-7.4)	7.7 (5.9-9.7)	6.2 (4.9-7.7)
Brazil	All causes	16.9 (15.4-18.4)	11.1 (10.0-12.1)	23.8 (22.0-25.5)	16.7 (15.5-18.0)
	Cardiovascular diseases	55.5 (51.6-59.2)	47.2 (43.7-50.6)	51.1 (47.0-54.8)	44.3 (40.7-47.9)
	Diabetes mellitus type 2	39.5 (30.7-49.4)	39.7 (31.4-49.2)	32.1 (24.2-41.0)	31.4 (24.2-40.1)
	Neoplasms	7.6 (5.7-9.6)	6.1 (5.1-7.0)	8.3 (6.2-10.5)	6.6 (5.5-7.6)
Brunei	All causes	23.6 (21.8-25.4)	17.3 (15.8-18.7)	28.6 (26.8-30.4)	22.9 (21.3-24.4)
	Cardiovascular diseases	64.9 (61.1-68.4)	59.5 (55.9-62.9)	60.9 (57.0-64.5)	55.7 (51.8-59.5)
	Diabetes mellitus type 2	40.5 (32.1-49.5)	40.5 (31.8-49.9)	34.1 (26.5-42.6)	33.7 (25.9-42.2)
	Neoplasms	13.0 (11.0-15.1)	9.6 (8.1-11.0)	13.6 (11.3-15.9)	10.3 (8.7-12.0)
Bulgaria	All causes	28.7 (26.5-31.0)	21.9 (19.9-23.8)	42.1 (39.6-44.7)	32.5 (30.2-34.9)
	Cardiovascular diseases	65.4 (61.9-69.0)	54.0 (50.7-57.7)	62.1 (58.1-66.1)	50.7 (47.0-54.6)
	Diabetes mellitus type 2	47.1 (38.2-56.7)	48.0 (39.2-57.3)	39.5 (31.4-48.4)	38.0 (30.3-46.6)
	Neoplasms	13.4 (11.5-15.4)	9.3 (7.8-10.9)	14.6 (12.2-16.8)	9.9 (8.3-11.6)
Burkina Faso	All causes	7.6 (6.4-8.7)	11.1 (10.0-12.2)	10.6 (8.9-12.1)	15.2 (13.7-16.6)
	Cardiovascular diseases	52.9 (48.7-57.3)	54.5 (50.3-58.7)	49.5 (45.0-54.3)	51.1 (46.7-55.4)
	Diabetes mellitus type 2	41.8 (31.5-52.3)	41.5 (31.0-52.1)	34.2 (25.2-44.0)	34.1 (25.0-43.6)
	Neoplasms	5.2 (2.2-7.1)	6.4 (5.0-7.8)	6.0 (2.7-8.1)	7.2 (5.7-8.8)
Burundi	All causes	12.7 (11.2-14.5)	10.9 (9.9-12.0)	15.9 (14.1-18.1)	14.6 (12.9-16.1)
	Cardiovascular diseases	55.0 (49.9-61.3)	55.4 (50.2-60.0)	51.1 (45.8-57.5)	51.7 (45.8-57.1)

	Diabetes mellitus type 2	41.3 (31.2-51.8)	42.1 (31.9-52.6)	35.2 (26.1-44.8)	35.1 (26.0-44.5)
	Neoplasms	7.4 (5.2-9.4)	6.5 (4.9-8.1)	7.9 (5.7-10.0)	7.2 (5.5-8.8)
Cambodia	All causes	16.8 (15.4-18.3)	13.5 (12.2-14.7)	20.8 (19.1-22.5)	17.8 (16.3-19.4)
	Cardiovascular diseases	63.1 (58.6-67.4)	54.8 (50.4-59.2)	57.7 (53.4-62.0)	50.1 (45.9-54.5)
	Diabetes mellitus type 2	37.7 (26.4-49.0)	34.7 (23.8-46.4)	31.2 (21.6-41.4)	28.3 (18.9-38.5)
	Neoplasms	14.1 (11.1-16.8)	9.8 (8.0-11.4)	14.9 (11.9-17.9)	10.8 (8.8-12.6)
Cameroon	All causes	7.3 (6.4-8.2)	7.9 (6.9-8.8)	10.4 (9.3-11.7)	11.0 (9.7-12.3)
	Cardiovascular diseases	43.7 (39.1-48.6)	44.9 (40.4-49.2)	40.9 (36.4-45.7)	41.6 (37.1-45.8)
	Diabetes mellitus type 2	33.7 (24.3-44.0)	33.8 (24.4-44.1)	27.4 (19.3-36.4)	27.5 (19.3-36.4)
	Neoplasms	3.9 (2.9-5.3)	4.4 (3.4-5.6)	4.3 (3.3-5.9)	4.8 (3.7-6.1)
Canada	All causes	15.6 (14.1-17.0)	9.3 (8.4-10.2)	25.3 (23.6-26.9)	17.0 (15.9-18.2)
	Cardiovascular diseases	58.2 (54.6-61.5)	49.1 (45.6-52.6)	56.6 (52.7-60.3)	49.5 (45.7-53.1)
	Diabetes mellitus type 2	46.1 (38.2-54.6)	45.4 (37.4-54.0)	35.7 (28.7-43.3)	34.6 (27.8-42.1)
	Neoplasms	7.2 (5.9-8.6)	6.3 (5.2-7.6)	7.6 (6.3-9.1)	6.9 (5.6-8.3)
Cape Verde	All causes	11.9 (10.6-13.3)	10.8 (9.6-11.9)	19.6 (17.4-21.6)	16.9 (15.2-18.6)
	Cardiovascular diseases	52.6 (47.8-57.2)	48.5 (44.0-52.9)	50.9 (45.5-56.2)	46.0 (41.4-50.7)
	Diabetes mellitus type 2	39.8 (29.5-50.3)	36.1 (27.0-46.3)	31.7 (23.1-41.2)	27.5 (20.0-36.1)
	Neoplasms	6.1 (3.8-9.5)	5.9 (4.4-7.5)	6.5 (4.0-10.5)	6.3 (4.6-8.2)
Central African Republic	All causes	10.2 (8.7-11.6)	11.1 (9.9-12.4)	13.7 (12.0-15.3)	14.8 (13.3-16.3)
	Cardiovascular diseases	51.2 (46.2-56.6)	54.3 (49.7-58.7)	48.1 (43.5-53.2)	51.0 (46.5-55.2)
	Diabetes mellitus type 2	39.4 (29.2-50.2)	40.6 (30.3-51.4)	33.3 (24.1-43.2)	34.3 (25.0-44.1)
	Neoplasms	6.3 (4.6-8.2)	6.7 (5.0-8.5)	6.7 (4.9-8.5)	7.1 (5.3-8.9)
Chad	All causes	9.4 (8.2-10.5)	11.0 (10.0-12.1)	13.3 (11.7-14.9)	15.1 (13.7-16.6)
	Cardiovascular diseases	54.4 (50.0-58.6)	56.8 (52.4-60.7)	50.6 (46.1-55.1)	52.7 (48.2-56.8)
	Diabetes mellitus type 2	44.2 (33.6-54.6)	43.9 (33.3-54.4)	36.3 (27.3-45.9)	36.0 (26.8-45.7)
	Neoplasms	4.8 (2.6-6.5)	6.2 (4.6-7.8)	5.3 (3.0-7.2)	6.6 (5.0-8.5)
Chile	All causes	14.8 (13.5-16.2)	10.9 (9.8-12.0)	22.8 (21.1-24.5)	17.2 (15.5-19.1)
	Cardiovascular diseases	57.5 (54.1-60.6)	50.8 (47.5-54.3)	55.4 (51.8-58.8)	47.6 (43.8-51.9)
	Diabetes mellitus type 2	46.9 (38.0-56.3)	49.2 (41.2-57.8)	40.0 (31.7-48.8)	38.8 (31.6-46.4)

	Neoplasms	9.3 (6.2-12.7)	8.6 (6.5-10.7)	10.1 (6.7-14.0)	9.3 (7.1-11.7)
China	All causes	20.7 (19.0-22.5)	21.3 (19.5-23.2)	26.1 (23.9-28.4)	30.2 (27.9-32.3)
	Cardiovascular diseases	65.5 (60.8-69.9)	64.7 (60.3-68.8)	58.7 (53.6-63.7)	58.2 (53.9-62.5)
	Diabetes mellitus type 2	45.3 (34.7-56.2)	44.9 (35.3-55.0)	35.0 (26.0-44.5)	33.1 (25.1-41.7)
	Neoplasms	20.6 (17.1-24.0)	15.0 (12.5-17.4)	21.4 (17.7-25.3)	15.7 (12.9-18.4)
Colombia	All causes	15.5 (14.2-16.9)	10.9 (9.9-12.0)	23.8 (22.0-25.7)	19.0 (17.5-20.5)
	Cardiovascular diseases	56.9 (53.3-60.5)	52.1 (48.3-55.7)	53.6 (49.7-57.5)	50.3 (46.1-54.2)
	Diabetes mellitus type 2	39.3 (29.6-49.6)	38.5 (29.3-48.8)	31.5 (23.1-40.8)	29.7 (21.9-38.3)
	Neoplasms	8.2 (5.5-11.4)	7.1 (5.5-8.9)	8.9 (5.9-12.4)	7.5 (5.8-9.5)
Comoros	All causes	16.1 (14.5-17.9)	13.7 (12.1-15.4)	20.6 (18.5-23.1)	18.4 (16.3-20.5)
	Cardiovascular diseases	57.7 (52.9-62.8)	56.9 (52.4-60.9)	53.8 (48.7-59.3)	53.4 (48.7-57.8)
	Diabetes mellitus type 2	39.6 (29.4-49.9)	40.2 (30.0-50.5)	33.1 (24.1-42.5)	32.9 (24.3-42.2)
	Neoplasms	7.8 (6.0-9.6)	6.4 (5.0-7.9)	8.5 (6.7-10.5)	7.2 (5.7-8.9)
Congo	All causes	11.8 (10.6-13.2)	11.8 (10.6-12.9)	16.0 (14.4-17.7)	16.2 (14.6-18.0)
	Cardiovascular diseases	51.9 (47.3-56.9)	52.0 (47.9-55.9)	48.9 (44.4-53.6)	49.4 (44.9-53.9)
	Diabetes mellitus type 2	39.9 (30.1-50.3)	39.2 (29.4-49.7)	33.5 (24.8-43.0)	32.2 (23.7-41.4)
	Neoplasms	6.9 (5.2-8.7)	6.4 (5.1-7.8)	7.3 (5.7-9.2)	7.0 (5.7-8.4)
Costa Rica	All causes	15.3 (13.8-16.8)	11.0 (9.9-12.2)	24.5 (22.5-26.7)	17.5 (15.8-19.4)
	Cardiovascular diseases	56.9 (53.3-60.4)	49.1 (45.3-52.8)	55.3 (51.4-59.0)	47.6 (43.6-51.5)
	Diabetes mellitus type 2	40.3 (30.6-50.8)	41.7 (32.4-51.8)	33.0 (24.5-42.5)	31.7 (23.7-40.4)
	Neoplasms	7.6 (4.2-12.2)	6.7 (5.0-8.9)	8.6 (4.6-13.9)	7.2 (5.3-9.7)
Cote d'Ivoire	All causes	11.0 (9.2-12.4)	12.1 (10.9-13.4)	15.8 (13.9-17.6)	16.7 (15.1-18.3)
	Cardiovascular diseases	58.2 (54.3-62.0)	57.5 (53.4-61.4)	54.5 (50.5-58.3)	53.8 (49.7-57.6)
	Diabetes mellitus type 2	37.7 (28.0-48.2)	36.4 (26.7-46.8)	30.6 (22.1-40.0)	29.3 (21.1-38.5)
	Neoplasms	4.1 (3.3-5.1)	3.6 (2.9-4.4)	4.3 (3.4-5.5)	3.8 (3.0-4.8)
Croatia	All causes	24.4 (22.5-26.2)	15.4 (13.9-16.8)	36.6 (34.5-38.8)	25.7 (23.9-27.6)
	Cardiovascular diseases	65.3 (61.8-68.6)	53.2 (49.7-56.6)	61.4 (57.6-65.2)	50.6 (46.6-54.4)
	Diabetes mellitus type 2	42.2 (33.0-52.5)	42.2 (33.1-52.2)	33.6 (25.4-42.8)	30.3 (22.9-38.7)
	Neoplasms	12.9 (10.9-14.8)	8.0 (6.6-9.7)	13.6 (11.5-15.7)	8.4 (6.9-10.0)

Cuba	All causes	18.6 (16.6-20.7)	11.1 (9.6-12.8)	28.2 (25.7-30.8)	16.9 (15.0-19.2)
	Cardiovascular diseases	57.8 (52.7-62.9)	42.4 (37.5-48.1)	55.8 (50.8-60.8)	41.2 (36.5-46.6)
	Diabetes mellitus type 2	41.1 (31.2-51.6)	37.9 (28.8-48.4)	33.1 (24.5-42.6)	28.9 (21.1-37.9)
	Neoplasms	6.6 (5.3-8.2)	4.0 (2.9-5.5)	7.3 (5.9-8.9)	4.5 (3.3-6.0)
Cyprus	All causes	15.4 (13.7-17.2)	11.8 (10.4-13.2)	22.8 (20.7-25.1)	20.0 (18.3-21.9)
	Cardiovascular diseases	49.6 (45.3-54.0)	50.9 (47.3-54.4)	45.6 (41.3-49.8)	46.9 (43.0-50.6)
	Diabetes mellitus type 2	36.6 (28.1-45.8)	40.9 (32.5-50.1)	29.7 (22.3-37.8)	30.8 (23.7-38.8)
	Neoplasms	5.6 (4.6-6.6)	6.2 (5.1-7.5)	5.8 (4.9-6.9)	6.6 (5.4-7.9)
Czech Republic	All causes	27.5 (25.3-29.5)	15.0 (13.6-16.4)	39.2 (37.1-41.2)	26.6 (24.8-28.5)
	Cardiovascular diseases	68.5 (65.2-71.7)	54.0 (50.4-57.5)	64.7 (61.1-68.2)	54.3 (50.3-58.2)
	Diabetes mellitus type 2	43.8 (35.1-53.2)	44.3 (35.9-53.4)	34.3 (26.8-42.7)	30.9 (24.1-38.5)
	Neoplasms	11.9 (10.0-14.0)	8.1 (6.4-9.7)	12.7 (10.7-14.8)	8.5 (6.8-10.2)
Democratic Republic of the Congo	All causes	9.9 (8.6-11.4)	11.2 (9.8-12.8)	13.7 (12.0-15.7)	15.1 (13.3-17.2)
	Cardiovascular diseases	50.3 (45.0-56.0)	51.1 (45.7-56.9)	46.9 (41.4-52.7)	47.1 (41.7-52.8)
	Diabetes mellitus type 2	38.1 (28.0-48.6)	40.1 (29.7-50.7)	30.9 (22.3-40.2)	32.6 (23.7-42.1)
	Neoplasms	6.1 (4.5-8.0)	6.3 (4.8-8.2)	6.5 (4.8-8.5)	6.7 (5.1-8.6)
Denmark	All causes	16.4 (14.7-18.0)	8.0 (7.2-8.9)	26.0 (23.8-28.2)	13.0 (12.0-14.2)
	Cardiovascular diseases	56.1 (51.7-60.5)	41.8 (38.7-44.9)	53.9 (49.4-58.3)	40.7 (37.4-43.9)
	Diabetes mellitus type 2	45.2 (36.6-54.2)	44.5 (35.9-53.5)	34.7 (27.4-42.7)	32.2 (25.1-40.2)
	Neoplasms	6.5 (5.1-8.1)	5.0 (3.6-6.6)	6.8 (5.4-8.4)	5.4 (4.0-7.1)
Djibouti	All causes	15.0 (13.3-16.8)	13.1 (11.5-14.6)	19.5 (17.4-22.0)	17.9 (16.0-19.7)
	Cardiovascular diseases	57.9 (53.0-63.2)	57.1 (52.8-61.2)	53.8 (48.5-59.5)	53.7 (49.0-58.1)
	Diabetes mellitus type 2	43.3 (33.0-53.6)	42.7 (32.4-52.9)	36.3 (27.3-45.9)	35.5 (26.5-44.8)
	Neoplasms	8.9 (7.0-11.1)	7.2 (5.7-8.8)	9.7 (7.6-12.1)	8.0 (6.4-9.7)
Dominica	All causes	14.5 (13.2-15.7)	11.0 (9.9-12.1)	21.1 (19.4-23.0)	14.9 (13.6-16.4)
	Cardiovascular diseases	44.9 (41.4-48.4)	35.3 (32.3-38.5)	43.9 (40.1-47.6)	34.3 (31.0-37.6)
	Diabetes mellitus type 2	40.2 (30.4-50.5)	39.8 (30.3-50.0)	32.5 (24.0-41.6)	31.7 (23.4-40.6)
	Neoplasms	5.2 (3.9-7.1)	4.2 (3.4-5.2)	6.0 (4.3-8.4)	4.5 (3.6-5.7)
Dominican Republic	All causes	15.1 (13.4-16.8)	14.2 (12.4-16.1)	22.5 (20.4-24.4)	20.8 (18.6-23.2)

	Cardiovascular diseases	51.9 (47.1-56.7)	48.3 (43.2-54.0)	48.0 (43.4-52.3)	45.0 (40.3-50.0)
	Diabetes mellitus type 2	37.6 (27.9-48.4)	36.2 (27.3-46.1)	29.9 (21.5-39.3)	29.4 (21.5-38.3)
	Neoplasms	4.6 (3.8-5.4)	3.6 (3.0-4.3)	5.2 (4.2-6.2)	3.9 (3.2-4.6)
Ecuador	All causes	12.8 (11.6-14.1)	10.7 (9.6-11.8)	19.0 (17.2-21.2)	15.9 (14.1-18.0)
	Cardiovascular diseases	54.4 (50.7-58.0)	50.0 (46.4-53.7)	51.2 (47.3-55.1)	47.0 (42.9-51.4)
	Diabetes mellitus type 2	37.9 (29.2-47.8)	39.9 (31.5-49.0)	30.8 (22.9-39.8)	31.2 (23.5-39.7)
	Neoplasms	7.6 (3.4-12.5)	6.2 (4.1-8.9)	8.6 (3.9-14.1)	7.2 (4.6-10.5)
Egypt	All causes	25.8 (23.5-28.1)	22.8 (20.5-25.4)	34.7 (32.2-37.4)	30.2 (27.4-33.3)
	Cardiovascular diseases	64.4 (60.3-68.3)	55.8 (51.3-60.3)	60.1 (56.0-64.1)	51.6 (47.1-56.2)
	Diabetes mellitus type 2	42.4 (32.2-53.4)	38.9 (29.1-49.8)	36.0 (26.9-46.0)	33.0 (24.2-42.8)
	Neoplasms	4.3 (3.5-5.1)	3.2 (2.6-4.0)	4.7 (3.7-5.6)	3.5 (2.7-4.4)
El Salvador	All causes	14.0 (12.8-15.2)	11.4 (10.4-12.6)	21.1 (19.7-22.6)	17.1 (15.6-18.6)
	Cardiovascular diseases	61.2 (57.3-65.1)	54.8 (50.7-58.9)	57.8 (53.8-61.8)	52.5 (48.2-56.8)
	Diabetes mellitus type 2	41.0 (30.7-51.5)	39.7 (30.0-50.1)	33.5 (24.6-43.2)	33.4 (24.7-42.8)
	Neoplasms	5.9 (4.0-8.6)	5.8 (4.3-7.7)	6.8 (4.5-10.0)	6.4 (4.7-8.7)
Equatorial Guinea	All causes	12.9 (11.5-14.5)	7.0 (6.0-8.0)	16.6 (14.9-18.3)	10.6 (9.1-12.4)
	Cardiovascular diseases	53.3 (48.3-58.3)	39.2 (34.5-45.0)	50.1 (45.2-55.0)	38.7 (33.5-45.1)
	Diabetes mellitus type 2	41.6 (31.1-52.1)	34.3 (26.3-43.4)	35.2 (25.8-45.0)	26.7 (19.8-34.7)
	Neoplasms	7.3 (5.2-9.4)	4.0 (3.1-5.3)	7.7 (5.6-9.8)	4.5 (3.5-5.7)
Eritrea	All causes	8.4 (7.1-9.6)	10.4 (9.4-11.4)	11.7 (10.1-13.4)	13.8 (12.2-15.4)
	Cardiovascular diseases	54.9 (49.7-60.1)	53.6 (49.2-57.7)	50.5 (45.0-56.4)	49.7 (44.6-54.8)
	Diabetes mellitus type 2	40.6 (30.1-51.2)	39.9 (29.7-50.4)	35.0 (25.6-45.1)	33.6 (24.4-43.3)
	Neoplasms	7.9 (6.1-9.8)	6.1 (4.8-7.5)	8.5 (6.5-10.5)	6.8 (5.4-8.4)
Estonia	All causes	25.0 (23.1-26.9)	13.5 (11.8-15.6)	39.1 (36.9-41.3)	25.0 (21.7-29.5)
	Cardiovascular diseases	64.2 (60.9-67.4)	47.8 (42.7-54.2)	62.3 (58.4-65.9)	48.3 (42.3-56.2)
	Diabetes mellitus type 2	48.8 (39.8-58.4)	45.8 (36.7-55.5)	40.1 (31.9-49.0)	35.9 (28.1-44.5)
	Neoplasms	9.8 (7.3-12.4)	6.6 (5.4-7.9)	10.4 (7.8-13.1)	7.1 (5.8-8.5)
Ethiopia	All causes	10.9 (9.4-12.3)	9.8 (8.5-11.1)	13.7 (12.1-15.4)	13.7 (11.8-15.7)
	Cardiovascular diseases	60.7 (55.9-65.4)	54.9 (49.7-59.4)	55.8 (50.8-60.9)	51.5 (45.9-56.3)

	Diabetes mellitus type 2	43.1 (32.6-53.6)	41.9 (31.6-52.6)	36.9 (27.3-46.9)	34.6 (25.5-44.0)
	Neoplasms	8.6 (6.4-10.6)	6.7 (5.4-8.1)	9.7 (7.4-11.8)	7.7 (6.2-9.3)
Federated States of Micronesia	All causes	22.4 (20.7-24.2)	23.5 (20.7-25.9)	26.3 (24.4-28.2)	28.1 (25.8-30.2)
	Cardiovascular diseases	59.6 (55.8-63.6)	61.7 (57.9-65.3)	56.5 (52.7-60.4)	57.6 (53.9-61.3)
	Diabetes mellitus type 2	43.3 (33.0-53.9)	42.8 (32.6-53.4)	36.6 (27.3-46.3)	36.0 (27.0-45.6)
	Neoplasms	7.9 (6.3-9.4)	7.4 (6.0-8.7)	9.3 (7.2-11.1)	8.5 (6.9-10.0)
Fiji	All causes	25.3 (22.9-27.5)	26.8 (24.2-29.3)	30.0 (27.6-32.4)	31.1 (28.6-33.4)
	Cardiovascular diseases	59.4 (55.4-63.0)	59.3 (55.6-62.8)	56.8 (52.5-60.8)	56.3 (52.4-60.2)
	Diabetes mellitus type 2	44.4 (34.0-54.7)	42.8 (32.7-53.2)	38.3 (28.7-48.0)	36.9 (27.8-46.4)
	Neoplasms	6.2 (5.1-7.5)	5.7 (4.8-6.8)	7.4 (6.0-9.0)	6.7 (5.5-8.0)
Finland	All causes	18.5 (16.8-20.3)	10.5 (9.5-11.6)	29.1 (27.0-31.1)	19.5 (17.9-21.2)
	Cardiovascular diseases	59.4 (55.6-63.4)	49.0 (45.6-52.6)	56.4 (52.2-60.5)	48.5 (44.4-52.4)
	Diabetes mellitus type 2	44.9 (36.3-54.0)	47.2 (38.8-56.4)	33.1 (25.6-41.2)	34.3 (26.9-42.5)
	Neoplasms	6.0 (4.8-7.3)	5.1 (4.0-6.3)	6.4 (5.1-7.8)	5.4 (4.3-6.7)
France	All causes	10.2 (9.2-11.2)	7.0 (6.3-7.7)	16.7 (15.5-17.9)	12.7 (11.7-13.7)
	Cardiovascular diseases	45.8 (42.6-48.9)	41.7 (38.7-44.8)	43.3 (40.0-46.6)	40.7 (37.3-43.7)
	Diabetes mellitus type 2	45.7 (37.3-54.6)	45.6 (37.0-54.6)	33.6 (26.5-41.6)	33.8 (26.6-41.8)
	Neoplasms	6.9 (5.5-8.4)	6.0 (4.6-7.6)	7.2 (6.0-8.7)	6.4 (5.0-7.9)
Gabon	All causes	9.5 (8.5-10.7)	10.4 (9.3-11.5)	13.2 (11.6-14.7)	14.9 (13.3-16.8)
	Cardiovascular diseases	42.4 (37.8-46.9)	45.6 (41.1-50.2)	40.2 (35.6-44.6)	44.0 (39.3-49.1)
	Diabetes mellitus type 2	32.1 (23.7-41.5)	32.5 (23.9-42.8)	26.0 (18.7-34.6)	26.0 (18.4-34.9)
	Neoplasms	4.2 (3.4-5.3)	4.7 (3.7-5.9)	4.6 (3.7-5.7)	5.1 (4.0-6.3)
Georgia	All causes	31.2 (28.7-33.4)	24.1 (22.1-26.0)	44.4 (41.7-47.1)	34.5 (32.0-37.1)
	Cardiovascular diseases	67.8 (64.2-71.3)	59.0 (55.5-62.6)	63.3 (59.4-67.0)	54.9 (50.9-59.0)
	Diabetes mellitus type 2	44.5 (34.8-55.0)	45.3 (35.5-55.6)	36.5 (27.7-46.3)	37.1 (28.4-46.8)
	Neoplasms	10.5 (8.5-12.7)	7.6 (5.9-9.2)	10.9 (8.7-13.3)	8.2 (6.4-9.9)
Germany	All causes	16.5 (14.9-18.0)	10.0 (9.0-11.1)	26.8 (24.8-28.8)	18.5 (17.0-19.9)
	Cardiovascular diseases	53.0 (49.1-56.6)	46.0 (42.4-49.4)	50.6 (46.4-54.5)	44.6 (40.7-48.2)
	Diabetes mellitus type 2	41.3 (33.4-50.0)	44.5 (36.5-53.2)	31.4 (24.5-39.0)	32.8 (26.1-40.3)

	Neoplasms	7.4 (6.2-8.6)	6.7 (5.4-8.3)	7.9 (6.6-9.1)	7.0 (5.7-8.5)
Ghana	All causes	10.5 (9.4-11.6)	11.8 (10.5-13.1)	14.6 (13.2-16.1)	16.1 (14.5-18.0)
	Cardiovascular diseases	53.6 (49.5-57.9)	52.7 (48.1-57.5)	49.8 (45.7-54.3)	48.9 (44.4-53.7)
	Diabetes mellitus type 2	39.0 (29.2-49.4)	34.0 (24.9-43.9)	31.7 (23.3-41.0)	27.8 (19.8-36.8)
	Neoplasms	3.9 (3.0-4.9)	3.4 (2.6-4.4)	4.3 (3.3-5.6)	3.9 (2.9-5.2)
Greece	All causes	13.9 (12.3-15.5)	11.5 (10.2-12.9)	22.3 (20.3-24.2)	19.5 (17.8-21.2)
	Cardiovascular diseases	45.8 (41.9-49.7)	48.1 (44.1-52.0)	41.2 (37.4-44.9)	43.5 (39.6-47.3)
	Diabetes mellitus type 2	39.6 (30.5-49.1)	42.3 (33.1-52.0)	29.7 (22.0-38.0)	31.7 (23.9-40.1)
	Neoplasms	4.2 (3.3-5.1)	4.7 (3.7-5.7)	4.5 (3.6-5.5)	5.0 (4.0-6.1)
Greenland	All causes	14.1 (13.0-15.4)	10.5 (9.5-11.6)	18.9 (17.5-20.6)	15.1 (13.7-16.4)
	Cardiovascular diseases	52.3 (48.8-56.2)	47.3 (43.7-50.8)	47.6 (43.9-51.5)	43.6 (40.1-47.1)
	Diabetes mellitus type 2	50.0 (42.3-58.1)	48.9 (41.0-57.1)	41.1 (33.9-48.9)	40.8 (33.5-48.5)
	Neoplasms	7.9 (6.3-9.8)	7.6 (5.8-9.3)	8.1 (6.5-10.1)	7.8 (6.0-9.7)
Grenada	All causes	19.1 (17.5-20.8)	14.0 (12.6-15.7)	25.3 (23.4-27.1)	18.4 (16.9-20.2)
	Cardiovascular diseases	51.4 (47.6-55.4)	44.0 (40.1-48.3)	48.6 (45.0-52.4)	41.1 (37.4-45.0)
	Diabetes mellitus type 2	40.9 (31.0-51.4)	39.5 (30.1-49.6)	33.7 (25.0-43.3)	32.0 (23.6-41.1)
	Neoplasms	5.2 (4.3-6.1)	4.4 (3.6-5.4)	5.9 (4.9-7.1)	4.8 (4.0-5.8)
Guam	All causes	20.4 (18.4-22.5)	21.4 (19.6-23.1)	27.8 (25.2-30.4)	28.9 (26.8-31.1)
	Cardiovascular diseases	56.2 (51.8-60.8)	60.4 (56.4-64.2)	55.0 (49.9-60.2)	58.8 (54.5-62.9)
	Diabetes mellitus type 2	41.1 (32.0-51.0)	42.0 (33.1-51.8)	32.9 (24.9-42.0)	33.5 (25.5-42.6)
	Neoplasms	6.2 (4.8-7.9)	4.7 (3.7-6.0)	7.0 (5.5-8.8)	5.2 (4.1-6.6)
Guatemala	All causes	9.2 (8.3-10.1)	10.4 (9.4-11.5)	12.8 (11.6-14.0)	15.1 (13.8-16.4)
	Cardiovascular diseases	55.6 (50.0-61.6)	52.0 (47.3-56.9)	52.0 (46.6-57.7)	49.5 (44.7-54.3)
	Diabetes mellitus type 2	40.1 (29.8-50.8)	39.6 (29.8-50.2)	31.9 (23.3-41.5)	33.2 (24.6-42.7)
	Neoplasms	4.5 (3.2-7.1)	5.0 (3.4-7.8)	5.3 (3.6-8.9)	5.7 (3.7-9.0)
Guinea	All causes	8.6 (7.6-9.7)	11.3 (10.1-12.7)	12.1 (10.7-13.5)	15.0 (13.5-16.6)
	Cardiovascular diseases	46.4 (41.7-51.6)	52.1 (47.5-57.2)	43.2 (38.4-48.5)	48.1 (43.5-53.1)
	Diabetes mellitus type 2	34.8 (25.3-45.2)	35.1 (25.6-45.5)	27.9 (19.9-37.2)	28.6 (20.4-37.6)
	Neoplasms	2.8 (2.1-4.0)	3.5 (2.6-5.0)	3.2 (2.4-4.5)	3.9 (2.9-5.4)

Guinea-Bissau	All causes	11.5 (10.2-12.8)	12.6 (11.3-14.0)	14.5 (12.9-16.2)	16.3 (14.7-17.9)
	Cardiovascular diseases	53.1 (48.5-58.3)	55.3 (50.8-60.1)	48.8 (44.2-54.1)	50.9 (46.5-55.6)
	Diabetes mellitus type 2	35.8 (25.6-46.4)	36.9 (26.9-47.4)	29.5 (20.8-39.1)	30.3 (21.6-39.6)
	Neoplasms	5.8 (4.2-7.7)	6.0 (4.5-7.5)	6.3 (4.6-8.3)	6.5 (4.9-8.1)
Guyana	All causes	21.5 (19.5-23.4)	17.0 (15.2-19.0)	28.0 (25.9-30.1)	23.0 (20.9-25.2)
	Cardiovascular diseases	54.1 (50.0-58.6)	49.3 (44.9-54.2)	50.5 (46.7-54.6)	46.2 (42.0-50.7)
	Diabetes mellitus type 2	40.1 (30.0-50.7)	40.6 (30.7-50.9)	33.7 (24.7-43.4)	34.3 (25.4-43.8)
	Neoplasms	5.7 (4.8-6.9)	4.7 (4.0-5.6)	6.4 (5.3-7.8)	5.1 (4.3-6.0)
Haiti	All causes	17.8 (15.8-19.9)	15.9 (14.0-17.8)	22.9 (20.8-25.1)	20.8 (18.7-22.9)
	Cardiovascular diseases	50.2 (45.6-55.0)	48.6 (43.8-53.7)	47.3 (43.2-51.5)	45.2 (40.7-49.8)
	Diabetes mellitus type 2	39.2 (29.3-50.1)	40.0 (29.7-50.6)	32.9 (23.9-42.9)	33.3 (24.4-42.7)
	Neoplasms	4.7 (3.4-6.6)	4.9 (3.7-6.0)	5.4 (3.9-7.7)	5.2 (4.1-6.5)
Honduras	All causes	14.9 (13.6-16.2)	14.3 (12.9-15.8)	21.1 (19.5-22.7)	21.2 (19.0-23.2)
	Cardiovascular diseases	60.5 (56.8-64.2)	57.0 (52.7-61.1)	56.1 (52.2-59.9)	53.8 (49.3-58.2)
	Diabetes mellitus type 2	39.5 (29.4-50.3)	40.0 (30.1-51.0)	31.7 (22.9-41.6)	32.3 (23.6-42.0)
	Neoplasms	5.0 (3.9-6.4)	4.9 (3.9-5.9)	5.7 (4.3-7.5)	5.5 (4.4-6.8)
Hungary	All causes	26.1 (24.4-27.8)	17.1 (15.7-18.5)	37.9 (35.9-39.9)	27.5 (25.7-29.4)
	Cardiovascular diseases	71.0 (67.8-74.1)	54.9 (51.5-58.4)	67.9 (64.2-71.8)	52.9 (48.9-57.0)
	Diabetes mellitus type 2	47.6 (38.7-56.8)	47.1 (38.1-56.4)	38.5 (30.4-47.1)	36.1 (28.4-44.4)
	Neoplasms	13.0 (10.9-15.1)	10.0 (8.0-12.1)	14.1 (11.9-16.4)	10.4 (8.4-12.6)
Iceland	All causes	16.2 (14.6-17.9)	9.0 (8.1-10.0)	27.3 (25.3-29.3)	17.1 (15.7-18.5)
	Cardiovascular diseases	59.8 (55.5-64.0)	47.9 (44.2-51.5)	57.2 (52.7-61.5)	47.2 (43.1-51.0)
	Diabetes mellitus type 2	47.6 (38.9-56.7)	45.8 (37.2-54.9)	36.0 (28.4-44.0)	32.3 (25.3-40.2)
	Neoplasms	6.3 (5.0-7.6)	4.6 (3.5-5.9)	6.7 (5.3-8.1)	5.0 (3.8-6.3)
India	All causes	13.2 (12.1-14.3)	14.0 (12.7-15.4)	15.9 (14.6-17.1)	17.6 (16.1-19.3)
	Cardiovascular diseases	60.9 (57.2-64.6)	56.8 (52.6-61.3)	56.1 (52.2-59.8)	51.6 (47.1-56.5)
	Diabetes mellitus type 2	41.1 (30.7-51.8)	38.0 (28.3-48.5)	32.6 (23.7-42.0)	29.7 (21.7-38.6)
	Neoplasms	9.0 (7.0-11.0)	6.9 (5.7-8.0)	9.3 (7.3-11.3)	7.1 (6.0-8.2)
Indonesia	All causes	18.6 (17.1-20.1)	20.1 (18.4-21.8)	23.0 (21.3-24.9)	25.3 (23.6-27.2)

	Cardiovascular diseases	67.9 (64.1-71.4)	60.3 (56.5-63.9)	62.1 (58.0-66.1)	55.2 (51.5-59.0)
	Diabetes mellitus type 2	37.9 (26.9-49.2)	35.1 (24.5-46.1)	31.5 (21.9-41.5)	29.3 (20.2-39.1)
	Neoplasms	11.6 (9.6-13.4)	7.7 (6.4-8.9)	12.6 (10.4-14.7)	8.6 (7.1-10.1)
Iran	All causes	19.4 (17.6-21.2)	13.8 (12.2-15.4)	31.2 (28.9-33.3)	23.5 (21.2-25.7)
	Cardiovascular diseases	59.0 (54.9-63.0)	46.2 (42.2-50.1)	55.0 (51.0-59.1)	43.0 (38.7-47.1)
	Diabetes mellitus type 2	39.6 (29.6-50.4)	35.3 (25.8-45.7)	31.4 (22.8-41.0)	27.8 (19.7-36.9)
	Neoplasms	8.1 (5.4-12.1)	6.3 (4.7-8.5)	8.5 (5.5-12.9)	6.7 (5.0-9.3)
Iraq	All causes	24.5 (22.2-27.0)	16.0 (14.4-17.6)	34.2 (31.5-36.9)	29.1 (27.0-31.3)
	Cardiovascular diseases	58.8 (54.5-62.9)	57.0 (53.0-61.3)	54.8 (50.5-58.9)	54.1 (49.8-58.6)
	Diabetes mellitus type 2	43.8 (33.5-54.5)	45.2 (35.0-55.7)	36.7 (27.6-46.4)	36.8 (27.8-46.1)
	Neoplasms	6.0 (4.7-7.4)	6.2 (5.0-7.3)	6.5 (5.1-7.9)	6.8 (5.5-8.0)
Ireland	All causes	18.3 (16.4-20.2)	9.0 (8.1-10.0)	26.5 (24.3-28.7)	16.1 (14.7-17.4)
	Cardiovascular diseases	56.2 (51.8-60.7)	44.9 (41.3-48.2)	52.5 (48.0-57.0)	43.5 (39.7-47.3)
	Diabetes mellitus type 2	42.1 (33.5-51.3)	42.5 (34.3-51.5)	32.2 (24.9-40.3)	28.2 (21.7-35.6)
	Neoplasms	7.4 (5.9-9.0)	5.5 (4.3-7.0)	7.9 (6.4-9.5)	6.0 (4.7-7.5)
Israel	All causes	13.7 (11.9-15.5)	7.0 (6.2-7.8)	22.2 (19.8-24.7)	12.4 (11.2-13.6)
	Cardiovascular diseases	46.8 (41.9-52.5)	37.8 (34.4-41.3)	44.4 (39.3-49.9)	36.3 (32.7-40.0)
	Diabetes mellitus type 2	36.9 (27.7-46.7)	37.4 (28.6-46.7)	29.5 (21.8-38.3)	28.1 (21.0-36.1)
	Neoplasms	4.6 (3.9-5.5)	4.3 (3.5-5.3)	5.2 (4.3-6.1)	4.8 (3.8-5.9)
Italy	All causes	12.2 (11.1-13.4)	8.2 (7.4-9.1)	19.7 (18.1-21.2)	15.4 (14.1-16.9)
	Cardiovascular diseases	45.2 (41.8-48.4)	42.0 (38.7-45.5)	40.8 (37.3-44.1)	39.5 (35.8-43.4)
	Diabetes mellitus type 2	41.2 (32.6-50.1)	42.7 (34.0-51.9)	32.8 (25.3-40.8)	31.1 (23.9-38.9)
	Neoplasms	6.3 (5.2-7.5)	5.3 (4.5-6.3)	6.8 (5.5-8.3)	5.7 (4.7-6.7)
Jamaica	All causes	15.1 (13.6-16.8)	13.3 (11.6-15.2)	22.1 (20.3-24.1)	18.4 (16.5-20.5)
	Cardiovascular diseases	44.5 (41.0-48.4)	43.3 (39.1-48.1)	41.9 (38.4-45.9)	40.6 (36.8-44.8)
	Diabetes mellitus type 2	38.1 (28.5-48.6)	38.7 (29.1-48.9)	31.7 (23.2-41.2)	31.9 (23.5-41.2)
	Neoplasms	5.3 (4.3-6.6)	4.9 (4.0-6.1)	6.2 (4.9-7.9)	5.3 (4.3-6.5)
Japan	All causes	15.7 (14.2-17.3)	9.5 (8.5-10.5)	24.3 (22.4-26.4)	15.6 (14.3-17.0)
	Cardiovascular diseases	56.3 (52.8-59.9)	49.5 (46.3-52.9)	51.3 (47.5-55.6)	44.0 (40.6-47.7)

	Diabetes mellitus type 2	40.9 (32.1-50.3)	46.2 (37.5-55.3)	31.4 (23.8-39.7)	33.6 (26.4-41.6)
	Neoplasms	17.2 (14.3-20.1)	11.5 (9.6-13.3)	17.3 (14.0-20.7)	11.7 (9.6-13.8)
Jordan	All causes	21.2 (19.1-23.4)	14.8 (13.2-16.7)	29.9 (27.6-32.5)	23.2 (21.2-25.3)
	Cardiovascular diseases	56.1 (52.2-60.2)	48.4 (44.5-52.3)	51.5 (47.7-55.5)	44.0 (40.0-48.1)
	Diabetes mellitus type 2	41.4 (31.2-52.0)	41.6 (31.5-52.0)	35.9 (26.7-45.7)	33.7 (25.1-43.2)
	Neoplasms	7.4 (6.1-8.6)	7.0 (5.7-8.4)	7.7 (6.5-9.0)	7.4 (6.1-8.8)
Kazakhstan	All causes	25.8 (23.8-27.6)	21.4 (19.5-23.3)	37.0 (34.8-39.1)	32.9 (30.6-35.1)
	Cardiovascular diseases	67.9 (64.4-71.2)	55.6 (51.8-59.4)	64.0 (60.0-67.6)	54.8 (50.8-58.5)
	Diabetes mellitus type 2	50.5 (40.7-60.1)	49.6 (40.4-59.0)	42.0 (32.9-51.3)	40.9 (32.3-50.0)
	Neoplasms	16.0 (12.7-19.2)	9.4 (7.5-11.3)	16.3 (12.9-19.7)	10.1 (8.1-12.2)
Kenya	All causes	8.7 (7.7-9.9)	8.5 (7.4-9.6)	12.0 (10.5-13.9)	11.9 (10.4-13.6)
	Cardiovascular diseases	48.0 (43.9-52.2)	49.1 (44.9-53.3)	44.6 (40.3-49.1)	45.7 (41.5-50.0)
	Diabetes mellitus type 2	37.1 (27.3-47.4)	37.7 (27.9-48.0)	30.5 (22.0-39.7)	30.9 (22.4-40.1)
	Neoplasms	7.1 (5.5-8.7)	5.5 (4.2-7.1)	7.7 (6.0-9.4)	6.1 (4.7-7.8)
Kiribati	All causes	21.5 (19.7-23.3)	23.5 (21.3-25.6)	23.3 (21.4-25.2)	24.9 (22.9-26.9)
	Cardiovascular diseases	61.6 (57.5-65.7)	62.8 (59.0-66.5)	58.6 (54.5-62.4)	58.7 (55.1-62.3)
	Diabetes mellitus type 2	44.4 (33.8-55.3)	44.8 (34.3-55.5)	38.9 (29.3-49.0)	39.1 (29.6-49.1)
	Neoplasms	6.8 (5.5-8.1)	6.6 (5.4-7.8)	8.3 (6.6-10.0)	7.7 (6.2-9.1)
Kuwait	All causes	17.2 (15.6-18.9)	14.2 (12.7-15.9)	27.1 (25.0-29.3)	24.3 (22.2-26.4)
	Cardiovascular diseases	56.4 (52.0-60.7)	52.5 (48.2-56.5)	52.6 (48.0-57.3)	48.6 (44.3-52.9)
	Diabetes mellitus type 2	42.2 (32.9-52.2)	44.1 (34.8-53.9)	33.3 (25.2-42.4)	33.0 (25.1-41.7)
	Neoplasms	5.3 (4.4-6.2)	5.4 (4.3-6.6)	5.6 (4.7-6.6)	5.8 (4.6-7.0)
Kyrgyzstan	All causes	24.4 (22.6-26.2)	23.9 (21.6-26.1)	34.5 (32.5-36.5)	38.3 (35.5-41.0)
	Cardiovascular diseases	65.6 (62.2-69.0)	61.3 (56.9-65.5)	62.1 (58.4-65.7)	60.6 (56.0-65.0)
	Diabetes mellitus type 2	48.6 (38.6-58.9)	47.1 (36.8-57.5)	41.6 (32.1-51.3)	40.9 (31.3-50.7)
	Neoplasms	15.6 (11.8-19.6)	8.4 (5.8-11.3)	15.5 (11.7-19.5)	9.1 (6.4-12.1)
Laos	All causes	24.1 (22.2-26.0)	18.3 (16.4-20.2)	29.2 (27.1-31.3)	23.6 (21.6-25.6)
	Cardiovascular diseases	67.5 (63.5-71.2)	54.8 (50.5-58.7)	62.8 (58.5-66.7)	51.0 (47.0-54.9)
	Diabetes mellitus type 2	37.3 (26.3-48.9)	32.8 (22.5-43.6)	30.8 (21.2-41.1)	26.6 (17.8-36.2)

	Neoplasms	13.6 (10.9-16.2)	8.4 (6.9-10.0)	14.5 (11.7-17.3)	9.3 (7.6-11.1)
Latvia	All causes	24.5 (22.7-26.2)	17.9 (16.4-19.7)	38.4 (36.4-40.4)	30.2 (28.2-32.4)
	Cardiovascular diseases	64.0 (61.0-66.9)	53.3 (50.4-56.5)	61.9 (58.5-65.2)	53.5 (50.2-56.9)
	Diabetes mellitus type 2	50.8 (41.6-60.2)	48.3 (39.5-57.6)	41.1 (32.8-50.0)	38.4 (30.5-47.0)
	Neoplasms	10.1 (7.6-12.6)	7.7 (6.3-9.0)	10.6 (8.1-13.3)	8.2 (6.7-9.6)
Lebanon	All causes	16.5 (14.4-18.7)	15.1 (13.0-17.4)	23.7 (20.6-26.6)	24.0 (21.2-27.3)
	Cardiovascular diseases	47.0 (40.9-52.5)	49.1 (43.6-55.3)	42.9 (37.1-48.4)	45.5 (39.7-51.6)
	Diabetes mellitus type 2	34.5 (24.7-45.1)	36.5 (26.4-47.2)	27.8 (19.4-37.1)	28.5 (19.9-37.5)
	Neoplasms	3.8 (3.0-4.7)	4.6 (3.7-5.7)	4.1 (3.3-5.1)	5.1 (4.1-6.3)
Lesotho	All causes	10.5 (9.3-11.6)	9.5 (8.3-10.7)	14.6 (13.1-16.2)	13.9 (12.4-15.6)
	Cardiovascular diseases	47.6 (43.2-52.0)	50.0 (45.4-54.7)	44.1 (40.1-48.4)	45.7 (41.4-50.5)
	Diabetes mellitus type 2	37.3 (26.6-48.0)	35.8 (25.3-46.9)	32.0 (22.5-42.0)	30.7 (21.4-40.9)
	Neoplasms	7.4 (5.3-9.5)	7.1 (5.2-9.0)	7.5 (5.5-9.5)	7.2 (5.4-9.0)
Liberia	All causes	9.0 (8.1-10.0)	11.3 (10.1-12.6)	13.2 (11.9-14.7)	16.1 (14.5-17.7)
	Cardiovascular diseases	52.8 (48.3-58.0)	56.2 (51.6-60.8)	49.3 (44.6-54.7)	52.4 (47.9-57.1)
	Diabetes mellitus type 2	37.2 (27.3-47.6)	38.9 (28.9-49.3)	30.2 (21.7-39.5)	31.6 (23.0-40.9)
	Neoplasms	5.0 (3.9-6.3)	6.0 (4.5-7.5)	5.5 (4.3-6.9)	6.4 (4.8-8.1)
Libya	All causes	18.5 (16.4-20.6)	19.2 (17.2-21.3)	27.6 (25.1-30.1)	27.9 (25.5-30.5)
	Cardiovascular diseases	56.6 (51.8-61.3)	59.1 (54.1-64.1)	52.3 (47.5-57.0)	54.4 (49.2-59.5)
	Diabetes mellitus type 2	40.9 (31.2-51.6)	42.6 (32.7-53.3)	32.9 (24.3-42.4)	35.3 (26.3-45.0)
	Neoplasms	6.0 (5.0-7.2)	6.4 (5.3-7.8)	6.2 (5.2-7.5)	6.8 (5.5-8.2)
Lithuania	All causes	22.5 (20.7-24.2)	17.1 (15.6-18.5)	37.9 (35.6-40.0)	30.4 (28.2-32.5)
	Cardiovascular diseases	65.4 (61.6-68.7)	56.2 (52.7-59.4)	64.6 (60.4-68.6)	56.2 (52.1-60.0)
	Diabetes mellitus type 2	50.8 (41.7-60.1)	49.3 (40.6-58.7)	40.9 (32.7-49.6)	39.9 (32.1-48.5)
	Neoplasms	9.7 (7.3-12.3)	7.4 (6.1-8.7)	10.3 (7.8-13.0)	7.9 (6.5-9.3)
Luxembourg	All causes	13.7 (12.4-15.0)	7.9 (7.1-8.7)	21.6 (20.0-23.4)	13.7 (12.4-15.1)
	Cardiovascular diseases	46.9 (43.5-50.3)	38.0 (34.4-41.5)	44.1 (40.5-47.7)	38.3 (34.6-41.7)
	Diabetes mellitus type 2	41.2 (33.1-50.1)	44.2 (36.2-52.5)	29.5 (22.9-37.1)	29.2 (22.7-36.4)
	Neoplasms	5.3 (4.5-6.3)	4.2 (3.3-5.2)	5.9 (5.0-6.9)	4.7 (3.8-5.8)

Macedonia	All causes	27.2 (25.0-29.7)	18.8 (16.9-20.7)	38.5 (35.9-41.3)	28.6 (26.3-30.8)
	Cardiovascular diseases	61.9 (58.3-65.6)	49.9 (46.6-53.5)	56.1 (52.3-60.2)	45.4 (41.8-48.9)
	Diabetes mellitus type 2	42.9 (33.6-52.7)	41.4 (32.1-51.2)	35.6 (27.4-44.7)	33.4 (25.0-42.2)
	Neoplasms	12.8 (10.6-14.9)	7.1 (5.9-8.4)	13.6 (11.2-15.9)	7.6 (6.3-8.9)
Madagascar	All causes	16.0 (14.5-17.8)	15.3 (13.8-17.0)	20.2 (18.2-22.6)	19.6 (17.8-21.8)
	Cardiovascular diseases	53.5 (48.8-58.7)	51.6 (47.3-56.0)	50.0 (45.1-55.6)	48.1 (43.6-52.8)
	Diabetes mellitus type 2	36.2 (26.3-46.6)	36.8 (26.7-47.2)	29.9 (21.2-39.3)	30.5 (21.8-39.9)
	Neoplasms	7.2 (5.6-8.8)	6.5 (5.1-8.1)	7.9 (6.2-9.8)	7.2 (5.7-9.0)
Malawi	All causes	8.7 (6.6-10.4)	9.1 (8.2-10.1)	12.1 (9.5-14.4)	12.7 (11.3-14.3)
	Cardiovascular diseases	54.8 (49.3-60.0)	52.0 (47.2-56.5)	50.6 (45.0-56.6)	48.4 (43.2-53.4)
	Diabetes mellitus type 2	34.5 (24.2-45.0)	34.6 (24.5-45.1)	28.2 (19.6-37.8)	28.2 (19.4-37.5)
	Neoplasms	6.9 (4.4-9.6)	6.4 (3.7-9.4)	7.4 (4.8-10.0)	6.8 (4.1-9.8)
Malaysia	All causes	24.7 (22.8-26.4)	19.2 (17.6-20.9)	31.7 (29.7-33.6)	24.7 (22.9-26.5)
	Cardiovascular diseases	67.7 (64.0-71.0)	62.4 (58.8-65.9)	63.5 (59.7-67.0)	59.5 (55.6-63.1)
	Diabetes mellitus type 2	40.3 (30.3-50.9)	41.3 (31.6-51.9)	32.8 (24.0-42.2)	31.9 (23.7-40.9)
	Neoplasms	13.0 (11.1-14.9)	9.5 (8.0-11.2)	14.4 (12.2-16.6)	10.9 (9.2-12.8)
Maldives	All causes	26.2 (23.9-28.6)	16.4 (14.6-18.1)	33.1 (30.6-35.8)	26.1 (24.0-28.2)
	Cardiovascular diseases	70.0 (65.8-73.9)	59.5 (55.3-63.5)	65.8 (61.4-69.7)	56.6 (52.3-60.7)
	Diabetes mellitus type 2	40.0 (29.5-50.8)	35.1 (25.0-45.9)	33.7 (24.5-43.9)	26.8 (18.5-35.9)
	Neoplasms	10.1 (7.8-12.6)	5.5 (4.5-6.6)	10.5 (8.4-12.6)	6.2 (5.0-7.3)
Mali	All causes	10.3 (9.3-11.4)	10.3 (9.2-11.7)	14.2 (12.7-15.8)	15.0 (13.3-16.7)
	Cardiovascular diseases	53.0 (48.7-57.5)	51.5 (47.2-55.9)	49.0 (44.7-53.8)	47.6 (43.3-51.9)
	Diabetes mellitus type 2	42.6 (32.4-53.1)	39.8 (29.6-50.0)	34.9 (25.9-44.6)	31.9 (23.2-41.4)
	Neoplasms	4.2 (3.0-6.3)	4.1 (2.8-5.9)	4.5 (3.3-6.6)	4.5 (3.1-6.3)
Malta	All causes	18.5 (16.5-20.4)	12.4 (11.2-13.7)	28.0 (25.6-30.4)	21.0 (19.4-22.8)
	Cardiovascular diseases	53.6 (49.1-58.1)	48.9 (45.2-52.6)	50.3 (45.8-54.8)	46.7 (42.8-50.7)
	Diabetes mellitus type 2	42.9 (34.3-52.2)	45.9 (37.5-55.1)	33.4 (25.8-41.6)	33.4 (26.4-41.3)
	Neoplasms	6.6 (5.5-7.8)	6.2 (5.1-7.5)	7.0 (5.8-8.3)	6.7 (5.5-8.0)
Marshall Islands	All causes	23.8 (21.8-25.9)	25.2 (23.1-27.5)	27.6 (25.4-29.8)	29.0 (26.9-31.4)

	Cardiovascular diseases	60.1 (56.1-63.9)	61.4 (57.4-65.1)	56.8 (52.7-60.7)	57.7 (53.9-61.6)
	Diabetes mellitus type 2	43.0 (32.7-53.8)	43.0 (32.7-53.7)	36.5 (27.1-46.2)	36.5 (27.3-46.4)
	Neoplasms	8.5 (6.7-10.3)	7.5 (6.0-9.2)	10.1 (8.0-12.3)	8.7 (6.9-10.4)
Mauritania	All causes	13.5 (12.1-14.8)	12.8 (11.5-14.2)	17.8 (16.1-19.5)	17.7 (16.0-19.5)
	Cardiovascular diseases	57.6 (53.2-62.0)	55.7 (51.4-59.8)	53.5 (48.9-58.1)	52.0 (47.5-56.3)
	Diabetes mellitus type 2	43.7 (33.5-54.1)	43.3 (33.1-53.7)	36.7 (27.4-46.2)	35.2 (26.2-44.7)
	Neoplasms	5.4 (4.1-6.9)	5.2 (4.1-6.5)	5.8 (4.4-7.4)	5.7 (4.4-7.0)
Mauritius	All causes	30.1 (27.7-32.3)	19.8 (17.9-21.6)	38.3 (35.8-40.9)	27.0 (24.8-29.2)
	Cardiovascular diseases	71.4 (67.0-75.4)	59.1 (55.0-63.2)	66.5 (61.9-70.8)	55.5 (50.8-60.2)
	Diabetes mellitus type 2	44.1 (33.3-54.9)	41.3 (31.6-51.6)	38.3 (28.4-48.5)	34.5 (25.9-44.0)
	Neoplasms	14.4 (12.0-16.6)	8.5 (7.2-9.8)	15.2 (12.7-17.8)	9.5 (8.1-11.0)
Mexico	All causes	12.6 (11.6-13.6)	12.2 (11.1-13.4)	17.4 (16.2-18.6)	16.9 (15.5-18.5)
	Cardiovascular diseases	54.4 (50.9-58.1)	50.6 (46.1-55.4)	51.8 (48.0-55.7)	48.7 (44.0-53.7)
	Diabetes mellitus type 2	41.6 (33.4-50.9)	41.6 (33.8-50.0)	34.6 (27.0-43.2)	33.7 (26.7-41.6)
	Neoplasms	4.1 (3.1-5.4)	4.4 (3.7-5.2)	4.5 (3.4-6.1)	4.7 (3.9-5.5)
Moldova	All causes	22.5 (20.6-24.4)	20.5 (18.5-22.4)	36.8 (34.3-39.2)	34.0 (31.3-36.8)
	Cardiovascular diseases	62.4 (58.4-66.4)	59.2 (54.9-63.5)	61.1 (56.8-65.1)	57.5 (52.9-62.1)
	Diabetes mellitus type 2	43.3 (34.0-53.5)	43.5 (33.6-53.9)	37.0 (28.2-46.6)	36.9 (27.9-46.6)
	Neoplasms	9.2 (7.3-11.2)	7.9 (6.7-9.3)	9.8 (7.8-11.9)	8.4 (7.1-9.8)
Mongolia	All causes	26.5 (24.3-28.9)	23.2 (21.3-25.1)	36.0 (33.5-38.8)	32.8 (30.5-35.2)
	Cardiovascular diseases	68.8 (65.2-72.5)	66.1 (62.1-70.0)	65.5 (61.5-69.5)	62.3 (58.4-66.4)
	Diabetes mellitus type 2	53.1 (42.8-63.1)	51.7 (41.7-61.7)	44.7 (35.2-54.2)	45.2 (36.0-54.4)
	Neoplasms	14.8 (10.3-19.4)	8.6 (5.7-11.6)	15.2 (10.6-20.0)	9.1 (6.0-12.1)
Montenegro	All causes	23.0 (20.9-25.1)	16.9 (15.2-18.6)	34.2 (31.6-36.9)	25.3 (23.2-27.7)
	Cardiovascular diseases	59.9 (56.0-63.6)	45.0 (41.5-48.7)	54.5 (50.3-58.8)	41.0 (37.4-44.9)
	Diabetes mellitus type 2	44.0 (34.5-54.0)	43.7 (34.6-53.1)	29.7 (22.4-37.9)	29.2 (22.1-36.9)
	Neoplasms	7.6 (6.0-9.5)	5.2 (4.1-6.6)	8.2 (6.5-10.1)	5.7 (4.6-7.1)
Morocco	All causes	25.7 (23.2-28.2)	21.1 (18.6-23.6)	36.1 (33.1-39.0)	31.4 (28.3-34.7)
	Cardiovascular diseases	63.4 (59.0-68.0)	55.0 (50.1-60.4)	58.5 (53.9-63.1)	50.6 (45.7-55.9)

	Diabetes mellitus type 2	43.6 (33.4-54.5)	39.2 (29.2-50.1)	36.2 (26.8-45.9)	31.8 (23.1-41.4)
	Neoplasms	5.9 (4.7-7.1)	4.2 (3.4-5.2)	6.5 (5.2-7.8)	4.8 (3.9-5.8)
Mozambique	All causes	13.3 (11.4-15.3)	10.5 (9.5-11.6)	17.4 (14.7-20.1)	14.6 (13.1-16.2)
	Cardiovascular diseases	57.4 (52.2-62.5)	54.3 (49.9-58.4)	53.0 (47.7-58.4)	50.3 (45.5-54.8)
	Diabetes mellitus type 2	41.9 (31.5-52.4)	40.5 (30.0-51.2)	34.9 (25.7-44.5)	34.1 (24.7-43.7)
	Neoplasms	6.7 (5.5-7.9)	6.4 (5.0-7.8)	7.6 (6.4-9.0)	7.1 (5.6-8.6)
Myanmar	All causes	15.0 (13.7-16.4)	12.0 (10.9-13.1)	18.5 (16.9-20.2)	15.6 (14.4-17.0)
	Cardiovascular diseases	66.1 (61.9-70.1)	56.0 (52.0-59.8)	61.2 (57.0-65.4)	52.6 (48.6-56.5)
	Diabetes mellitus type 2	39.7 (28.8-50.8)	35.3 (24.7-46.2)	33.0 (23.5-43.1)	28.8 (19.8-38.5)
	Neoplasms	13.5 (10.6-16.1)	9.1 (7.6-10.8)	14.5 (11.7-17.1)	10.2 (8.4-12.1)
Namibia	All causes	13.0 (11.6-14.3)	9.2 (8.1-10.3)	18.1 (16.4-19.7)	14.4 (12.9-16.0)
	Cardiovascular diseases	53.8 (49.8-57.7)	50.3 (46.2-54.3)	49.9 (45.9-53.9)	46.8 (42.8-50.8)
	Diabetes mellitus type 2	43.1 (33.2-53.3)	40.9 (31.1-50.8)	37.0 (27.9-46.6)	33.8 (25.0-43.0)
	Neoplasms	5.8 (4.7-6.9)	5.0 (4.0-6.1)	6.2 (5.1-7.3)	5.4 (4.4-6.5)
Nepal	All causes	11.6 (10.2-13.0)	14.0 (12.3-15.7)	14.5 (12.8-16.5)	18.9 (16.8-20.9)
	Cardiovascular diseases	57.4 (52.7-62.5)	57.1 (52.3-61.5)	53.2 (48.6-58.4)	53.3 (48.8-57.6)
	Diabetes mellitus type 2	39.3 (28.7-50.2)	35.9 (25.9-46.4)	31.1 (22.0-40.4)	28.1 (19.8-37.0)
	Neoplasms	8.1 (6.3-9.9)	8.4 (6.8-10.0)	8.6 (6.7-10.5)	8.9 (7.3-10.6)
Netherlands	All causes	14.4 (12.8-15.9)	7.8 (7.0-8.6)	21.9 (20.1-23.8)	12.8 (11.8-13.9)
	Cardiovascular diseases	52.5 (48.5-56.6)	41.5 (38.6-44.8)	48.3 (44.4-52.4)	39.1 (35.9-42.4)
	Diabetes mellitus type 2	41.3 (33.0-50.3)	43.3 (35.0-52.1)	30.6 (23.6-38.4)	29.4 (22.6-36.9)
	Neoplasms	5.8 (4.6-7.1)	5.5 (4.1-7.2)	6.2 (5.0-7.6)	6.0 (4.5-7.6)
New Zealand	All causes	16.0 (14.3-17.7)	9.0 (8.0-10.0)	25.5 (23.6-27.6)	17.9 (16.5-19.3)
	Cardiovascular diseases	53.6 (49.7-57.6)	46.6 (43.3-49.7)	50.7 (46.7-54.9)	44.4 (40.8-47.9)
	Diabetes mellitus type 2	41.7 (33.0-50.6)	42.2 (33.5-51.2)	34.9 (26.9-43.2)	34.0 (26.5-41.9)
	Neoplasms	8.1 (6.6-9.7)	7.6 (6.1-9.1)	8.6 (7.1-10.3)	8.3 (6.7-10.0)
Nicaragua	All causes	14.6 (13.3-16.0)	12.1 (10.9-13.4)	23.4 (21.7-25.2)	20.3 (18.6-22.0)
	Cardiovascular diseases	57.5 (53.8-61.2)	54.1 (50.3-58.1)	54.1 (50.1-57.9)	52.2 (48.0-56.3)
	Diabetes mellitus type 2	42.2 (31.9-52.7)	40.6 (30.5-51.0)	35.4 (26.2-45.2)	34.2 (25.2-44.1)

	Neoplasms	6.0 (4.2-8.5)	5.8 (4.6-7.4)	7.0 (4.8-9.8)	6.5 (5.2-8.2)
Niger	All causes	10.3 (8.6-11.6)	9.9 (8.6-11.2)	14.0 (11.8-15.9)	13.4 (11.6-15.1)
	Cardiovascular diseases	54.3 (49.7-58.9)	52.3 (47.7-56.8)	50.2 (45.6-55.0)	48.3 (43.7-52.8)
	Diabetes mellitus type 2	42.6 (32.0-53.2)	40.7 (30.4-51.3)	35.1 (26.0-44.9)	33.1 (24.1-42.7)
	Neoplasms	4.7 (1.7-6.9)	4.9 (2.6-6.7)	5.1 (1.9-7.4)	5.3 (2.8-7.4)
Nigeria	All causes	7.7 (6.7-8.8)	6.6 (5.5-7.7)	11.7 (10.4-13.3)	10.6 (9.1-12.3)
	Cardiovascular diseases	46.9 (42.5-51.8)	45.5 (41.0-50.3)	44.0 (39.7-49.0)	42.9 (38.5-47.6)
	Diabetes mellitus type 2	38.9 (29.3-49.1)	36.3 (26.7-46.8)	31.0 (22.9-40.1)	28.4 (20.4-37.4)
	Neoplasms	4.6 (3.5-5.8)	3.9 (3.1-5.0)	5.0 (3.9-6.3)	4.3 (3.4-5.4)
North Korea	All causes	19.0 (16.6-21.2)	18.7 (16.9-20.5)	23.6 (19.9-26.4)	24.5 (21.8-26.8)
	Cardiovascular diseases	62.9 (57.8-67.6)	58.0 (53.7-62.2)	56.4 (51.2-61.6)	53.5 (49.2-58.1)
	Diabetes mellitus type 2	40.3 (29.9-51.1)	40.9 (30.3-51.8)	32.3 (23.2-42.0)	33.2 (24.0-43.3)
	Neoplasms	15.9 (11.7-19.5)	11.1 (8.0-14.1)	16.5 (12.5-20.3)	12.6 (9.4-15.9)
Northern Mariana Islands	All causes	17.3 (15.7-18.8)	17.0 (15.5-18.4)	22.1 (20.4-23.9)	22.0 (20.5-23.6)
	Cardiovascular diseases	54.7 (50.7-58.7)	57.4 (54.0-61.0)	52.1 (48.0-56.2)	54.6 (50.9-58.3)
	Diabetes mellitus type 2	41.0 (31.9-51.1)	40.8 (31.1-51.4)	32.4 (24.5-41.5)	32.6 (24.3-41.8)
	Neoplasms	6.2 (5.1-7.6)	5.7 (4.5-7.1)	7.4 (6.0-9.0)	6.4 (5.1-7.9)
Norway	All causes	16.8 (15.1-18.5)	8.5 (7.7-9.3)	26.3 (24.3-28.2)	15.0 (13.8-16.3)
	Cardiovascular diseases	58.0 (53.9-61.9)	45.5 (42.3-48.7)	53.3 (49.0-57.4)	42.5 (39.0-45.9)
	Diabetes mellitus type 2	48.3 (40.0-56.9)	51.6 (44.0-59.5)	35.9 (28.8-43.7)	35.8 (29.1-43.0)
	Neoplasms	6.9 (5.9-8.0)	6.0 (4.8-7.4)	7.3 (6.2-8.5)	6.4 (5.1-7.9)
Oman	All causes	22.9 (20.5-25.3)	18.1 (16.1-20.2)	32.5 (29.8-35.0)	27.9 (25.5-30.2)
	Cardiovascular diseases	61.0 (56.5-65.7)	55.3 (50.9-59.6)	57.3 (52.6-62.0)	52.8 (48.1-57.3)
	Diabetes mellitus type 2	41.1 (31.3-51.4)	37.0 (28.2-46.7)	34.1 (25.3-43.7)	29.8 (22.3-38.5)
	Neoplasms	6.9 (4.5-10.0)	4.2 (3.1-5.8)	7.2 (4.8-10.4)	4.7 (3.5-6.4)
Pakistan	All causes	17.6 (15.7-19.6)	21.8 (19.8-23.7)	22.9 (20.7-25.2)	28.2 (26.2-30.3)
	Cardiovascular diseases	60.4 (56.4-64.5)	65.6 (62.1-69.0)	55.4 (51.4-59.4)	60.4 (56.7-64.0)
	Diabetes mellitus type 2	45.0 (34.6-55.7)	43.3 (33.4-53.9)	35.0 (26.3-44.5)	34.2 (25.6-43.2)
	Neoplasms	7.0 (5.4-8.7)	7.2 (5.4-8.9)	7.3 (5.7-9.1)	7.6 (5.9-9.2)

Palestine	All causes	22.7 (20.3-25.1)	18.4 (16.4-20.5)	32.7 (30.3-35.3)	29.0 (26.6-31.3)
	Cardiovascular diseases	61.5 (57.3-65.9)	56.9 (52.7-61.3)	56.9 (52.7-61.4)	53.2 (48.8-57.7)
	Diabetes mellitus type 2	42.6 (32.5-53.1)	41.7 (31.8-52.1)	35.8 (26.9-45.5)	34.2 (25.5-43.7)
	Neoplasms	8.3 (6.8-9.8)	7.7 (6.5-9.1)	8.5 (6.9-10.0)	7.9 (6.6-9.3)
Panama	All causes	14.8 (13.4-16.1)	10.7 (9.8-11.7)	24.5 (22.7-26.2)	17.6 (16.2-19.0)
	Cardiovascular diseases	54.7 (50.9-58.4)	47.2 (43.9-50.8)	52.6 (48.6-56.6)	45.6 (42.0-49.4)
	Diabetes mellitus type 2	39.5 (30.0-49.9)	38.2 (29.5-47.6)	30.4 (22.5-39.0)	29.7 (22.2-38.4)
	Neoplasms	7.2 (5.5-9.1)	6.6 (5.4-7.9)	7.9 (6.1-10.1)	7.2 (5.9-8.6)
Papua New Guinea	All causes	18.7 (17.2-20.3)	21.4 (19.7-23.0)	20.8 (19.2-22.4)	23.7 (22.0-25.4)
	Cardiovascular diseases	60.3 (56.1-64.4)	63.3 (59.2-67.2)	57.8 (53.7-61.9)	60.1 (56.0-64.0)
	Diabetes mellitus type 2	45.1 (34.5-55.9)	44.4 (34.1-55.0)	39.2 (29.4-49.5)	38.5 (28.9-48.4)
	Neoplasms	7.5 (5.9-9.2)	7.4 (5.8-9.1)	9.1 (7.0-11.1)	8.6 (6.7-10.5)
Paraguay	All causes	16.5 (14.9-18.0)	13.8 (12.4-15.2)	24.9 (23.1-26.7)	20.9 (19.2-22.5)
	Cardiovascular diseases	53.8 (50.1-57.5)	51.2 (47.2-55.2)	49.9 (46.0-53.4)	48.7 (44.7-52.6)
	Diabetes mellitus type 2	39.7 (30.7-49.8)	38.1 (29.1-48.0)	30.9 (23.1-39.8)	30.8 (23.0-39.5)
	Neoplasms	5.9 (4.4-7.6)	6.1 (5.1-7.2)	6.8 (5.0-8.8)	6.8 (5.7-8.0)
Peru	All causes	11.9 (10.8-13.0)	8.6 (7.7-9.4)	18.7 (17.3-20.2)	14.0 (12.8-15.3)
	Cardiovascular diseases	59.7 (56.1-63.2)	51.5 (47.9-55.0)	56.5 (52.5-60.3)	50.8 (47.0-54.6)
	Diabetes mellitus type 2	41.5 (31.6-51.9)	38.4 (28.9-48.9)	33.9 (25.3-43.3)	31.3 (22.9-40.6)
	Neoplasms	8.2 (5.2-11.6)	6.2 (4.3-8.5)	9.1 (5.8-13.0)	7.1 (4.9-9.8)
Philippines	All causes	17.1 (15.7-18.4)	18.7 (17.1-20.3)	23.2 (21.6-24.8)	23.7 (21.9-25.7)
	Cardiovascular diseases	65.2 (61.1-69.2)	57.5 (53.5-61.7)	60.0 (55.8-64.2)	53.7 (49.3-58.3)
	Diabetes mellitus type 2	36.5 (27.0-47.0)	34.7 (25.5-45.0)	28.2 (20.2-37.4)	28.1 (20.1-37.0)
	Neoplasms	7.3 (6.3-8.4)	7.1 (6.0-8.4)	8.2 (6.9-9.4)	7.8 (6.5-9.2)
Poland	All causes	23.1 (21.2-24.9)	14.3 (13.1-15.6)	34.0 (31.8-36.1)	24.0 (22.3-25.7)
	Cardiovascular diseases	60.5 (56.9-63.9)	52.0 (48.9-55.2)	57.5 (53.5-61.2)	50.1 (46.5-53.6)
	Diabetes mellitus type 2	48.9 (39.5-58.7)	47.4 (38.3-56.7)	40.8 (32.1-49.9)	35.9 (28.3-44.2)
	Neoplasms	9.0 (7.2-10.7)	8.5 (6.9-10.3)	9.5 (7.6-11.2)	8.9 (7.2-10.7)
Portugal	All causes	14.5 (13.1-16.1)	9.3 (8.4-10.3)	21.8 (20.0-24.0)	14.5 (13.4-15.7)

	Cardiovascular diseases	45.2 (41.4-50.0)	41.0 (38.0-44.3)	40.4 (36.7-45.0)	37.1 (33.9-40.5)
	Diabetes mellitus type 2	40.3 (31.3-50.0)	42.4 (33.6-51.6)	31.8 (23.8-40.5)	30.1 (23.1-37.9)
	Neoplasms	7.4 (5.8-9.4)	7.4 (6.3-8.6)	7.7 (6.2-9.9)	7.6 (6.4-8.7)
Puerto Rico	All causes	15.0 (13.7-16.2)	9.9 (8.9-11.0)	22.6 (20.9-24.2)	14.0 (12.8-15.2)
	Cardiovascular diseases	56.8 (52.6-60.8)	45.3 (41.6-49.3)	54.4 (50.0-58.5)	43.5 (39.5-47.4)
	Diabetes mellitus type 2	43.1 (34.0-53.1)	41.9 (32.9-51.6)	34.2 (26.3-43.0)	32.7 (24.8-41.1)
	Neoplasms	7.2 (6.1-8.5)	5.5 (4.4-6.9)	7.9 (6.5-9.4)	5.9 (4.7-7.3)
Qatar	All causes	19.0 (16.9-21.0)	11.8 (10.3-13.4)	25.0 (22.6-27.3)	18.1 (16.1-20.3)
	Cardiovascular diseases	48.2 (43.3-52.7)	41.8 (36.8-46.8)	44.6 (39.9-48.9)	39.1 (34.0-44.0)
	Diabetes mellitus type 2	32.8 (24.6-41.4)	33.3 (25.4-42.0)	25.8 (18.8-33.5)	25.0 (18.4-32.4)
	Neoplasms	4.6 (3.7-5.6)	3.5 (2.9-4.2)	4.9 (3.9-6.0)	3.8 (3.1-4.6)
Romania	All causes	25.0 (23.1-27.0)	17.0 (15.5-18.6)	38.2 (35.8-40.7)	27.2 (25.1-29.4)
	Cardiovascular diseases	63.0 (59.5-66.5)	50.1 (46.7-53.7)	59.0 (55.2-62.8)	46.7 (43.2-50.9)
	Diabetes mellitus type 2	47.5 (38.2-56.9)	46.7 (38.0-56.0)	39.2 (30.5-48.5)	36.7 (29.0-45.1)
	Neoplasms	11.6 (9.6-13.4)	7.0 (5.7-8.4)	12.4 (10.2-14.4)	7.3 (6.0-8.7)
Russian Federation	All causes	22.7 (20.9-24.5)	18.5 (16.9-20.1)	35.0 (32.8-37.3)	29.9 (27.7-32.0)
	Cardiovascular diseases	60.8 (57.0-64.6)	53.6 (50.1-57.2)	56.8 (52.9-60.8)	51.9 (48.1-55.6)
	Diabetes mellitus type 2	50.3 (41.4-59.7)	45.4 (36.5-54.9)	41.3 (33.0-50.1)	36.5 (28.5-45.3)
	Neoplasms	10.7 (8.1-13.7)	8.0 (6.6-9.3)	11.2 (8.5-14.3)	8.5 (7.1-10.0)
Rwanda	All causes	7.7 (6.4-9.4)	6.8 (5.8-7.9)	9.8 (7.8-12.2)	10.0 (8.0-12.0)
	Cardiovascular diseases	42.3 (35.1-50.5)	40.9 (34.6-47.3)	39.0 (31.4-47.8)	38.7 (31.4-46.2)
	Diabetes mellitus type 2	33.4 (24.3-43.6)	33.5 (24.3-43.6)	27.8 (19.9-37.2)	27.0 (19.1-35.7)
	Neoplasms	3.4 (2.3-4.8)	2.8 (2.1-3.6)	3.8 (2.6-5.3)	3.3 (2.5-4.1)
Saint Lucia	All causes	16.7 (15.2-18.2)	11.7 (10.4-13.1)	22.6 (20.8-24.1)	15.8 (14.3-17.2)
	Cardiovascular diseases	47.1 (43.6-50.9)	38.5 (35.2-42.2)	44.8 (41.4-48.2)	36.3 (33.0-39.7)
	Diabetes mellitus type 2	39.9 (30.4-50.2)	39.6 (29.8-49.8)	32.9 (24.5-42.3)	32.0 (23.6-40.9)
	Neoplasms	5.0 (3.9-6.3)	4.2 (3.5-5.0)	5.7 (4.4-7.4)	4.5 (3.7-5.4)
Saint Vincent and the Grenadines	All causes	17.4 (15.9-19.1)	14.1 (12.7-15.8)	24.5 (22.8-26.4)	19.5 (17.8-21.4)
	Cardiovascular diseases	51.3 (47.6-55.0)	44.4 (40.6-48.6)	48.8 (45.0-52.6)	42.8 (39.1-46.9)

	Diabetes mellitus type 2	40.6 (30.6-51.1)	40.4 (30.8-50.7)	32.8 (24.3-42.2)	33.4 (24.8-42.7)
	Neoplasms	4.8 (3.9-5.9)	4.1 (3.5-4.9)	5.7 (4.6-7.1)	4.5 (3.7-5.3)
Samoa	All causes	18.5 (16.4-20.6)	20.1 (18.0-22.3)	22.3 (20.1-24.5)	25.8 (23.8-27.8)
	Cardiovascular diseases	53.8 (48.7-58.9)	55.1 (50.5-59.6)	49.0 (44.4-53.7)	52.2 (48.1-56.3)
	Diabetes mellitus type 2	43.3 (33.1-53.7)	41.7 (31.8-52.2)	36.0 (27.1-45.5)	34.0 (25.4-43.3)
	Neoplasms	5.2 (4.3-6.3)	5.6 (4.6-6.6)	5.9 (4.8-7.1)	6.7 (5.4-8.3)
Sao Tome and Principe	All causes	11.3 (10.2-12.6)	12.4 (11.2-13.8)	15.8 (14.3-17.3)	16.5 (14.9-18.4)
	Cardiovascular diseases	53.1 (49.0-57.6)	52.5 (48.3-56.9)	49.1 (44.9-53.9)	48.9 (44.6-53.2)
	Diabetes mellitus type 2	41.7 (31.4-52.1)	40.5 (30.4-51.0)	33.6 (24.7-43.2)	32.7 (23.9-42.0)
	Neoplasms	6.5 (5.1-8.3)	6.9 (5.3-8.7)	7.2 (5.5-9.3)	7.6 (5.9-9.6)
Saudi Arabia	All causes	16.3 (14.4-18.5)	17.4 (15.7-19.3)	23.9 (21.6-26.8)	25.6 (23.6-27.8)
	Cardiovascular diseases	55.7 (51.4-60.1)	58.1 (53.5-62.8)	51.9 (47.8-56.1)	54.3 (49.6-59.1)
	Diabetes mellitus type 2	43.2 (33.4-53.6)	44.0 (34.2-54.2)	34.7 (26.1-44.2)	34.8 (26.6-44.1)
	Neoplasms	6.3 (5.0-7.9)	6.1 (5.1-7.2)	6.6 (5.1-8.6)	6.5 (5.4-7.7)
Senegal	All causes	10.7 (9.6-11.7)	11.7 (10.5-12.9)	14.3 (12.8-15.8)	15.5 (14.0-17.1)
	Cardiovascular diseases	56.5 (52.1-61.1)	54.8 (50.4-59.0)	52.5 (47.8-57.4)	51.0 (46.4-55.3)
	Diabetes mellitus type 2	42.2 (31.9-52.6)	40.4 (29.9-50.9)	34.5 (25.5-43.9)	32.8 (23.9-42.2)
	Neoplasms	6.3 (4.9-7.8)	5.3 (4.0-6.6)	6.8 (5.3-8.4)	5.7 (4.3-7.1)
Serbia	All causes	25.7 (23.6-28.0)	18.0 (16.3-19.6)	37.4 (35.0-40.0)	27.8 (25.8-29.9)
	Cardiovascular diseases	63.8 (60.3-67.3)	50.0 (46.8-53.2)	59.3 (55.5-63.0)	46.1 (42.5-49.6)
	Diabetes mellitus type 2	42.2 (33.0-52.4)	41.7 (32.7-51.5)	34.2 (25.8-43.3)	31.4 (23.7-40.0)
	Neoplasms	10.0 (8.2-11.9)	7.6 (6.0-9.3)	10.6 (8.8-12.5)	8.0 (6.4-9.7)
Seychelles	All causes	20.0 (18.0-22.1)	14.2 (12.9-15.8)	26.0 (23.1-29.1)	19.0 (16.7-21.7)
	Cardiovascular diseases	60.4 (54.9-66.0)	49.3 (44.2-55.0)	57.1 (50.7-64.1)	47.6 (41.3-55.2)
	Diabetes mellitus type 2	37.6 (27.7-48.4)	36.0 (26.6-47.0)	29.4 (20.8-39.0)	27.2 (19.4-36.1)
	Neoplasms	9.9 (8.3-11.5)	7.2 (5.8-8.8)	10.5 (8.7-12.1)	7.8 (6.2-9.4)
Sierra Leone	All causes	11.0 (9.0-12.6)	12.2 (10.9-13.5)	14.7 (12.2-16.8)	16.3 (14.7-17.9)
	Cardiovascular diseases	54.2 (49.5-59.2)	54.6 (50.0-59.2)	50.4 (45.7-55.5)	50.6 (46.1-55.2)
	Diabetes mellitus type 2	38.7 (28.5-49.2)	37.8 (27.6-48.2)	31.3 (22.7-41.0)	30.7 (22.1-40.0)

	Neoplasms	5.4 (1.8-7.6)	6.1 (4.7-7.5)	5.9 (2.0-8.3)	6.6 (5.1-8.3)
Singapore	All causes	20.5 (18.6-22.2)	11.7 (10.4-13.2)	28.2 (26.4-30.1)	19.6 (17.8-21.4)
	Cardiovascular diseases	62.2 (58.2-66.1)	53.5 (48.8-58.1)	59.0 (54.8-63.1)	51.4 (46.6-56.5)
	Diabetes mellitus type 2	37.4 (28.0-47.5)	38.2 (29.2-48.4)	30.4 (22.2-39.7)	27.4 (20.2-35.6)
	Neoplasms	13.4 (11.4-15.4)	8.8 (7.2-10.5)	14.4 (12.1-16.6)	9.7 (7.9-11.5)
Slovakia	All causes	28.1 (26.1-30.1)	17.4 (15.7-19.2)	41.4 (39.2-43.4)	30.5 (28.4-32.7)
	Cardiovascular diseases	72.0 (68.6-74.9)	56.9 (53.5-60.4)	69.3 (65.5-72.8)	56.9 (52.9-60.9)
	Diabetes mellitus type 2	45.6 (36.5-55.1)	46.8 (38.0-56.1)	36.6 (28.6-45.3)	35.3 (27.9-43.5)
	Neoplasms	12.7 (10.8-14.6)	9.5 (7.9-11.1)	13.4 (11.4-15.4)	10.1 (8.5-11.8)
Slovenia	All causes	18.1 (16.6-19.6)	10.0 (9.0-11.0)	27.7 (25.7-29.6)	18.2 (16.7-19.9)
	Cardiovascular diseases	57.4 (53.7-61.0)	46.1 (42.6-50.0)	51.6 (47.6-55.6)	43.2 (39.2-47.5)
	Diabetes mellitus type 2	43.3 (34.9-52.5)	43.9 (35.5-53.0)	32.5 (25.3-40.7)	31.2 (24.4-38.7)
	Neoplasms	12.4 (10.5-14.2)	6.8 (5.6-8.2)	13.0 (10.9-15.0)	7.3 (5.9-8.7)
Solomon Islands	All causes	22.8 (21.0-24.5)	22.5 (20.6-24.3)	26.9 (25.0-28.9)	26.6 (24.8-28.5)
	Cardiovascular diseases	63.9 (59.7-68.1)	63.0 (58.9-66.7)	60.7 (56.6-64.7)	59.0 (55.1-62.8)
	Diabetes mellitus type 2	44.4 (33.9-55.3)	42.2 (31.9-53.1)	38.5 (28.7-48.7)	36.3 (27.0-46.2)
	Neoplasms	7.4 (5.7-9.2)	6.7 (5.3-8.2)	8.9 (6.7-11.2)	7.8 (6.1-9.6)
Somalia	All causes	13.3 (10.1-15.9)	13.2 (11.8-14.8)	17.0 (13.7-20.0)	16.9 (15.0-19.0)
	Cardiovascular diseases	56.6 (51.2-61.7)	57.9 (53.0-62.2)	52.6 (47.2-57.9)	53.8 (48.6-58.5)
	Diabetes mellitus type 2	41.1 (30.8-51.6)	42.0 (31.6-52.5)	34.7 (25.5-44.5)	35.2 (26.1-44.7)
	Neoplasms	8.5 (6.3-10.7)	7.3 (5.5-9.3)	9.2 (6.9-11.6)	8.1 (6.1-10.2)
South Africa	All causes	9.6 (8.6-10.6)	7.9 (7.1-8.7)	13.7 (12.1-15.4)	12.4 (11.2-13.6)
	Cardiovascular diseases	49.8 (45.7-53.9)	47.2 (43.1-51.3)	44.9 (40.6-49.2)	43.4 (39.2-47.6)
	Diabetes mellitus type 2	38.0 (28.4-48.3)	37.9 (28.5-47.9)	31.4 (22.7-40.9)	31.6 (23.2-40.8)
	Neoplasms	7.5 (5.5-9.8)	7.4 (5.6-9.3)	7.7 (5.6-9.9)	7.6 (5.9-9.6)
South Korea	All causes	18.7 (17.0-20.6)	8.7 (7.7-9.7)	26.2 (24.1-28.6)	14.3 (13.0-15.8)
	Cardiovascular diseases	55.4 (51.2-59.6)	45.7 (41.6-49.7)	51.1 (47.0-55.4)	42.8 (38.5-47.2)
	Diabetes mellitus type 2	35.5 (25.5-46.0)	35.6 (27.2-45.2)	30.1 (21.4-39.8)	26.7 (19.8-34.9)
	Neoplasms	15.7 (10.4-20.9)	10.0 (8.1-11.9)	16.2 (11.0-21.4)	10.8 (8.6-12.9)

South Sudan	All causes	10.8 (8.5-13.0)	10.1 (8.8-11.4)	14.8 (12.2-17.6)	14.0 (12.3-15.8)
	Cardiovascular diseases	53.0 (47.3-58.7)	53.6 (49.3-58.2)	49.3 (43.6-55.4)	50.1 (45.2-55.0)
	Diabetes mellitus type 2	37.5 (27.8-48.0)	38.1 (28.2-48.6)	31.4 (22.8-41.1)	31.7 (23.0-41.2)
	Neoplasms	8.1 (6.1-10.2)	6.6 (5.1-8.3)	8.6 (6.5-11.0)	7.3 (5.6-9.1)
Spain	All causes	11.3 (10.0-12.7)	7.7 (6.8-8.6)	18.0 (16.3-20.1)	12.7 (11.5-14.2)
	Cardiovascular diseases	43.7 (39.5-48.8)	39.3 (35.8-42.9)	40.0 (35.8-45.1)	35.6 (31.9-39.6)
	Diabetes mellitus type 2	39.6 (31.3-48.5)	41.7 (33.5-50.6)	30.6 (23.5-38.5)	28.1 (21.5-35.3)
	Neoplasms	6.3 (5.2-7.4)	6.8 (5.6-8.2)	6.6 (5.4-7.7)	7.1 (5.8-8.6)
Sri Lanka	All causes	19.3 (17.7-21.0)	16.3 (14.8-17.8)	26.1 (24.3-28.1)	23.8 (22.1-25.4)
	Cardiovascular diseases	65.9 (62.2-69.5)	59.2 (55.2-62.7)	61.3 (57.4-64.9)	56.4 (52.2-60.3)
	Diabetes mellitus type 2	39.3 (29.1-49.7)	37.8 (27.9-48.1)	30.9 (22.4-39.9)	29.5 (21.3-38.3)
	Neoplasms	10.8 (9.1-12.5)	7.8 (6.5-9.2)	11.4 (9.4-13.3)	8.7 (7.2-10.1)
Sudan	All causes	26.3 (23.0-29.1)	22.8 (20.2-25.4)	35.4 (31.8-38.9)	33.2 (29.9-36.3)
	Cardiovascular diseases	64.3 (59.7-68.9)	60.4 (55.6-65.2)	59.1 (54.3-63.8)	55.5 (50.6-60.5)
	Diabetes mellitus type 2	44.9 (34.4-55.9)	44.0 (33.7-54.8)	37.4 (28.1-47.3)	36.5 (27.4-46.4)
	Neoplasms	8.5 (5.8-12.1)	8.1 (6.0-10.9)	8.7 (5.9-12.2)	8.3 (6.1-11.2)
Suriname	All causes	18.6 (16.9-20.3)	14.3 (12.9-16.0)	25.9 (24.0-27.8)	19.0 (17.4-20.8)
	Cardiovascular diseases	55.2 (51.0-59.8)	47.8 (43.5-52.9)	52.0 (47.8-56.2)	44.0 (40.1-48.4)
	Diabetes mellitus type 2	40.5 (30.9-50.9)	39.8 (30.5-50.0)	32.9 (24.4-42.2)	32.3 (24.0-41.5)
	Neoplasms	6.4 (5.4-7.5)	6.0 (5.1-7.2)	7.2 (6.1-8.5)	6.6 (5.6-7.8)
Swaziland	All causes	12.0 (10.9-13.1)	10.1 (8.9-11.2)	16.1 (14.6-17.6)	14.7 (13.2-16.3)
	Cardiovascular diseases	47.6 (43.4-52.0)	49.3 (44.9-53.8)	44.4 (40.3-48.6)	45.7 (41.3-50.1)
	Diabetes mellitus type 2	36.6 (27.1-46.8)	33.7 (24.4-43.8)	31.2 (22.8-40.5)	28.5 (20.1-37.6)
	Neoplasms	7.1 (5.2-9.2)	6.3 (4.7-8.1)	7.3 (5.5-9.3)	6.5 (5.0-8.2)
Sweden	All causes	16.8 (15.2-18.5)	9.7 (8.9-10.7)	28.5 (26.4-30.6)	18.0 (16.6-19.5)
	Cardiovascular diseases	57.3 (53.3-61.0)	46.7 (43.4-50.2)	55.4 (51.0-59.5)	44.9 (41.0-48.6)
	Diabetes mellitus type 2	46.8 (38.5-55.7)	48.3 (40.6-56.7)	35.3 (28.0-43.2)	34.3 (27.5-41.8)
	Neoplasms	6.1 (5.1-7.2)	5.4 (4.3-6.7)	6.5 (5.4-7.6)	5.8 (4.6-7.1)
Switzerland	All causes	12.7 (11.4-14.0)	7.7 (6.9-8.6)	22.5 (20.9-24.3)	16.0 (14.5-17.6)

	Cardiovascular diseases	52.2 (48.4-56.0)	44.6 (41.0-48.2)	49.4 (45.6-53.3)	44.0 (39.9-48.1)
	Diabetes mellitus type 2	40.6 (32.2-49.6)	44.7 (36.1-53.7)	29.8 (22.7-37.7)	30.2 (23.3-37.8)
	Neoplasms	4.8 (3.9-5.8)	5.7 (4.5-7.1)	4.9 (3.9-5.9)	6.0 (4.7-7.4)
Syria	All causes	29.5 (26.6-32.3)	19.6 (17.3-21.9)	40.6 (37.4-43.6)	31.8 (28.8-34.7)
	Cardiovascular diseases	66.1 (61.5-70.8)	64.9 (59.6-70.3)	61.8 (57.0-66.7)	61.1 (55.4-66.7)
	Diabetes mellitus type 2	45.7 (35.1-56.7)	45.4 (35.0-56.2)	35.8 (26.8-45.2)	34.2 (25.5-43.7)
	Neoplasms	5.7 (4.5-7.0)	5.5 (4.5-6.7)	6.0 (4.7-7.5)	5.9 (4.8-7.1)
Taiwan	All causes	14.9 (13.6-16.2)	9.5 (8.5-10.5)	21.0 (19.2-22.8)	14.1 (12.9-15.3)
	Cardiovascular diseases	50.3 (46.3-54.4)	42.5 (38.7-46.4)	48.2 (43.9-52.6)	43.2 (38.9-47.7)
	Diabetes mellitus type 2	37.3 (28.1-47.6)	36.7 (27.9-46.4)	29.9 (21.8-39.0)	27.7 (20.1-36.0)
	Neoplasms	9.1 (7.6-10.7)	7.2 (6.0-8.6)	10.5 (8.6-12.5)	8.3 (6.8-9.8)
Tajikistan	All causes	24.2 (22.1-26.2)	25.9 (23.5-28.0)	35.4 (33.1-37.9)	37.7 (35.1-40.2)
	Cardiovascular diseases	64.7 (61.1-68.3)	62.3 (58.2-66.1)	61.1 (57.1-65.1)	59.7 (55.3-63.8)
	Diabetes mellitus type 2	47.2 (36.9-57.8)	47.0 (36.4-57.7)	41.6 (31.8-51.8)	42.2 (32.2-52.5)
	Neoplasms	15.8 (11.8-20.1)	9.8 (6.7-13.6)	15.9 (11.6-20.1)	10.8 (7.5-14.7)
Tanzania	All causes	8.4 (6.9-9.8)	9.6 (8.4-10.9)	12.4 (10.4-14.6)	14.0 (11.9-16.1)
	Cardiovascular diseases	51.0 (45.5-57.2)	51.0 (46.0-56.3)	47.2 (41.4-54.1)	47.9 (42.3-54.0)
	Diabetes mellitus type 2	33.5 (23.4-44.0)	32.5 (23.0-42.9)	27.6 (18.9-37.0)	26.2 (18.0-35.3)
	Neoplasms	6.1 (4.9-7.4)	4.9 (4.1-5.8)	7.1 (5.7-8.5)	5.7 (4.7-6.8)
Thailand	All causes	15.1 (13.8-16.5)	10.1 (9.1-11.0)	21.1 (19.5-22.7)	14.8 (13.7-16.1)
	Cardiovascular diseases	61.5 (57.2-65.6)	53.8 (49.8-57.6)	57.5 (53.2-61.7)	51.6 (47.6-55.4)
	Diabetes mellitus type 2	36.0 (25.9-46.8)	34.7 (25.1-45.4)	30.2 (21.3-39.8)	27.8 (19.5-37.2)
	Neoplasms	8.6 (7.3-9.9)	5.9 (4.9-7.0)	9.2 (7.8-10.7)	6.5 (5.4-7.7)
The Bahamas	All causes	14.0 (12.8-15.3)	11.2 (10.1-12.2)	19.1 (17.5-20.9)	15.2 (13.8-16.9)
	Cardiovascular diseases	43.4 (39.7-47.0)	37.7 (34.5-41.2)	41.6 (37.8-45.7)	35.6 (32.0-39.9)
	Diabetes mellitus type 2	43.0 (34.2-52.5)	40.8 (32.1-50.0)	35.2 (27.3-44.2)	32.3 (24.6-40.7)
	Neoplasms	5.4 (4.6-6.2)	4.1 (3.5-4.9)	5.9 (5.0-7.0)	4.6 (3.9-5.4)
The Gambia	All causes	11.8 (10.4-13.3)	13.6 (12.1-15.2)	16.2 (14.4-18.1)	18.5 (16.7-20.5)
	Cardiovascular diseases	56.2 (51.5-61.1)	58.1 (53.5-62.6)	52.1 (47.2-57.2)	53.8 (49.3-58.5)

	Diabetes mellitus type 2	40.6 (30.1-51.4)	40.1 (29.6-50.9)	33.6 (24.4-43.4)	33.0 (23.8-42.6)
	Neoplasms	4.5 (3.6-5.4)	3.9 (2.9-5.4)	5.0 (4.1-6.1)	4.5 (3.3-6.0)
Timor-Leste	All causes	20.5 (18.5-22.4)	18.9 (16.5-21.2)	27.3 (25.0-29.6)	25.6 (23.2-28.1)
	Cardiovascular diseases	65.9 (61.9-69.7)	60.8 (56.6-64.7)	60.8 (56.7-64.9)	56.0 (51.9-60.0)
	Diabetes mellitus type 2	40.5 (29.6-51.5)	39.8 (28.8-50.9)	32.9 (23.5-42.6)	32.1 (22.8-41.9)
	Neoplasms	12.3 (10.0-14.6)	9.0 (7.2-10.9)	13.0 (10.5-15.6)	9.8 (7.8-11.9)
Togo	All causes	11.0 (9.9-12.1)	11.7 (10.5-12.9)	15.3 (13.8-16.8)	16.2 (14.6-17.7)
	Cardiovascular diseases	54.8 (50.5-59.5)	56.4 (52.0-60.7)	51.1 (46.5-55.8)	52.3 (47.8-56.8)
	Diabetes mellitus type 2	41.8 (31.4-52.3)	40.8 (30.3-51.3)	34.3 (25.3-43.9)	33.3 (24.2-42.7)
	Neoplasms	5.1 (3.9-6.5)	5.7 (4.3-7.1)	5.5 (4.2-7.1)	6.2 (4.7-7.7)
Tonga	All causes	18.0 (16.4-19.5)	18.1 (16.5-19.8)	22.1 (20.6-23.6)	21.9 (20.3-23.4)
	Cardiovascular diseases	61.6 (57.8-65.0)	61.6 (58.1-64.9)	58.8 (54.8-62.5)	58.3 (54.6-61.9)
	Diabetes mellitus type 2	41.1 (31.1-51.6)	40.8 (31.1-51.2)	34.6 (25.7-44.2)	34.0 (25.3-43.5)
	Neoplasms	6.3 (4.9-7.8)	5.5 (4.3-6.7)	7.7 (5.8-9.6)	6.5 (5.0-8.0)
Trinidad and Tobago	All causes	22.0 (19.8-24.5)	17.2 (15.4-19.3)	27.4 (25.0-30.4)	23.1 (20.9-25.7)
	Cardiovascular diseases	54.3 (49.5-59.6)	50.5 (45.7-55.4)	49.7 (45.1-55.1)	47.1 (42.4-52.1)
	Diabetes mellitus type 2	43.1 (33.4-53.3)	43.4 (33.9-53.3)	37.7 (28.8-47.2)	36.6 (28.2-45.6)
	Neoplasms	5.5 (4.6-6.4)	5.0 (4.0-6.2)	5.9 (5.0-7.0)	5.3 (4.3-6.5)
Tunisia	All causes	19.6 (17.0-22.1)	16.3 (14.1-18.5)	30.7 (27.4-34.1)	27.0 (24.1-30.0)
	Cardiovascular diseases	54.1 (48.7-59.7)	49.5 (44.5-54.9)	50.0 (44.7-55.3)	45.9 (40.8-51.4)
	Diabetes mellitus type 2	41.1 (30.9-51.8)	39.7 (29.7-50.4)	31.2 (23.0-40.1)	30.5 (22.2-39.6)
	Neoplasms	6.8 (5.4-8.2)	5.6 (4.4-7.0)	7.2 (5.7-8.7)	6.0 (4.8-7.4)
Turkey	All causes	18.3 (16.1-20.5)	11.5 (10.2-12.9)	25.8 (23.1-28.4)	18.8 (16.9-20.9)
	Cardiovascular diseases	53.9 (48.2-59.5)	44.4 (39.9-48.9)	50.1 (44.8-55.5)	42.1 (37.5-46.6)
	Diabetes mellitus type 2	37.9 (28.2-48.5)	36.8 (27.2-47.1)	31.8 (22.9-41.4)	29.2 (20.9-38.4)
	Neoplasms	5.4 (4.0-7.0)	4.3 (3.4-5.3)	5.7 (4.3-7.4)	4.6 (3.7-5.7)
Turkmenistan	All causes	30.6 (28.2-32.8)	28.8 (26.3-31.0)	42.9 (40.2-45.3)	40.6 (37.8-43.2)
	Cardiovascular diseases	68.2 (64.6-71.6)	61.8 (57.8-65.5)	66.0 (62.0-69.7)	60.3 (56.2-64.4)
	Diabetes mellitus type 2	48.7 (38.9-58.8)	49.4 (40.2-59.0)	42.7 (33.2-52.3)	44.0 (35.2-53.4)

	Neoplasms	16.7 (12.5-20.7)	8.2 (6.5-10.0)	17.0 (12.5-21.3)	9.3 (7.3-11.1)
Uganda	All causes	4.7 (3.6-5.9)	7.5 (6.5-8.5)	7.7 (6.0-9.8)	11.0 (9.4-12.8)
	Cardiovascular diseases	43.2 (36.2-51.1)	43.8 (38.6-48.8)	40.6 (33.3-48.7)	41.5 (35.9-47.3)
	Diabetes mellitus type 2	29.8 (21.1-39.7)	29.1 (20.0-39.4)	24.0 (16.6-32.7)	23.2 (15.7-32.1)
	Neoplasms	3.0 (2.4-3.8)	3.2 (2.5-4.3)	3.3 (2.6-4.1)	3.5 (2.7-4.6)
Ukraine	All causes	23.5 (21.6-25.4)	22.8 (20.7-24.7)	37.5 (35.1-39.9)	37.0 (34.0-39.8)
	Cardiovascular diseases	62.5 (58.8-66.2)	59.4 (54.9-63.7)	60.0 (56.0-63.9)	58.7 (53.8-63.2)
	Diabetes mellitus type 2	48.5 (38.8-58.4)	46.4 (36.6-56.5)	39.3 (30.5-48.7)	37.6 (28.9-47.0)
	Neoplasms	10.9 (8.4-13.7)	7.9 (6.6-9.3)	11.5 (9.0-14.4)	8.5 (7.1-10.0)
United Arab Emirates	All causes	16.1 (14.3-18.1)	16.0 (14.1-18.1)	21.7 (19.5-24.3)	21.7 (19.2-24.2)
	Cardiovascular diseases	48.0 (43.8-52.3)	51.6 (46.9-56.2)	44.7 (40.6-49.2)	47.8 (43.3-52.3)
	Diabetes mellitus type 2	37.8 (28.8-47.2)	37.4 (28.0-47.4)	30.8 (22.8-39.4)	30.4 (22.2-39.5)
	Neoplasms	4.1 (3.1-5.4)	4.7 (3.7-6.0)	4.5 (3.3-6.2)	5.0 (3.9-6.5)
United Kingdom	All causes	17.3 (15.5-19.1)	8.8 (7.9-9.7)	26.4 (24.4-28.5)	15.0 (13.9-16.2)
	Cardiovascular diseases	56.3 (52.0-60.5)	46.6 (43.3-49.7)	53.0 (48.8-57.1)	44.2 (40.8-47.5)
	Diabetes mellitus type 2	44.0 (35.9-53.0)	47.2 (39.1-56.1)	34.3 (27.1-42.4)	33.3 (26.3-41.0)
	Neoplasms	7.0 (5.6-8.6)	6.4 (5.0-8.0)	7.6 (6.2-9.2)	6.9 (5.4-8.6)
United States	All causes	15.4 (13.9-16.8)	11.0 (10.1-11.9)	25.4 (23.5-27.5)	17.7 (16.4-18.9)
	Cardiovascular diseases	56.4 (52.5-60.0)	50.9 (47.5-54.3)	54.4 (50.0-58.5)	48.4 (44.7-52.1)
	Diabetes mellitus type 2	51.5 (44.2-59.4)	50.2 (42.6-58.3)	42.0 (35.2-49.6)	41.2 (34.3-48.8)
	Neoplasms	6.8 (5.1-8.7)	6.6 (4.9-8.4)	7.2 (5.5-9.2)	6.8 (5.1-8.7)
Uruguay	All causes	18.7 (17.3-20.2)	11.6 (10.6-12.6)	26.5 (24.9-28.0)	17.1 (16.0-18.3)
	Cardiovascular diseases	59.3 (56.1-62.3)	50.5 (47.4-53.6)	55.7 (52.2-59.1)	47.3 (44.0-50.7)
	Diabetes mellitus type 2	48.2 (39.3-57.2)	47.2 (38.8-56.2)	39.2 (31.2-47.6)	37.4 (29.8-45.6)
	Neoplasms	9.5 (7.8-11.1)	7.2 (6.1-8.4)	10.1 (8.4-11.8)	7.9 (6.6-9.2)
Uzbekistan	All causes	27.2 (24.8-29.5)	31.0 (28.3-33.6)	40.8 (37.8-43.7)	43.7 (40.2-46.9)
	Cardiovascular diseases	67.4 (63.3-71.1)	62.7 (58.2-66.9)	64.5 (59.9-68.7)	60.1 (55.2-64.7)
	Diabetes mellitus type 2	48.0 (37.7-58.6)	45.7 (36.0-55.9)	43.4 (33.3-53.6)	41.2 (32.0-51.0)
	Neoplasms	15.6 (12.0-19.5)	8.1 (6.1-10.2)	16.0 (12.1-19.9)	8.9 (6.9-11.0)

Vanuatu	All causes	23.2 (20.1-26.0)	24.0 (20.4-27.4)	27.8 (24.5-30.9)	28.6 (24.5-32.3)
	Cardiovascular diseases	62.2 (58.0-66.4)	62.8 (58.4-66.9)	59.9 (55.7-63.8)	59.7 (55.3-63.8)
	Diabetes mellitus type 2	44.3 (34.1-55.1)	43.7 (33.3-54.3)	36.9 (27.7-46.9)	36.1 (27.0-46.0)
	Neoplasms	7.1 (4.8-9.3)	6.6 (5.0-8.3)	8.5 (5.8-11.1)	7.5 (5.8-9.6)
Venezuela	All causes	18.4 (16.9-20.1)	14.3 (13.0-15.7)	26.6 (24.7-28.7)	21.9 (20.2-23.7)
	Cardiovascular diseases	58.1 (54.3-61.7)	54.3 (50.3-58.3)	54.4 (50.3-58.3)	50.3 (46.1-54.4)
	Diabetes mellitus type 2	37.7 (28.4-47.9)	38.2 (29.1-48.2)	31.4 (23.1-40.9)	31.1 (23.2-40.0)
	Neoplasms	6.3 (4.5-8.5)	6.1 (5.0-7.3)	7.2 (5.0-9.8)	6.6 (5.4-7.9)
Vietnam	All causes	19.4 (17.6-21.1)	15.0 (13.5-16.4)	25.5 (23.4-27.6)	20.8 (19.2-22.5)
	Cardiovascular diseases	62.4 (58.1-66.6)	50.7 (46.7-54.7)	57.1 (52.7-61.3)	47.2 (43.5-51.0)
	Diabetes mellitus type 2	36.2 (25.8-47.1)	33.0 (23.3-43.4)	28.9 (20.0-38.5)	25.6 (17.5-34.6)
	Neoplasms	14.5 (12.0-16.8)	9.7 (8.1-11.5)	15.2 (12.7-17.5)	10.6 (8.7-12.7)
Virgin Islands, U.S.	All causes	17.2 (15.5-18.9)	13.7 (12.3-15.2)	24.5 (22.4-26.5)	19.1 (17.3-21.0)
	Cardiovascular diseases	51.1 (46.8-55.4)	43.6 (39.6-48.0)	49.6 (45.2-53.9)	42.3 (38.2-46.5)
	Diabetes mellitus type 2	38.8 (30.0-48.5)	41.8 (33.0-50.9)	31.1 (23.2-40.1)	33.2 (25.5-41.4)
	Neoplasms	5.4 (4.5-6.2)	4.6 (3.8-5.5)	6.0 (5.1-7.0)	5.0 (4.1-6.0)
Yemen	All causes	25.8 (20.1-29.6)	23.2 (20.4-25.9)	35.9 (30.6-39.9)	34.3 (31.4-37.4)
	Cardiovascular diseases	65.7 (61.0-70.2)	62.9 (58.3-67.5)	60.4 (55.8-64.9)	57.7 (53.1-62.4)
	Diabetes mellitus type 2	45.8 (35.3-56.6)	44.6 (34.3-55.4)	38.2 (28.6-48.2)	37.1 (27.9-46.8)
	Neoplasms	9.5 (6.2-13.8)	8.8 (6.3-12.0)	9.7 (6.4-14.1)	9.0 (6.5-12.4)
Zambia	All causes	9.6 (8.2-10.9)	9.5 (8.6-10.4)	13.3 (11.6-15.1)	13.2 (12.1-14.6)
	Cardiovascular diseases	57.7 (53.2-62.7)	54.5 (50.2-58.8)	53.8 (49.0-59.0)	51.0 (46.4-55.7)
	Diabetes mellitus type 2	35.8 (25.0-47.0)	37.6 (27.1-48.6)	29.9 (20.8-39.8)	31.0 (21.9-40.5)
	Neoplasms	6.4 (5.1-7.7)	6.1 (5.0-7.2)	7.4 (6.0-8.8)	7.0 (5.8-8.3)
Zimbabwe	All causes	8.0 (6.6-9.4)	10.5 (9.5-11.6)	12.7 (10.8-14.5)	14.6 (13.2-16.2)
	Cardiovascular diseases	51.3 (46.9-55.3)	53.5 (48.8-58.3)	48.7 (44.1-52.9)	50.6 (45.7-55.5)
	Diabetes mellitus type 2	39.0 (28.6-49.8)	39.3 (28.7-50.0)	32.1 (23.1-41.8)	33.3 (24.0-43.3)
	Neoplasms	7.2 (5.3-9.1)	6.4 (4.8-8.1)	7.4 (5.5-9.3)	6.6 (5.0-8.3)

Supplemental Table 9. Number of DALYs and deaths attributable to individual dietary risks at the global level in 2017.			
Risk	Cause	Measure	Attributable burden (95% UI)
Diet high in processed meat	Cardiovascular diseases	DALYs	2267396 (111482-4859265)
Diet high in processed meat	Cardiovascular diseases	Deaths	107436 (5392-229636)
Diet high in processed meat	Diabetes mellitus type 2	DALYs	1073011 (490276-1900503)
Diet high in processed meat	Diabetes mellitus type 2	Deaths	12357 (5670-21238)
Diet high in processed meat	Neoplasms	DALYs	227150 (114474-373121)
Diet high in processed meat	Neoplasms	Deaths	10556 (5227-17462)
Diet high in red meat	Diabetes mellitus type 2	DALYs	934010 (112809-1830814)
Diet high in red meat	Diabetes mellitus type 2	Deaths	8954 (1111-16757)
Diet high in red meat	Neoplasms	DALYs	377910 (89074-677554)
Diet high in red meat	Neoplasms	Deaths	15880 (3763-28651)
Diet high in sodium	Cardiovascular diseases	DALYs	59636303 (27226908-101800198)
Diet high in sodium	Cardiovascular diseases	Deaths	2733272 (1149730-4790587)
Diet high in sodium	Neoplasms	DALYs	7353863 (4116005-11089950)
Diet high in sodium	Neoplasms	Deaths	326681 (174683-506196)
Diet high in sugar-sweetened beverages	Cardiovascular diseases	DALYs	2805297 (0-5704936)
Diet high in sugar-sweetened beverages	Cardiovascular diseases	Deaths	116680 (0-241694)
Diet high in sugar-sweetened beverages	Diabetes mellitus type 2	DALYs	1646811 (851826-2520224)
Diet high in sugar-sweetened beverages	Diabetes mellitus type 2	Deaths	20674 (11449-29717)
Diet high in trans fatty acids	Cardiovascular diseases	DALYs	6162986 (1872151-13762845)
Diet high in trans fatty acids	Cardiovascular diseases	Deaths	257629 (80321-577132)
Diet low in calcium	Neoplasms	DALYs	3893119 (2454580-5483483)
Diet low in calcium	Neoplasms	Deaths	184760 (116030-261773)

Diet low in fiber	Cardiovascular diseases	DALYs	17702354 (9936801-27739836)
Diet low in fiber	Cardiovascular diseases	Deaths	767672 (429297-1205047)
Diet low in fiber	Neoplasms	DALYs	2196966 (1136614-3473788)
Diet low in fiber	Neoplasms	Deaths	105736 (54507-166682)
Diet low in fruits	Cardiovascular diseases	DALYs	52136716 (31226923-76023345)
Diet low in fruits	Cardiovascular diseases	Deaths	2044359 (1193292-3065400)
Diet low in fruits	Diabetes mellitus type 2	DALYs	5901564 (1093714-11294833)
Diet low in fruits	Diabetes mellitus type 2	Deaths	79649 (15015-150890)
Diet low in fruits	Neoplasms	DALYs	6768001 (3582922-10661642)
Diet low in fruits	Neoplasms	Deaths	299440 (155293-474265)
Diet low in legumes	Cardiovascular diseases	DALYs	10989971 (4389661-18665383)
Diet low in legumes	Cardiovascular diseases	Deaths	534767 (213706-909458)
Diet low in milk	Neoplasms	DALYs	2721750 (964644-4729928)
Diet low in milk	Neoplasms	Deaths	126069 (44918-219524)
Diet low in nuts and seeds	Cardiovascular diseases	DALYs	42621808 (27297366-58673133)
Diet low in nuts and seeds	Cardiovascular diseases	Deaths	1959621 (1228326-2753926)
Diet low in nuts and seeds	Diabetes mellitus type 2	DALYs	7309305 (3414823-12144930)
Diet low in nuts and seeds	Diabetes mellitus type 2	Deaths	102901 (49743-160038)
Diet low in polyunsaturated fatty acids	Cardiovascular diseases	DALYs	17881679 (7770061-29034837)
Diet low in polyunsaturated fatty acids	Cardiovascular diseases	Deaths	799449 (342919-1310343)
Diet low in seafood omega-3 fatty acids	Cardiovascular diseases	DALYs	32373396 (15170010-52690324)
Diet low in seafood omega-3 fatty acids	Cardiovascular diseases	Deaths	1444907 (667199-2383250)
Diet low in vegetables	Cardiovascular diseases	DALYs	34210780 (17743947-55686795)

Diet low in vegetables	Cardiovascular diseases	Deaths	1462367 (731753-2403760)
Diet low in whole grains	Cardiovascular diseases	DALYs	70307644 (49026457-93031392)
Diet low in whole grains	Cardiovascular diseases	Deaths	2896518 (1976602-3909984)
Diet low in whole grains	Diabetes mellitus type 2	DALYs	12191579 (6399473-19433561)
Diet low in whole grains	Diabetes mellitus type 2	Deaths	169071 (95103-258504)

Supplemental Table 10. Age-standardised DALY rate and mortality rate (per 100, 000 population) attributable to individual dietary risks across SDI levels in 2017.

Risk	Location	Measure	Attributable burden (95% UI)
Diet high in processed meat	High SDI	DALYs	236 (95-377)
Diet high in processed meat	High-middle SDI	DALYs	78 (15-178)
Diet high in processed meat	Low SDI	DALYs	6 (0-39)
Diet high in processed meat	Low-middle SDI	DALYs	13 (0-63)
Diet high in processed meat	Middle SDI	DALYs	31 (8-82)
Diet high in processed meat	High SDI	Deaths	8 (2-14)
Diet high in processed meat	High-middle SDI	Deaths	3 (0-8)
Diet high in processed meat	Low SDI	Deaths	0 (0-1)
Diet high in processed meat	Low-middle SDI	Deaths	0 (0-2)
Diet high in processed meat	Middle SDI	Deaths	1 (0-3)
Diet high in red meat	High SDI	DALYs	59 (22-101)
Diet high in red meat	High-middle SDI	DALYs	46 (14-91)
Diet high in red meat	Low SDI	DALYs	0 (0-0)
Diet high in red meat	Low-middle SDI	DALYs	5 (1-10)
Diet high in red meat	Middle SDI	DALYs	22 (6-48)
Diet high in red meat	High SDI	Deaths	1 (0-2)
Diet high in red meat	High-middle SDI	Deaths	1 (0-2)
Diet high in red meat	Low SDI	Deaths	0 (0-0)
Diet high in red meat	Low-middle SDI	Deaths	0 (0-0)
Diet high in red meat	Middle SDI	Deaths	0 (0-1)
Diet high in sodium	High SDI	DALYs	652 (174-1290)
Diet high in sodium	High-middle SDI	DALYs	2471 (1208-3906)
Diet high in sodium	Low SDI	DALYs	906 (60-2342)
Diet high in sodium	Low-middle SDI	DALYs	1441 (373-3035)
Diet high in sodium	Middle SDI	DALYs	2389 (1187-3736)
Diet high in sodium	High SDI	Deaths	32 (8-66)
Diet high in sodium	High-middle SDI	Deaths	122 (53-201)
Diet high in sodium	Low SDI	Deaths	44 (3-113)
Diet high in sodium	Low-middle SDI	Deaths	67 (17-144)
Diet high in sodium	Middle SDI	Deaths	117 (53-189)
Diet high in sugar-sweetened beverages	High SDI	DALYs	156 (51-281)
Diet high in sugar-sweetened beverages	High-middle SDI	DALYs	116 (29-231)
Diet high in sugar-sweetened beverages	Low SDI	DALYs	15 (4-33)
Diet high in sugar-sweetened beverages	Low-middle SDI	DALYs	53 (14-106)
Diet high in sugar-sweetened beverages	Middle SDI	DALYs	112 (43-198)
Diet high in sugar-sweetened beverages	High SDI	Deaths	5 (0-10)
Diet high in sugar-sweetened beverages	High-middle SDI	Deaths	4 (0-8)
Diet high in sugar-sweetened beverages	Low SDI	Deaths	0 (0-1)
Diet high in sugar-sweetened beverages	Low-middle SDI	Deaths	2 (0-4)
Diet high in sugar-sweetened beverages	Middle SDI	Deaths	3 (1-7)
Diet high in trans fatty acids	High SDI	DALYs	101 (39-197)
Diet high in trans fatty acids	High-middle SDI	DALYs	77 (12-224)
Diet high in trans fatty acids	Low SDI	DALYs	187 (43-425)

Diet high in trans fatty acids	Low-middle SDI	DALYs	325 (113-647)
Diet high in trans fatty acids	Middle SDI	DALYs	114 (30-273)
Diet high in trans fatty acids	High SDI	Deaths	5 (2-10)
Diet high in trans fatty acids	High-middle SDI	Deaths	4 (1-12)
Diet high in trans fatty acids	Low SDI	Deaths	8 (2-18)
Diet high in trans fatty acids	Low-middle SDI	Deaths	13 (5-27)
Diet high in trans fatty acids	Middle SDI	Deaths	5 (1-13)
Diet low in calcium	High SDI	DALYs	92 (50-140)
Diet low in calcium	High-middle SDI	DALYs	101 (62-144)
Diet low in calcium	Low SDI	DALYs	90 (62-121)
Diet low in calcium	Low-middle SDI	DALYs	81 (54-110)
Diet low in calcium	Middle SDI	DALYs	93 (62-126)
Diet low in calcium	High SDI	Deaths	5 (3-7)
Diet low in calcium	High-middle SDI	Deaths	5 (3-7)
Diet low in calcium	Low SDI	Deaths	4 (3-6)
Diet low in calcium	Low-middle SDI	Deaths	4 (3-5)
Diet low in calcium	Middle SDI	Deaths	4 (3-6)
Diet low in fiber	High SDI	DALYs	256 (148-392)
Diet low in fiber	High-middle SDI	DALYs	491 (278-762)
Diet low in fiber	Low SDI	DALYs	582 (337-893)
Diet low in fiber	Low-middle SDI	DALYs	607 (336-950)
Diet low in fiber	Middle SDI	DALYs	488 (286-740)
Diet low in fiber	High SDI	Deaths	14 (8-21)
Diet low in fiber	High-middle SDI	Deaths	26 (15-41)
Diet low in fiber	Low SDI	Deaths	25 (14-38)
Diet low in fiber	Low-middle SDI	Deaths	26 (14-41)
Diet low in fiber	Middle SDI	Deaths	23 (14-35)
Diet low in fruits	High SDI	DALYs	645 (366-974)
Diet low in fruits	High-middle SDI	DALYs	1606 (950-2351)
Diet low in fruits	Low SDI	DALYs	2169 (1416-2994)
Diet low in fruits	Low-middle SDI	DALYs	2142 (1327-3035)
Diet low in fruits	Middle SDI	DALYs	1628 (985-2335)
Diet low in fruits	High SDI	Deaths	25 (14-40)
Diet low in fruits	High-middle SDI	Deaths	69 (40-104)
Diet low in fruits	Low SDI	Deaths	86 (55-121)
Diet low in fruits	Low-middle SDI	Deaths	85 (51-123)
Diet low in fruits	Middle SDI	Deaths	67 (40-98)
Diet low in legumes	High SDI	DALYs	176 (70-298)
Diet low in legumes	High-middle SDI	DALYs	435 (178-723)
Diet low in legumes	Low SDI	DALYs	170 (63-306)
Diet low in legumes	Low-middle SDI	DALYs	163 (57-311)
Diet low in legumes	Middle SDI	DALYs	272 (107-469)
Diet low in legumes	High SDI	Deaths	10 (4-16)
Diet low in legumes	High-middle SDI	Deaths	24 (10-40)
Diet low in legumes	Low SDI	Deaths	7 (3-13)
Diet low in legumes	Low-middle SDI	Deaths	8 (3-15)
Diet low in legumes	Middle SDI	Deaths	14 (5-24)

Diet low in milk	High SDI	DALYs	66 (22-121)
Diet low in milk	High-middle SDI	DALYs	77 (28-133)
Diet low in milk	Low SDI	DALYs	57 (20-97)
Diet low in milk	Low-middle SDI	DALYs	54 (19-92)
Diet low in milk	Middle SDI	DALYs	62 (21-106)
Diet low in milk	High SDI	Deaths	3 (1-6)
Diet low in milk	High-middle SDI	Deaths	4 (1-6)
Diet low in milk	Low SDI	Deaths	3 (1-5)
Diet low in milk	Low-middle SDI	Deaths	3 (1-4)
Diet low in milk	Middle SDI	Deaths	3 (1-5)
Diet low in nuts and seeds	High SDI	DALYs	534 (343-752)
Diet low in nuts and seeds	High-middle SDI	DALYs	1245 (817-1722)
Diet low in nuts and seeds	Low SDI	DALYs	1538 (1028-2080)
Diet low in nuts and seeds	Low-middle SDI	DALYs	1722 (1139-2361)
Diet low in nuts and seeds	Middle SDI	DALYs	1245 (833-1688)
Diet low in nuts and seeds	High SDI	Deaths	25 (16-36)
Diet low in nuts and seeds	High-middle SDI	Deaths	64 (40-90)
Diet low in nuts and seeds	Low SDI	Deaths	64 (41-88)
Diet low in nuts and seeds	Low-middle SDI	Deaths	73 (47-102)
Diet low in nuts and seeds	Middle SDI	Deaths	56 (36-77)
Diet low in polyunsaturated fatty acids	High SDI	DALYs	114 (45-202)
Diet low in polyunsaturated fatty acids	High-middle SDI	DALYs	420 (175-703)
Diet low in polyunsaturated fatty acids	Low SDI	DALYs	652 (278-1046)
Diet low in polyunsaturated fatty acids	Low-middle SDI	DALYs	719 (308-1157)
Diet low in polyunsaturated fatty acids	Middle SDI	DALYs	443 (189-719)
Diet low in polyunsaturated fatty acids	High SDI	Deaths	7 (3-12)
Diet low in polyunsaturated fatty acids	High-middle SDI	Deaths	24 (10-40)
Diet low in polyunsaturated fatty acids	Low SDI	Deaths	29 (12-47)
Diet low in polyunsaturated fatty acids	Low-middle SDI	Deaths	33 (14-53)
Diet low in polyunsaturated fatty acids	Middle SDI	Deaths	22 (9-36)
Diet low in seafood omega-3 fatty acids	High SDI	DALYs	264 (114-454)
Diet low in seafood omega-3 fatty acids	High-middle SDI	DALYs	758 (344-1272)
Diet low in seafood omega-3 fatty acids	Low SDI	DALYs	1127 (531-1801)
Diet low in seafood omega-3 fatty acids	Low-middle SDI	DALYs	1261 (594-2026)
Diet low in seafood omega-3 fatty acids	Middle SDI	DALYs	795 (373-1285)
Diet low in seafood omega-3 fatty acids	High SDI	Deaths	15 (6-25)
Diet low in seafood omega-3 fatty acids	High-middle SDI	Deaths	42 (19-70)
Diet low in seafood omega-3 fatty acids	Low SDI	Deaths	50 (23-81)
Diet low in seafood omega-3 fatty acids	Low-middle SDI	Deaths	56 (26-92)
Diet low in seafood omega-3 fatty acids	Middle SDI	Deaths	39 (18-64)
Diet low in vegetables	High SDI	DALYs	334 (156-566)
Diet low in vegetables	High-middle SDI	DALYs	659 (284-1188)
Diet low in vegetables	Low SDI	DALYs	1469 (805-2246)
Diet low in vegetables	Low-middle SDI	DALYs	1206 (612-1964)
Diet low in vegetables	Middle SDI	DALYs	886 (446-1450)
Diet low in vegetables	High SDI	Deaths	17 (8-29)
Diet low in vegetables	High-middle SDI	Deaths	35 (15-63)

Diet low in vegetables	Low SDI	Deaths	63 (34-97)
Diet low in vegetables	Low-middle SDI	Deaths	54 (27-88)
Diet low in vegetables	Middle SDI	Deaths	41 (20-69)
Diet low in whole grains	High SDI	DALYs	947 (664-1275)
Diet low in whole grains	High-middle SDI	DALYs	2246 (1611-2942)
Diet low in whole grains	Low SDI	DALYs	2221 (1533-2988)
Diet low in whole grains	Low-middle SDI	DALYs	2737 (1940-3617)
Diet low in whole grains	Middle SDI	DALYs	1982 (1385-2648)
Diet low in whole grains	High SDI	Deaths	36 (24-50)
Diet low in whole grains	High-middle SDI	Deaths	98 (67-131)
Diet low in whole grains	Low SDI	Deaths	85 (57-116)
Diet low in whole grains	Low-middle SDI	Deaths	107 (74-144)
Diet low in whole grains	Middle SDI	Deaths	80 (55-109)

Supplemental Figure 1. Flowchart of the estimation process of dietary risk factors.

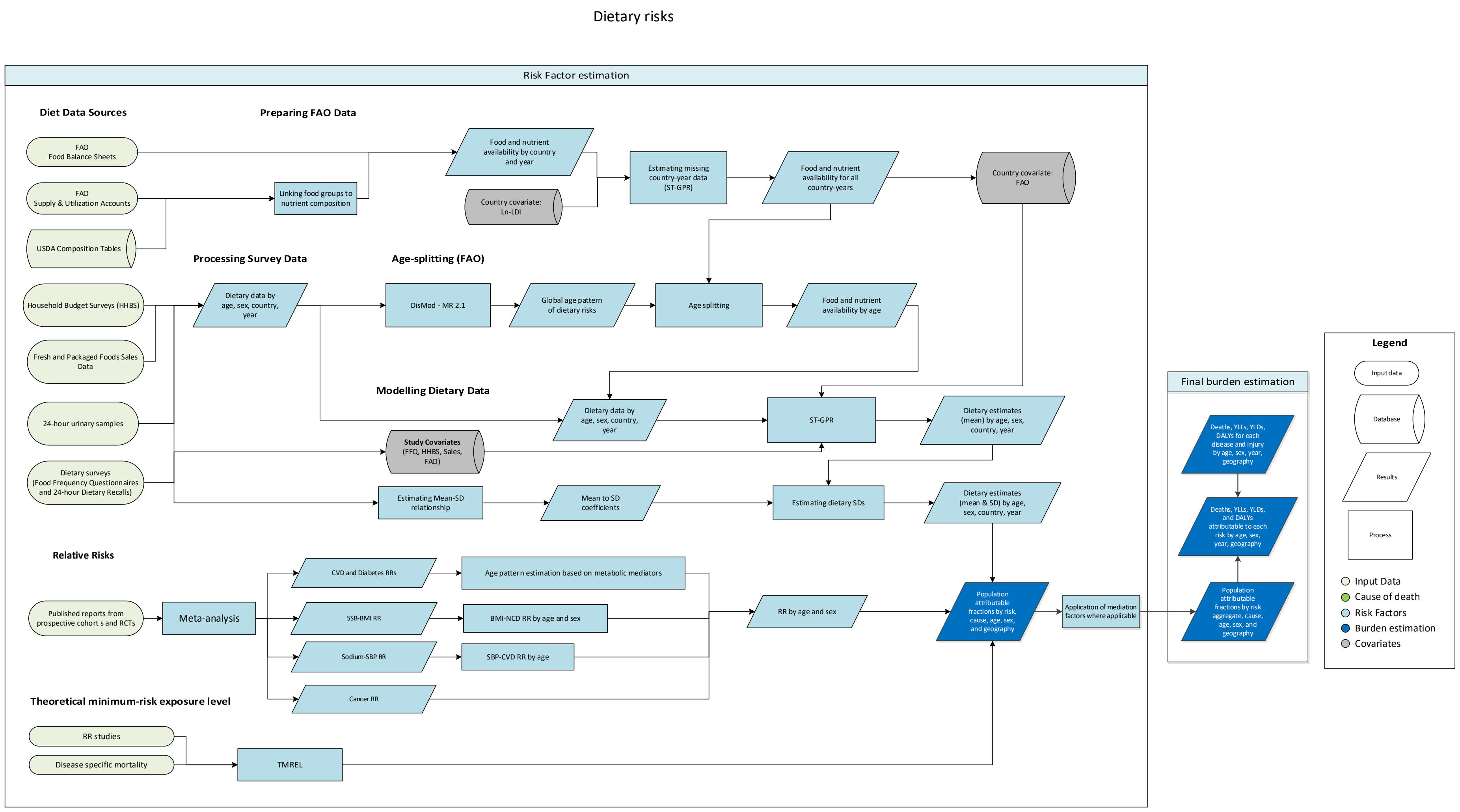

Supplemental Figure 2. Flowchart of the analytic process for Spatiotemporal Gaussian Process Regression.

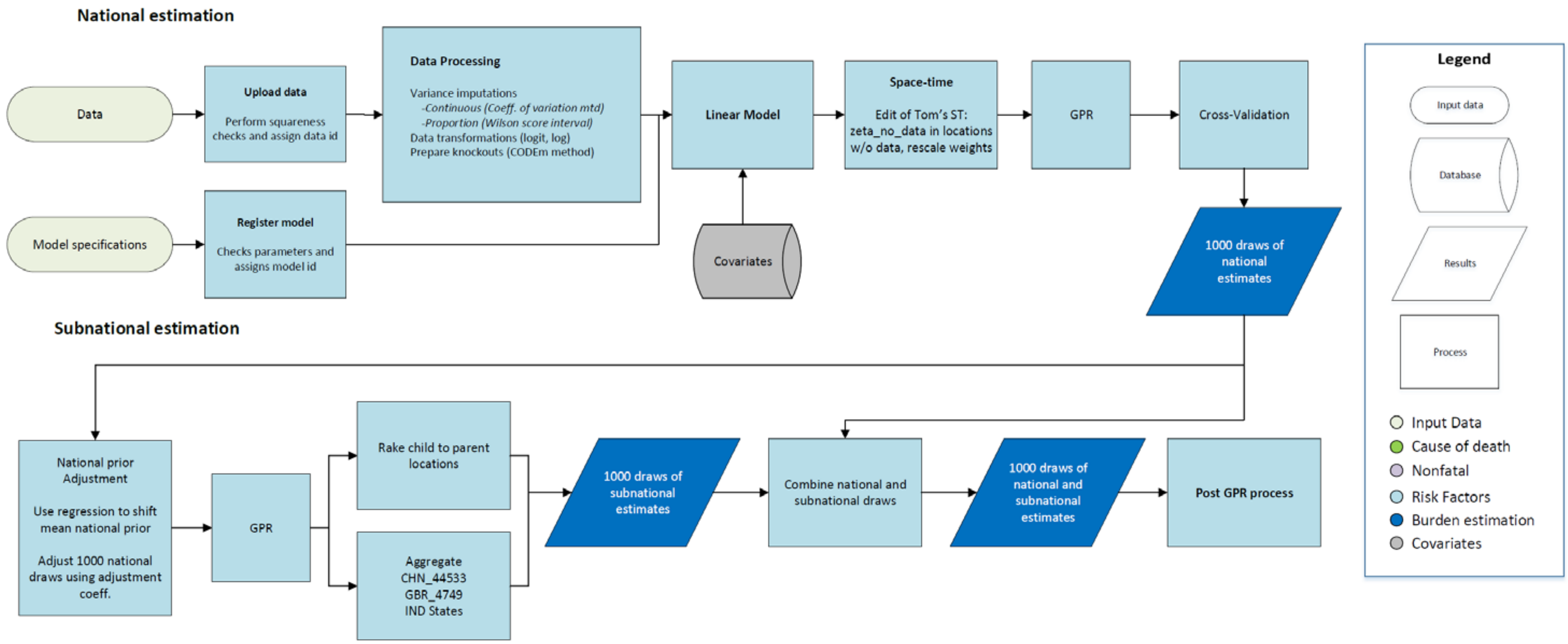

Supplemental Figure 3. Ranking of dietary risk factors according to their attributable deaths at the global and regional level.

	E Asia	SE Asia	Oceania	C Asia	C Europe	E Europe	HI Asia Pac	Australasia	W Europe	S Latin Am	HI N Am	Caribbean	Andean Latin Am	Central Latin Am	Trop Latin Am	MENA	S Asia	C Sub-Sah Africa	E Sub-Sah Africa	S Sub-Sah Africa	W Sub-Sah Africa	**Earth**
High sodium	1	1	6	4	2	4	1	6	3	2	3	6	2	5	2	5	6	8	5	5	6	1
Low whole grains	2	3	1	1	1	1	2	1	1	1	1	1	1	2	1	1	1	1	1	2	1	2
Low fruit	3	2	2	2	4	3	3	3	4	5	4	5	5	4	5	4	2	2	2	1	2	3
Low nuts and seeds	4	5	4	3	3	2	4	2	2	3	2	2	3	1	3	2	3	4	4	3	4	4
Low vegetables	6	4	3	9	6	6	5	4	5	4	5	3	4	3	4	7	5	3	3	4	3	5
Low omega-3	5	6	5	5	5	5	12	7	6	8	6	4	6	6	6	3	4	5	6	6	5	6
Low fiber	7	7	9	8	7	9	6	5	7	6	7	7	7	9	7	8	8	7	8	8	8	7
Low PUFA	8	8	7	7	8	8	8	8	9	9	13	8	8	8	9	6	7	6	7	9	7	8
Low legumes	9	9	8	6	9	7	11	9	8	7	10	12	9	11	15	10	10	9	11	7	10	9
High trans fat	12	13	10	10	13	11	14	11	14	15	9	13	10	10	12	9	9	12	12	13	13	10
Low calcium	10	10	11	13	11	12	7	10	11	11	12	9	11	13	10	12	11	10	9	10	9	11
High sweetened beverages	13	12	12	11	10	13	13	12	12	10	11	10	12	7	8	11	13	13	13	11	12	12
High processed meat	15	14	14	12	14	10	10	13	10	13	8	14	14	12	14	14	14	14	14	14	14	13
Low milk	11	11	13	14	12	14	9	14	13	12	14	11	13	14	11	13	12	11	10	12	11	14
High red meat	14	15	15	15	15	15	15	15	15	14	15	15	15	15	13	15	15	15	15	15	15	15

Supplemental Figure 4. Ranking of dietary risk factors according to their attributable DALYs at the global and regional level.

	E Asia	SE Asia	Oceania	C Asia	C Europe	E Europe	HI Asia Pac	Australasia	W Europe	S Latin Am	HI N Am	Caribbean	Andean Latin Am	Central Latin Am	Trop Latin Am	MENA	S Asia	C Sub-Sah Africa	E Sub-Sah Africa	S Sub-Sah Africa	W Sub-Sah Africa	**Earth**
Low whole grains	2	1	1	1	1	1	2	1	1	1	1	1	1	2	1	1	1	1	1	2	1	1
High sodium	1	3	6	4	2	4	1	6	4	3	2	7	4	6	4	5	6	8	6	5	6	2
Low fruit	3	2	2	2	3	2	3	2	2	4	3	4	5	3	5	3	2	2	2	1	2	3
Low nuts and seeds	4	4	3	3	4	3	4	3	3	2	4	2	2	1	2	2	3	3	4	3	4	4
Low vegetables	5	5	4	9	6	6	5	4	5	5	6	3	3	4	3	7	5	4	3	4	3	5
Low omega-3	6	6	5	5	5	5	13	7	6	8	7	5	6	7	6	4	4	5	5	6	5	6
Low fiber	7	7	9	8	7	9	6	5	7	6	8	6	7	8	7	8	8	7	8	7	8	7
Low PUFA	9	8	7	7	9	8	10	8	9	10	15	8	8	10	9	6	7	6	7	9	7	8
Low legumes	8	9	8	6	8	7	12	9	8	7	11	12	9	12	15	10	10	9	11	8	10	9
High trans fat	14	13	10	11	14	11	14	14	15	15	10	13	11	9	12	9	9	13	13	14	13	10
High sweetened beverages	13	11	11	10	10	12	11	12	11	9	9	9	10	5	8	11	13	12	12	10	12	11
Low calcium	10	10	12	13	11	13	7	10	12	11	12	10	12	13	10	12	11	10	9	11	9	12
High processed meat	15	14	14	12	13	10	9	13	10	12	5	14	14	11	14	14	14	14	14	15	14	13
Low milk	11	12	13	14	12	14	8	15	13	14	14	11	13	14	13	13	12	11	10	12	11	14
High red meat	12	15	15	15	15	15	15	11	14	13	13	15	15	15	11	15	15	15	15	13	15	15

Supplemental Figure 5. Ranking of dietary risk factors according to their attributable deaths among the world's 20 most populous countries.

	**China**	India	USA	Indonesia	Pakistan	Brazil	Nigeria	Bangladesh	Russian Federation	Japan	Mexico	Philippines	Ethiopia	Egypt	Vietnam	Germany	Iran	Congo DR	Turkey	Thailand
High sodium	1	6	3	1	5	2	7	3	4	1	8	1	6	7	1	3	4	9	4	1
Low whole grains	2	1	1	3	1	1	1	4	1	2	3	2	2	1	3	1	1	2	1	2
Low fruit	3	2	4	2	2	5	2	1	3	3	4	5	1	5	2	4	5	1	6	5
Low nuts and seeds	4	3	2	5	4	3	5	5	2	4	1	3	4	2	4	2	2	4	2	4
Low omega-3	5	4	6	6	6	6	4	6	6	14	6	6	5	3	5	6	3	5	3	6
Low vegetables	6	5	5	4	3	4	3	2	5	5	2	4	3	11	7	5	10	3	10	3
Low fiber	7	8	8	7	8	7	8	7	9	6	10	7	8	8	6	7	7	7	7	7
Low PUFA	8	7	14	8	7	9	6	8	8	8	9	8	7	6	8	9	8	6	5	8
Low legumes	9	12	10	9	9	15	14	9	7	11	12	9	13	9	10	8	9	8	12	9
Low calcium	10	10	12	10	11	10	9	11	12	7	13	11	9	12	9	13	12	10	9	10
Low milk	11	11	13	11	12	11	10	12	14	9	14	12	10	13	11	14	13	11	11	11
High trans fat	12	9	9	12	10	12	12	10	11	13	7	13	11	4	12	12	6	12	13	13
High sweetened beverages	13	13	11	13	14	8	11	14	13	12	5	10	12	10	13	11	11	13	8	12
High red meat	14	15	15	15	15	13	15	15	15	15	15	15	15	15	14	15	15	15	15	15
High processed meat	15	14	7	14	13	14	13	13	10	10	11	14	14	14	15	10	14	14	14	14

Supplemental Figure 6. Ranking of dietary risk factors according to their attributable DALYs among the world's 20 most populous countries.

	**China**	India	USA	Indonesia	Pakistan	Brazil	Nigeria	Bangladesh	Russian Federation	Japan	Mexico	Philippines	Ethiopia	Egypt	Vietnam	Germany	Iran	Congo DR	Turkey	Thailand
High sodium	1	6	2	3	5	4	7	4	4	1	8	2	6	7	2	4	4	9	5	1
Low whole grains	2	1	1	2	1	1	1	3	1	2	4	1	1	1	3	1	1	1	1	2
Low fruit	3	2	3	1	2	5	2	1	2	3	3	5	2	5	1	2	5	2	4	4
Low nuts and seeds	4	3	4	5	3	2	4	5	3	4	1	3	4	2	4	3	2	4	2	5
Low vegetables	5	5	6	4	4	3	3	2	5	5	5	4	3	11	7	5	10	3	11	3
Low omega-3	6	4	7	6	6	6	5	6	6	15	6	6	5	3	5	6	3	5	3	7
Low fiber	7	8	8	7	7	7	8	7	8	7	11	7	8	8	6	7	7	7	7	6
Low legumes	8	12	11	9	9	15	14	9	7	12	14	9	14	10	10	8	9	8	13	9
Low PUFA	9	7	15	8	8	9	6	8	9	10	10	8	7	6	8	10	8	6	6	8
Low calcium	10	10	13	10	11	11	9	11	13	6	12	11	9	12	9	12	12	10	9	11
Low milk	11	11	14	12	12	13	10	12	14	9	15	12	10	13	11	14	13	11	10	12
High red meat	12	15	12	15	15	10	15	15	15	14	13	15	15	15	12	15	15	15	15	15
High sweetened beverages	13	13	9	13	14	8	11	14	11	11	2	10	12	9	13	11	11	13	8	10
High trans fat	14	9	10	11	10	12	12	10	12	13	7	13	11	4	14	13	6	12	12	13
High processed meat	15	14	5	14	13	14	13	13	10	8	9	14	13	14	15	9	14	14	14	14
